# Design, Synthesis, and Evaluation of Braylin Derivatives as Novel PDE4 Inhibitors with Anti-Inflammatory Effects

**DOI:** 10.3390/pharmaceutics18050516

**Published:** 2026-04-23

**Authors:** Yongdan Guo, Xue Wang, Feng Zhang, Tianshen Zheng, Zhuo Chen, Sen Wang, Guofeng Yang, Haibo Wang, Wenbo Yin, Shuheng Huang, Hai-Bin Luo, Yi-You Huang, Deyan Wu

**Affiliations:** 1 Key Laboratory of Tropical Biological Resources of Ministry of Education, School of Pharmaceutical Sciences, Hainan University, Haikou 570228, Chinahbluo@hainanu.edu.cn (H.-B.L.); 2 School of Pharmaceutical Sciences, Song Li’s Academician Workstation of Hainan University, Yazhou Bay, Sanya 572000, China

**Keywords:** PDE4, braylin, structural optimization, anti-inflammatory

## Abstract

**Background/Objectives**: PDE4 is a key regulator of cAMP signaling and a clinically validated anti-inflammatory target; however, the use of PDE4 inhibitors is often limited by adverse effects such as nausea, vomiting, and diarrhea. The natural compound braylin was previously identified as a novel PDE4 inhibitor scaffold, exhibiting an IC_50_ of 0.96 µM. Using the PDE4–braylin co-crystal structure, we conducted structure-based design and optimization to enhance its potency. **Methods**: A series of novel braylin derivatives was synthesized and characterized. Their inhibitory activities against PDE4D were evaluated via enzymatic assays, and binding thermodynamics were analyzed by isothermal titration calorimetry (ITC). Molecular modeling was used to predict binding modes, and anti-inflammatory effects were assessed in LPS-stimulated macrophages. **Results**: Structure-guided optimization yielded lead compound **L27**, which showed significantly improved PDE4D inhibition (IC_50_ = 67 nM) and high-affinity binding *(K_d_* = 45 nM) as confirmed by ITC. **L27** also exhibited remarkable selectivity against PDE isoforms. Molecular simulations highlighted key interactions with Gln369 and hydrophobic residues in the PDE4 active site. In cellular assays, **L27** dose-dependently suppressed LPS-induced inflammation in macrophages at non-cytotoxic concentrations with efficacy comparable to roflumilast. **Conclusions**: We developed **L27**, a potent and selective PDE4 inhibitor derived from natural braylin. It demonstrated promising in vitro anti-inflammatory activity and represents a valuable lead for further therapeutic development.

## 1. Introduction

Phosphodiesterases (PDEs) are a class of key enzymes responsible for the hydrolysis of the second messengers cyclic adenosine monophosphate (cAMP) and cyclic guanosine monophosphate (cGMP), thereby playing critical regulatory roles in a wide range of cellular signal transduction pathways [1]. Among them, phosphodiesterase 4 (PDE4) specifically catalyzes the degradation of cAMP and is widely expressed in immune cells and fibroblasts [2]. Aberrant activation or overexpression of PDE4 has been closely associated with the onset and progression of various diseases, including chronic and inflammatory disorders such as asthma and chronic obstructive pulmonary disease (COPD), autoimmune diseases, neurological disorders, and fibrosis-related diseases such as idiopathic pulmonary fibrosis (IPF) [3]. Consequently, PDE4 inhibition represents a well-established therapeutic strategy for treating inflammatory diseases.

**Figure 1:**
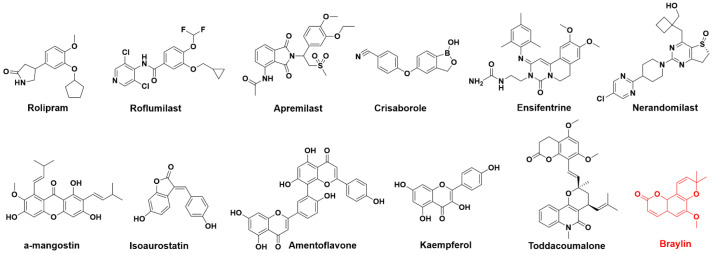
The structures of approved PDE4 inhibitors, reported natural PDE4 inhibitors, and the hit compound Braylin (highlighted in red).

To date, several PDE4 inhibitors have been approved by the FDA for clinical use, as shown in Figure 1, including roflumilast (oral, for severe COPD), apremilast (systemic, for psoriatic arthritis), crisaborole (topical, for mild-to-moderate atopic dermatitis), ensifentrine (COPD), and nerandomilast (IPF) [4,5,6,7,8]. However, most currently available PDE4 inhibitors are associated with a high incidence of adverse effects—notably nausea, vomiting, and diarrhea—which significantly limit their long-term or widespread clinical application [9]. These limitations may be partly attributed to structural features such as common catechol moieties. In addition, it has been reported that these adverse effects are closely related to PDE4 subtype selectivity, especially PDE4D inhibition. Thus, developing structurally diverse PDE4 inhibitors with improved efficacy and reduced side effects remains an important research objective.

Natural products have long served as an important source of innovative drug discovery. In the development of enzyme inhibitors, natural products offer unique advantages due to their structural diversity, favorable biocompatibility, and safety profiles shaped by long-term evolutionary selection, making them ideal lead scaffolds for the discovery of novel PDE4 inhibitors [10,11]. For instance, recent studies have reported that a series of PDE4 inhibitors derived from natural α-mangostin demonstrated superior safety profiles compared to conventional agents such as rolipram or roflumilast, underscoring the promise of natural products in this field [12,13]. Furthermore, other naturally derived compounds and their derivatives—including amentoflavone, isoaurostatin, and kaempferol, which featured novel structural scaffolds—had also been identified as potent PDE4 inhibitors with significant anti-inflammatory activity (Figure 1) [14,15,16,17,18]. These findings further motivate the ongoing exploration of natural products as sources of PDE4 inhibitors with minimal adverse effects.

The root and stem bark of *Toddalia asiatica* have been traditionally used in Chinese and ethnic medicine for the treatment of rheumatic pain, traumatic injury, gastric pain, and toothache, suggesting a potential role in inflammatory regulation [19]. In our previous activity-guided phytochemical study of this plant, a series of prenylated coumarins were isolated and structurally characterized, among which toddacoumalone and its structurally optimized derivatives exhibited notable PDE4 inhibitory activity as well as promising efficacy in relevant inflammatory disease models [20,21]. Notably, a coumarin-type natural product, braylin, also exhibited appreciable inhibitory activity against PDE4 with an IC_50_ of 0.96 μM [19]. Its simple structure, combined with this promising activity, highlighted braylin’s potential as a natural hit compound for PDE4 inhibitor discovery. More recently, we successfully determined the co-crystal structure of the PDE4–braylin complex, which unambiguously revealed the key interaction patterns between braylin and the catalytic pocket of PDE4. Leveraging this structural insight, we performed a structure-based optimization campaign on the braylin scaffold. A series of derivatives was designed and synthesized to explore structure–activity relationships and enhance inhibitory potency. Among these, compound **L27** was identified as a significantly improved PDE4 inhibitor, with an IC_50_ value of 67 nM, representing a substantial enhancement over the parent compound. Furthermore, the binding mode and anti-inflammatory effects of **L27** were subsequently investigated.

## 2. Materials and Methods

### 2.1. Chemicals and Instrumentation

All starting materials and reagents were purchased from commercial suppliers (Adamas (Basel, Switzerland), Bide (Shanghai, China), Energy (Bingham, UK), Bepharm (Arlington Heights, IL, USA), Meryer (Shanghai, China), and Sigma-Aldrich (St. Louis, MO, USA)) and used directly without further purification. Chemical HG/T2354-92 silica gel (200–300 mesh, Xinnuo, Qingdao, China) was used for chromatography, and silica gel plates with fluorescence F254 (0.25 mm, Xinnuo, Qingdao, China) were used for thin-layer chromatography (TLC) analysis. Reactions requiring anhydrous conditions were performed under argon or in a calcium chloride tube. Nuclear magnetic resonance (NMR) spectroscopy was recorded at room temperature on a Bruker AVANCE III 400 instrument (Billerica, MA, USA) in CDCl3 or DMSO-d6 solutions with tetramethylsilane (TMS) as an internal standard. The following abbreviations are used: s (singlet), br (broad signal), d (doublet), dd (doublet of doublets), t (triplet), q (quartet), and m (multiplet). Coupling constants were reported in Hz. The purity of compounds was determined by reverse-phase high-performance liquid chromatography (HPLC) analysis, confirming that it was over 95%. HPLC system: SHIMADZU LC-2030 Plus; Column: SHIMADZU C18-AQ (Kyoto, Japan), 5.0 μm, 4.6 × 250 mm (HSS); Detector: SPD-20A UV/VIS; Detection wavelength: 254 nm; Mobile phase: CH3CN in MeOH (70%, *v*/*v*); Temperature: 30 °C; Flow rate: 0.8 mL/min.

### 2.2. In Vitro Enzymatic Assay

Protein preparation. The catalytic domain of human PDE4D2 (residues 86–413) was prepared as previously described [22]. Briefly, the gene fragment was cloned into a pET15b vector and introduced into *E. coli* BL21 (CodonPlus) cells for expression. Cell cultures were grown at 37 °C in LB medium to an OD_600_ of ~0.7, at which point protein production was induced with 0.1 mM IPTG. Following induction, cultures were further incubated at 16 °C for 24 h before harvesting. The recombinant His-tagged protein was first captured on a Ni-NTA affinity column, followed by sequential purification using anion-exchange (Q column) and size-exclusion (Superdex 100, Cytiva, Shanghai, China) chromatography. The final protein purity was confirmed to exceed 95% by SDS-PAGE analysis. The catalytic domains of various other PDE family members (e.g., PDE1B2, PDE2A, PDE3A, PDE5A1, PDE7A1, PDE8A1, PDE9A2, and PDE10A2) were expressed and purified using analogous protocols [23,24,25,26].

Enzymatic activity and inhibitory assays. Phosphodiesterase activity was measured using a standard scintillation proximity assay with ^3^H-labeled cAMP or cGMP as the substrate. Reactions were conducted in a buffer containing 20 mM Tris-HCl (pH 7.5), 10 mM MgCl_2_ (or 4 mM MnCl_2_), 1 mM DTT, and 10–30 nM of the respective radiolabeled cAMP or cGMP. After a 15 min incubation at room temperature, reactions were terminated by adding 0.2 M ZnSO_4_. The hydrolyzed product (^3^H-AMP or ^3^H-GMP) was then precipitated with 0.2 N Ba(OH)_2_, allowing the unreacted substrate to remain in the supernatant. The radioactivity of the supernatant, corresponding to residual cyclic nucleotide, was quantified using a PerkinElmer 4910 scintillation counter (PerkinElmer, Shanghai, China). For inhibitor testing, dose–response curves were generated using a minimum of eight compound concentrations. IC_50_ values were determined via nonlinear regression analysis of data from three independent.

### 2.3. Isothermal Titration Calorimetry Test

For ITC experiments, the buffer composition of the PDE4D protein and the ligand (**L27**) was carefully matched. The protein sample was subjected to overnight dialysis at 4 °C against a buffer consisting of 20 mM Tris (pH 7.5), 50 mM NaCl, 1 mM EDTA, and 1 mM β-mercaptoethanol. **L27** was directly dissolved in the final dialysis buffer to prepare the ligand solution. Protein concentration was assessed by UV spectrophotometry using a NanoDrop instrument (Wilmington, DE, USA). All titrations were conducted at 25 °C using a MicroCal PEAQ-ITC calorimeter (Malvern Panalytical, Malvern, UK). In a typical experiment, the ligand solution was titrated into the protein sample cell with an initial injection of 0.2 µL, followed by 18 subsequent injections of 2 µL each. A spacing interval of 120 s was maintained between injections to allow for signal equilibration. The resulting thermograms were analyzed with the instrument’s proprietary software (MicroCal PEAQ-ITC Analysis, v 1.41, Northampton, MA, USA) by fitting the data to a single-site binding model, from which the dissociation constant (*K_d_*), enthalpy change (Δ*H*), and binding stoichiometry (n) were derived. The Gibbs free energy change (Δ*G*) and the entropic contribution (*T*Δ*S*) were subsequently calculated using the standard thermodynamic relationships.

### 2.4. Crystallization, Data Collection, and Structure Determination

The PDE4D–PDME complex was crystallized via the hanging-drop vapor-diffusion technique at 4 °C. Prior to setup, purified PDE4D2 (10 mg/mL in 20 mM Tris-HCl, pH 7.5, 50 mM NaCl, 1 mM EDTA, 1 mM β-mercaptoethanol) was incubated overnight with 10 mM **L27**. Crystallization droplets were formed by mixing the protein-ligand solution 1:1 with a reservoir solution containing 0.1 M HEPES, pH 7.4, 0.1 M MgCl_2_, 13–15% PEG 3350, 10% isopropanol, and 25% ethylene glycol. Well-formed crystals grew within approximately one week. X-ray diffraction data were collected at 100 K using an in-house Rigaku XtaLAB Synergy diffractometer (Tokyo, Japan) and processed with CrysAlisPro (v 1.171.41.64) The structure was determined by molecular replacement in MOLREP (v 9.2), employing the PDE4D coordinates (PDB: 5WQA) as the initial template. The model was subsequently improved through iterative rounds of manual rebuilding in Coot (v 0.9.6) [27] and refinement in Phenix (v 1.19.2) [28]. Coordinates and structure factors have been deposited in the Protein Data Bank under accession code 9WP5 and are publicly available. All structural representations were prepared with PyMOL (v 2.5.2).

### 2.5. Molecular Docking, Molecular Dynamics Simulations, and Binding Free Energy Calculation

Molecular docking was conducted using the CDOCKER protocol implemented in Accelrys Discovery Studio (Accelrys Discovery Studio 4.0), with the co-crystal structure of PDE4D in complex with roflumilast (PDB ID: 1XOQ) serving as the receptor model. During structure preparation, all water molecules were removed except for six molecules coordinated to Mg^2+^ and Zn^2+^ ions. Hydrogen atoms and partial charges were added according to the CHARMM (CHARMM22/CMAP) force field and the Momany–Rone charge assignment method. Ionizable residues were protonated, consistent with neutral pH, and a spherical binding site with a 10 Å radius centered on the active site was defined. To validate the docking protocol, the crystallographic roflumilast ligand was extracted from the complex and re-docked into the PDE4D binding site under the same parameter set. The root-mean-square deviations (RMSDs) between the top 20 docking poses and the original crystal pose ranged from 0.65 Å to 1.7 Å, confirming that the CDOCKER method was suitable for the PDE4D system. Subsequently, compound **L27** was docked using the default parameters. The final binding poses were selected based on the docking scores and manual visual inspections.

The dynamic behavior of the PDE4D–**L27** system was investigated through all-atom molecular dynamics simulations performed using GROMACS software (version 2022.5). The simulation system was constructed by immersing the complex in a cubic water box with a minimum 10 Å clearance distance from any protein atom to the box boundary, employing the TIP3P water model for solvation. Sodium ions (Na^+^) were added to neutralize the system charge. Initial structural optimization was conducted through 5000 iterations of the steepest descent minimization algorithm to remove unfavorable atomic contacts. System equilibration proceeded through sequential phases: an initial 100 ps simulation in the canonical ensemble (NVT) maintained at 300 K using velocity rescaling temperature coupling, followed by a 100 ps isothermal–isobaric ensemble (NPT) simulation at 1 bar pressure regulated through the Parrinello–Rahman pressure coupling scheme.

Production dynamics simulations extended for 50 nanoseconds under isothermal–isobaric conditions (300 K, 1 bar), with system coordinates recorded at 2 picosecond intervals. Trajectory analysis incorporated evaluation of root-mean-square deviation, residue fluctuation profiles, and macromolecular compactness metrics to characterize system stability and conformational evolution. Binding free energies (∆G_bind_) were estimated using the molecular mechanics Poisson–Boltzmann surface area (MM-PBSA) method as implemented in the gmx_MMPBSA package. A total of 40 snapshots were evenly extracted from the last 20 ns of the production molecular dynamics trajectory. The binding free energy was calculated according to the following equations:

(1) $$∆G_{bind}=E_{MM}+E_{PB}+E_{SA}-T∆S$$

(2) $$∆E_{MM}=E_{vdw}+E_{elec}$$

where $E_{MM}$ represents the gas-phase molecular mechanics energy, comprising van der Waals ($E_{vdw}$) and electrostatic ($E_{elec}$) interactions. It is worth noting that the energy calculations presented here are primarily intended to compare the binding of the same small molecule to the target protein PDE4 in different binding modes. Given that this small molecule possesses a relatively rigid backbone structure and exhibits similar binding modes, it is theoretically plausible that the associated entropy changes upon binding are also comparable. Moreover, entropy calculations for such large composite systems are computationally expensive. Therefore, the binding free energy calculations reported herein do not include entropy contributions. Consequently, the resulting binding free energies may be subject to overestimation.

### 2.6. In Vitro Anti-Inflammatory

The cytotoxicity of compound **L27** towards RAW 264.7 cells was evaluated using the CCK-8 assay. Murine RAW 264.7 cells were cultured in Dulbecco’s modified Eagle medium (DMEM) supplemented with 10% fetal bovine serum (FBS), 100 U/mL penicillin, and 100 µg/mL streptomycin at 37 °C in a 5% CO_2_-humidified incubator. For the assay, cells were seeded into 96-well plates at a density of 8 × 10^3^ cells/well. After 24 h, the cells were treated with various concentrations of **L27** (0, 5, 10, 20, 40, 50, 60, 80, and 100 μM; three replicates per concentration) for another 24 h. Subsequently, the medium was replaced with fresh medium containing the CCK-8 working solution. Following a 1 h incubation, the absorbance at 450 nm was measured using an enzyme-linked immunosorbent assay (ELISA) plate reader. Cell viability was expressed as a percentage relative to the vehicle-treated control group, which was set at 100%.

The nitrite concentration in cell culture supernatants was measured using a commercial Nitric Oxide Assay Kit (Beyotime, S0021S, Shanghai, China). RAW 264.7 cells were seeded overnight, then co-treated with 1 µg/mL LPS and indicated concentrations of compound **L27** for 24 h. After incubation, 50 µL supernatant from each sample was mixed with Griess Reagents I and II according to the kit protocol. Absorbance at 540 nm was measured following a 10 min reaction at room temperature. Nitrite concentrations were determined using a sodium nitrite standard curve.

The effect of **L27** on the mRNA expression of key inflammatory factors in RAW 264.7 cells was assessed by quantitative real-time PCR (RT-qPCR). Briefly, RAW 264.7 cells were treated simultaneously with 1 µg/mL LPS and the indicated concentrations of compound **L27** for 4 h. Total RNA was isolated from treated cells using TRIzol reagent. cDNA was synthesized from 1 µg of total RNA using a reverse transcription kit. Quantitative PCR was performed on a real-time PCR system with SYBR Green master mix. The PCR protocol consisted of an initial denaturation step, followed by 40 cycles of amplification. The primer sequences used were as follows: TNF-α forward sequence, CAGGCGGTGCCTATGTCTC; TNF-α reverse sequence, CGATCACCCCGAAGTTCAGTAG; IL-6 forward sequence, CTGCAAGAGACTTCCATCCAG; IL-6 reverse sequence, AGTGGTATAGACAGGTCTGTTGG; IL-1β forward sequence, AAATGCCACCTTTTGACAGTG; IL-1β reverse sequence, TGGATGCTCTCATCAGGACAG. Relative quantification of gene expression was analyzed using the 2^−ΔΔCt^ method.

The anti-inflammatory effect of **L27** on the protein expression levels of TNF-α and IL-1β was further evaluated by Western blot. Cells were co-treated with LPS (1 µg/mL) and compound **L27** for 4 h, following the same regimen as for qPCR. Cells were lysed in RIPA buffer containing protease and phosphatase inhibitors. The protein concentration of lysates was determined via a bicinchoninic acid (BCA) assay. Equal amounts of protein were separated by SDS-PAGE and transferred to polyvinylidene difluoride (PVDF) membranes. After blocking with 5% non-fat milk, membranes were probed overnight at 4 °C with primary antibodies against IL-1β, TNF-α, and β-actin. Following incubation with appropriate horseradish peroxidase (HRP)-conjugated secondary antibodies, protein bands were visualized using an enhanced chemiluminescence (ECL) substrate. Band intensity was quantified using ImageJ software (v 1.53k), with β-actin serving as the loading control.

All data are presented as the mean ± standard error of the mean (SEM). Statistical comparisons were performed using one-way analysis of variance (ANOVA) followed by Bonferroni’s multiple comparison test via GraphPad Prism software (version 9.0). A *p*-value of less than 0.05 was considered statistically significant.

## 3. Results and Discussions

### 3.1. Rational Design Based on the Co-Crystal Structure of PDE4D–Braylin

**Figure 2:**
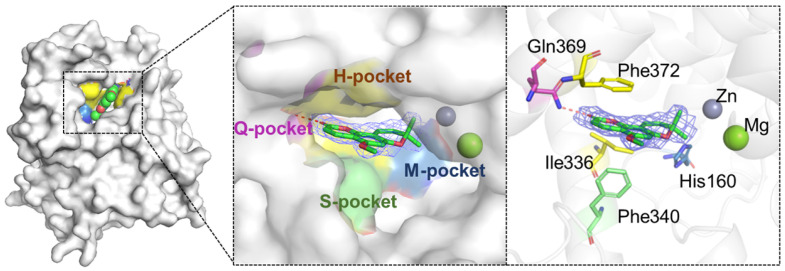
Co-crystal structure of the PDE4D–braylin complex (PDB ID: 9WP5). The protein is shown as a surface representation, with the H-, Q-, S-, and M-pockets colored in orange, purple, green, and blue, respectively. Braylin is shown as stick representation, and key interacting residues are shown as sticks. Zn^2+^ and Mg^2+^ ions are depicted as spheres. The 2Fo–Fc electron density map is contoured at 1.0σ and displayed in light blue.

To elucidate the binding mode between PDE4 and its natural inhibitor braylin, we successfully resolved the co-crystal structure of PDE4D–braylin complex. As shown in Figure 2, the 2Fo-Fc electron density map clearly demonstrated that braylin occupied the active site of PDE4D. Structural analysis revealed that the carbonyl oxygen of the lactone ring in braylin formed a hydrogen bond with the conserved residue Gln369, while the conjugated benzopyranone scaffold engaged in π–π stacking interactions with the hydrophobic clamp formed by Phe372 and Phe340/Ile336. These hydrogen-bonding and stacking interactions, which were characteristic of PDE inhibitors, likely contributed to the basal binding affinity of braylin for PDE4 (IC_50_ of 0.96 μM).

Previous structural studies had established that PDE4 inhibitors commonly interacted with the catalytic site through three principal mechanisms: (i) direct or water-mediated coordination to the metal ions; (ii) hydrogen bonding with the invariant glutamine or other residues; and (iii) π–π stacking with the hydrophobic clamp [29,30]. Notably, recent optimization efforts on several natural PDE4 inhibitors had highlighted that engaging the metal-binding sub-pocket (M-pocket) could substantially enhance the inhibitory potencies [13,20]. As revealed by the co-crystal structure of PDE4D–braylin, the isoprenyl moiety of braylin was directed toward the M-pocket but did not form specific interactions with this region, suggesting a potential site for structural modification to improve activity. Additionally, the methoxy group of braylin extended into a solvent-accessible outer pocket, leaving space for further functionalization. Based on these structural insights, we designed and synthesized a series of substituted braylin derivatives to target both the M-pocket and the adjacent outer region, with the aim of enhancing PDE4 inhibitory activity.

### 3.2. Chemistry

**Scheme 1:**
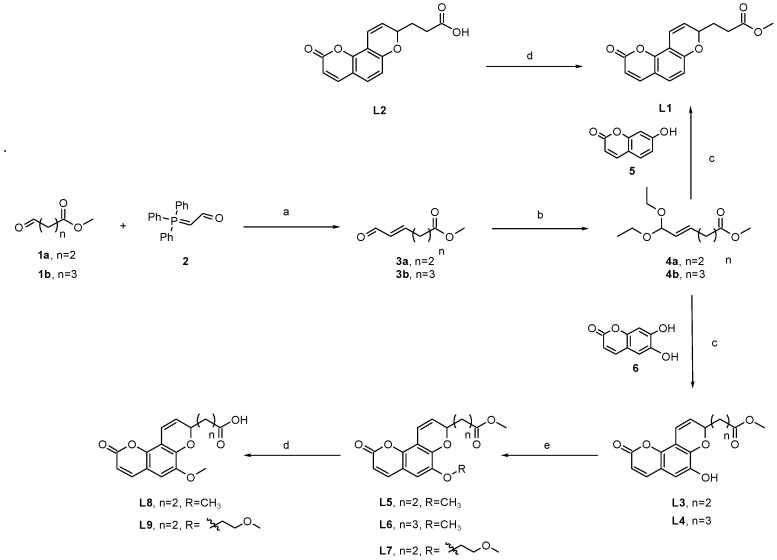
Synthesis of compounds **L1–L9**. Reagents and conditions: (a) dry toluene, 100 °C, 2 h; (b) (i) triethyl orthoformate, KHSO_4_, EtOH, 0 °C to rt, 1 h; (ii) K_2_CO_3_, 2 h; (c) dry xylene, 3-methylpyridine, 160 °C, 6 h; (d) LiOH, THF:H_2_O = 3:1, rt, 1.5 h; (e) acetone, alkyl halide, 56 °C, 8 h.

**Scheme 2:**
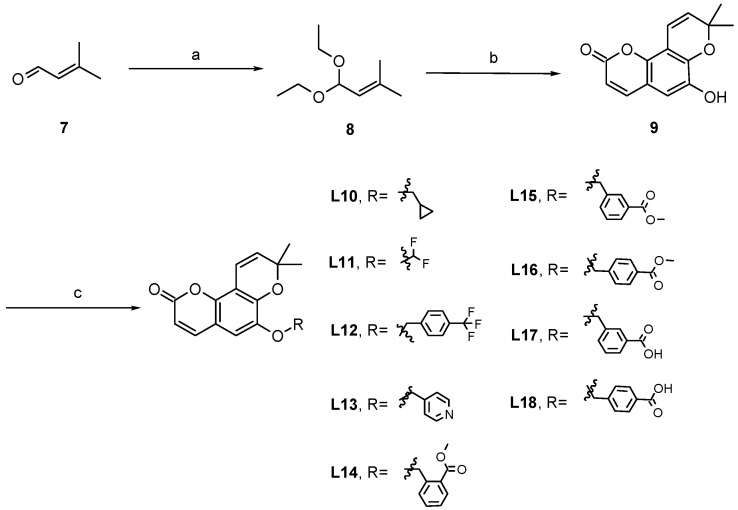
Synthesis of compounds **L10–L18**. Reagents and conditions: (a) (i) triethyl orthoformate, KHSO_4_, EtOH, 0 °C to rt, 1 h; (ii) K_2_CO_3_, 2 h; (b) **6**, dry xylene, 3-methylpyridine, 160 °C, 6 h; (c) acetone, alkyl halide, 56 °C, 8 h.

**Scheme 3:**
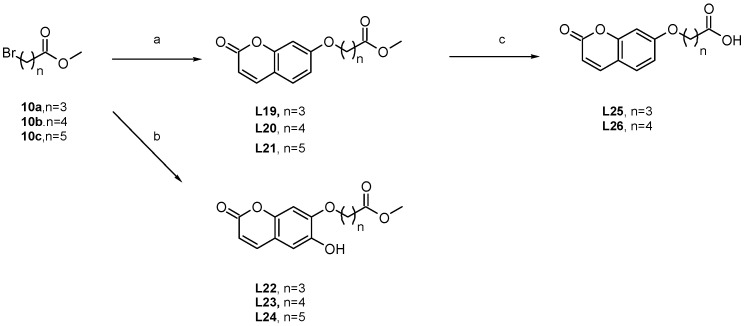
Synthesis of compounds **L19–L26**. Reagents and conditions: (a) **5**, DMF, K_2_CO_3_, 80 °C, 3 h; (b) **6**, DMF, K_2_CO_3_, 80 °C, 3 h; (c) LiOH, THF:H_2_O = 3:1, rt, 1.5 h.

**Scheme 4:**
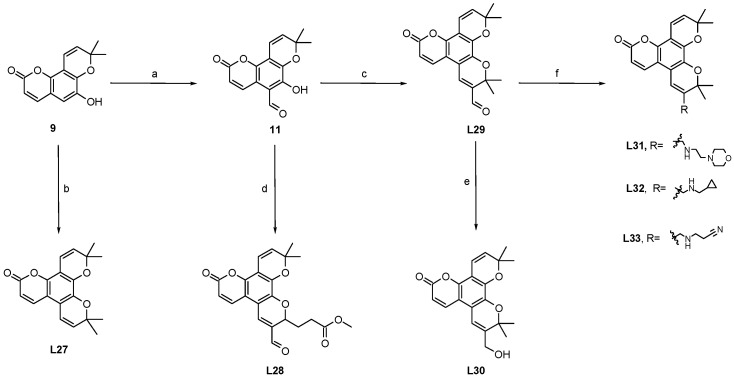
Synthesis of compounds **L27–L33**. Reagents and conditions: (a) hexamethylenetetramine, TFA, 90 °C, 12 h; (b) **8**, dry xylene, 3-methylpyridine, 160 °C, 6 h; (c) 7, K_2_CO_3_, 1,4-dioxane:H_2_O = 1:1, 60 °C, 12 h; (d) **3a**, K_2_CO_3_, 1,4-dioxane:H_2_O = 1:1, 60 °C, 12 h; (e) sodium triaceoxyborohydride, THF, rt, 1.5 h; (f) sodium triaceoxyborohydride, amines, THF, rt, 1.5 h.

As shown in Scheme 1, starting from **1a/1b**, intermediates **3a/3b** were obtained through a Wittig reaction, and the intermediates **4a/4b** were obtained by treating them with potassium bisulfate/trimethyl borate, respectively. Intermediates **4a/4b** were then reacted with the **5** or **6** to obtain the compounds **L1**, **L3,** and **L4**. After being treated with potassium carbonate, **L3** and **L4** reacted with alkyl halides to obtain compounds **L5–L7**, respectively. And the compounds **L1**, **L5,** and **L7** were hydrolyzed with lithium hydroxide to yield the corresponding acids **L2**, **L8,** and **L9**. The same procedures as Scheme 1 were used to get the intermediate **9** (Scheme 2), which was then reacted with alkyl halides to afford the compounds **L10–L18**. In Scheme 3, the raw materials **5** or **6** were treated with 10a-10c, and K_2_CO_3_ in DMF to afford the compounds **L19–L24**, and **L19/L20** were treated with lithium hydroxide to obtain the corresponding acids **L25** and **L26**. In Scheme 4, the intermediate **9** was reacted with **8** to obtain the compound **L27**, and reacted with hexamethylene tetramine to obtain the intermediate **11**, which can be reacted with **3a**/7 by the treatment of K_2_CO_3_ in a bi-component solvent of 1,4-dioxane/H_2_O to afford the compounds **L28** and **L29**. Finally, the compounds **L30–L33** were obtained by a reduction reaction or a reductive amination reaction.

### 3.3. Structure–Activity Relationships (SARs)

Guided by the PDE4D–braylin co-crystal structure, initial modifications focused on two regions: the isoprenyl moiety (oriented toward the metal-binding M-pocket) and the adjacent methoxy group (facing a solvent-accessible area). To potentially engage the catalytic metal ions (Zn^2+^/Mg^2+^), carboxylic acid and various ester groups were introduced at the isoprenyl side chain. Concurrently, polar nitrogen- and oxygen-containing groups were appended to the methoxy group to improve physicochemical properties. Accordingly, compounds **L1–L18** were designed and synthesized.

Contrary to expectations, this rational design strategy proved ineffective. As summarized in Table 1, nearly all analogs (**L1–L18**) exhibited substantially reduced PDE4D inhibitory activity compared to the hit compound, indicating that direct targeting of the metal-binding site or the solvent-exposed region with polar groups was detrimental to binding.

Further investigation into the isoprenyl moiety revealed a strict dependence of activity on linker length. Extending the side chain by two carbons abolished activity, whereas a three-carbon extension restored or even enhanced it (e.g., **L4**, **L6**). This suggests that a three-carbon linker optimally projects the terminal group into a complementary sub-pocket, while a shorter linker may misdirect the group, leading to unproductive interactions. The critical importance of the isoprenyl group itself was confirmed by its removal. Analogs featuring only the coumarin core appended with flexible carboxylic acid chains of varying lengths (**L19–L26**, Table 2) showed near-complete loss of activity, underscoring that the dimethyl-substituted isoprenyl unit was an indispensable pharmacophore for PDE4D inhibition.

**Figure 3:**
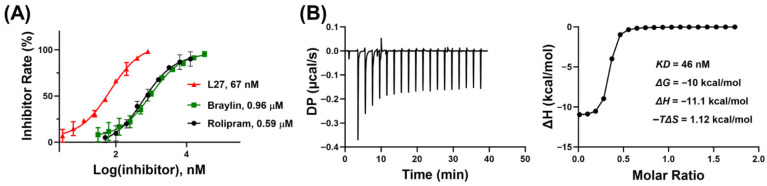
(**A**) The inhibitory curves of **L27** and braylin against PDE4D. (**B**) ITC analysis of the interactions between PDE4 and PDME.

Based on these results, a refined design strategy was formulated: (1) retain the essential dimethylisoprenyl group; (2) preserve the optimal three-carbon linker; and (3) introduce extended conjugated systems directed toward the solvent-exposed region. The implementation of this strategy yielded promising outcomes. As shown in Table 3, derivatives such as **L27** and **L31** maintained high activity. Notably, introducing a second isoprenyl ring at the 6-position phenolic hydroxyl yielded the most potent compound, **L27**, which achieved 96.46% inhibition at 1 µM and a significant 65.74% inhibition at 100 nM. Further quantification revealed that **L27** has an IC_50_ value of 67 nM, representing a 14-fold improvement in potency relative to the hit compound braylin. The dose–response curves of both compounds, along with the positive control rolipram, were presented in Figure 3A. Together, these findings indicated that extending the hydrophobic conjugated scaffold represented a productive strategy for enhancing inhibitor potency within this chemotype.

Characterization of Binding Interactions between **L27** and PDE4D by Isothermal Titration Calorimetry.

The thermodynamic profile of the PDE4D–**L27** interaction, obtained by isothermal titration calorimetry (ITC), revealed an enthalpy-driven binding mechanism. As shown in Figure 3B, analysis of the titration data yielded a highly favorable enthalpy change (Δ*H* = −11.1 kcal/mol), indicating that the association was stabilized by specific non-covalent interactions—such as hydrogen bonds and van der Waals contacts—within the binding pocket. In contrast, the binding was entropically disfavored to a lesser extent (*T*Δ*S* = −1.12 kcal/mol), consistent with a net reduction in solvation and conformational freedom upon complex formation. As a result, the calculated binding free energy was strongly favorable (Δ*G* = −10 kcal/mol), corresponding to a dissociation constant (*K_d_*) of 45 nM based on the relationship Δ*G* = RT ln(*K_d_*). This thermodynamic affinity was in close agreement with the functional inhibition constant (IC_50_ = 67 nM) determined from enzymatic assays. The consistency between the biophysical *K_d_* and the biochemical IC_50_ confirmed the high affinity and specificity of **L27** for PDE4D, underscoring the biological relevance of the measured interaction.

### 3.4. Selective Profile of **L27** Towards Other PDEs

The selectivity profile of **L27** across different PDE isoforms was evaluated using previously established protocols, and results were summarized in Table 4. **L27** exhibited remarkable selectivity (≥86-fold) against isoforms including PDE1C, PDE2A, PDE3A, PDE8A and PDE9A, while demonstrating moderate inhibitory activity against PDE5A, PDE7A and PDE10A (8~16-fold selectivity). Taken together, these findings support the potential of **L27** as a potent PDE4 inhibitor, and further optimization efforts targeting the selective Q1/Q2 pocket of PDE4 are underway.

It has been reported that adverse effects associated with PDE4 inhibitors, particularly nausea and emesis, are closely related to PDE4D inhibition in the central nervous system. Therefore, subtype selectivity plays a critical role in determining the therapeutic window of PDE4 inhibitors. In this study, L27 exhibited good selectivity for PDE4D over most other PDE isoforms, while retaining moderate activity against PDE5A, PDE7A, and PDE10A. This selectivity profile suggests that L27 maintains strong target engagement with PDE4 while limiting broad off-target interactions, which may contribute to a favorable balance between efficacy and safety. Further optimization of subtype selectivity may help to reduce potential adverse effects while maintaining therapeutic potency.

### 3.5. Putative Binding Pattern of **L27** with PDE4

To elucidate the binding mode of **L27** to PDE4D, we attempted but failed to obtain the co-crystal structure of the PDE4D–**L27** complex. As an alternative approach, molecular docking, molecular dynamics (MD) simulations, and binding free energy calculations were employed to investigate the binding pattern of **L27** to PDE4D.

Molecular docking suggested two favorable binding conformations of **L27** within the PDE4D active site: one with the dimethylisoprenyl group oriented toward the solvent-exposed S-pocket, and the other with this group directed toward the inner H-pocket. To assess the stability of these two binding poses, 50 ns MD simulations were performed for each system, and the last 20 ns trajectories were used for subsequent binding free energy analysis. The energy profiles remained stable during the simulations (Figures S1 and S2). Both systems reached equilibrium, with the root-mean-square deviation (RMSD) of the ligand stabilizing below 2.0 Å during the final 20 ns production phase (Figure S3). Moreover, the root-mean-square fluctuation (RMSF) of residues lining the binding pocket remained below 2 Å, indicating limited conformational flexibility (Figure 3A).

**Figure 4:**
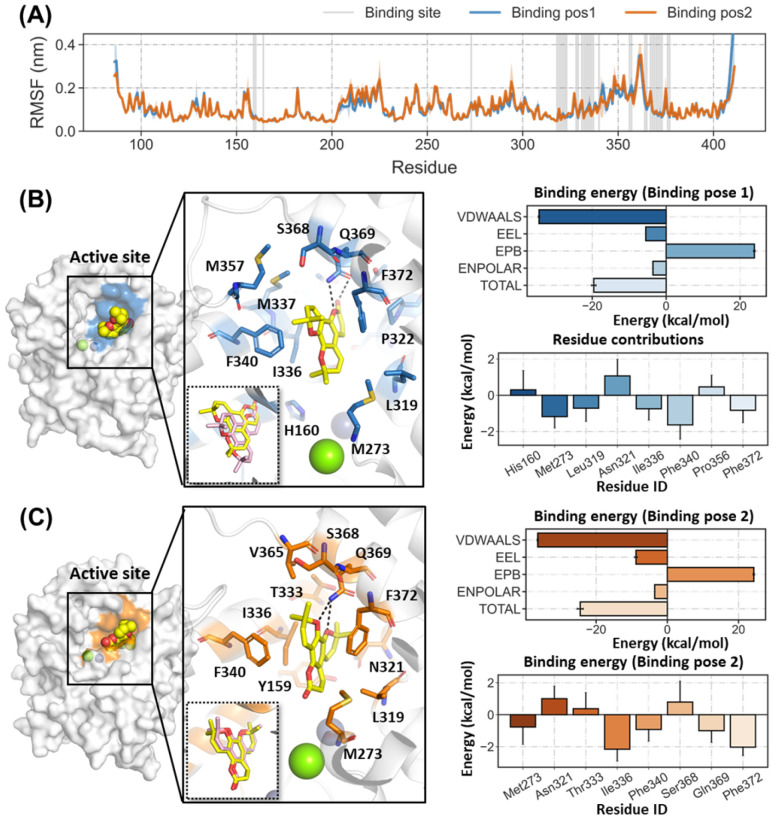
Two predicted binding modes of compound **L27** with PDE4. (**A**) Root-mean-square fluctuation (RMSF) of Cα atoms of PDE4D. Binding pose 1 and pose 2 are shown in blue and orange, respectively. Each simulation was repeated three times, and the error bars are represented by light shading. (**B**) The lowest-energy binding mode, total binding free energy, and representative residue contributions of the **L27**–PDE4D complex in pose 1. (**C**) The lowest-energy binding mode, total binding free energy, and representative residue contributions of the L27–PDE4D complex in pose 2. In the structural representations, the protein is shown as a cartoon/surface in white, and the ligand is shown as sticks in yellow. Key interacting residues are shown as sticks (colored according to binding pose: blue for pose 1 and orange for pose 2). Zn^2+^ and Mg^2+^ ions are shown as green spheres. Hydrogen bonds are represented as dark dashed lines. water molecules, and hydrogen atoms were omitted.

As illustrated in Figure 3, **L27** occupies the catalytic pocket of PDE4D and forms key interactions, including hydrogen bonds between the oxygen atoms of its isoprenyl groups and the catalytic triad residue Gln369, as well as extensive van der Waals contacts with residues such as Met273, Ile336, Phe340, and Phe372. The binding free energies (Δ*G*) for pose 1 and pose 2 were calculated to be −19.58 ± 2.76 kcal·mol^−1^ and −24.46 ± 4.58 kcal·mol^−1^, respectively (Figure 4B,C). Although the neglect of entropy contributions and the presence of divalent metal ions in the PDE4 catalytic pocket may compromise the accuracy of Δ*G* calculations, potentially leading to overestimation, the lower binding free energy and smaller ligand RMSD fluctuations in pose 2—where the dimethylisoprenyl group was oriented toward the H-pocket—suggested that this conformation likely represented the lowest-energy binding mode for the **L27**–PDE4D complex. Detailed analysis indicated the binding was predominantly driven by the van der Waals interactions (−36.4 ± 2.88 kcal·mol^−1^), with additional stabilization provided by electrostatic interactions (−8.8 ± 1.89 kcal·mol^−1^), which provided structural rationales for the proposed enthalpy-driven binding mechanism (Δ*H* = −11.1 kcal/mol) revealed by ITC. In addition, per-residue energy decomposition identified Ile336 (−2.16 ± 0.75 kcal·mol^−1^), Phe372 (−2.02 ± 0.54 kcal·mol^−1^), Gln369 (−1.00 ± 0.73 kcal·mol^−1^), and Phe340 (−0.92 ± 0.73 kcal·mol^−1^) as major favorable contributors to binding (Figure 4C), whereas residues Ser368, Asn321, and Thr333 might exhibit unfavorable contributions.

It should be noted that the absolute binding free energies calculated by the MM-PBSA method are often overestimated due to methodological limitations, including the neglect of entropic contributions and the use of implicit solvent models.

To further evaluate the reliability of the MM-PBSA method, a comparison between calculated binding free energies and experimental IC_50_ values was performed. Although the absolute values are not directly comparable, a generally consistent trend was observed, where compounds with lower IC_50_ values tended to exhibit more favorable binding free energies.

### 3.6. Remarkable Anti-Inflammatory Effects of **L27**

**Figure 5:**
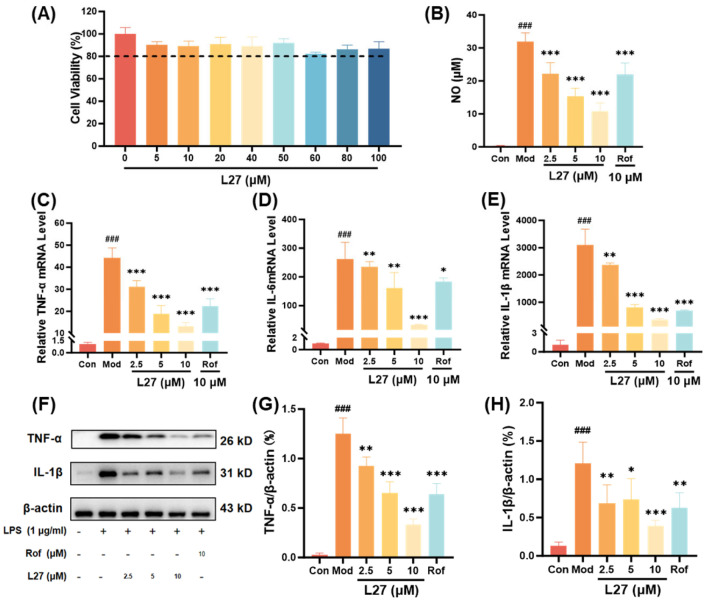
**L27** inhibited the expression levels of inflammatory factors in RAW 264.7 cells stimulated by 10 ng/mL LPS. (**A**) Cytotoxicity of **L27** in RAW 264.7 cells. The dashed line indicates the cell viability threshold of 80%. (**B**) **L27** (2.5, 5, and 10 μM) and roflumilast (10 μM, positive control) effectively inhibited the release of NO. (**C**–**E**) **L27** reduced the relative expressions of mRNA of TNF-α, IL-6, and IL-1β quantified by RT-PCR. (**F**–**H**) **L27** reduced the relative expressions of proteins of TNF-α and IL-1β, as analyzed by Western blot with β-actin serving as the loading control. Data were presented as mean ± standard error of the mean (SEM) (n = 3). The differences between groups were determined using one-way ANOVA analysis. ### *p* < 0.001 compared with control group. * *p* < 0.05, ** *p* < 0.01, *** *p* < 0.001 compared with model group.

Inflammatory cytokines were reported to play an important role in the occurrence, development, and maintenance of various diseases. Therefore, the anti-inflammatory activity of the PDE4 inhibitor **L27** was systematically evaluated using a multi-layered experimental approach in the relevant RAW264.7 macrophage cell line. Initial CCK-8 assays established that **L27** exhibited no significant cytotoxicity at concentrations up to 100 μM (Figure 5A), confirming that the subsequent pharmacological observations at lower concentrations (2.5–10 μM) reflected specific anti-inflammatory effects rather than general toxicity. As shown in Figure 5B, LPS stimulation markedly increased nitric oxide (NO) production compared with the control group. However, pretreatment with **L27** (2.5, 5, and 10 μM) significantly attenuated this increase in a concentration-dependent manner, achieving approximately 30% inhibition at 10 μM, which demonstrated its notable anti-inflammatory activity. Furthermore, RT-qPCR analysis revealed that **L27** pretreatment effectively suppressed LPS-induced mRNA expression of key inflammatory mediators, including TNF-α, IL-6, and IL-1β, also in a concentration-dependent manner (Figure 5C). Extending these findings to the protein level, Western blot analysis (Figure 5D) showed a parallel inhibitory effect, with **L27** significantly reducing LPS-induced protein expression of both TNF-α and IL-1β. Notably, the inhibitory potency of **L27** at 10 μM was comparable to that of the positive control, roflumilast, a marketed PDE4 inhibitor with established anti-inflammatory activity. This comparison with a clinically used PDE4 inhibitor further highlights the pharmacological relevance of **L27** as a promising alternative scaffold for anti-inflammatory drug development.

Collectively, these results demonstrate that **L27** exerted its anti-inflammatory effects through multi-level suppression of pro-inflammatory mediators—from transcriptional regulation to protein synthesis—without affecting cell viability. The logical experimental progression, from safety assessment (CCK-8) to transcriptional analysis (RT-qPCR) and finally to functional protein validation (Western blot), formed a robust and internally consistent evidence chain. The reproducible dose-dependent response across all assays further strengthened the validity of the findings and supports a specific, potent mechanism of action. This integrated analysis provided a comprehensive characterization of the anti-inflammatory profile of **L27** and established a solid foundation for future mechanistic studies.

## 4. Conclusions

In summary, the structure-based optimization of a natural PDE4 inhibitor, braylin (IC_50_ of 0.96 µM), successfully led to the discovery of lead compound **L27** with improved PDE4 inhibitory potency (IC_50_ of 67 nM). Guided by the co-crystal structure of the PDE4D–braylin complex, a systematic SAR exploration revealed that the dimethyl-substituted isoprenyl moiety is an indispensable pharmacophore. The most productive strategy for enhancing activity involved retaining this core and extending the hydrophobic conjugated scaffold, as exemplified by the introduction of a second isoprenyl ring in **L27**. Biophysical characterization by ITC confirmed the high affinity and enthalpy-driven binding of **L27** to PDE4 (*K_d_* = 45 nM). Computational simulations further supported a stable, low-energy binding mode with key interactions involving the conserved residue Gln369 and hydrophobic clamp. Functionally, **L27** exhibited a pronounced multi-level anti-inflammatory profile in LPS-stimulated RAW 264.7 macrophages. At non-cytotoxic concentrations (up to 100 µM), **L27** dose-dependently inhibited NO production, suppressed the mRNA expression of TNF-α, IL-6, and IL-1β, and attenuated the corresponding protein expression with potency comparable to the clinical PDE4 inhibitor roflumilast.

Collectively, this study provides a compelling example of rational optimization from a natural product hit to a potent and functionally active PDE4 inhibitor. Compound **L27** not only serves as a valuable chemical probe for dissecting PDE4-related biology but also represents a promising lead candidate for the development of novel anti-inflammatory therapeutics. Future work will focus on improving the selectivity and pharmacokinetic properties of **L27** and further evaluating its in vivo efficacy in relevant inflammatory disease models.

## Figures and Tables

**Table 1:** The results of the PDE4D inhibitory activity tests for compounds **L1–L18**.

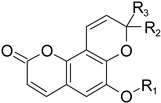					
Compounds	R_1_	R_2_	R_3_	PDE4D Inhibition Rate	
				1 μM	100 nM
**L1**	**-**	H	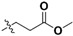	32.05%	-
**L2**	**-**	H	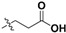	33.07%	-
**L3**	H	H	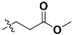	9.34%	-
**L4**	H	H	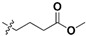	80.61%	37.31%
**L5**	CH_3_	H	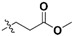	17.27%	10.39%
**L6**	CH_3_	H	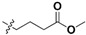	78.45%	58.80%
**L7**	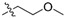	H	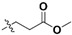	15.14%	23.17%
**L8**	CH_3_	H	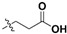	0.14%	7.89%
**L9**	** 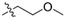 **	H	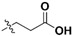	4.60%	11.00%
**L10**	** 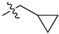 **	CH_3_	CH_3_	32.40%	13.69%
**L12**	** 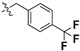 **	CH_3_	CH_3_	12.36%	4.14%
**L13**	** 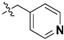 **	CH_3_	CH_3_	35.20%	-
**L14**	** 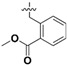 **	CH_3_	CH_3_	64.88%	22.50%
**L15**	** 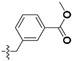 **	CH_3_	CH_3_	78.80%	27.33%
**L16**	** 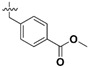 **	CH_3_	CH_3_	22.11%	13.77%
**L17**	** 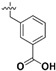 **	CH_3_	CH_3_	4.13%	-
**L18**	** 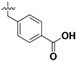 **	CH_3_	CH_3_	14.94%	-

**Table 2:** The results of the PDE4D inhibitory activity tests for compounds **L19–L26**.

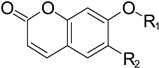			
Compounds	R_1_	R_2_	PDE4D Inhibition Rate
			1 μM
**L19**	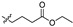	H	3.85%
**L20**	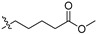	H	15.31%
**L21**	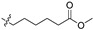	H	29.82%
**L22**	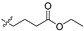	OH	5.03%
**L23**	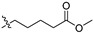	OH	9.15%
**L24**	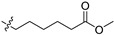	OH	8.72%
**L25**	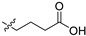	H	2.80%
**L26**	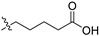	H	26.96%

**Table 3:** The results of the PDE4D inhibitory activity tests for compounds **L27–L33**.

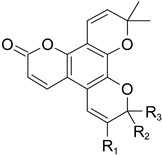					
Compounds	R_1_	R_2_	R_3_	PDE4D Inhibition Rate	
				1 μM	100 nM
**L27**	H	CH_3_	CH_3_	96.46%	65.74%
**L28**	CHO	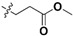	H	61.02%	10.40%
**L29**	CHO	CH_3_	CH_3_	69.40%	15.82%
**L30**	CH_2_OH	CH_3_	CH_3_	71.83%	22.18%
**L31**	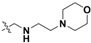	CH_3_	CH_3_	92.25%	24.10%
**L32**	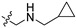	CH_3_	CH_3_	66.05%	10.82%
**L33**	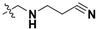	CH_3_	CH_3_	82.56%	25.75%

**Table 4:** Selectivity Profile of **L27** across PDEs.

PDEs	IC_50_ (nM)	Selective Index
PDE4D2 (86–413)	67 ± 4	-
PDE1C2 (147–531)	9320 ± 250	139
PDE2A (580–919)	5760 ± 630	86
PDE3A (679–1087)	>10,000	>149
PDE5A1 (535–860)	1070 ± 180	16
PDE7A1 (130–482)	820 ± 30	12
PDE8A1 (480–828)	>10,000	>149
PDE9A2 (181–506)	>10,000	>149
PDE10A2 (449–770)	540 ± 80	8

## Data Availability

The original contributions presented in this study are included in the article/Supplementary Materials. Further inquiries can be directed to the corresponding author. Informed consent was obtained from all subjects involved in the study.

## Supplementary Materials

The following supporting information can be downloaded at: https://www.mdpi.com/article/10.3390/pharmaceutics18050516/s1, Table S1. Statistics data of PDE4D-**L27** co-crystal structure. Figure S1. Energy fluctuations of PDE4D-**L27** complex in Pose 1 during MD simulations. (A) Potential, (B) Kinetic energy, (C) Total energy, (D) Pressure, (E) Temperature and (F) Volume. Each simulation was repeated three times, and the error bars for the three simulations were represented with light shading. Figure S2. Energy fluctuations of PDE4D-**L27** complex in Pose 2 during MD simulations. (A) Potential, (B) Kinetic energy, (C) Total energy, (D) Pressure, (E) Temperature and (F) Volume. Each simulation was repeated three times, and the error bars for the three simulations were represented with light shading. Figure S3. The Stability of PDE4D-**L27** Complexes in Pose1 and Pose2. The root mean square deviation (RMSD) of PDE4D (A) and compound L27 (B) in Pose1 during 50-ns MD simulations. The RMSD of the PDE4D binding pocket residues (C) and compound L27 (D) in Pose2 during 50-ns MD simulations. Each simulation was repeated three times, and the error bars for the three simulations are represented with light shading. Table S2. Binding free energy of PDE4D-**L27** complex in Pose 1 and Pose 2.
