# Supplementary material for: Design, Synthesis, and Evaluation of Braylin Derivatives as Novel PDE4 Inhibitors with Anti-Inflammatory Effects
Source: Pharmaceutics. 2026 Apr 23;18(5):516. doi: 10.3390/pharmaceutics18050516 (PMC13210620; doi:10.3390/pharmaceutics18050516)
Supplement: Supplementary file 1 [file pharmaceutics-18-00516-s001.zip › pharmaceutics-4216175-supplementary.pdf]

Supporting Information

Design, Synthesis and Evaluation of Braylin Derivatives as Novel PDE4 Inhibitors with Anti-inflammatory Effects

Yongdan Guo [1,†], Xue Wang [1,†], Feng Zhang [1,†], Tianshen Zheng [1], Zhuo Chen [1], Sen Wang [1], Guofeng Yang [1], Haibo Wang [1], Wenbo Yin [1,2], Shuheng Huang [1], Yi-You Huang [1,2,*], Hai-Bin Luo [1,2,*] and Deyan Wu [1,2,*].

[1] Key Laboratory of Tropical Biological Resources of Ministry of Education, School of Pharmaceutical Sciences, Hainan University, Haikou 570228, Hainan, China

[2] Song Li's Academician Workstation of Hainan University (School of Pharmaceutical Sciences), Yazhou Bay, Sanya, 572000, China

† These authors contribute equally to this work.

* Corresponding author. Email:

Yi-You Huang: hyyou@hainanu.edu.cn

Hai-Bin Luo: hbluo@hainanu.edu.cn

Deyan Wu: wudeyan@hainanu.edu.cn

Table of Contents:

1. Diffraction data and structure refinement statistics of crystallography…. ……. ….S2

2. Energy fluctuations and RMSD analysis of PDE4-**L27** complex………………....S3

3. Chemistry……………….. ……………………………………………………..S5

4. ^{1}H NMR, ^{13}C NMR and MS data for tested compounds………………. ……… ..S17

5. HPLC data for tested compounds…………………………………………………S64

1. Diffraction data and structure refinement statistics of crystallography

Table S1. **S**tatistics data of PDE4D-**L27** co-crystal structure

	PDE4D-**L27** **(PDB ID: 23IX)**
Data collection	
Wavelength (Å)	1.5418
Temperature (K)	100
Resolution (Å)	23.55-2.40
Space group	$P2_12_12_1$
Unit Cell: *a, b, c* (Å)	58.02, 80.69, 163.14
Unit Cell: *α, β, γ* (°)	90, 90, 90
No. reflections	30610(3015 [a])
Completeness (%)	99.6(100[a])
R_{merge}	0.06(0.127 [a])
$<I/\sigma(I)>$	18.5(7.7 [a])
Redundancy	4.7(4.4 [a])
Structure refinement	
R-factor/R-free	0.21/0.26
RMS deviations	
Bond lengths	0.002 Å
Bond angles	0.537
Average B-factor (Å^2) (atoms)	
Protein	26.5(5230)
Inhibitor	37.1(38)
Zn	21.3(2)
Mg	10.6(2)
Waters	20.5(89)
Ramachandran plot	
Preferred	96.88%
Allowed	2.96%

[a] The high resolution annulus.

2. Energy fluctuations and RMSD analysis of PDE4-L27 complex

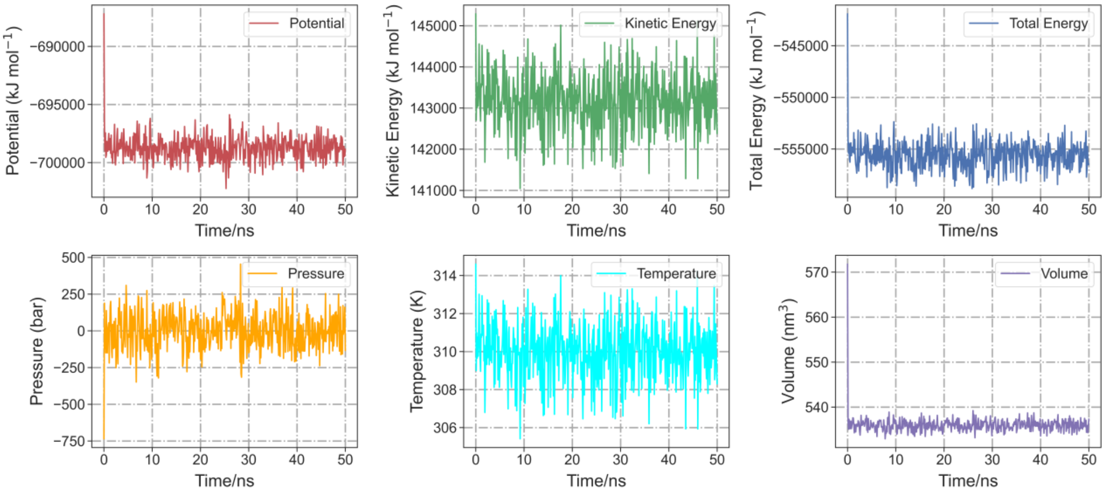

Figure S1. Energy fluctuations of PDE4D-**L27** complex in Pose 1 during MD simulations. (A) Potential, (B) Kinetic energy, (C) Total energy, (D) Pressure, (E) Temperature and (F) Volume. Each simulation was repeated three times, and the error bars for the three simulations were represented with light shading.

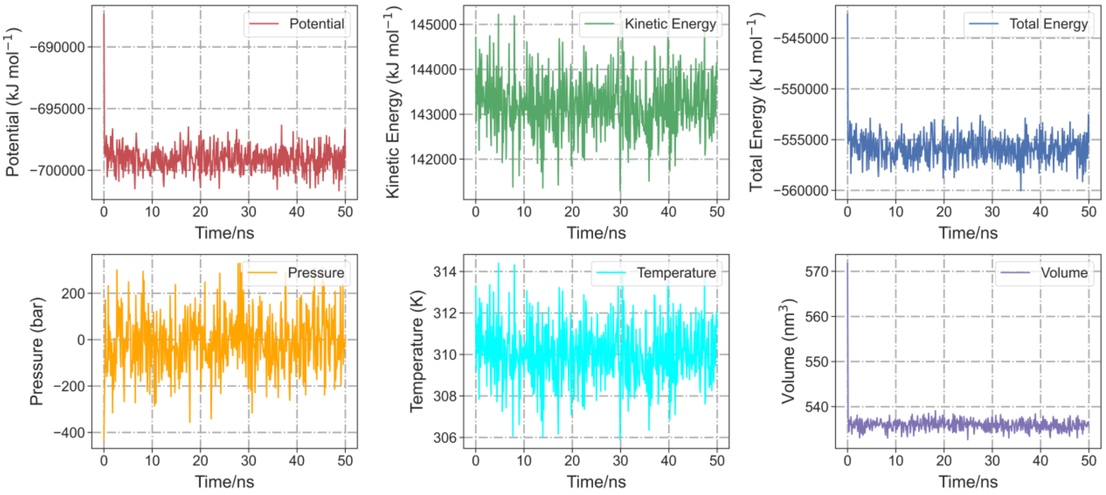

Figure S2. Energy fluctuations of PDE4D-**L27** complex in Pose 2 during MD simulations. (A) Potential, (B) Kinetic energy, (C) Total energy, (D) Pressure, (E) Temperature and (F) Volume. Each simulation was repeated three times, and the error bars for the three simulations were represented with light shading.

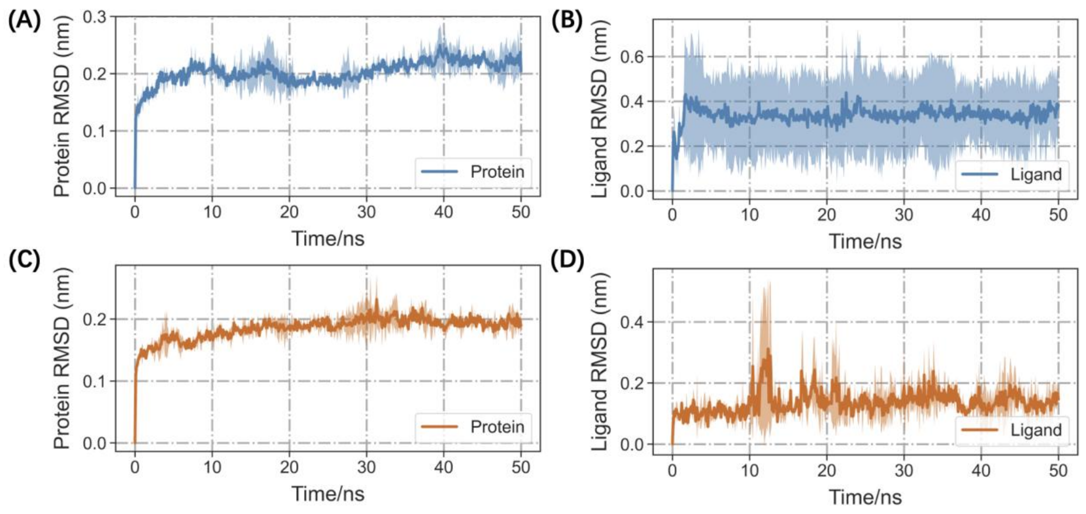

Figure S3. The Stability of PDE4D-**L27** Complexes in Pose1 and Pose2. The root mean square deviation (RMSD) of PDE4D (A) and compound L27 (B) in Pose1 during 50-ns MD simulations. The RMSD of the PDE4D binding pocket residues (C) and compound L27 (D) in Pose2 during 50-ns MD simulations. Each simulation was repeated three times, and the error bars for the three simulations are represented with light shading.

Table S2. Binding free energy of PDE4D-**L27** complex in Pose 1 and Pose 2

Energy (kcal/mol)	Pose 1		Pose 2	
	Average	SD	Average	SD
ΔVDWAALS	-34.38	2.82	-36.4	2.88
ΔEEL	-5.51	2.45	-8.8	1.89
ΔEPB	23.87	3.23	24.29	2.94
ΔENPOLAR	-3.57	0.12	-3.55	0.10
ΔGGAS	-39.89	4.17	-45.21	3.80
ΔGSOLV	20.31	3.22	20.75	2.93
ΔTOTAL	-19.58	2.76	-24.46	4.58

3. Chemistry.

Methyl (E)-6-oxohex-4-enoate (**3a**).

Procedure A. A solution of compound **1a** (1.0 mmol) and (formylmethyl)triphenylphosphorane (1.02 mmol) in anhydrous toluene (2.0 mL) was heated at 100 °C for 1 h. After completion of the reaction as monitored by TLC, the mixture was concentrated under reduced pressure. The residue was purified by silica gel column chromatography (petroleum ether/EtOAc = 5:1) to afford the target compound as a yellowish oil. Yield: 60%. ^{1}H NMR (400 MHz, $CDCl_3$) δ 9.47 (d, J = 7.8 Hz, 1H), 6.83 (dt, J = 15.7, 6.4 Hz, 1H), 6.09 (dd, J = 15.8, 7.8 Hz, 1H),3.66 (s, 3H), 2.63 (dd, J = 13.8, 7.0 Hz, 2H), 2.51 (t, J = 7.2 Hz, 2H).

Methyl (E)-7-oxohept-5-enoate (**3b**).

Procedure B. A solution of compound **1b** (1.0 mmol) and (formylmethyl)triphenylphosphorane (1.02 mmol) in dichloromethane (2.0 mL) was heated at 40 °C for 6 h. After completion of the reaction as monitored by TLC, the mixture was concentrated under reduced pressure. The residue was purified by silica gel column chromatography (petroleum ether/EtOAc = 5:1) to afford the target compound as a yellowish oil. Yield: 60%. ^{1}H NMR (400 MHz, Acetone-d_6) δ 9.51 (d, J = 7.8 Hz, 1H), 7.01 – 6.92 (m, 1H),6.07 (dd, J = 15.6, 7.8 Hz, 1H), 3.61 (s, 1H), 2.43 – 2.34 (m, 4H), 1.86 – 1.76 (m, 2H).

General procedure for the synthesis of compounds 4a-4b and 8.

Procedure C. Under an argon atmosphere, a solution of compound **3a** or **3b** (1.0 mmol) and triethyl orthoformate (2.2 mmol) in anhydrous ethanol (5.0 mL) was cooled to 0 °C using an ice-water bath. Potassium hydrogen sulfate (0.05 mmol) was added to the mixture, and the reaction was stirred at 0 °C for 30 min. The mixture was then allowed to warm to room temperature and stirred for an additional 30 min. The reaction was quenched by the addition of saturated aqueous sodium bicarbonate solution, and the resulting mixture was filtered. The solid residue was washed with ethanol (0.5 mL × 2), and the combined filtrates were collected. Potassium carbonate (0.1 mmol) was added to the filtrate, and the resulting mixture was stirred at room temperature for 2 h. After the reaction as monitored by TLC, the mixture was filtered again. The solid was washed with ethanol (0.5 mL × 2), and the combined filtrates were concentrated under reduced pressure at 35°C to afford a colorless clear liquid. The crude product was used directly in the next step without further purification.

*Methyl 3-(2-oxo-2H,8H-pyrano[2,3-f]chromen-8-yl)propanoate (**L1**).*

Procedure D. To a solution of compound **4a** (2.0 mmol) and compound **5** (1.0 mmol) in xylene (5.0 mL) at room temperature 3-methylpyridine (1.0 mmol) was added. The reaction mixture was heated to 160 °C and stirred for 6 h. The reaction progress was monitored by TLC until the disappearance of 7-hydroxycoumarin. Upon completion, the mixture was acidified to pH 7.0 with 2 N aqueous HCl, and extracted with ethyl acetate (10 mL × 3). The combined organic phases were washed with saturated NaCl solution, dried over anhydrous $MgSO_4$, and concentrated under reduced pressure. The crude product was purified by silica gel column chromatography (petroleum ether/ethyl acetate = 5:1) to afford compounds **L1** as a colorless oil. Yield: 65%. ^{1}H NMR (400 MHz, $CDCl_3$) δ 7.58 (d, J = 9.6 Hz, 1H), 7.23 – 7.18 (m, 1H), 6.96 (dd, J = 10.1, 1.2 Hz, 1H), 6.79 – 6.67 (m, 1H), 6.21 (dd, J = 9.5, 2.6 Hz, 1H), 5.75 (dd, J =10.1, 3.4 Hz, 1H), 5.08 – 5.00 (m, 1H), 3.69 – 3.64 (m, 3H), 2.58 – 2.49 (m, 2H), 2.16 –2.01 (m, 2H). ^{13}C NMR (101 MHz, $CDCl_3$) δ 173.39, 160.84, 156.31, 150.03, 143.81,128.04, 124.94, 117.33, 113.23 – 112.75, 109.65, 74.95, 51.75, 30.51, 29.27. Purity: 97.39%. MS (ESI) m/z: calcd for $C_{16}H_{15}O_5$ $[M + H]^+$ 287.09, found 287.25.

*3-(2-Oxo-2H,8H-pyrano[2,3-f]chromen-8-yl)propanoic acid (**L2**).*

Procedure E. Compound **L1** (0.1 mmol) was dissolved in a mixture of THF/H_2O (3:1, 1.0 mL). Lithium hydroxide (LiOH, 0.2 mmol) was added, and the reaction was stirred at room temperature for 1.5 h. Upon completion as monitored by TLC, the mixture was acidified to pH 4.0 with 2 N aqueous HCl, and extracted with ethyl acetate (5 mL × 3). The combined organic layers were washed with saturated aqueous NaCl solution, dried over anhydrous Na_2SO_4, and concentrated under reduced pressure to afford the crude product, which was purification by silica gel column chromatography (dichloromethane/methanol = 30:1) to get the target compound as a white solid. Yield: 89%. ^{1}H NMR (400 MHz, DMSO-d_6) δ 12.17 (s, 1H), 7.94 (d, J = 9.2 Hz, 1H),7.47 (d, J = 7.8 Hz, 1H), 6.79 (d, J = 8.7 Hz, 2H), 6.28 (d, J = 9.2 Hz, 1H), 5.98 (d, J = 8.3 Hz, 1H), 5.21 (d, J = 140.8 Hz, 1H), 2.39 (s, 2H), 1.94 (d, J = 6.0 Hz, 2H). ^{13}C NMR (101 MHz, DMSO-d_6) δ 174.38, 160.29, 156.10, 149.81, 145.08, 129.24, 126.94, 116.38, 113.37 (d, J = 5.7 Hz), 112.93, 109.38, 75.13, 30.47, 29.53. Purity: 99.3%. MS (ESI) m/z: calcd for $C_{15}H_{13}O_5$ $[M +H]^+$ 273.08, found 273.20.

Methyl 3-(6-hydroxy-2-oxo-2H,8H-pyrano[2,3-f]chromen-8-yl)propanoate (**L3**). ***Procedure D***. Yield: 57%. ^{1}H NMR (400 MHz, $CDCl_3$) δ 7.54 (d, J = 9.5 Hz, 1H), 6.95 (d, J = 10.1 Hz, 1H), 6.85 (s, 1H), 6.25 (d, J = 9.5 Hz, 1H), 5.76 (dd, J =10.1, 3.1 Hz,

1H), 5.15 – 5.07 (m, 1H), 3.67 (s, 3H), 2.54 (dd, J = 16.9, 7.1 Hz, 2H), 2.17(ddd, J = 18.3, 11.7, 4.7 Hz, 2H). ^{13}C NMR (101 MHz, $CDCl_3$) δ 174.06, 161.15, 143.78(2C), 143.27, 141.49, 124.72, 117.80, 113.79, 112.63, 112.21, 109.90, 75.99, 51.97, 30.47,30.05. Purity: 95.0%. MS (ESI) m/z: calcd for $C_{16}H_{15}O_6$ $[M + H]^+$ 303.09, found 303.20.

Methyl 4-(6-hydroxy-2-oxo-2H,8H-pyrano[2,3-f]chromen-8-yl)butanoate (**L4**). ***Procedure D***. Yield: 89%. ^{1}H NMR (400 MHz, $CDCl_3$) δ 7.55 (d, J = 9.4 Hz, 1H), 6.95 (dd, J = 10.2, 1.3 Hz, 1H), 6.86 (s, 1H), 6.25 (d, J = 9.5 Hz, 1H), 5.78 (dd, J = 10.1, 3.2 Hz, 1H),5.07 (s, 1H), 3.69 (s, 3H), 2.41 (t, J = 6.6 Hz, 2H), 1.97 – 1.88 (m, 2H), 1.84 (dd, J = 13.4,6.4 Hz, 2H). ^{13}C NMR (101 MHz, $CDCl_3$) δ 174.11, 161.25, 144.05, 143.87, 143.45, 141.56, 125.44, 117.57, 113.90, 112.65, 112.10, 110.14, 76.45, 51.93, 34.60, 33.61, 20.58. Purity: 99.9%. MS (ESI) m/z: calcd for $C_{17}H_{17}O_6$ $[M + H]^+$ 317.10, found 317.25.

Methyl 3-(6-methoxy-2-oxo-2H,8H-pyrano[2,3-f]chromen-8-yl)propanoate (**L5**).

Procedure F. To a solution of **L3** (1.0 mmol) in acetone (5.0 mL) was added potassium carbonate (2.0 mmol) and iodomethane (1.0 mmol). The reaction mixture was then stirred at 56°C for 6 h. After completion of the reaction as monitored by TLC, the mixture was acidified to pH 6.0 with 2 N aqueous HCl, and extracted with ethyl acetate (5 mL × 3). The combined organic layers were washed with saturated aqueous NaCl solution, dried over anhydrous $MgSO_4$, and concentrated to give a crude,, which was purified by silica gel column chromatography (petroleum ether/ethyl acetate = 5:1) afforded the compound **L5** as white solids. Yield: 82%. ^{1}H NMR (400 MHz, $CDCl_3$) δ 7.57 (d, J = 9.4 Hz, 1H), 6.98 (d, J = 10.1 Hz, 1H), 6.77 (s, 1H), 6.27 (d, J = 9.5 Hz, 1H), 5.82 (dd, J =10.1, 3.5 Hz, 1H), 5.18 – 5.05 (m, 1H), 3.88 (s, 3H), 3.68 (s, 3H), 2.56 (t, J = 7.4 Hz, 2H),2.11 (dt, J = 13.2, 6.6 Hz, 2H). ^{13}C NMR (101 MHz, $CDCl_3$) δ 173.33, 161.00, 145.71,145.38, 144.77, 143.63, 125.00, 117.41, 113.46, 111.82, 110.56, 108.85, 75.15, 56.40,51.69, 30.26, 29.18. Purity: 98.0%. MS (ESI) m/z: calcd for $C_{17}H_{17}O_6$ $[M + H]^+$ 317.10, found 317.25.

Methyl 4-(6-methoxy-2-oxo-2H,8H-pyrano[2,3-f]chromen-8-yl)butanoate (**L6**). ***Procedure F.*** Yield: 78%. ^{1}H NMR (400 MHz, $CDCl_3$) δ 7.58 (t, J = 9.3 Hz, 1H), 6.96 (d, J = 10.1 Hz, 1H), 6.76 (d, J = 6.4 Hz, 1H), 6.26 (d, J = 9.4 Hz, 1H), 5.82 (dd, J = 10.1, 3.2 Hz, 1H),5.07 (s, 1H), 3.89 (d, J = 4.0 Hz, 3H), 3.66 (d, J = 4.8 Hz, 3H), 2.38 (t, J = 6.8 Hz, 2H),2.01 – 1.89 (m, 2H), 1.88 – 1.79 (m, 2H). ^{13}C NMR (101

MHz, $CDCl_3$) δ 173.67, 161.12,147.34, 146.02, 145.38, 143.68, 125.44, 117.12, 113.38, 111.72, 110.72, 108.75, 75.94,56.44, 51.57, 34.64, 33.61, 20.15. Purity: 96.0%. MS (ESI) m/z calcd for $C_{18}H_{19}O_6$ $[M + H]^+$ 331.12, found 331.25.

Methyl 3-(6-(2-methoxyethoxy)-2-oxo-2H,8H-pyrano[2,3-f]chromen-8-yl)prop anoate (**L7**). ***Procedure F.*** Yield: 65%. 1H NMR (400 MHz, $CDCl_3$) δ 7.54 (d, J = 9.3 Hz, 1H), 6.95 (d, J = 10.1 Hz, 1H), 6.85 (s, 1H), 6.24 (d, J = 9.4 Hz, 1H), 5.80 (dd, J = 10.1, 3.1 Hz, 1H), 5.08 (s,1H), 4.14 (dd, J = 10.0, 4.9 Hz, 2H), 3.79 – 3.74 (m, 2H), 3.67 (s, 3H), 3.44 (s, 3H), 2.57 (t, J = 7.3 Hz, 2H), 2.15 – 2.02 (m, 2H). ^{13}C NMR (101 MHz, $CDCl_3$) δ 173.42, 161.01,146.59, 145.14, 144.54, 143.63, 125.15, 117.46, 113.50, 112.25, 111.90, 110.83, 74.93,71.03, 69.69, 59.28, 51.72, 30.25, 29.17. Purity: 96.5%. MS (ESI) m/z: calcd for $C_{19}H_{21}O_7$ $[M + H]^+$ 361.13, found 361.30.

4-(6-methoxy-2-oxo-2H,8H-pyrano[2,3-f]chromen-8-yl)propanoic acid (**L8**). ***Procedure E***. Yield: 90%. ^{1}H NMR (400 MHz, DMSO-d_6) δ 7.90 (d, J = 9.5 Hz, 1H), 7.19 (s, 1H), 6.78 (d, J = 11.6 Hz,1H), 6.29 (d, J = 9.5 Hz, 1H), 6.00 (dd, J = 10.1, 3.5 Hz, 1H), 5.03 (dt, J = 6.3, 4.5 Hz, 1H),3.81 (s, 3H), 2.39 (td, J = 7.3, 2.0 Hz, 2H), 1.93 (dd, J = 14.1, 7.4 Hz, 2H). ^{13}C NMR (101MHz, DMSO-d_6) δ 174.37, 160.49, 145.45 (d, J = 14.1 Hz), 145.00, 144.38, 126.92, 116.70,113.35, 112.21, 110.35 (d, J = 18.1 Hz), 74.96, 56.55, 30.24, 29.47. Purity: 99.1%. MS (ESI) m/z: calcd for $C_{16}H_{15}O_6$ $[M + H]^+$ 303.09, found 303.20.

4-(6-(2-methoxyethoxy)-2-oxo-2H,8H-pyrano[2,3-f]chromen-8-yl)propanoic a cid (**L9**). ***Procedure E.*** Yield: 47%. ^{1}H NMR (400 MHz, CD_3OD) δ 7.79 (d, J = 9.5 Hz, 1H), 7.06 (s, 1H), 6.87 (d, J = 10.1 Hz, 1H), 6.24 (d, J = 9.5 Hz, 1H), 5.93 (dd, J =10.1, 3.6 Hz, 1H), 5.09 – 5.03 (m, 1H), 4.18 – 4.14 (m, 2H), 3.79 – 3.76 (m, 2H), 3.45 (s,3H), 2.54 (td, J = 7.4, 3.5 Hz, 2H), 2.11 – 2.00 (m, 2H). ^{13}C NMR (101 MHz,CD_3OD) δ 161.75, 146.34, 144.76, 144.67, 144.59, 125.82, 116.26, 112.29, 112.08, 111.58, 110.40,75.12, 70.75, 68.97, 57.98, 30.00, 28.96. Purity: 99.0%. MS (ESI) m/z: calcd for $C_{18}H_{19}O_7$ $[M + H]^+$ 347.11, found 347.30.

6-hydroxy-8,8-dimethyl-2H,8H-pyrano[2,3-f]chromen-2-one (**9**). ***Procedure D.*** Yield: 48%. ^{1}H NMR (400 MHz, $CDCl_3$) δ 7.56 (d, J = 9.5 Hz, 1H), 6.90 – 6.84 (m, 2H), 6.26 (d, J = 9.5 Hz, 1H), 5.74 (d, J = 10.1 Hz, 1H), 5.51 (s, 1H), 1.51 (s, 6H).

*6-(cyclopropylmethoxy)-8,8-dimethyl-2H,8H-pyrano[2,3-f]chromen-2-one (***L10***)*

Procedure G. A solution of compound 9 (1.0 mmol) in acetone (5.0 mL) was added K_2CO_3 (2.0 mmol) and Iodomethyl-cyclopropane (1.2 mmol). The reaction mixture was stirred at 56 °C for 6 h. After completion of the reaction as monitored by TLC, the mixture was concentrated under reduced pressure, and the residue was purified by silica gel column chromatography (petroleum ether/ethyl acetate = 5:1) afforded the product as white solid.. Yield: 52%. ^{1}H NMR (400 MHz, $CDCl_3$) δ 7.54 (d, J = 9.5 Hz, 1H), 6.85 (d, J = 10.1 Hz, 1H), 6.82 (s, 1H), 6.21 (d, J = 9.4 Hz, 1H), 5.72 (d, J = 10.0 Hz, 1H), 3.84 (d, J = 6.9Hz, 2H), 1.49 (s, 6H), 1.30 – 1.22 (m, 1H), 0.60 (q, J = 5.3 Hz, 2H), 0.33 (q, J = 5.0 Hz,2H). ^{13}C NMR (101 MHz, CDCl3) δ 161.23, 147.31, 145.30, 144.83, 143.76, 130.81,115.28, 113.31, 113.05, 111.55, 110.42, 77.82, 75.50, 28.00 (2C), 10.38, 3.31 (2C). Purity: 99.29%. MS(ESI) m/z: calcd for $C_{18}H_{19}O_4$ $[M + H]^+$ 299.13, found 299.25.

*6-(difluoromethoxy)-8,8-dimethyl-2H,8H-pyrano[2,3-f]chromen-2-one (***L11***)*

Procedure H. To a solution of 9 (1.0 mmol) in a mixture of acetonitrile and water (1:1, 2.0 ml)was added potassium hydroxide (KOH, 20.0 mmol) and diethyl bromofluoromethylphosphonate (1.2 mmol) at 0 ℃. Then reaction mixture was allowed to warm to room temperature and stirred for 2 h. After completion of the reaction as monitored by TLC, the mixture was acidified to pH = 6.0 with 2 N aqueous HCl, and extracted with ethyl acetate (5 mL×3). The combined organic layers were washed with saturated aqueous NaCl solution, dried over anhydrous $MgSO_4$, and concentrated to give a crude,, which was purified by silica gel column chromatography (petroleum ether/ethyl acetate = 5:1) afforded the compound L21 as white solids. Yield: 86%. ^{1}H NMR (400 MHz, $CDCl_3$) δ 7.56 (d, J = 9.5 Hz, 1H), 7.13 (s, 1H), 6.88 (d, J = 10.1 Hz, 1H), 6.55 (t, J = 74.6Hz, 1H), 6.28 (d, J = 9.5 Hz, 1H), 5.79 (d, J = 10.1 Hz, 1H), 1.51 (s, 6H). ^{13}C NMR (101 MHz, $CDCl_3$) δ 160.43, 148.03, 147.58, 143.22, 135.46, 131.40, 120.50, 118.36, 115.76, 114.91, 114.08, 113.15, 111.89, 111.06, 78.77, 28.03 (2C). Purity: 98.43%. MS (ESI) m/z: calcd for $C_{15}H_{13}F_2O_4$ $[M + H]^+$ 295.08, found 295.20.

8,8-dimethyl-6-((4-(trifluoromethyl)benzyl)oxy)-2H,8H-pyrano[2,3-f]chromen- 2-one (**L12**). ***Procedure G.*** Yield: 88%. ^{1}H NMR (400 MHz, $CDCl_3$) δ 7.64 (d, J = 8.1 Hz, 2H), 7.55 (d, J = 8.0 Hz, 2H), 7.50 (d, J = 9.5 Hz, 1H), 6.88 (d, J = 10.1 Hz, 1H),6.79 (s, 1H), 6.22 (d, J = 9.5 Hz, 1H), 5.77 (d, J = 10.1 Hz, 1H), 5.19 (s, 2H), 1.52 (s, 6H).^{13}C NMR (101 MHz, $CDCl_3$) δ 161.12, 147.21, 145.69, 144.21, 143.68, 141.11,

131.09,127.39 (2C), 125.72, 125.68, 125.65, 125.61, 115.35, 113.47, 113.13, 111.65, 110.78,78.20, 77.16, 71.52, 28.18(2C). Purity: 98.3%. MS (ESI) m/z: calcd for $C_{22}H_{18}F_3O_4$ [M + H]$^+$ 403.12,found 403.30.

8,8-dimethyl-6-(pyridin-4-ylmethoxy)-2H,8H-pyrano[2,3-f]chromen-2-one (**L13**). ***Procedure G.*** Yield: 92%. ^{1}H NMR (400 MHz, $CDCl_3$) δ 8.60 (d, J = 5.1 Hz, 2H), 7.48 (d, J = 9.5 Hz, 1H), 7.35 (d, J = 5.2 Hz, 2H), 6.86 (d, J = 10.0 Hz, 1H), 6.77 (s,1H), 6.21 (d, J = 9.4 Hz, 1H), 5.75 (d, J = 10.0 Hz, 1H), 5.13 (d, J = 9.0 Hz, 2H), 1.50 (d, J= 8.3 Hz, 6H). ^{13}C NMR (101 MHz, $CDCl_3$) δ 161.03, 150.05, 147.04, 146.24, 145.69,144.04, 143.60, 131.11, 121.51, 115.27, 113.51, 112.89, 111.61, 110.78, 78.22, 77.16,70.49, 28.16(2C). Purity: 96.5%. MS (ESI) m/z: calcd for $C_{20}H_{18}NO_4$ [M + H]$^+$ 336.12, found 336.25.

Methyl 2-(((8,8-dimethyl-2-oxo-2H,8H-pyrano[2,3-f]chromen-6-yl)oxy)methyl) benzoate (**L14**). ***Procedure G.*** Yield: 90%.^{1}H NMR (400 MHz, $CDCl_3$) δ 8.02 (d, J = 7.8 Hz, 1H), 7.81 (d, J = 7.8 Hz, 1H), 7.56 (t, J = 7.6 Hz,1H), 7.51 (d, J = 9.4 Hz, 1H), 7.37 (t, J = 7.6 Hz, 1H), 6.88 (d, J = 10.0 Hz, 1H), 6.82 (s,1H), 6.21 (d, J = 9.5 Hz, 1H), 5.75 (d, J = 10.0 Hz, 1H), 5.56 (s, 2H), 3.90 (s, 3H), 1.52 (s,6H). 13C NMR (101 MHz, $CDCl_3$) δ 167.40, 161.28, 146.80, 145.30, 144.72, 143.87,139.73, 132.84, 130.98, 130.83, 127.46, 127.39, 127.26, 115.39, 113.20, 111.68, 111.64,110.53, 78.00, 77.16, 69.89, 52.21, 28.14(2C). Purity: 98.7%. MS (ESI) m/z: calcd for $C_{23}H_{21}O_6$ [M + H]$^+$393.13, found 393.30.

Methyl 3-(((8,8-dimethyl-2-oxo-2H,8H-pyrano[2,3-f]chromen-6-yl)oxy)methyl) benzoate (**L15**). ***Procedure G.*** Yield: 90%. ^{1}H NMR (400 MHz, $CDCl_3$) δ 8.12 (s, 1H), 7.99 (d, J = 7.6 Hz, 1H), 7.63 (d, J = 7.6 Hz, 1H), 7.49(d, J = 9.5 Hz, 1H), 7.45 (t, J = 7.7 Hz, 1H), 6.87 (d, J = 10.1 Hz, 1H), 6.80 (s, 1H), 6.21 (d, J = 9.5 Hz, 1H), 5.76 (d, J = 10.1 Hz, 1H), 5.17 (s, 2H), 3.91 (s, 3H), 1.53 (s, 6H). ^{13}C NMR (101 MHz, $CDCl_3$) δ 166.94, 161.18, 147.35, 145.65, 144.28, 143.74, 137.50,131.85, 131.07, 130.63, 129.34, 128.82, 128.58, 115.34, 113.51, 113.36, 111.64, 110.72,78.17, 77.16, 71.95, 52.30, 28.16(2C). Purity: 99.1%MS (ESI) m/z: calcd for $C_{23}H_{21}O_6$ [M + H]$^+$ 393.13, found 393.25.

Methyl 4-(((8,8-dimethyl-2-oxo-2H,8H-pyrano[2,3-f]chromen-6-yl)oxy)methyl) benzoa te (**L16**). ***Procedure G.*** Yield: 91%. ^{1}H NMR (400 MHz, $CDCl_3$) δ 8.04 (d, J = 7.7 Hz, 2H), 7.49 (t, J = 8.2 Hz, 3H), 6.87 (d, J = 10.1 Hz,1H), 6.77 (s, 1H), 6.21 (d, J = 9.5 Hz, 1H), 5.76 (d, J = 10.0 Hz, 1H), 5.19 (s, 2H), 3.91 (s,3H), 1.53 (s, 6H). ^{13}C NMR (101 MHz, $CDCl_3$) δ 166.91, 161.17, 147.19, 1

45.61, 144.24,143.71, 142.19, 131.08, 130.00(2C), 129.90, 127.00(2C), 115.35, 113.41, 113.11, 111.62,110.73, 78.18, 77.16, 71.70, 52.29, 28.19(2C). Purity: 9 7.8%. MS (ESI) m/z: calcd for $C_{23}H_{21}O_6$ [M + H]$^+$ 393.13, found 393.30.

3-(((8,8-dimethyl-2-oxo-2H,8H-pyrano[2,3-f]chromen-6-yl)oxy)methyl)benzoic acid (**L17**). ***Procedure E***. Yield:71%. ^{1}H NMR (400 MHz, DMSO-*d6*) *δ* 8.06(s,1H),7.89(s, 2H),7.59 (d, *J* = 62.0 Hz, 2H), 7.29 (s, 1H), 6.72 (s, 1H), 6.28 (s, 1H), 5.93 (s, 1H),5.21 (s, 2H),1.44 (s, 6H). ^{13}C NMR (101 MHz, DMSO-*d6*) *δ* 167.34, 159.99, 145.81, 144.46, 143.75,137.37, 131.68, 128.78, 128.67, 128.48, 128.44, 114.31, 112.85, 111.48, 109.49, 77.68,70.36, 39.52, 27.51(2C). Purity: 98.9%. MS (ESI) m/z: calcd for $C_{22}H_{19}O_6$ [M + H]$^+$ 379.12, found 379.30.

4-(((8,8-dimethyl-2-oxo-2H,8H-pyrano[2,3-f]chromen-6-yl)oxy)methyl)benzoic acid (**L18**). ***Procedure E***. Yield:75%. ^{1}H NMR (400 MHz, DMSO-*d6*) *δ* 7.96 (d, *J* = 6.9 Hz, 2H), 7.86 (d, *J* = 9.2 Hz, 1H), 7.56 (d, *J* = 7.0 Hz, 2H), 7.27 (s, 1H), 6.72 (d, *J* = 9.7 Hz, 1H),6.28 (d, *J* = 9.1 Hz, 1H), 5.94 (d, *J* = 9.6 Hz, 1H), 5.23 (s, 2H), 1.45 (s, 6H). ^{13}C NMR (101MHz, DMSO-*d6*) *δ* 167.12, 159.97, 145.73, 144.45, 143.67, 141.82, 131.72, 130.47,129.47(2C), 127.37(2C), 114.30, 112.86, 112.70, 111.47, 109.47, 77.70, 70.15, 39.52,27.53(2C). Purity: 99.6%. MS (ESI) m/z: calcd for $C_{22}H_{19}O_6$ [M + H]$^+$ 379.12, found 379.30.

Ethyl 4-((2-oxo-2H-chromen-7-yl)oxy)butanoate (**L19**).

Procedure I. To a solution of compound 10a (1.0 mmol) in DMF (5.0 ml) was added compound 5 (1.0 mmol) and K_2CO_3 (1.5 mmol). The reaction mixture was heated to 80 °C and stirred for 3 h. Upon completion as monitored by TLC, the mixture was extracted with ethyl acetate (5 mL × 3). The combined organic layers were washed with saturated NaCl solution, dried over anhydrous Na_2SO_4, and concentrated to give a crude, . which was purified by silica gel column chromatography (petroleum ether/ethyl acetate = 20:1) to get the target compound as a white solid. Yield: 96%. ^{1}H NMR (400 MHz,$CDCl_3$) δ 7.61 (d, J = 9.5 Hz, 1H), 7.34 (d, J = 8.5 Hz, 1H), 6.81 (dd, J = 8.5, 2.4 Hz, 1H), 6.77 (d, J = 2.4 Hz, 1H), 6.22 (d, J = 9.5 Hz, 1H), 4.14 (q, J = 7.1 Hz, 2H), 4.07 (s, 0H), 2.51 (t, J = 7.2 Hz, 2H), 2.20 – 2.08 (m, 2H), 1.24 (t, J = 7.1 Hz, 3H).^{13}C NMR (101 MHz, $CDCl_3$) δ 172.97, 162.14, 161.20, 155.95, 143.45, 128.86, 113.17, 112.83, 112.67, 101.56, 67.47, 60.59, 30.69, 24.44, 14.28. Purity: 99.59%. MS (ESI) m/z: calcd for $C_{15}H_{16}O_5$ [M + H]$^+$ 277.10, found 277.30.

Methyl 5-((2-oxo-2H-chromen-7-yl)oxy)pentanoate (**L20**). ***Procedure I.*** Yield: 80%. ^{1}H NMR (400 MHz, $CDCl_3$) δ 7.62 (dd, J = 9.5, 2.4 Hz, 1H), 7.35 (dd, J = 8.5, 2.5 Hz, 1H), 6.85 – 6.75 (m, 2H), 6.23 (dd, J = 9.5, 2.9 Hz, 1H), 4.06 – 3.98 (m, 2H), 3.67 (d, J = 2.7 Hz, 3H), 2.44 – 2.38 (m, 2H), 1.84 (dd, J = 6.5, 3.3 Hz, 4H). ^{13}C NMR (101 MHz, $CDCl_3$) δ 173.34, 161.86, 160.80, 155.54, 143.11, 128.46, 112.63, 112.49, 112.16, 101.01, 67.71, 51.20, 33.20, 28.04, 21.18. Purity: 99.0%. MS (ESI) m/z: calcd for $C_{15}H_{16}O_5$ $[M + H]^+$ 277.10, found 277.35.

Methyl 6-((2-oxo-2H-chromen-7-yl)oxy)hexanoate (**L21**). ***Procedure I.*** Yield: 98%. ^{1}H NMR (400 MHz, $CDCl_3$) δ 7.61 (d, J = 9.5 Hz, 1H), 7.34 (d, J = 8.6 Hz, 1H), 6.84 – 6.74 (m, 2H), 6.22 (d, J = 9.5 Hz, 1H), 4.00 (t, J = 6.4 Hz, 2H), 3.66 (s, 3H), 2.34 (t, J = 7.4 Hz, 2H), 1.88 – 1.76 (m, 2H), 1.70 (p, J = 7.5 Hz, 2H), 1.56 – 1.44 (m, 2H). ^{13}C NMR (101 MHz, $CDCl_3$) δ 173.74, 162.09, 161.03, 155.69, 143.23, 128.52, 112.75, 112.69, 112.23, 68.07, 51.30, 33.67, 28.44, 25.34, 24.38. Purity: 100%. MS (ESI) m/z: calcd for $C_{16}H_{18}O_5$ $[M + H]^+$ 291.12, found 291.40.

Ethyl 4-((6-hydroxy-2-oxo-2H-chromen-7-yl)oxy)butanoate (**L22**). ***Procedure I.*** Yield: 12%. ^{1}H NMR (400 MHz, $CDCl_3$) δ 7.58 (d, J = 9.5 Hz, 1H), 6.95 (s, 1H), 6.79 (s, 1H), 6.26 (d, J = 9.5 Hz, 1H), 4.15 (t, J = 6.2 Hz, 4H), 2.53 (t, J = 6.8 Hz, 2H), 2.21 (p, J = 6.3 Hz, 2H), 1.25 (t, J = 7.2 Hz, 3H).^{13}C NMR (101 MHz, $CDCl_3$) δ 173.71, 161.57, 149.52, 149.20, 143.51, 143.18, 113.99, 112.51, 111.43, 100.20, 68.91, 60.98, 31.34, 24.38, 14.27. Purity: 98.1%. MS (ESI) m/z: calcd for $C_{15}H_{16}O_6$ $[M + H]^+$ 293.10, found 293.35.

Methyl 5-((6-hydroxy-2-oxo-2H-chromen-7-yl)oxy)pentanoate (**L23**). ***Procedure I.*** Yield: 13%. ^{1}H NMR (400 MHz, $CDCl_3$) δ 7.58 (d, J = 9.5 Hz, 1H), 6.96 (s, 1H), 6.78 (s, 1H), 6.27 (d, J = 9.5 Hz, 1H), 4.12 (t, J = 5.5 Hz, 2H), 3.71 (s, 3H), 2.46 (t, J = 6.6 Hz, 2H), 1.89 (ddt, J = 12.5, 8.8, 6.4 Hz, 5H). ^{13}C NMR (101 MHz, $CDCl_3$) δ 173.87, 161.30, 149.34, 148.95, 143.22, 142.79, 113.62, 112.06, 111.07, 99.66, 69.01, 51.62, 33.43, 27.72, 21.81. Purity: 99.8%. MS (ESI) m/z: calcd for $C_{15}H_{16}O_6$ $[M + H]^+$ 293.10, found 293.35.

Methyl 6-((6-hydroxy-2-oxo-2H-chromen-7-yl)oxy)hexanoate (**L24**). ***Procedure I.*** Yield: 11%. ^{1}H NMR (400 MHz, $CDCl_3$) δ 7.57 (d, J = 9.5 Hz, 1H), 6.94 (s, 1H), 6.77 (s, 1H), 6.25 (d, J = 9.5 Hz, 1H), 5.95 (s, 1H), 4.09 (t, J = 6.3 Hz, 2H), 3.66 (s, 3H), 2.35 (t, J = 7.3 Hz, 2H), 1.87 (p, J = 6.7 Hz, 2H), 1.70 (p, J = 7.4 Hz, 2H), 1.52 (qd, J = 10.2, 9.2, 6.2 Hz, 2H). ^{13}C NMR (101 MHz, $CDCl_3$) δ 174.11, 161.63, 149.69, 149.26, 143.54,

142.98, 113.79, 112.22, 111.27, 100.04, 69.26, 51.70, 33.81, 28.44, 25.49, 24.52. Purity: 96.8%. MS (ESI) m/z: calcd for $C_{16}H_{18}O_6$ [M + H]$^+$ 307.11, found 307.40.

4-((2-oxo-2H-chromen-7-yl)oxy)butanoic acid (**L25**). ***Procedure E***. Yield: 27%. ^{1}H NMR (400 MHz, $CDCl_3$) δ 7.98 (d, J = 9.5 Hz, 1H), 7.62 (d, J = 8.6 Hz, 1H), 7.00 – 6.91 (m, 2H), 6.28 (d, J = 9.5 Hz, 1H), 4.09 (t, J = 6.4 Hz, 2H), 2.39 (t, J = 7.3 Hz, 2H), 1.96 (p, J = 6.9 Hz, 2H). ^{13}C NMR (101 MHz, $CDCl_3$) δ 174.06, 161.72, 160.34, 155.43, 144.37, 129.54, 112.72, 112.49, 112.37, 101.21, 67.45, 30.02, 24.00. Purity: 99.2%MS (ESI) m/z: calcd for $C_{13}H_{12}O_5$ [M + H]$^+$ 249.07, found 249.35.

5-((2-oxo-2H-chromen-7-yl)oxy)pentanoic acid (**L26**). ***Procedure E***. Yield: 28%. ^{1}H NMR (400 MHz, DMSO-d_6) δ 7.97 (d, J = 9.5 Hz, 1H), 7.60 (d, J = 8.5 Hz, 1H), 6.98 – 6.89 (m, 2H), 6.26 (d, J = 9.5 Hz, 1H), 4.06 (t, J = 6.3 Hz, 2H), 2.20 (t, J = 7.3 Hz, 2H), 1.73 (dq, J = 11.5, 6.5 Hz, 2H), 1.68 – 1.56 (m, 2H).^{13}C NMR (101 MHz, DMSO-d_6) δ 161.91, 160.36, 155.45, 144.39, 129.49, 112.75, 112.38, 112.26, 101.15, 68.14, 34.39, 28.08, 21.55. Purity: 98.8%. MS (ESI) m/z: calcd for $C_{14}H_{14}O_5$ [M + H]$^+$ 263.09, found 263.30.

6-hydroxy-8,8-dimethyl-2-oxo-2H,8H-pyrano[2,3-f]chromene-5-carbaldehyde (**11**).

Procedure J. Dissolve hamethylenetetramine (1.02 mmol) and **9** (1.0 mmol) in trifluoroacetic acid (6.0 mL), stir at 0°C , and react at 72°C for 6 hours. After the TLC monitoring reaction was completed, saturated $NaHCO_3$ was neutralized to pH 7.0, and the white solid product **11** was purified through post-treatment. Yield: 37%. ^{1}H NMR (400 MHz, $CDCl_3$) δ 11.42 (s, 1H), 10.45 (s, 1H), 8.30 (d, J = 9.8 Hz, 1H), 6.92 (d, J = 10.2 Hz, 1H), 6.46 (d, J = 9.8 Hz, 1H), 5.94 (d, J = 10.1 Hz, 1H).

2,2,11,11-tetramethyl-2,11-dihydro-6H-dipyrano[2,3-f:3',2'-h]chromen-6-one (**L27**). ***Procedure D***. Yield: 56%. ^{1}H NMR (400 MHz, $CDCl_3$) δ 7.80 (d, J = 9.7 Hz, 1H), 6.85 (d, J = 10.0 Hz, 1H), 6.61 (d, J = 10.0 Hz, 1H), 6.25 (d, J = 9.7 Hz, 1H), 5.81 (d, J = 10.0 Hz, 1H), 5.72 (d, J = 10.0 Hz, 1H), 1.46 (d, J = 11.3 Hz, 12H). ^{13}C NMR (101 MHz, $CDCl_3$) δ 161.26, 145.41, 144.74, 139.31, 138.43, 133.77, 131.16, 117.49, 117.06, 115.54, 112.92, 110.22, 107.60, 77.62, 77.16, 76.02, 27.94(2C), 27.18(2C). Purity: 95.1%. MS (ESI) m/z: calcd for $C_{19}H_{19}O_4$ [M + H]$^+$ 331.13, found 331.35.

Methyl 3-(10-formyl-2,2-dimethyl-6-oxo-6,11-dihydro-2H-dipyrano[2,3-f:3',2'-h]chromen-11-yl)propanoate (**L28**). ***Procedure K.*** Compound 11 (1.0 mmol) and sodium carbonate (1.5 mmol) were dissolved in a mixture of acetone/water or 1,4-dioxane/water (2:1, 2.0 mL) at room temperature. Compound 3a(1.5 mmol) was

added dropwise, and the reaction mixture was stirred at 56 °C for 72 h.The m ixture was then extracted with portions of ethyl acetate (2 × 50 mL). The co mbined organic extracts were dried over anhydrous sodium sulfate and concentr ated to give a crude, which was purified by silica gel column chromatography (petroleum ether/EtOAc, 6:1 to 2:1) to afford the desired product as a yellow solid. Yield: 46%. ^{1}H NMR (400 MHz, $CDCl_3$) δ 9.65 (s, 1H), 7.87 (d, J = 9. 8 Hz, 1H), 7.52 (s, 1H), 6.88 (d, J = 10.1 Hz, 1H), 6.38 (d, J = 9.7 Hz, 1H), 5.85 (d, J = 10.1 Hz, 1H), 5.40 (dd, J = 10.1, 3.7 Hz, 1H), 3.64 (s, 3H), 2. 57 – 2.47 (m, 2H), 2.07 – 2.00 (m, 1H), 1.89 – 1.82 (m, 1H), 1.52 (s, 6H).^{13}C NMR (101 MHz, $CDCl_3$) δ 189.44, 173.14, 160.36, 145.45, 145.29, 139.59, 138.12, 136.03,134.15, 133.13, 115.91, 115.20, 114.75, 113.70, 108.98, 78.44, 7 7.16, 72.10, 51.85, 29.81,28.20, 28.09, 27.98. Purity: 99.39%. MS (ESI) m/z: c alcd for $C_{22}H_{21}O_7$ [M + H]$^+$ 397.13, found 397.30.

2,2,11,11-tetramethyl-6-oxo-6,11-dihydro-2H-dipyrano[2,3-f:3',2'-h]chromene-10-carbaldehyde (**L29**). ***Procedure K.*** Yield:55%. ^{1}H NMR (400 MHz, $CDCl_3$) *δ* 9.55 (s, 1H), 7.89 (d, *J* = 9.7 Hz, 1H), 7.39 (s, 1H), 6.87 (d, *J* = 10.0 Hz, 1H), 6.36 (d, *J* = 9.7 Hz, 1H), 5.83 (d, *J* = 10.0 Hz, 1H), 1.64 (s,6H), 1.50 (s, 6H). ^{13}C NMR (101 MHz, $CDCl_3$) *δ* 189.89, 160.60, 145.24, 145.13, 140.35,140.16, 138.19, 135.72, 133.03, 115.35, 115.22, 114.37, 113.68, 108.51, 78.77, 78.09,77.16, 28.00(2C), 26.05(2C). Purity: 98.6%. MS (ESI) m/z: calcd for $C_{20}H_{19}O_5$ [M + H]$^+$ 339.12, found 339.30.

10-(hydroxymethyl)-2,2,11,11-tetramethyl-2,11-dihydro-6H-dipyrano[2,3-f:3', 2'-h]chromen-6-one (**L30**).

Procedure L. A solution of **L29** (1.0 mmol) and 4-(2-Aminoethyl)morpholine in tetrahydrofuran (THF, 3.0 mL) was added sodium triacetoxyborohydride (1.5 mmol) portionwise at 0°C. The reaction was allowed to warm to room temperature and stirred for 1.5 h. Upon complete consumption of **L29**, a small amount of water was added to quench the reaction. The mixture was then extracted with portions of ethyl acetate (2 × 50 mL). The combined organic extracts were dried over anhydrous sodium sulfate and concentrated to give a crude, which was purified by silica gel column chromatography (petroleum ether/EtOAc, 6:1 to 2:1) to afford the desired product as a white solid. Yield: 80%. ^{1}H NMR (400 MHz, $CDCl_3$) *δ* 7.88 (d, *J* = 9.7 Hz, 1H), 6.88 (d, *J* = 10.0 Hz, 1H), 6.72 (s,1H), 6.26 (d, *J* = 8.8 Hz, 1H), 5.73 (d, *J* = 10.0 Hz, 1H), 5.30 (s, 1H), 4.34 (s, 2H), 1.49 (s,12H). ^{13}C NMR (101 MHz, $CDCl_3$) *δ* 161.52, 145.43, 144.87, 143.65,

139.62, 137.76,131.09, 118.45, 115.54, 113.04, 112.68, 109.98, 107.58, 77.68, 77.48, 77.16, 62.40,27.95(2C), 25.33(2C). Purity: 98.0%. MS (ESI) m/z: calcd for $C_{20}H_{21}O_5$ $[M + H]^+$ 341.14, found 341.30.

2,2,11,11-tetramethyl-10-(((2-morpholinoethyl)amino)methyl)-2,11-dihydro-6H-dipyrano[2,3-f:3',2'-h]chromen-6-one (**L31**).

Procedure M. To a solution of **L29** (1.0 mmol) in tetrahydrofuran (THF, 3.0 mL) was added sodium triacetoxyborohydride (1.5 mmol) portionwise at 0°C. The reaction was allowed to warm to room temperature and stirred for 1.5 h. Upon complete consumption of **L29**, a small amount of water was added to quench the reaction. The mixture was then extracted with portions of ethyl acetate (2 × 50 mL). The combined organic extracts were dried over anhydrous sodium sulfate and concentrated to give a crude, which was purified by silica gel column chromatography (petroleum ether/EtOAc, 6:1 to 2:1) to afford the desired product as a white solid. Yield:86%. ^{1}H NMR (400 MHz, $CDCl_3$) δ 7.89 (d, J = 8.4 Hz, 1H), 6.86 (d, J = 9.9 Hz, 1H), 6.66 (s, 1H), 6.24 (d, J = 9.5 Hz, 1H), 5.71 (d, J = 9.9 Hz,1H), 3.71 (s, 4H), 3.40 (s, 2H), 2.77 (s, 2H), 2.55 (s, 2H), 2.48 (s, 2H), 2.12 (s, 2H), 1.47 (d,J = 4.7 Hz, 12H). ^{13}C NMR (101 MHz, $CDCl_3$) δ 161.40, 145.39, 144.91, 139.62, 137.57,130.93, 118.77, 115.63, 113.32, 112.67, 109.81, 107.44, 78.06, 77.60, 77.16, 67.04, 58.38,53.93, 50.98, 45.94, 27.95(2C), 25.24(2C). Purity: 97.8%. MS (ESI) m/z: calcd for $C_{26}H_{32}N_2O_5$ $[M + H]^+$ 453.24, found 453.40.

10-(((cyclopropylmethyl)amino)methyl)-2,2,11,11-tetramethyl-2,11-dihydro-6H-dipyrano[2,3-f:3',2'-h]chromen-6-one (**L32**). ***Procedure M.*** Yield:82%. ^{1}H NMR (400 MHz, $CDCl_3$) δ 7.93 (d, J = 9.3 Hz, 1H), 6.87 (d, J = 9.8 Hz, 1H), 6.72 (s, 1H), 6.25 (d, J = 9.4 Hz, 1H), 5.71 (d, J = 9.7 Hz,1H), 3.44 (s, 2H), 2.57 (d, J = 6.0 Hz, 2H), 1.48 (d, J = 5.1 Hz, 12H), 1.02 (s, 1H), 0.54 (d, J = 6.2 Hz, 2H), 0.17 (s, 2H). ^{13}C NMR (101 MHz, $CDCl_3$) δ 161.43, 145.40, 144.95,142.75, 139.67, 137.56, 130.94, 118.76, 115.67, 113.25, 112.73, 109.85, 107.50, 78.04,77.62, 77.16, 54.74, 50.27, 27.98(2C), 25.24(2C), 11.03, 3.69(2C). Purity: 95.3%. MS (ESI) m/z: calcd for $C_{24}H_{27}NO_4$ $[M + H]^+$ 393.19, found 391.25.

3-(((2,2,11,11-tetramethyl-6-oxo-6,11-dihydro-2H-dipyrano[2,3-f:3',2'-h]chromen-10-yl)methyl)amino)propanenitrile (**L33**). ***Procedure M.*** ^{1}H NMR (400 MHz, $CDCl_3$) δ 7.92 (d, J = 9.7 Hz, 1H), 6.87 (d, J= 10.0 Hz, 1H), 6.74 (s, 1H), 6.26 (d, J = 9.7 Hz, 1H), 5.72 (d, J = 10.0 Hz, 1H), 3.46 (s,2H), 3.00 (t, J = 6.3 Hz, 2H), 2.60 (t, J = 6.3 Hz,

2H), 1.48 (d, J = 3.8 Hz, 12H). ^{13}C NMR (101 MHz, $CDCl_3$) δ 161.39, 145.41, 144.96, 141.91, 139.62, 137.69, 131.07, 118.75,118.50, 115.64, 114.12, 112.89, 110.05, 107.55, 77.94, 77.67, 77.16, 50.28, 44.81,27.98(2C), 25.27(2C), 19.00. Purity: 98.9%. MS (ESI) m/z: calcd for $C_{23}H_{24}N_2O_4$ $[M + H]^+$ 392.17, found 391.35.

4. ^{1}H NMR, ^{13}C NMR and MS data for tested compounds.

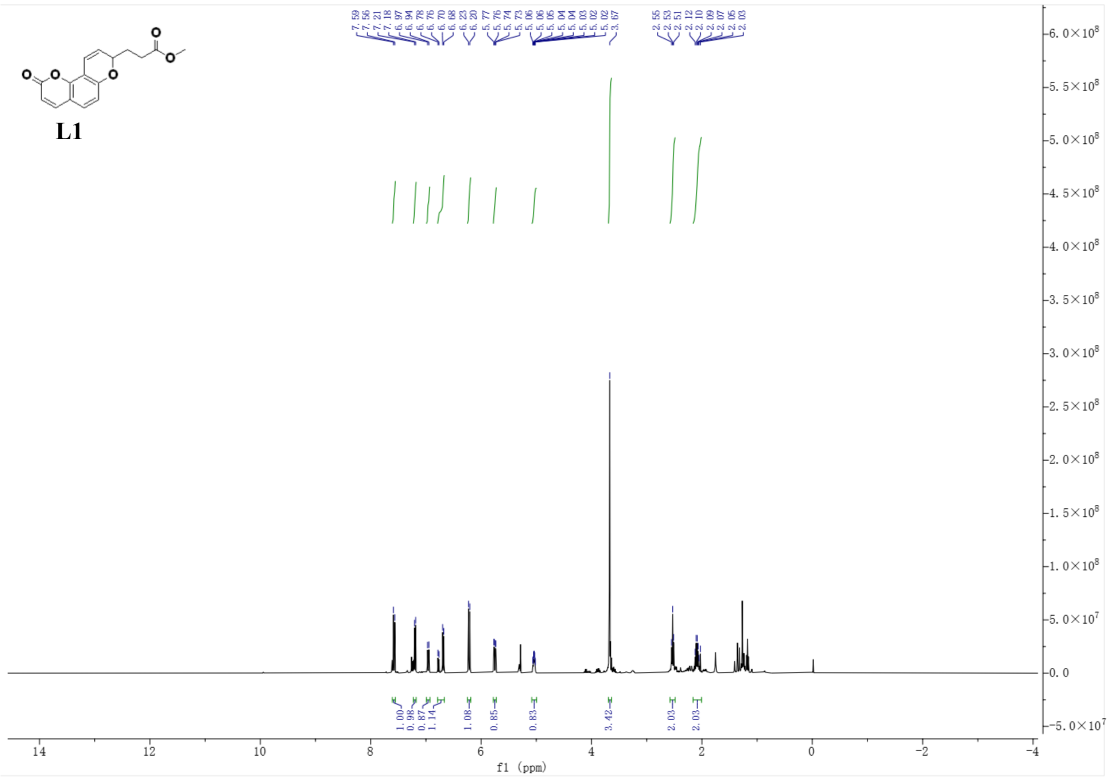

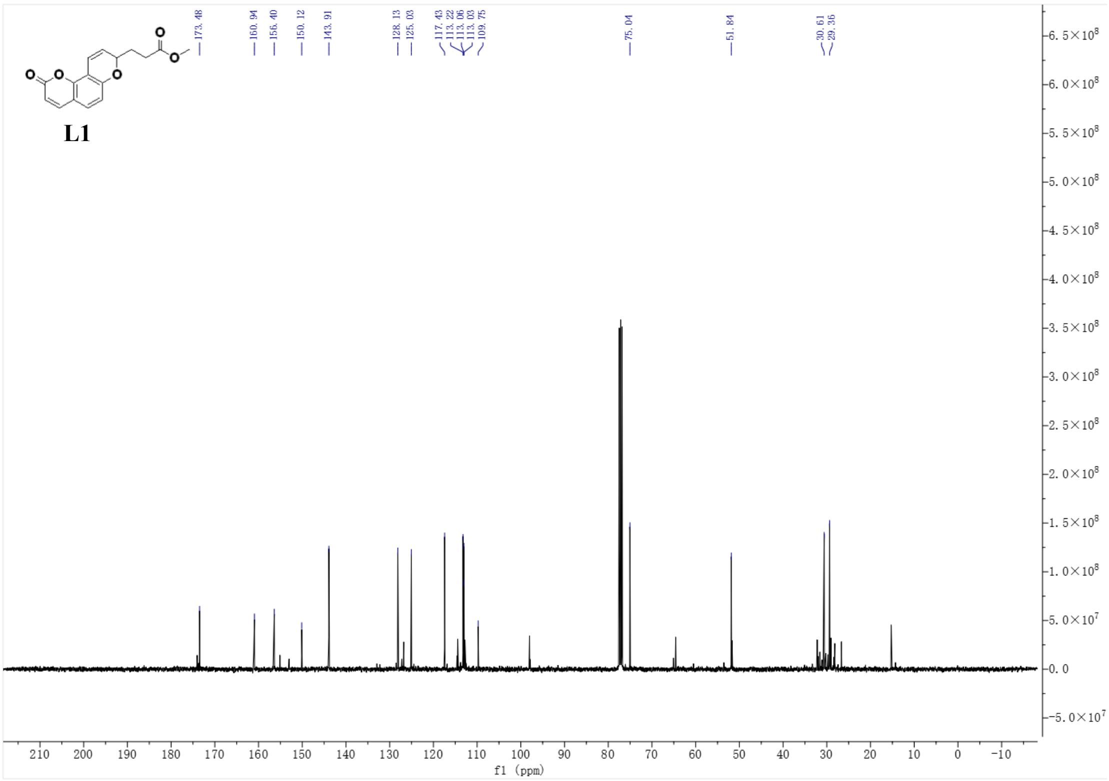

^{1}H NMR and ^{13}C NMR spectroscopic data of **L1**

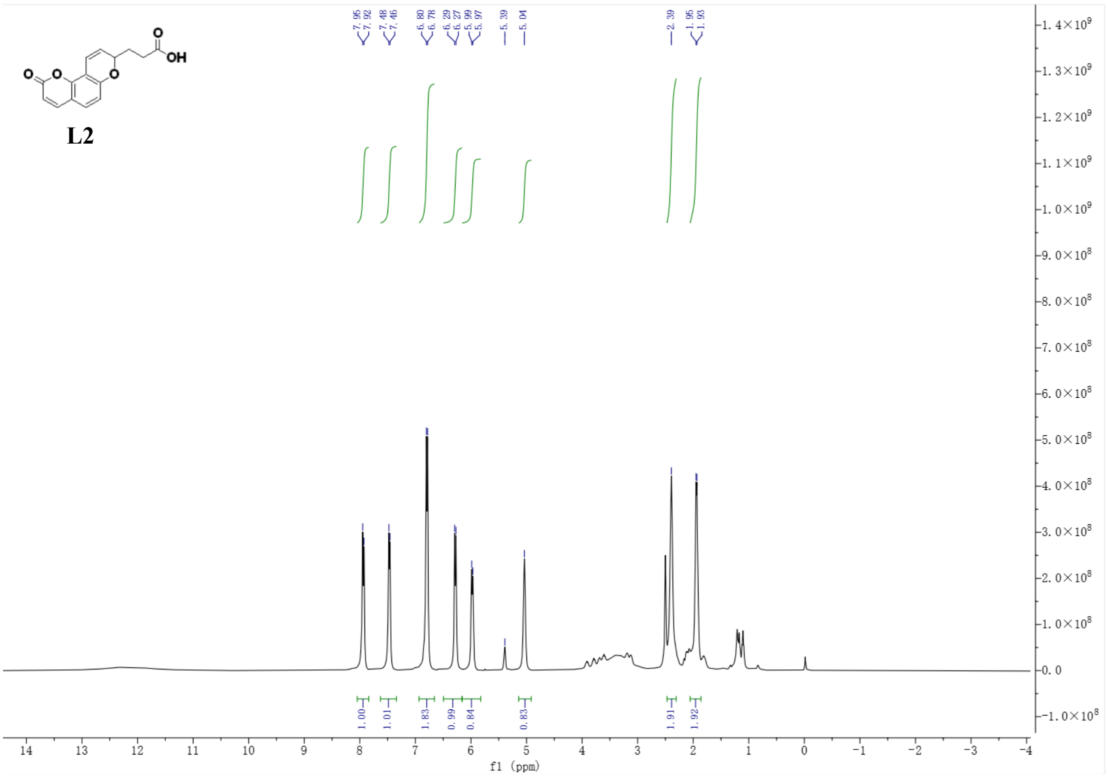

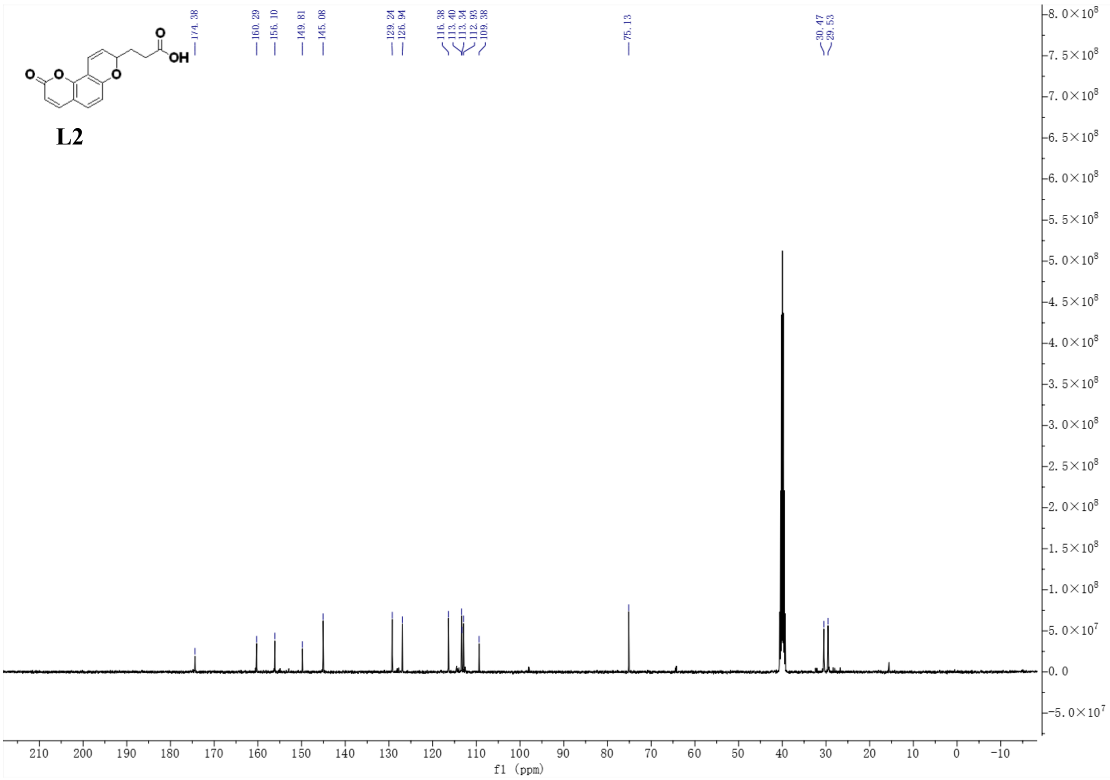

^{1}H NMR and ^{13}C NMR spectroscopic data of **L2**

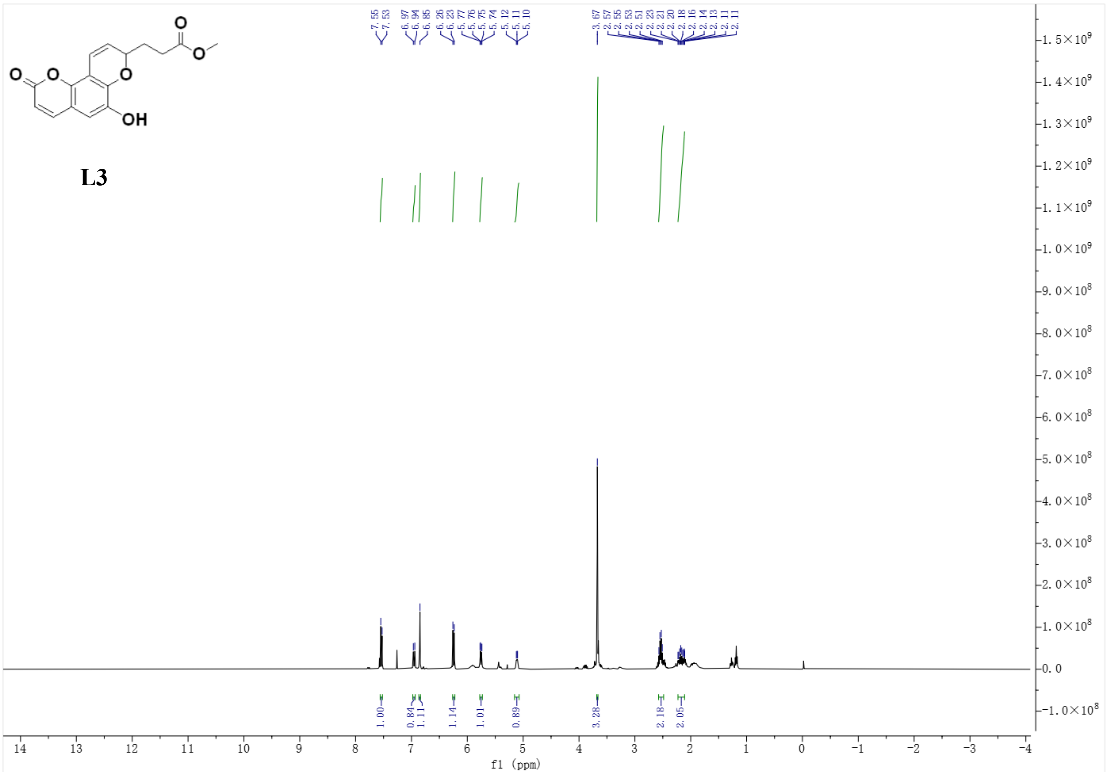

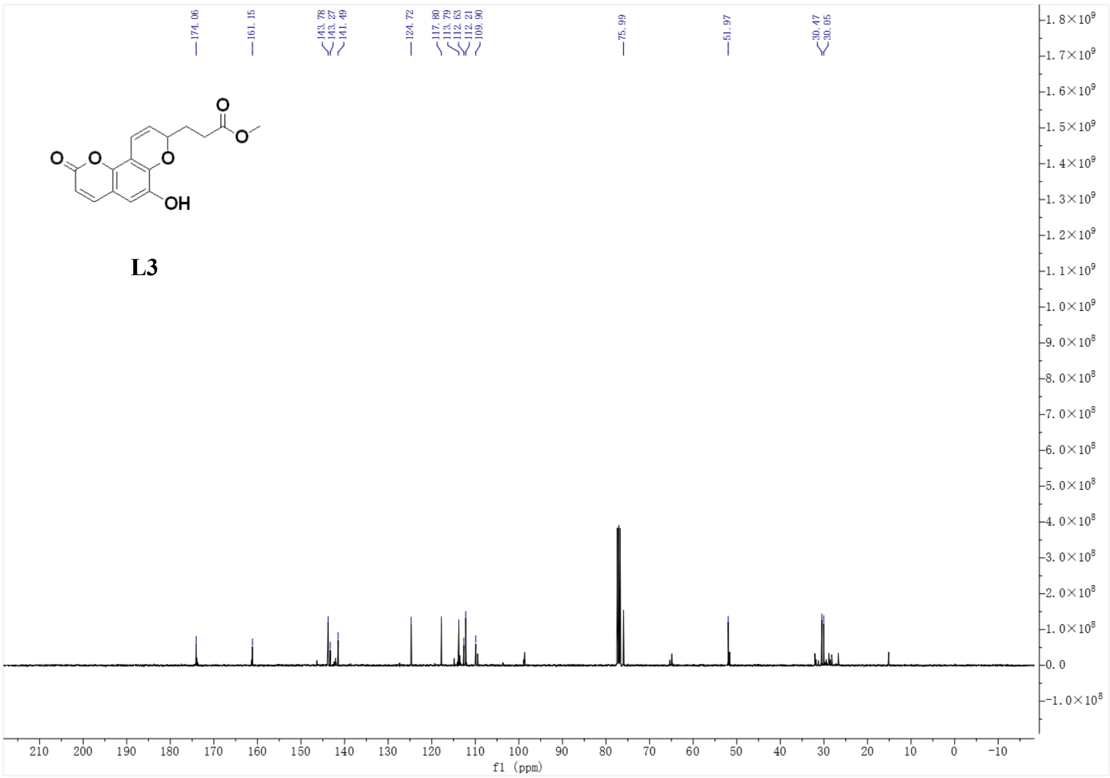

^{1}H NMR and ^{13}C NMR spectroscopic data of **L3**

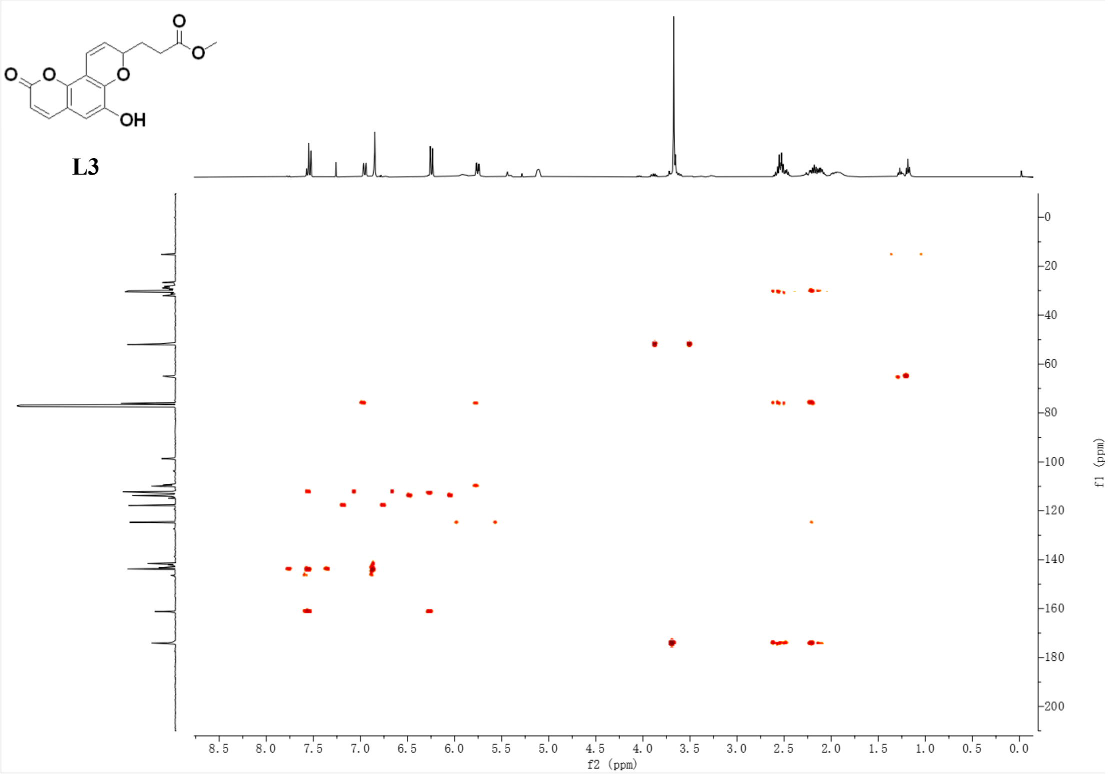

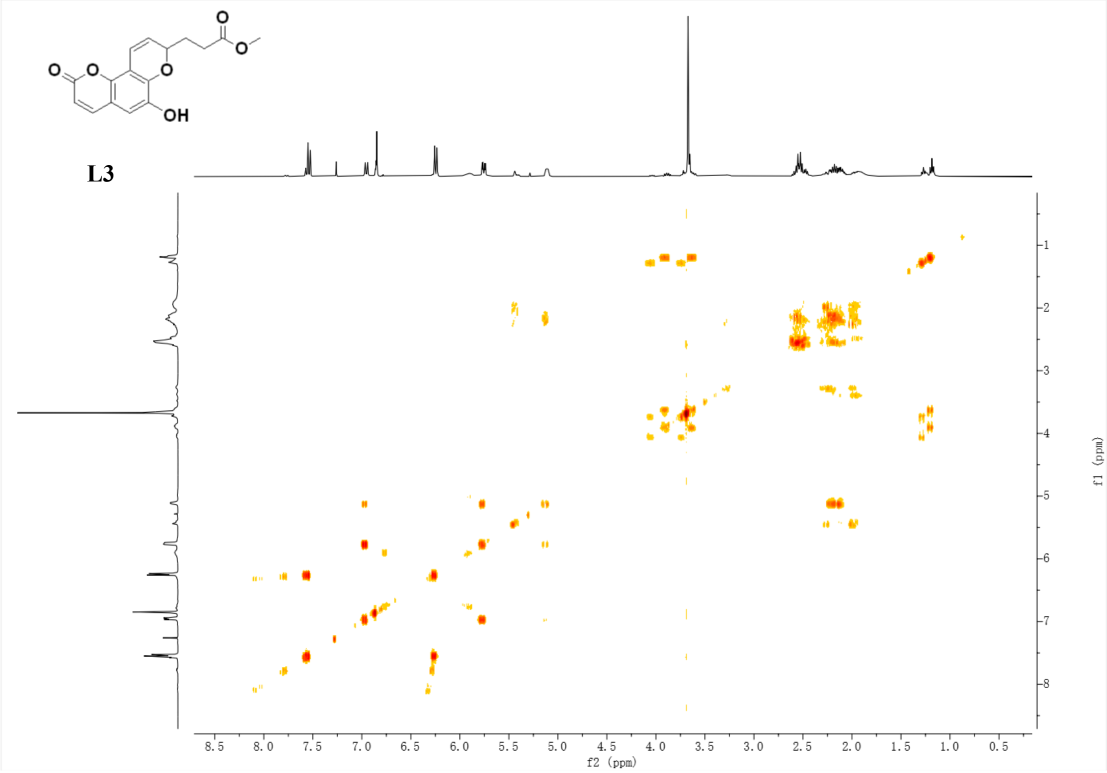

HMBC and NOESY spectroscopic data of **L3**

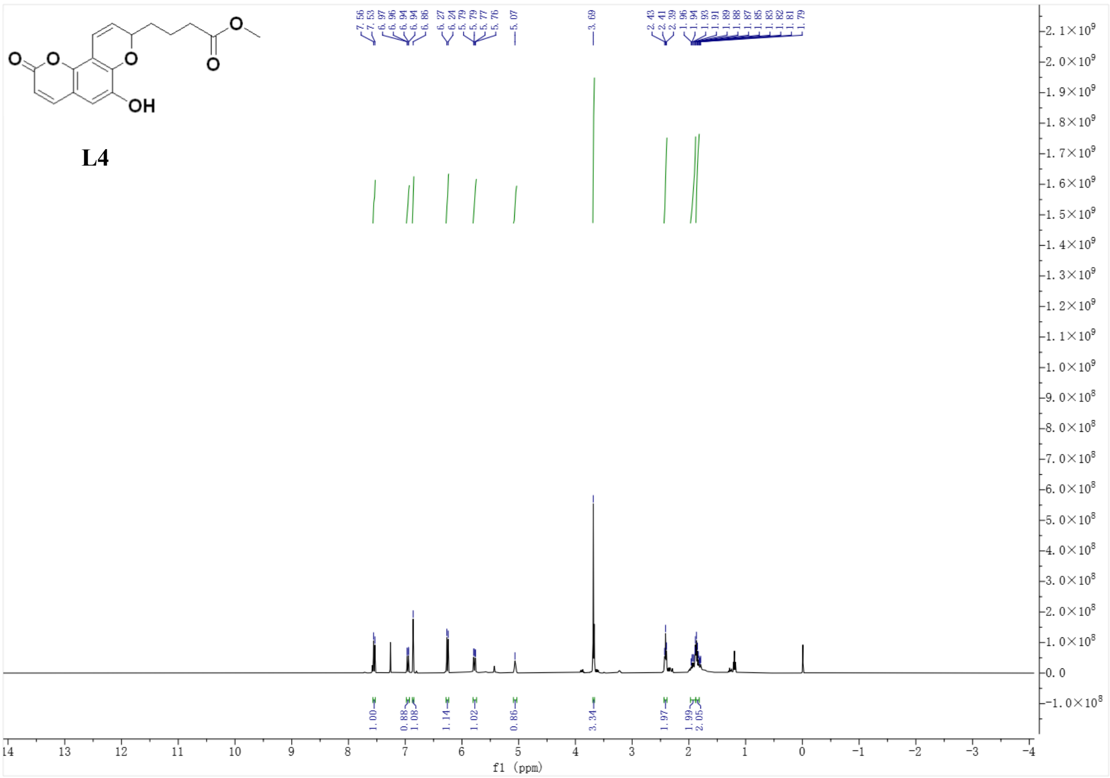

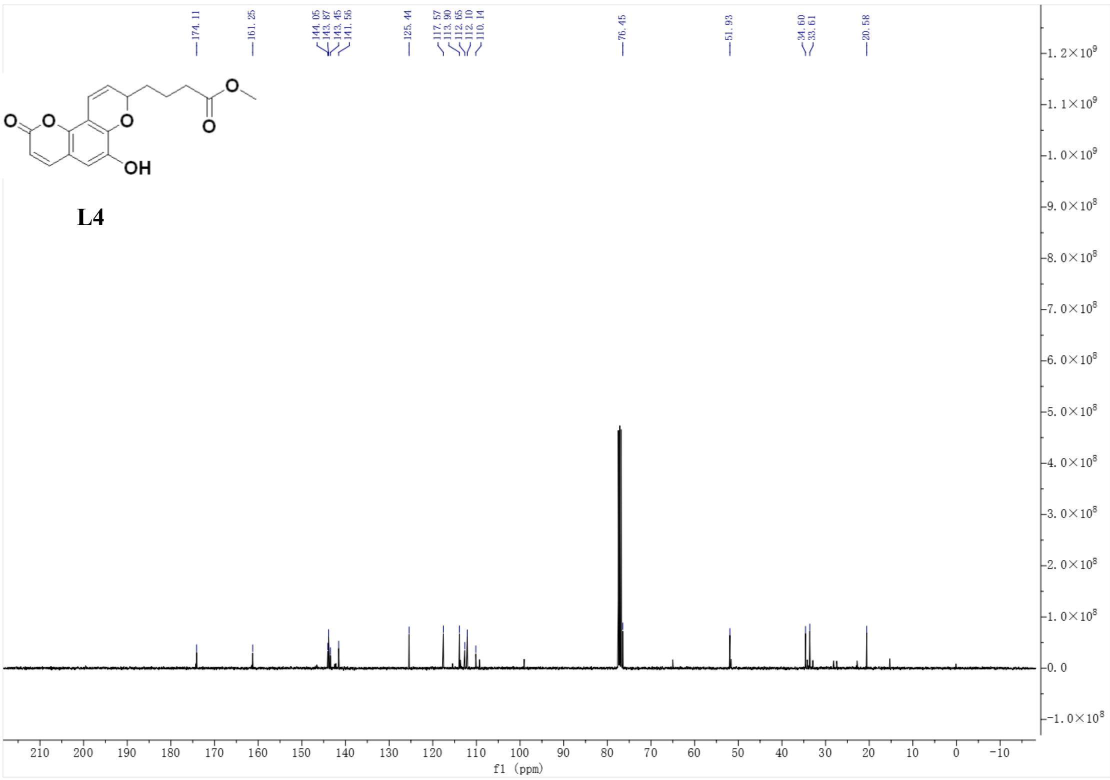

^{1}H NMR and ^{13}C NMR spectroscopic data of **L4**

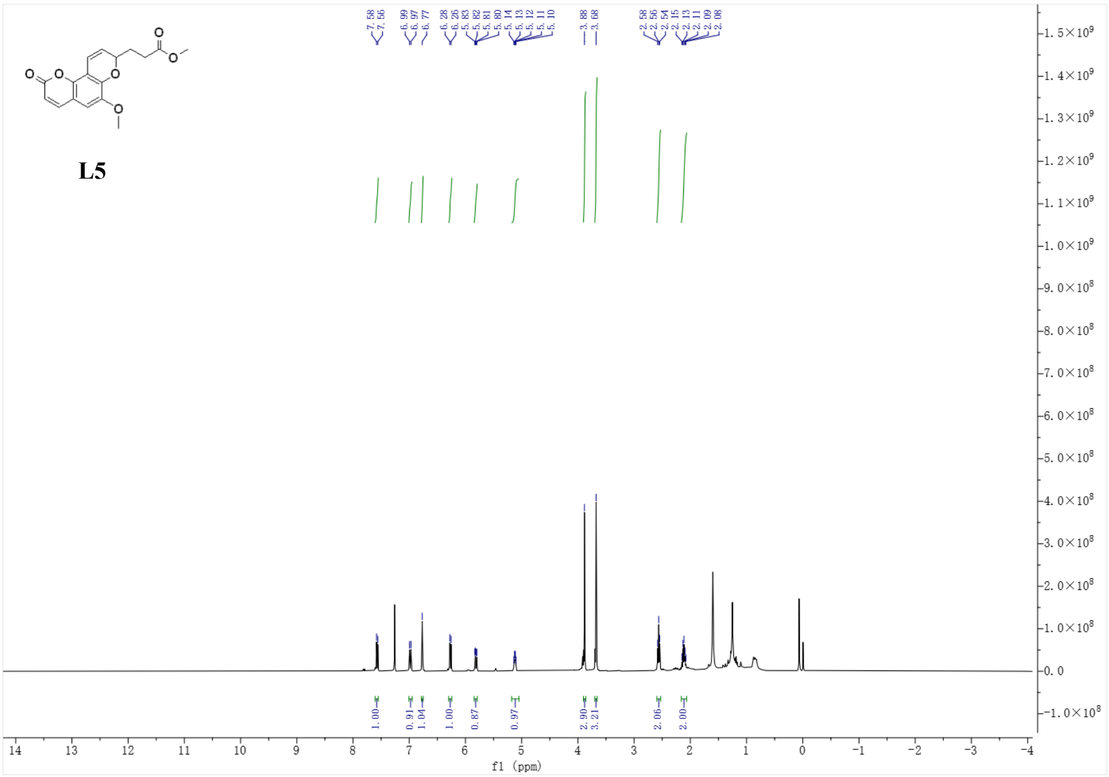

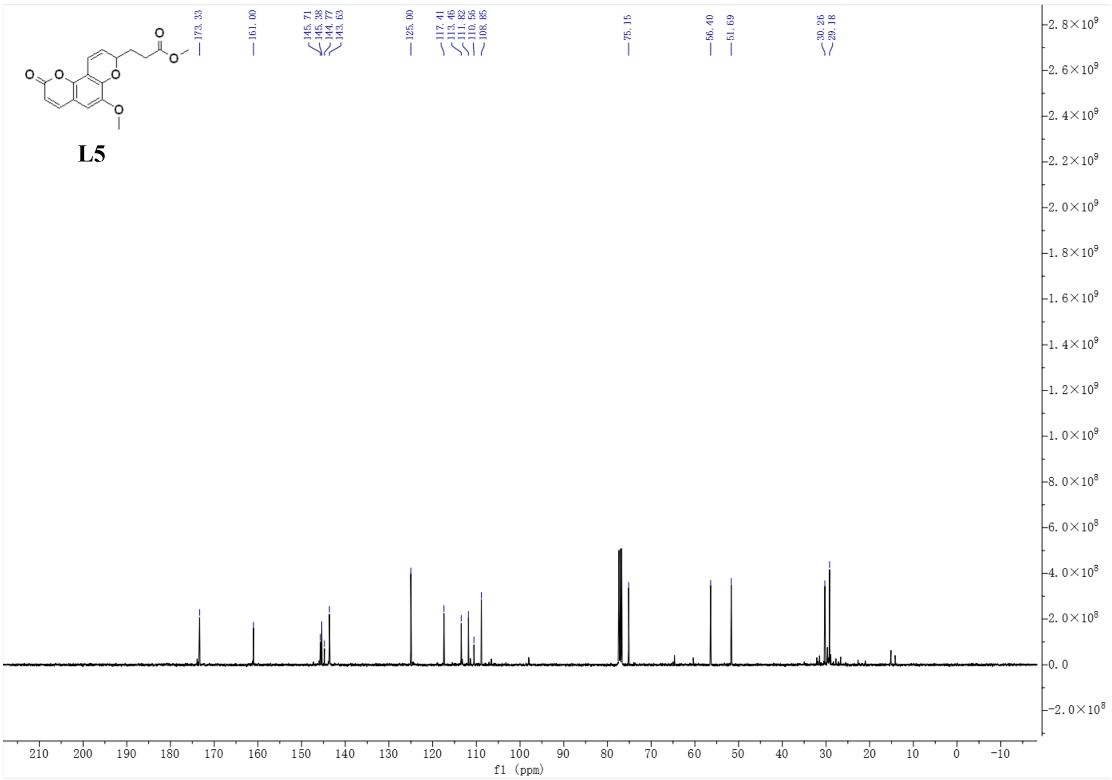

^{1}H NMR and ^{13}C NMR spectroscopic data of **L5**

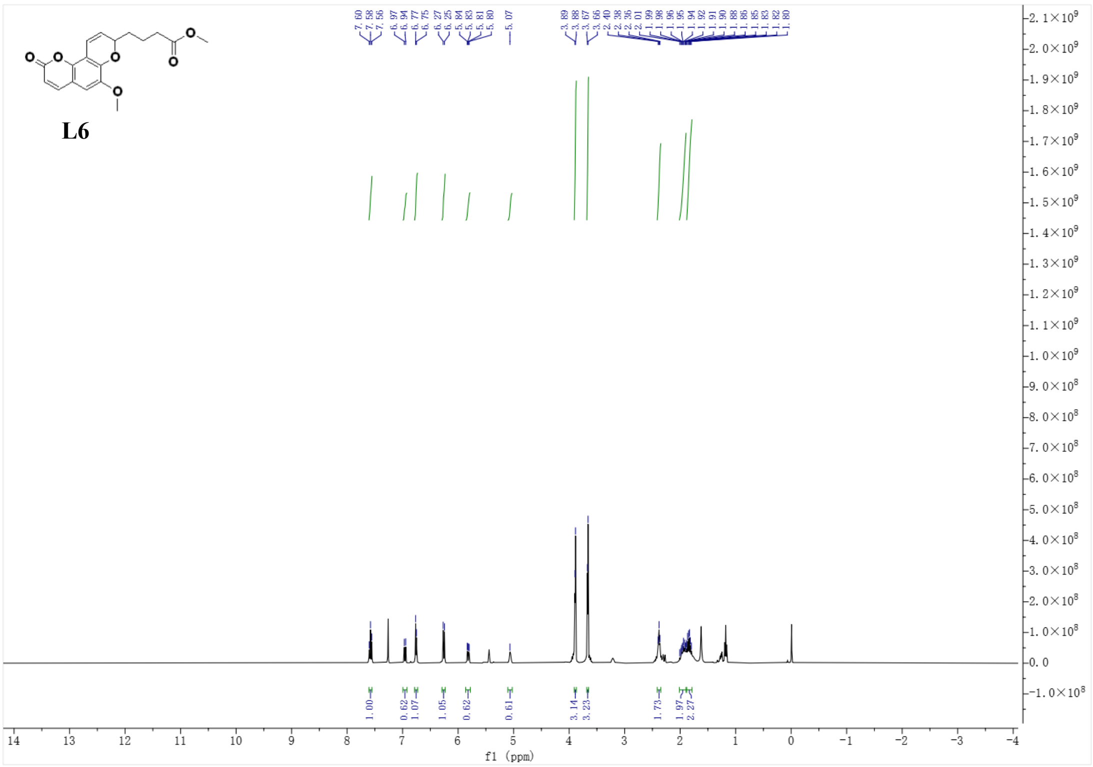

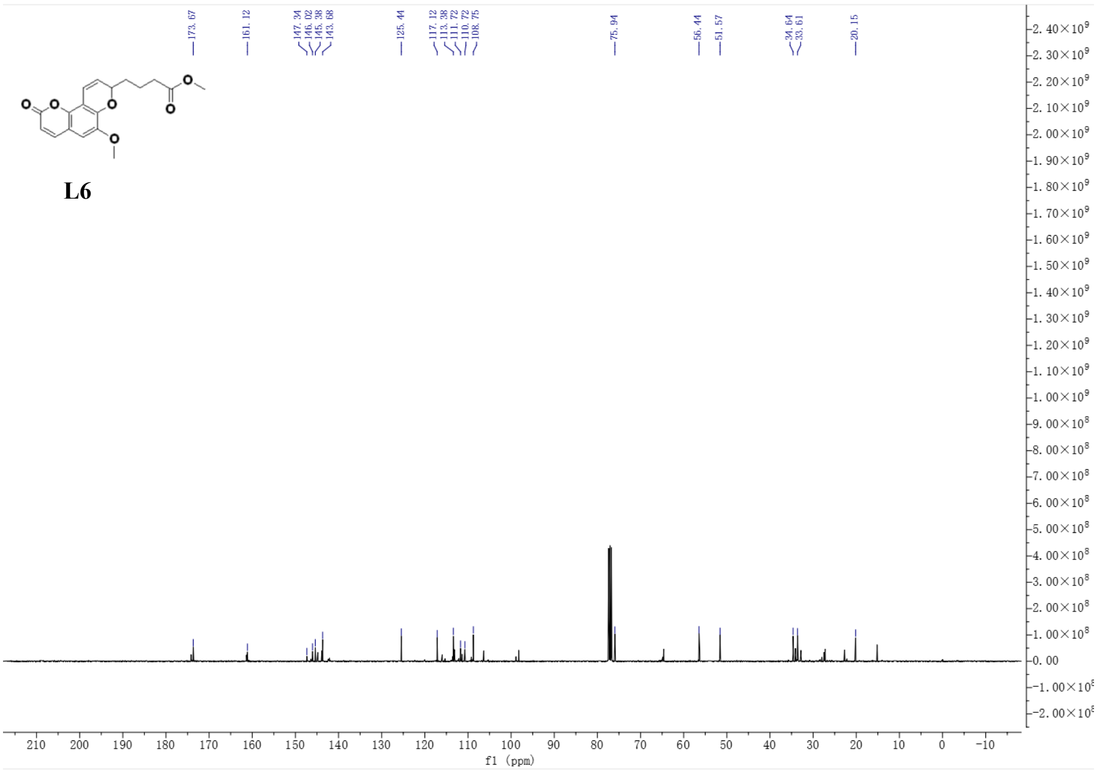

^{1}H NMR and ^{13}C NMR spectroscopic data of **L6**

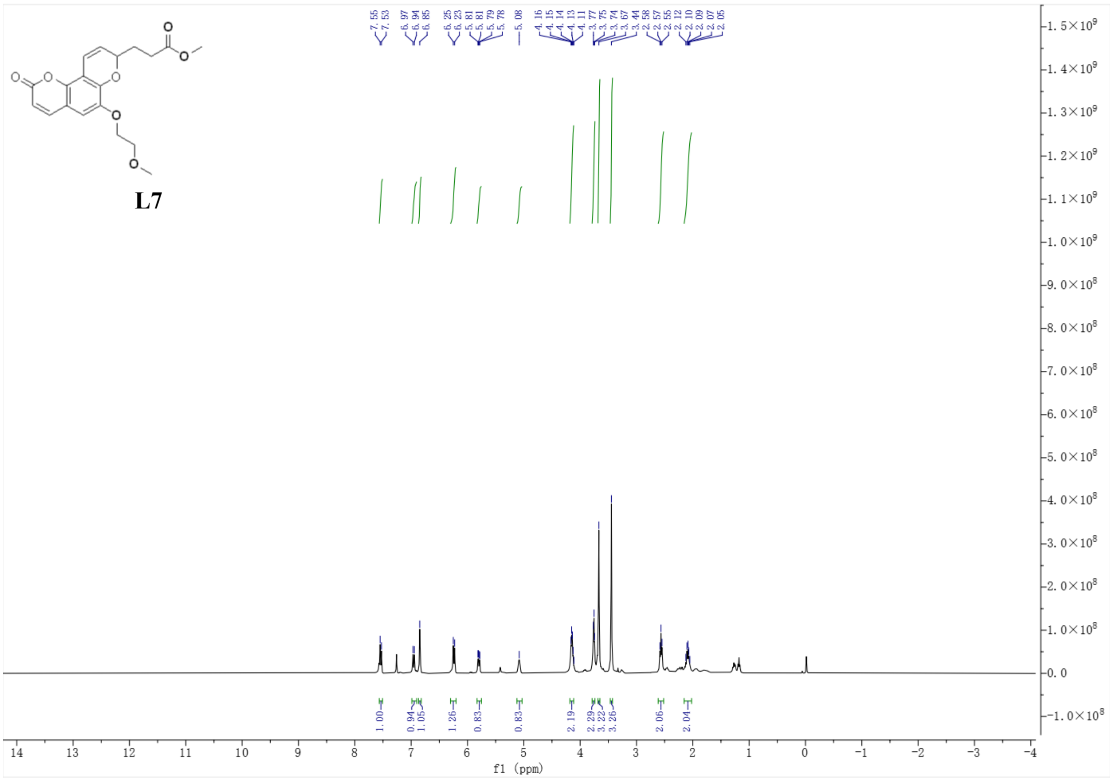

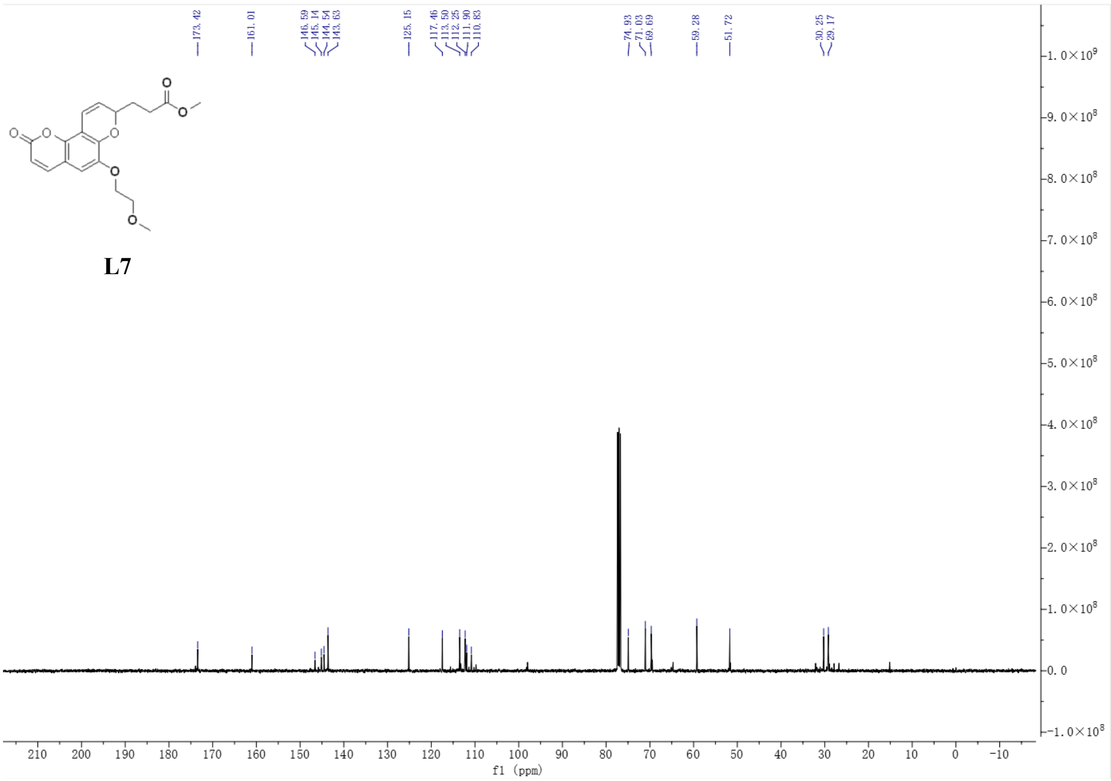

^{1}H NMR and ^{13}C NMR spectroscopic data of **L7**

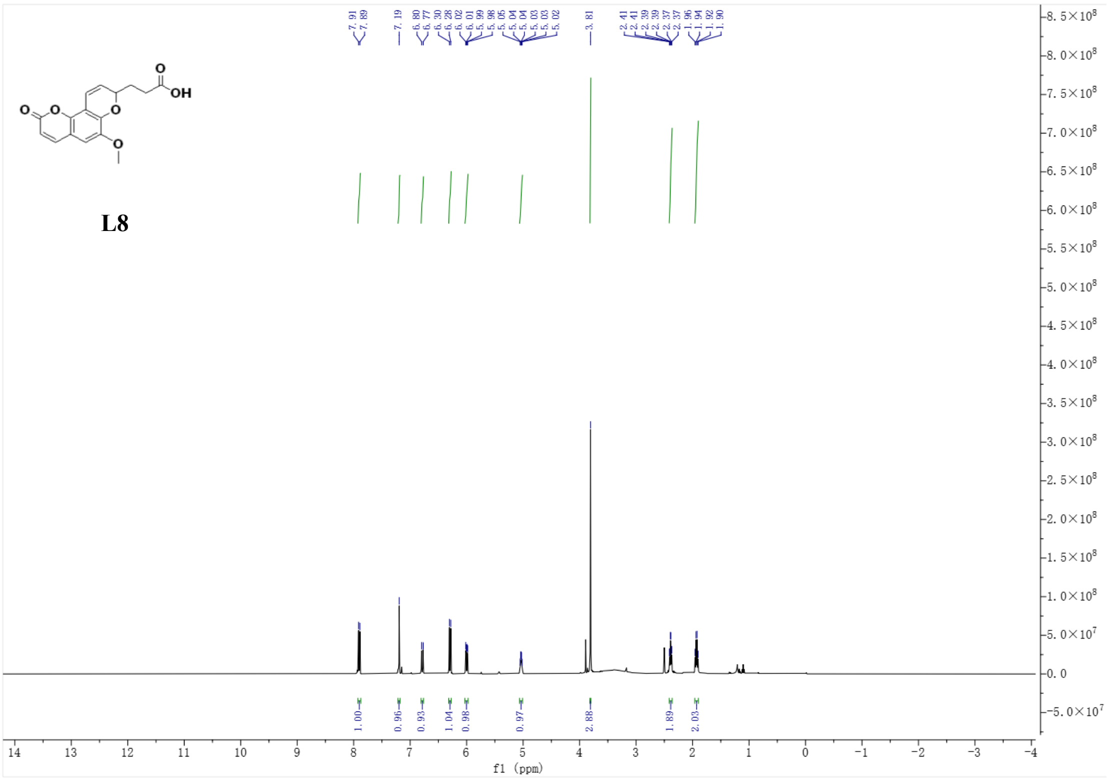

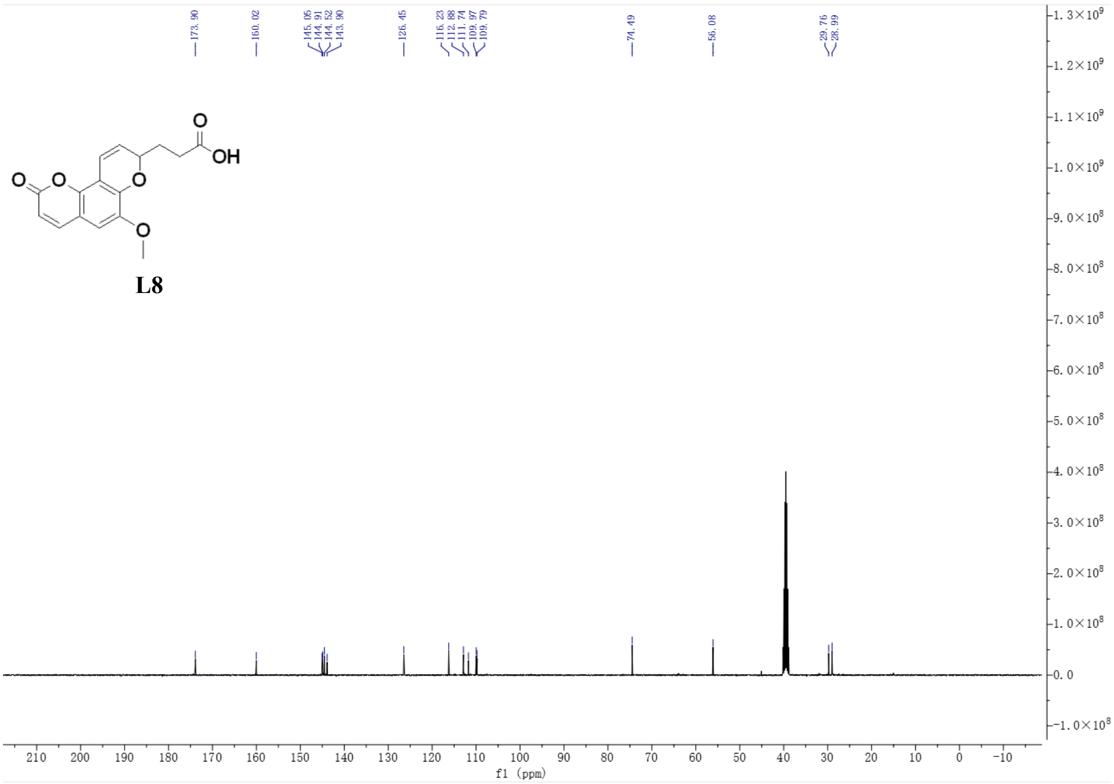

^{1}H NMR and ^{13}C NMR spectroscopic data of **L8**

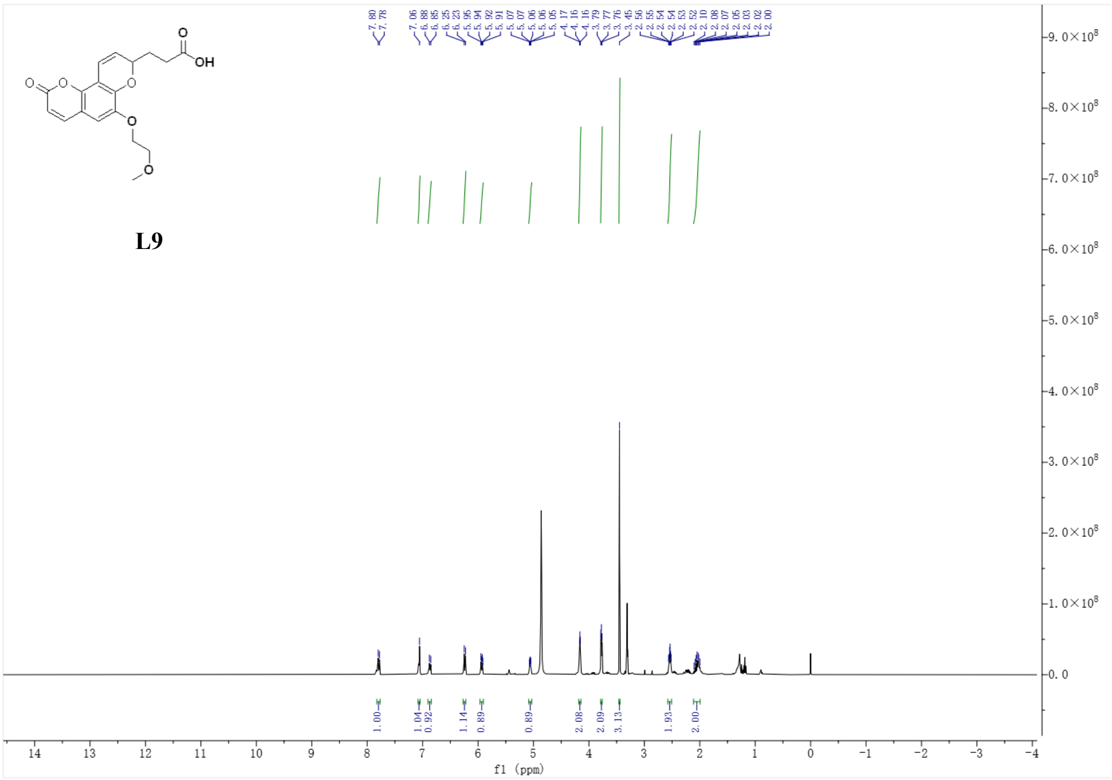

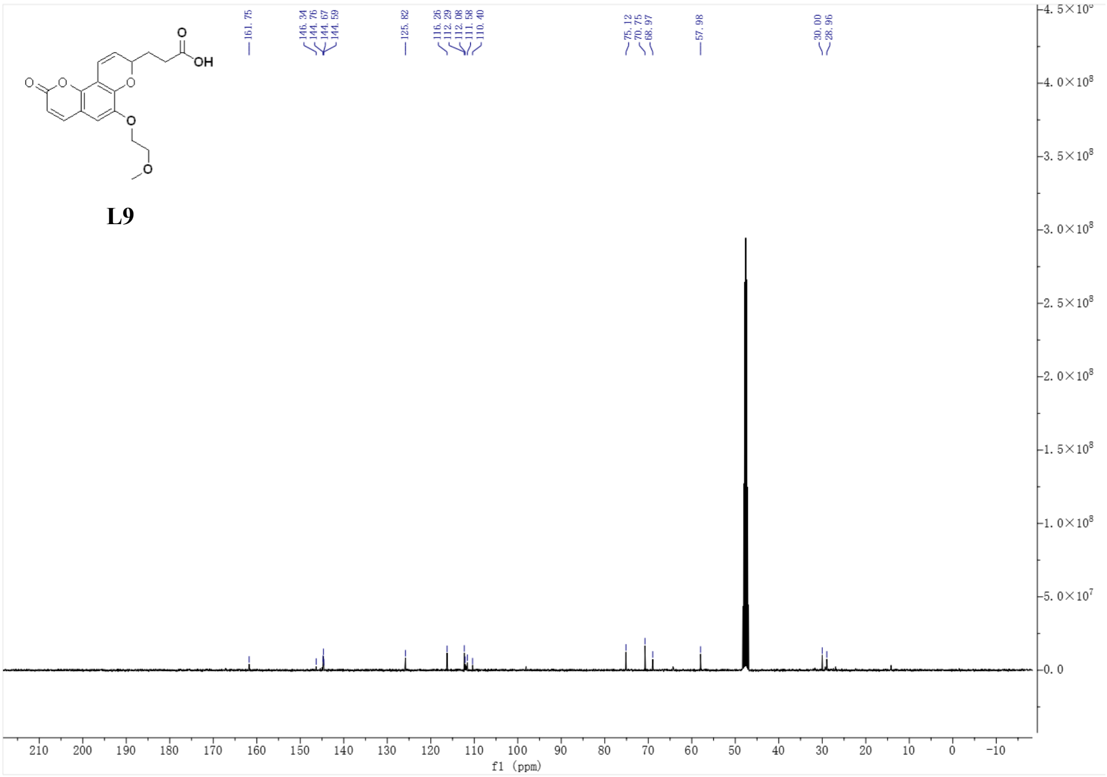

^{1}H NMR and ^{13}C NMR spectroscopic data of **L9**

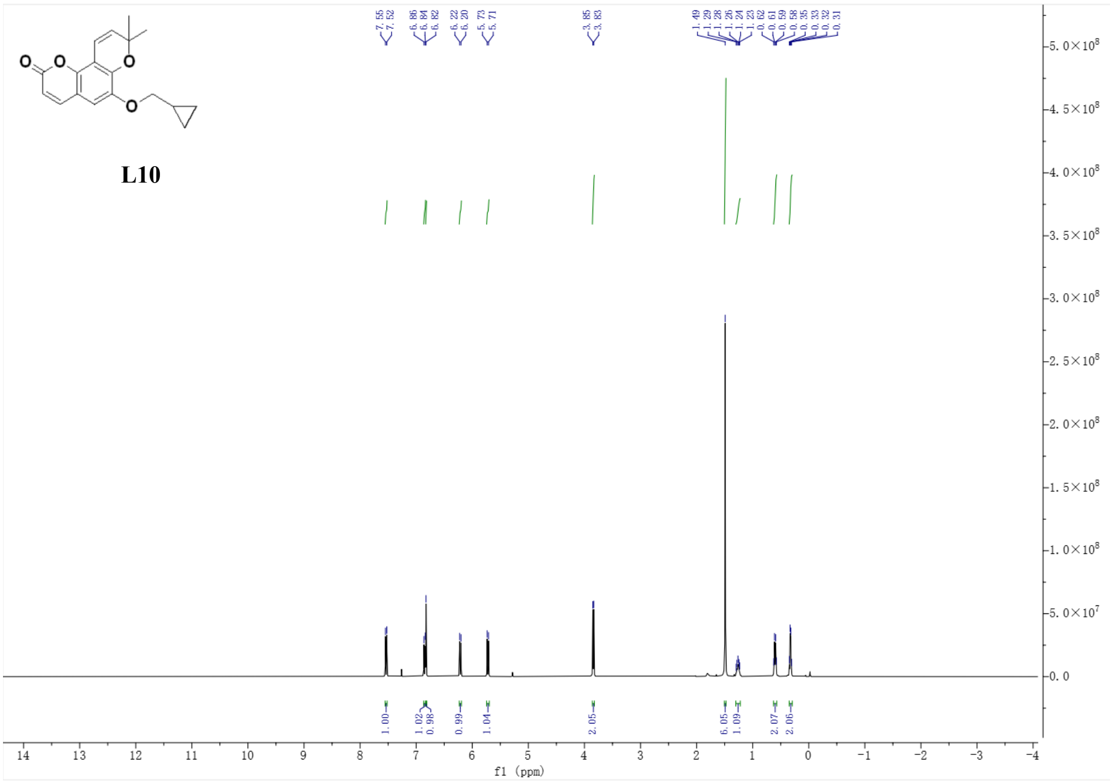

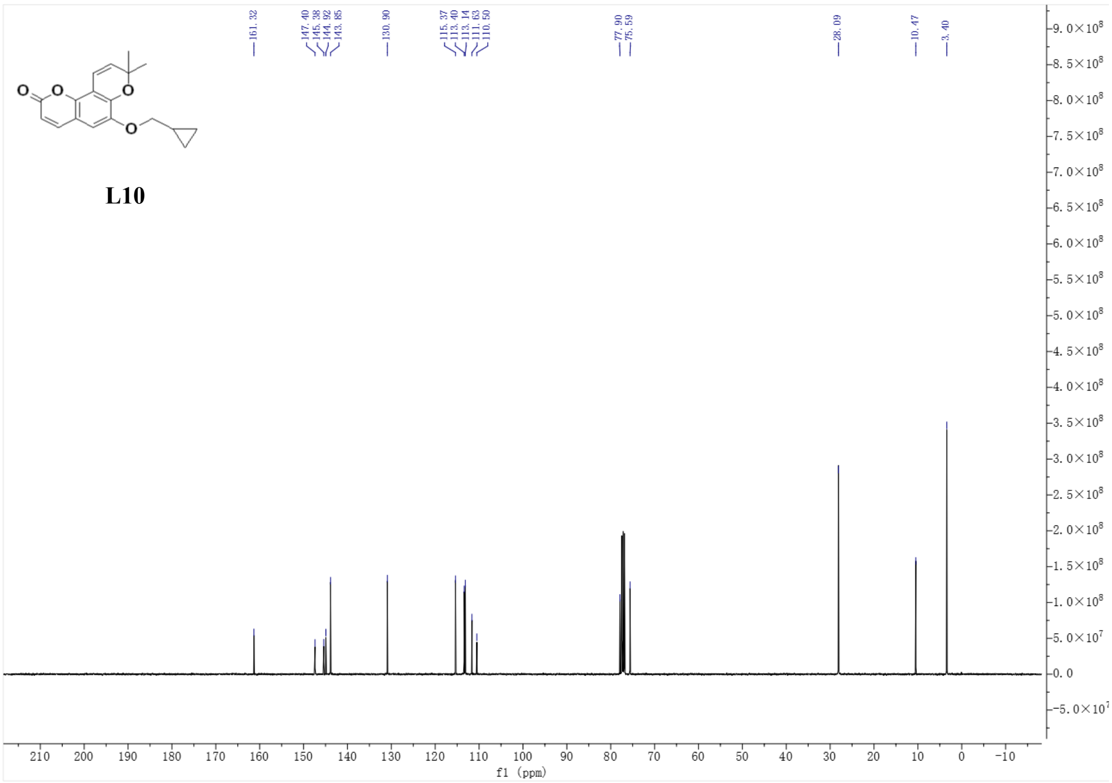

^{1}H NMR and ^{13}C NMR spectroscopic data of **L10**

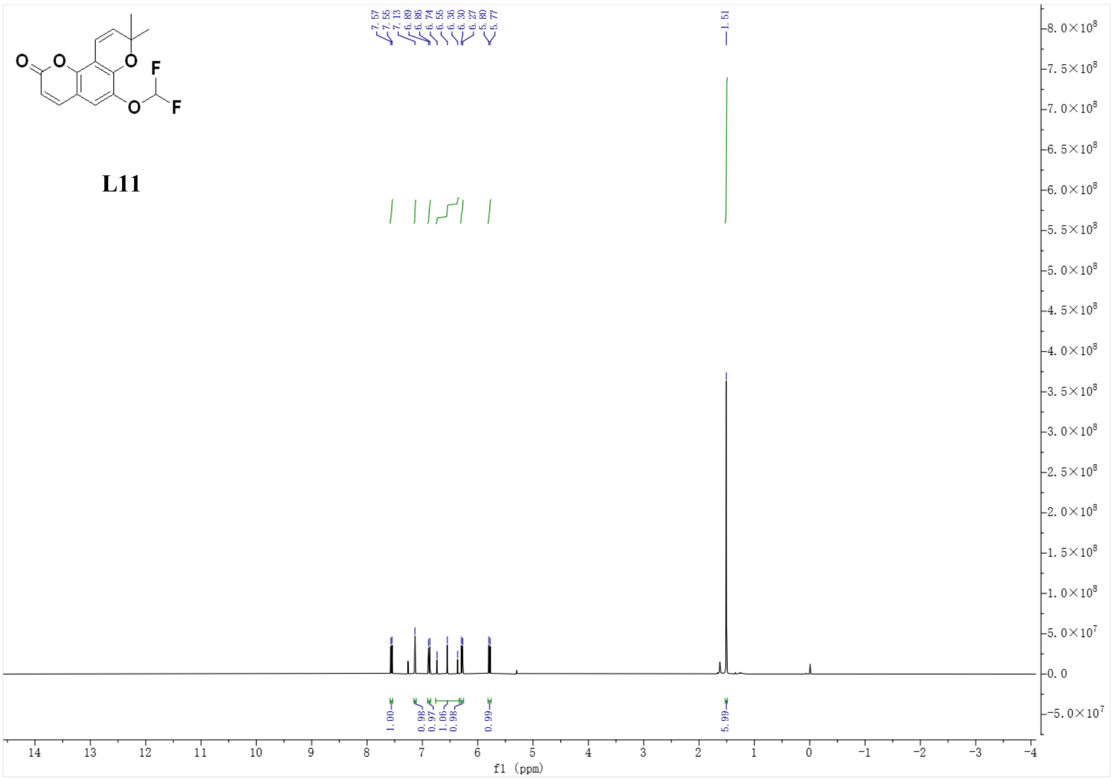

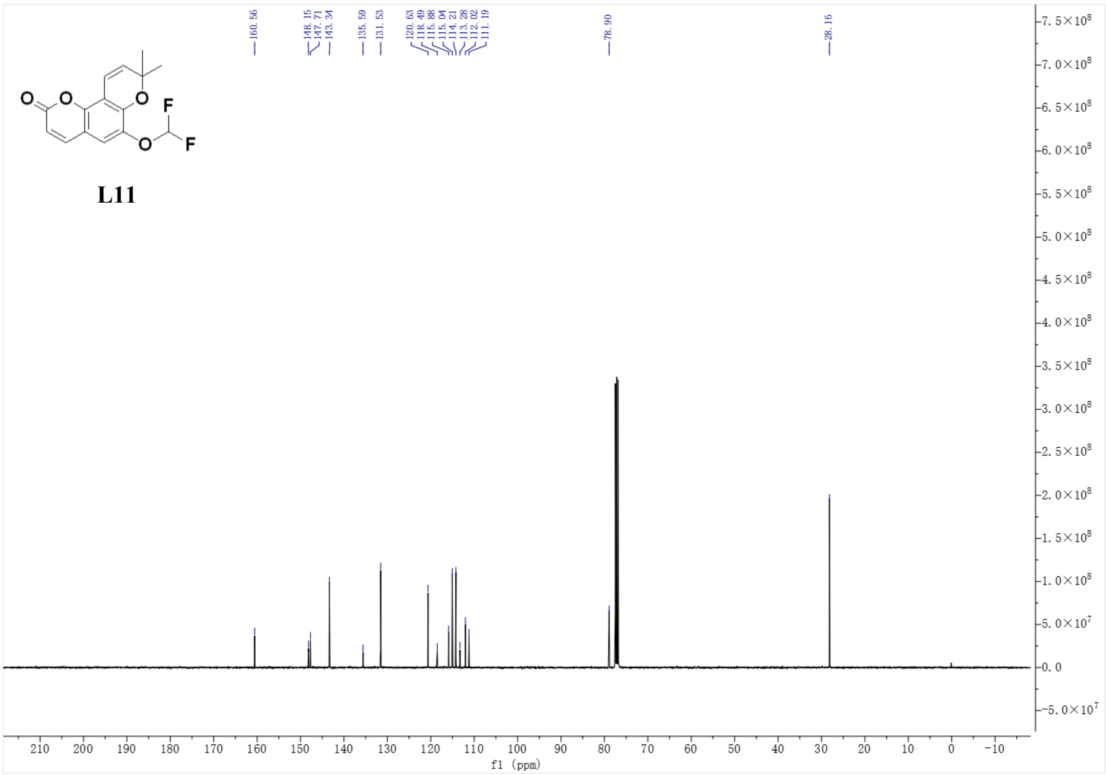

^{1}H NMR and ^{13}C NMR spectroscopic data of **L11**

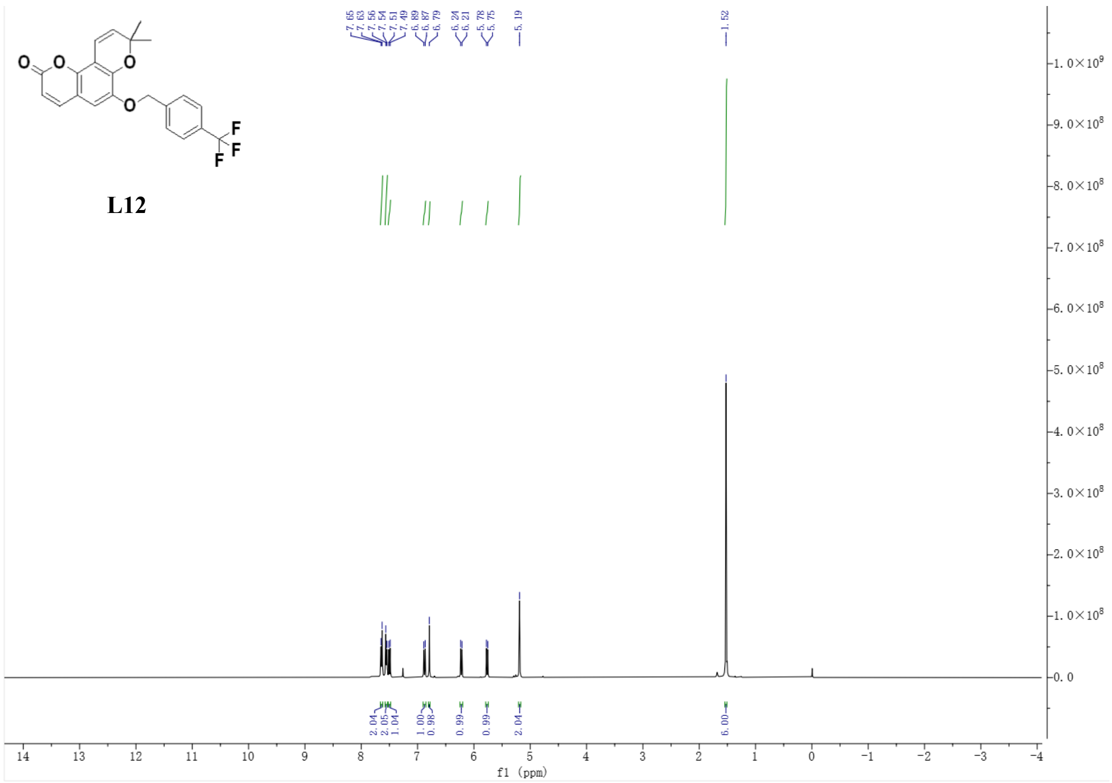

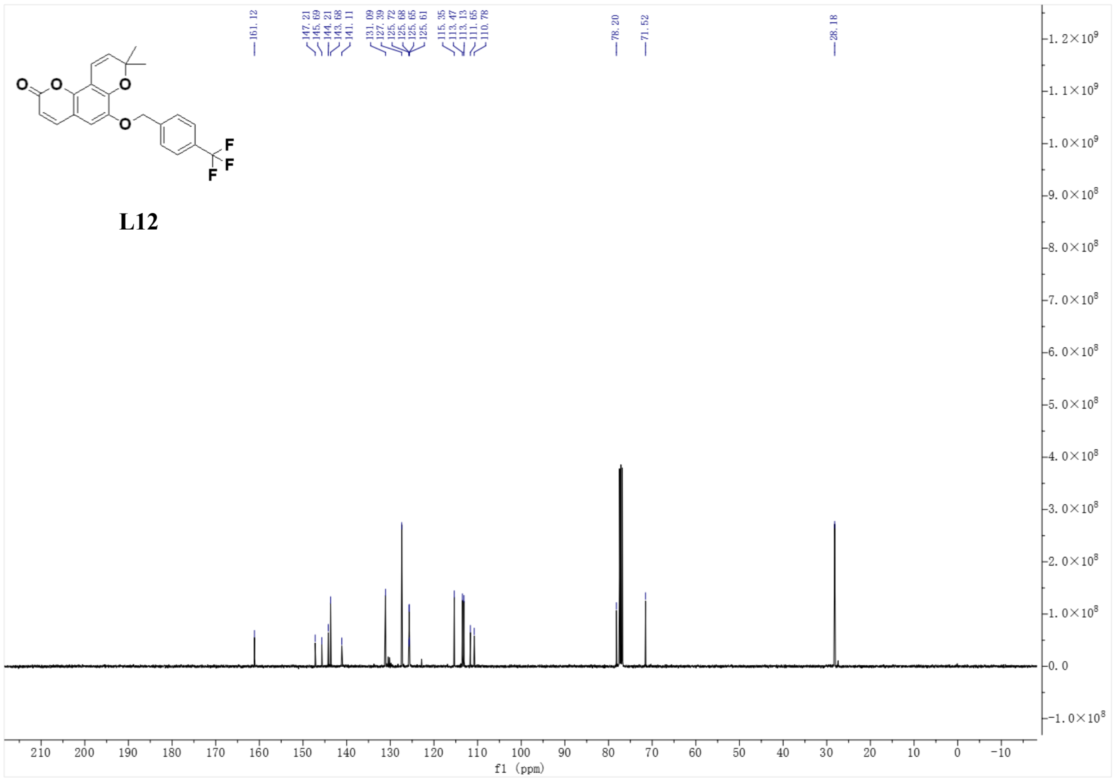

^{1}H NMR and ^{13}C NMR spectroscopic data of **L12**

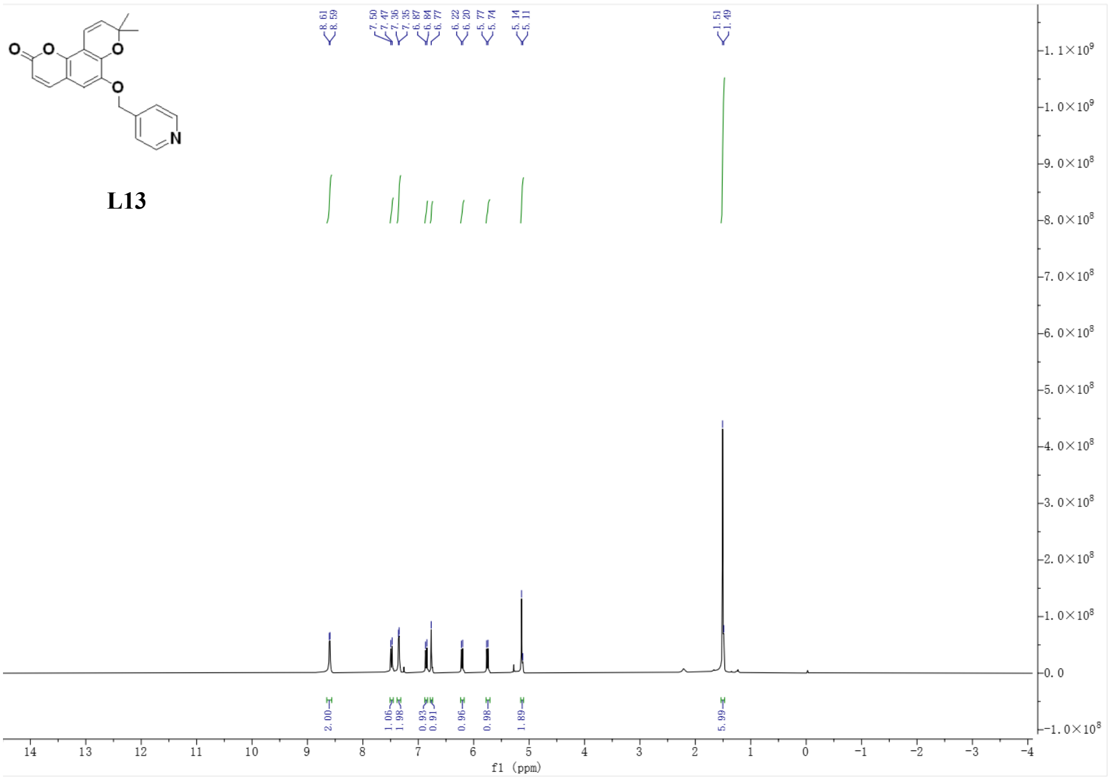

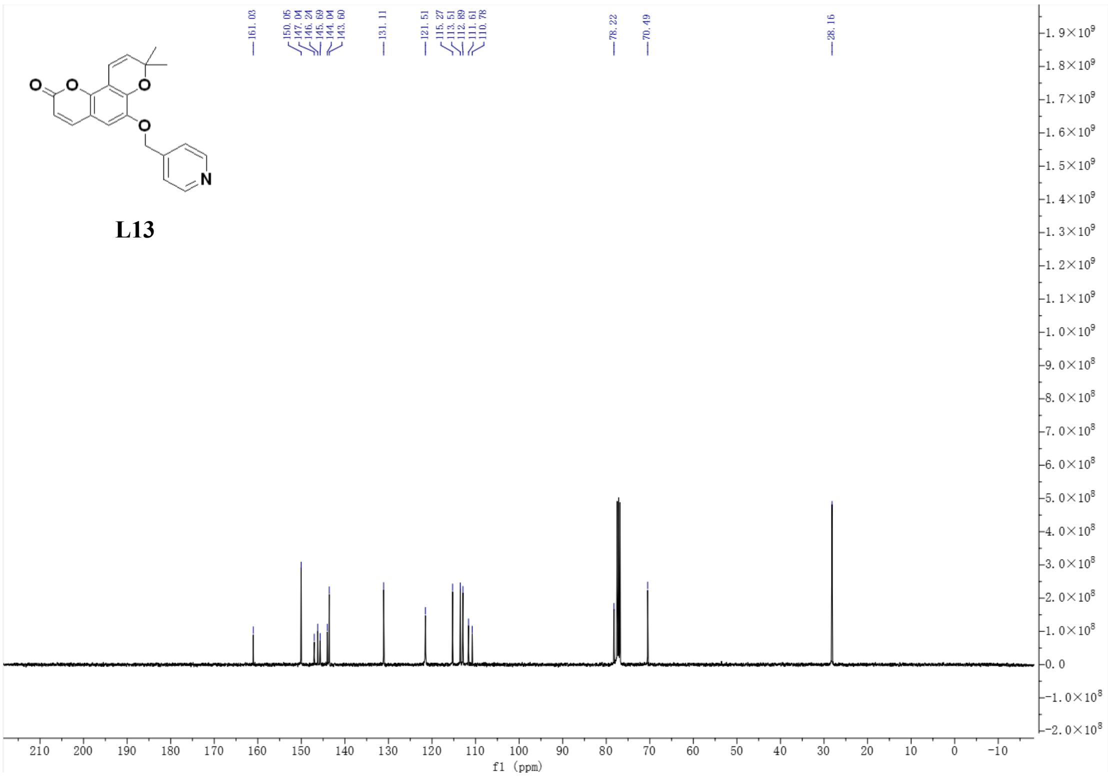

^{1}H NMR and ^{13}C NMR spectroscopic data of **L13**

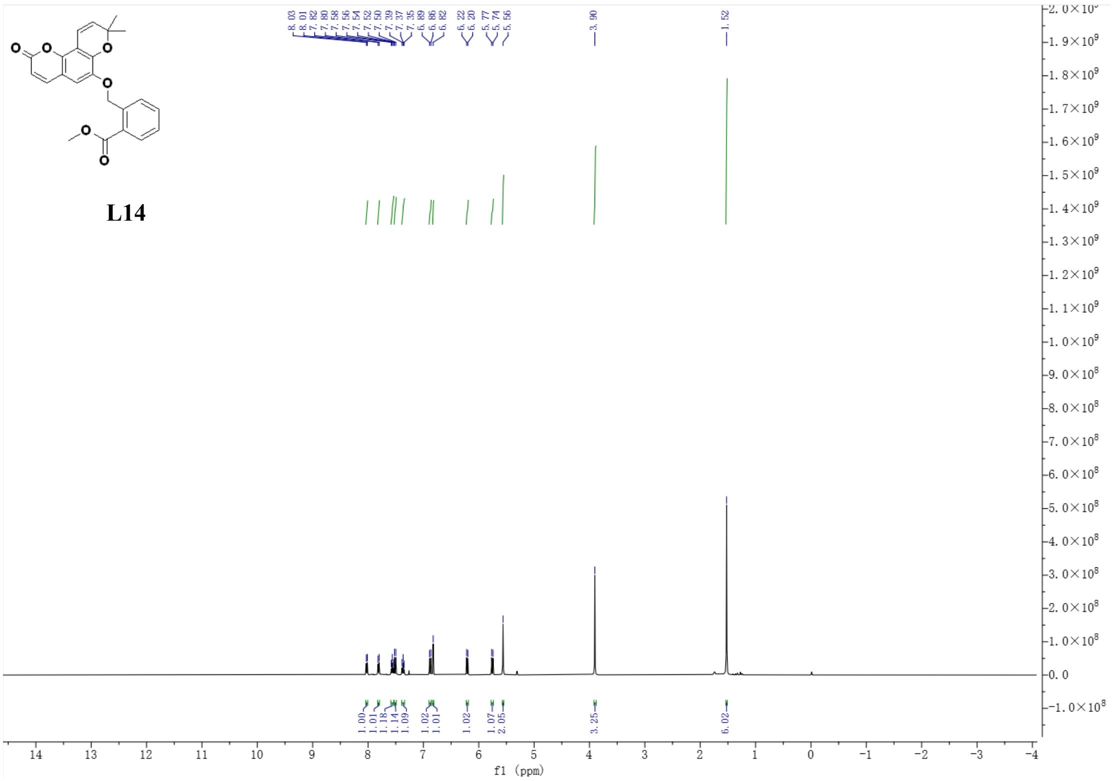

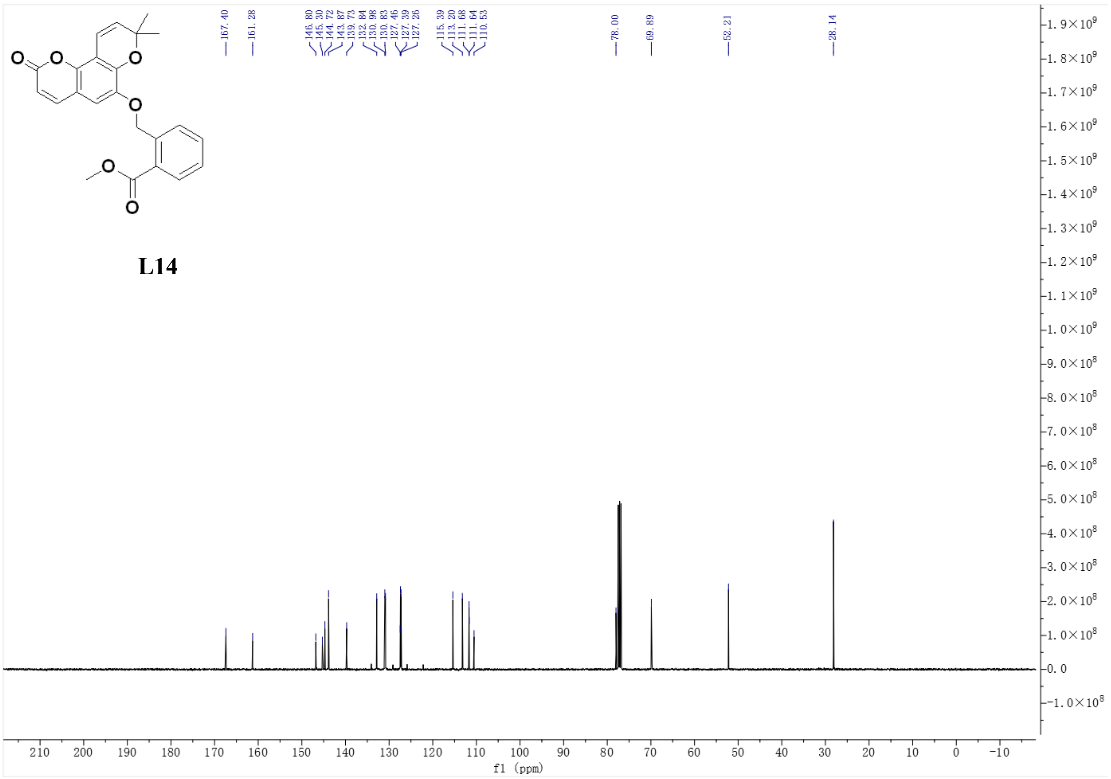

^{1}H NMR and ^{13}C NMR spectroscopic data of **L14**

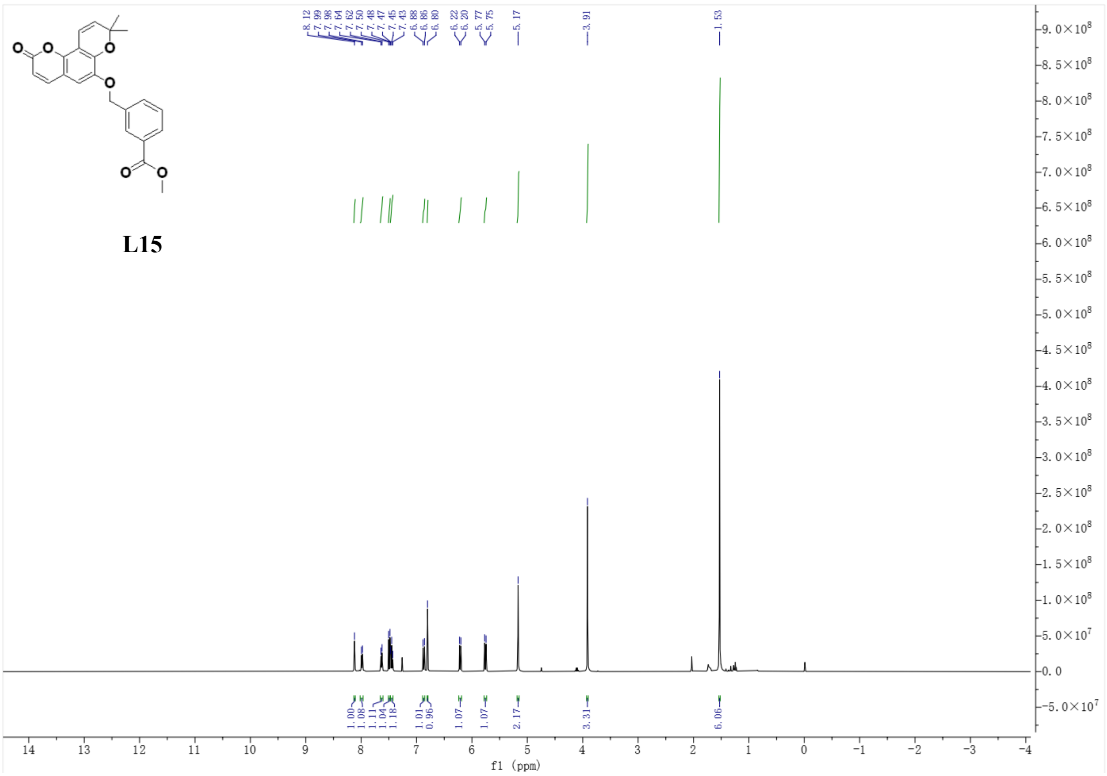

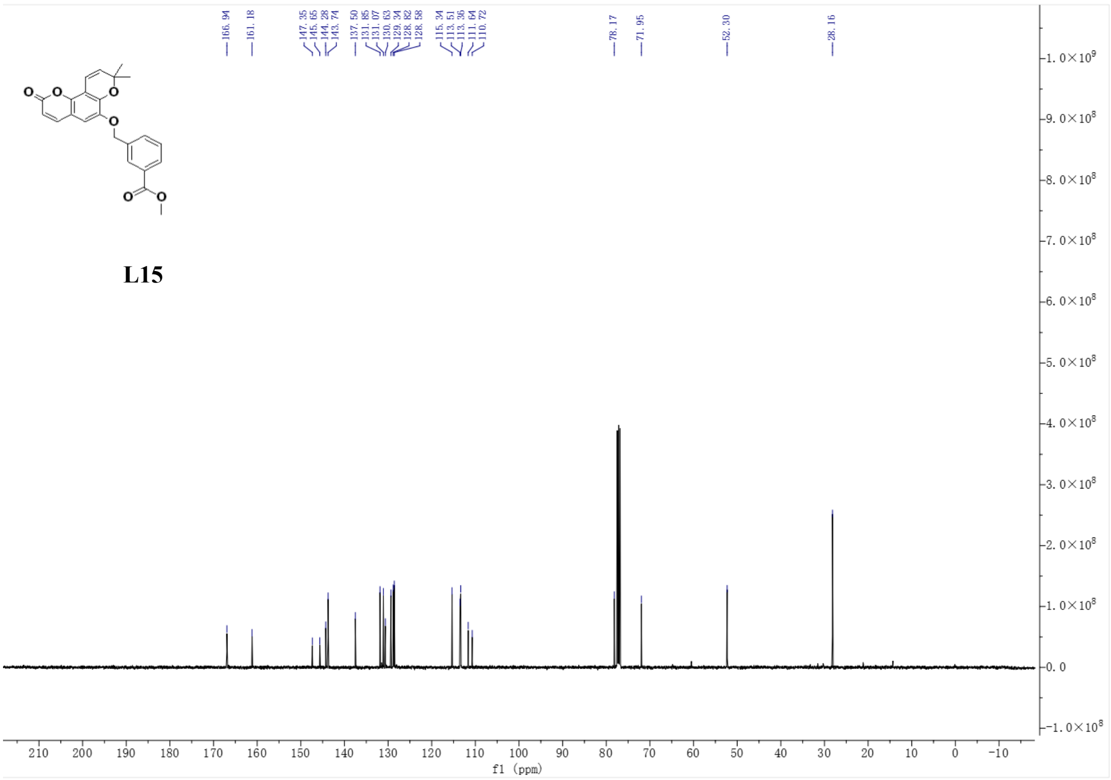

^{1}H NMR and ^{13}C NMR spectroscopic data of **L15**

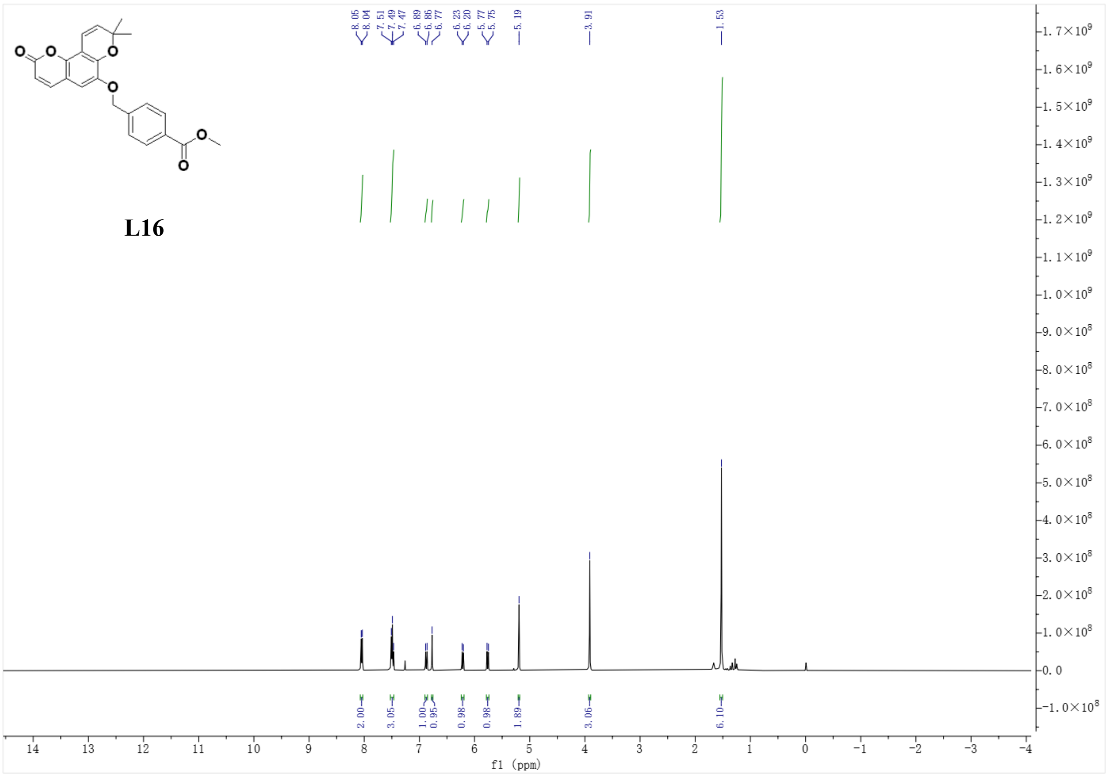

L16

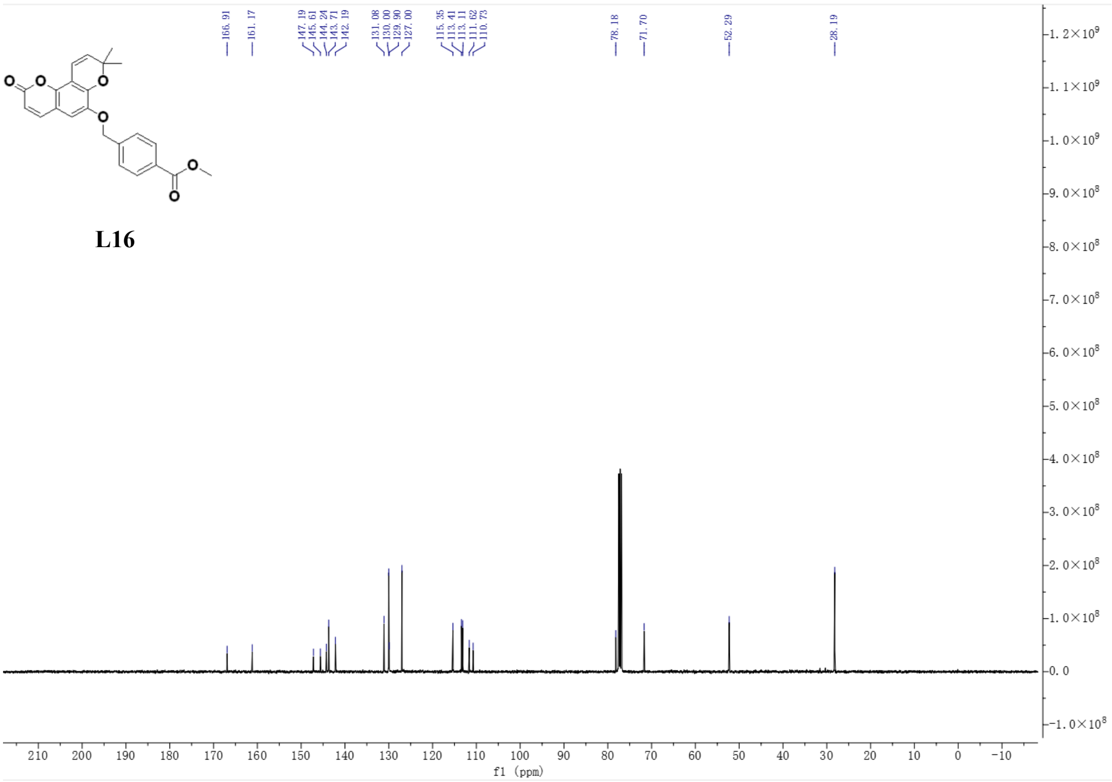

L16

^{1}H NMR and ^{13}C NMR spectroscopic data of **L16**

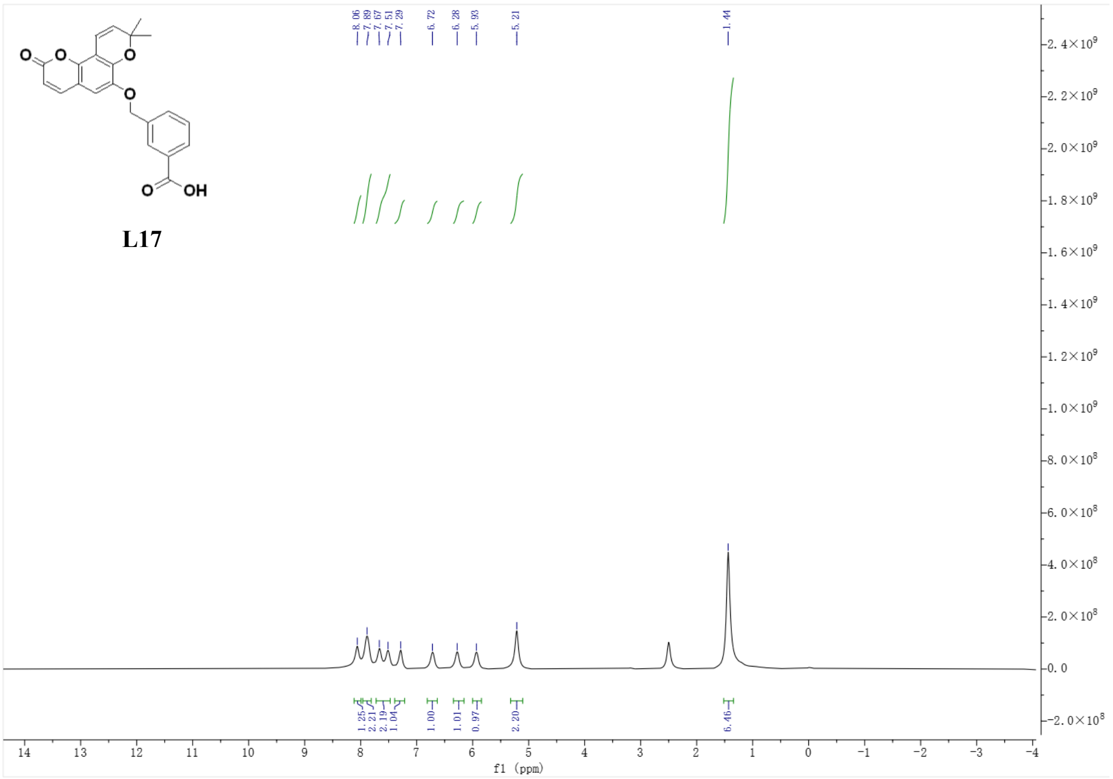

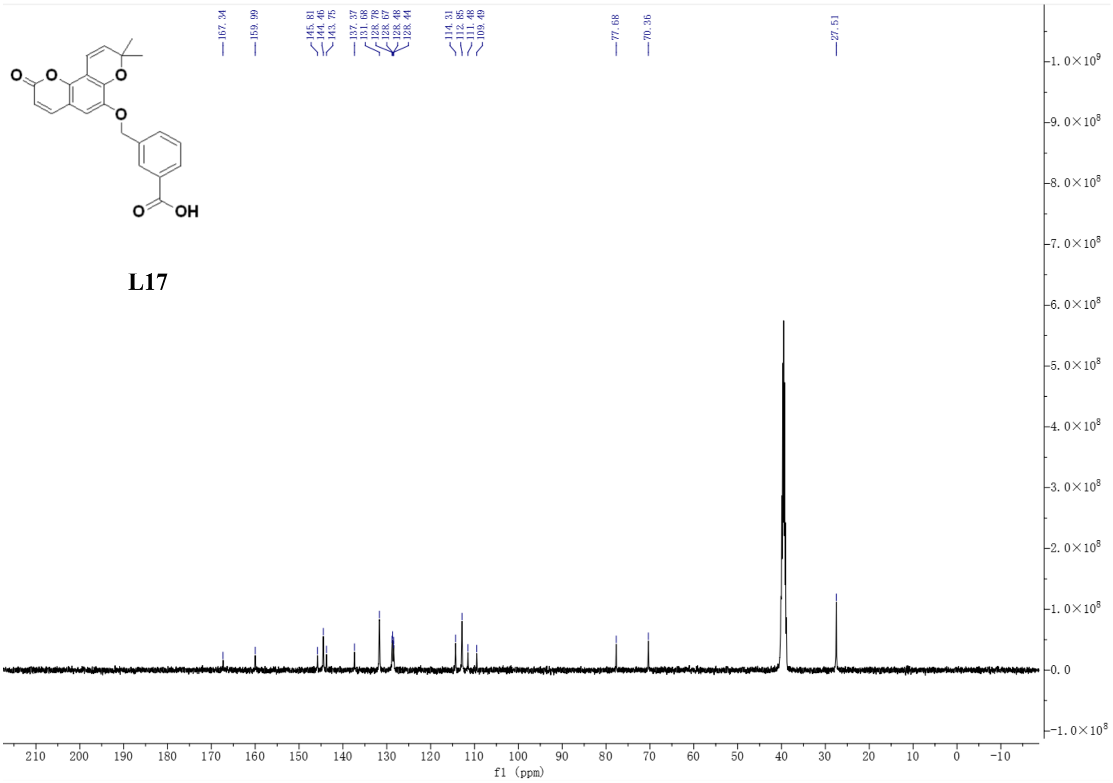

^{1}H NMR and ^{13}C NMR spectroscopic data of **L17**

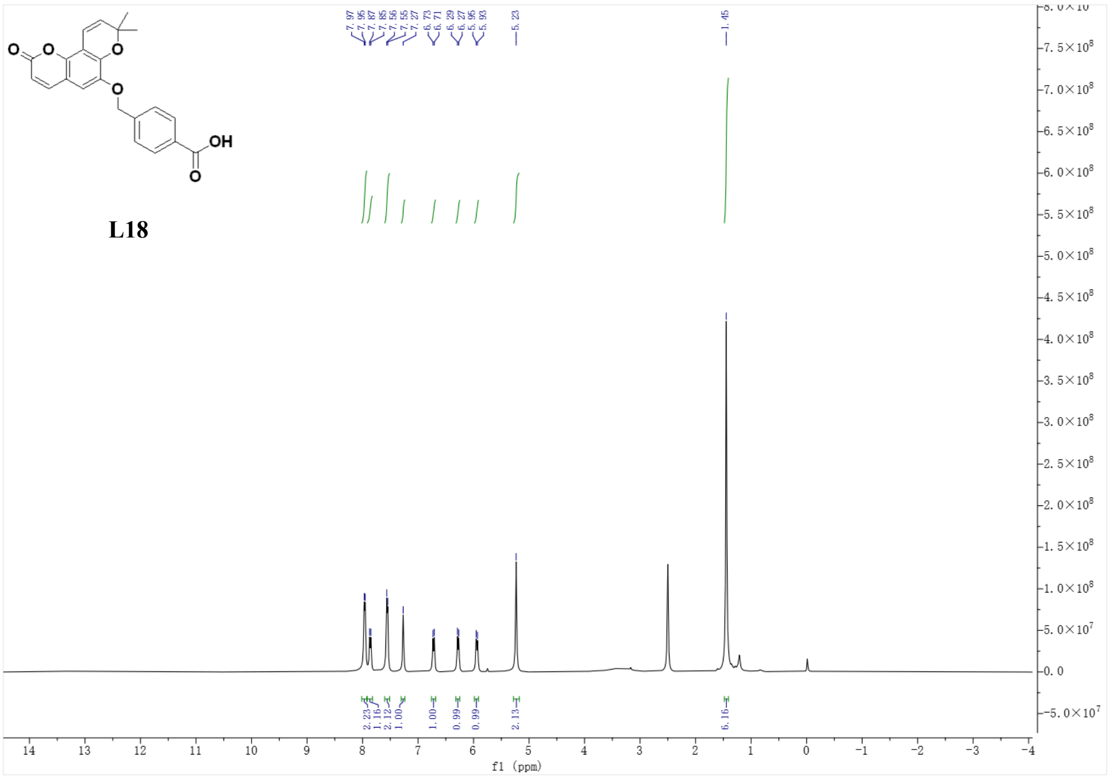

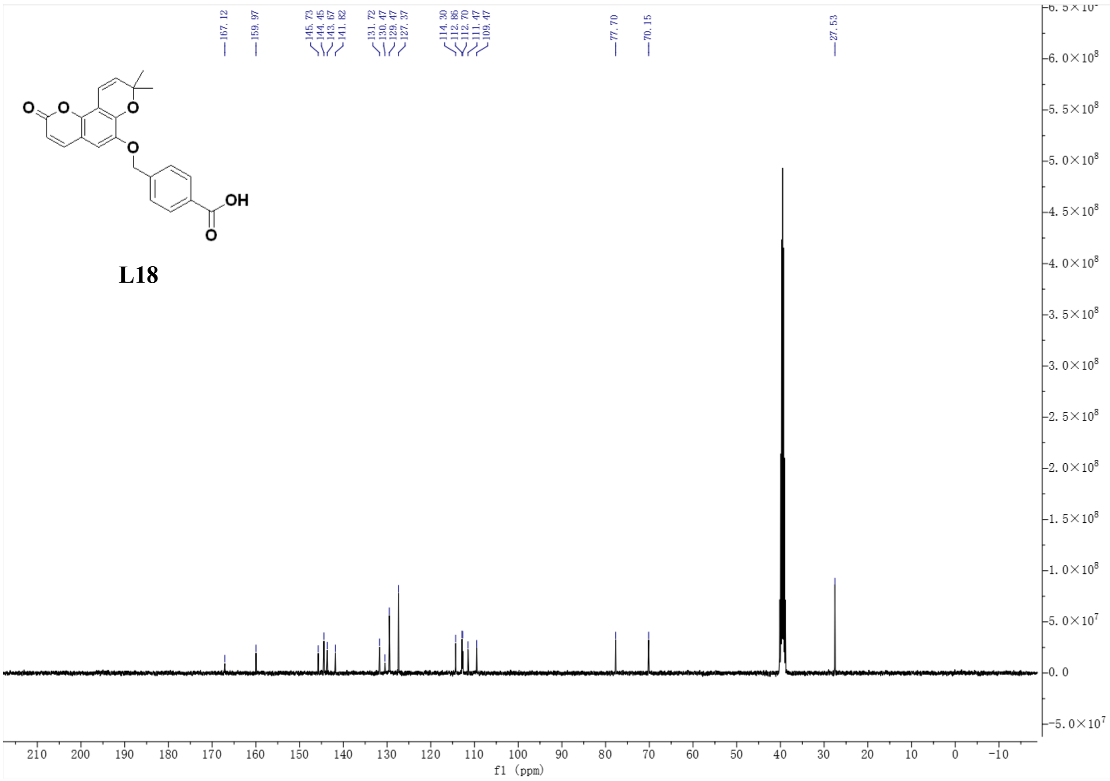

^{1}H NMR and ^{13}C NMR spectroscopic data of **L18**

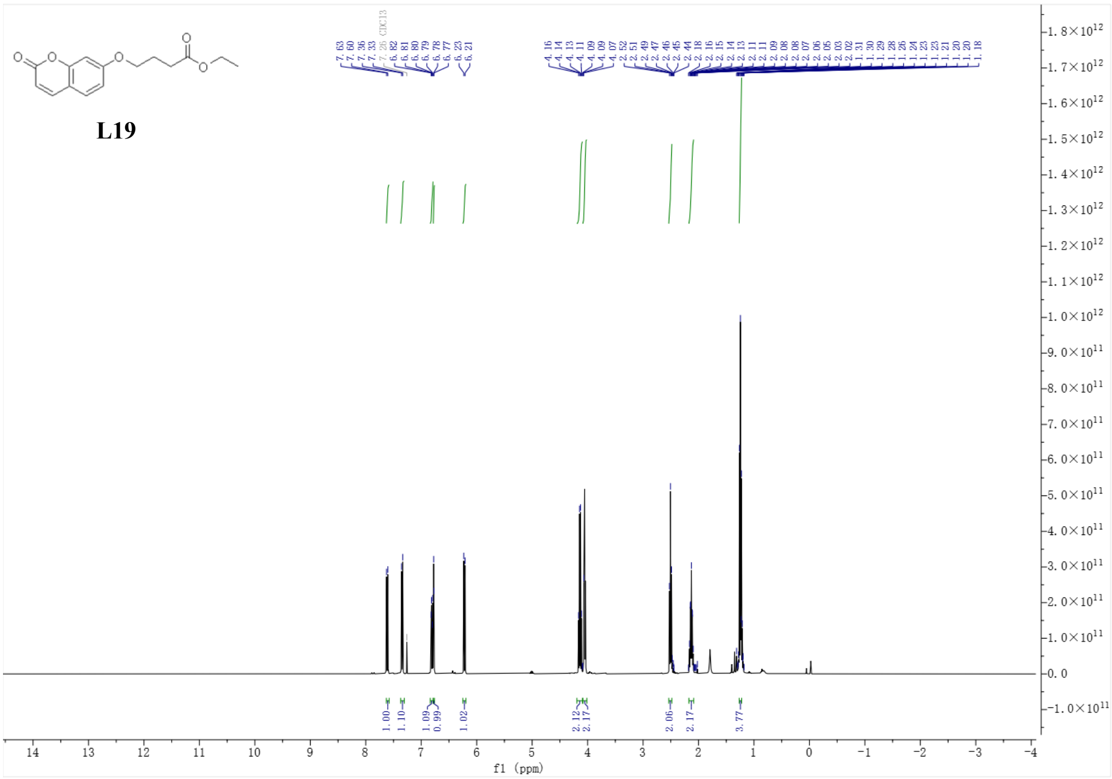

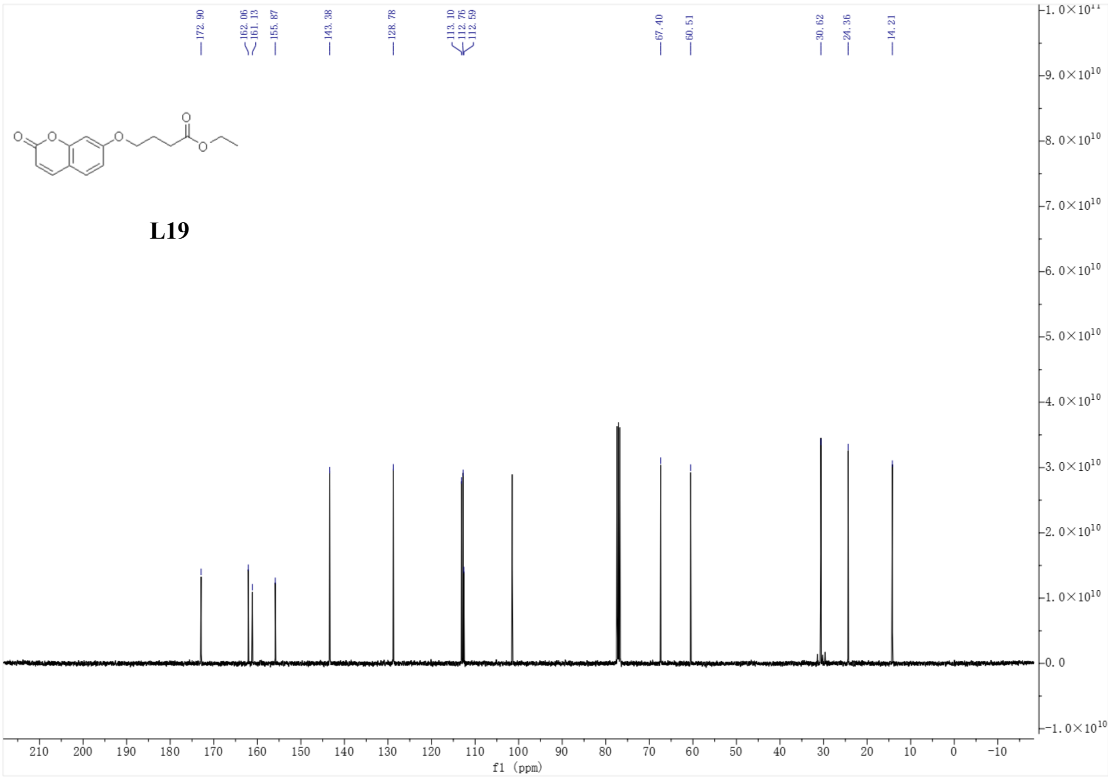

^{1}H NMR and ^{13}C NMR spectroscopic data of **L19**

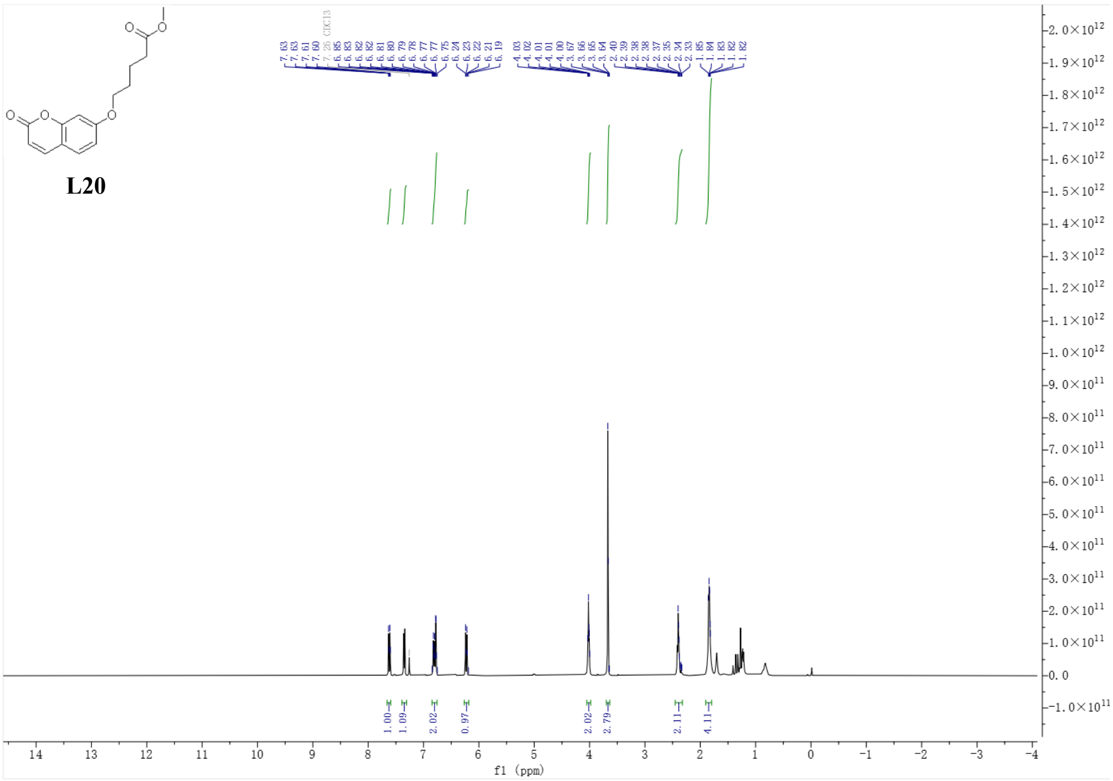

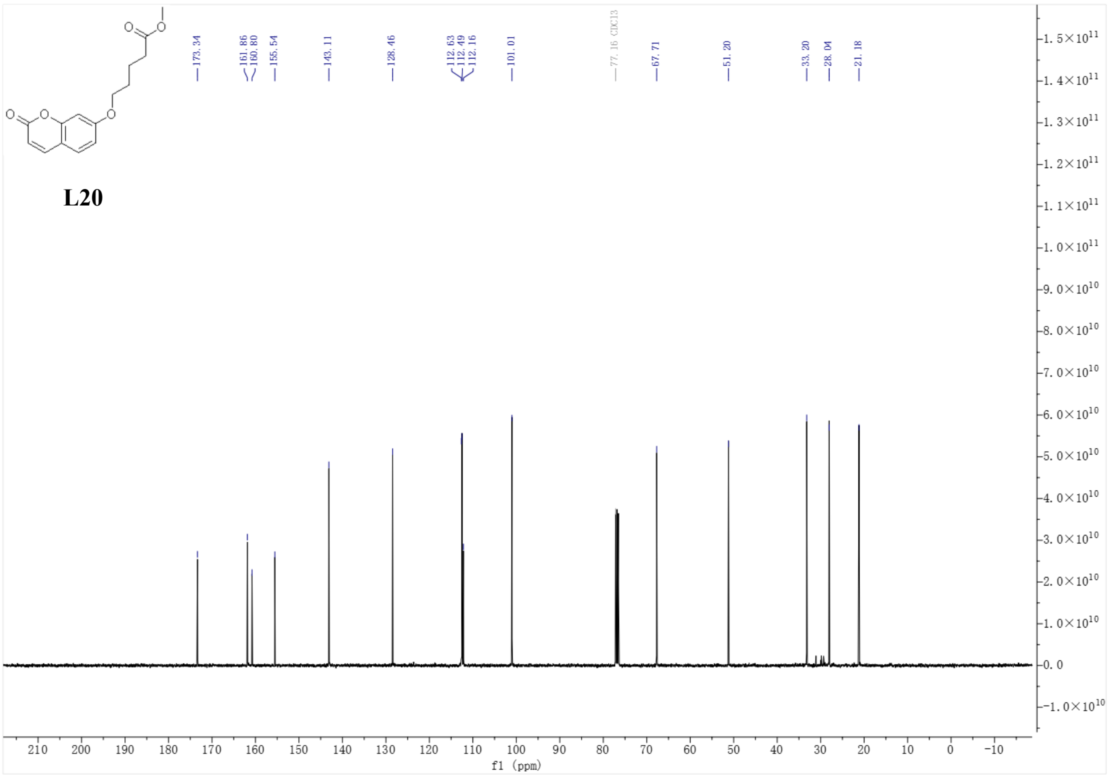

^{1}H NMR and ^{13}C NMR spectroscopic data of **L20**

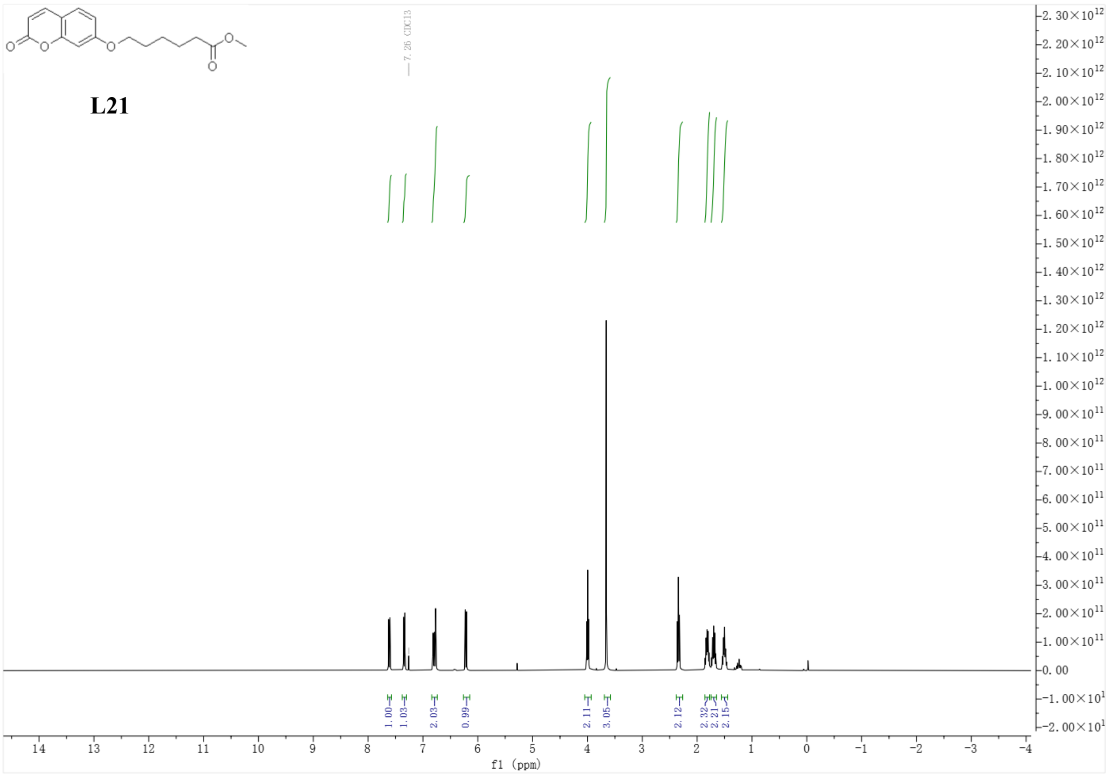

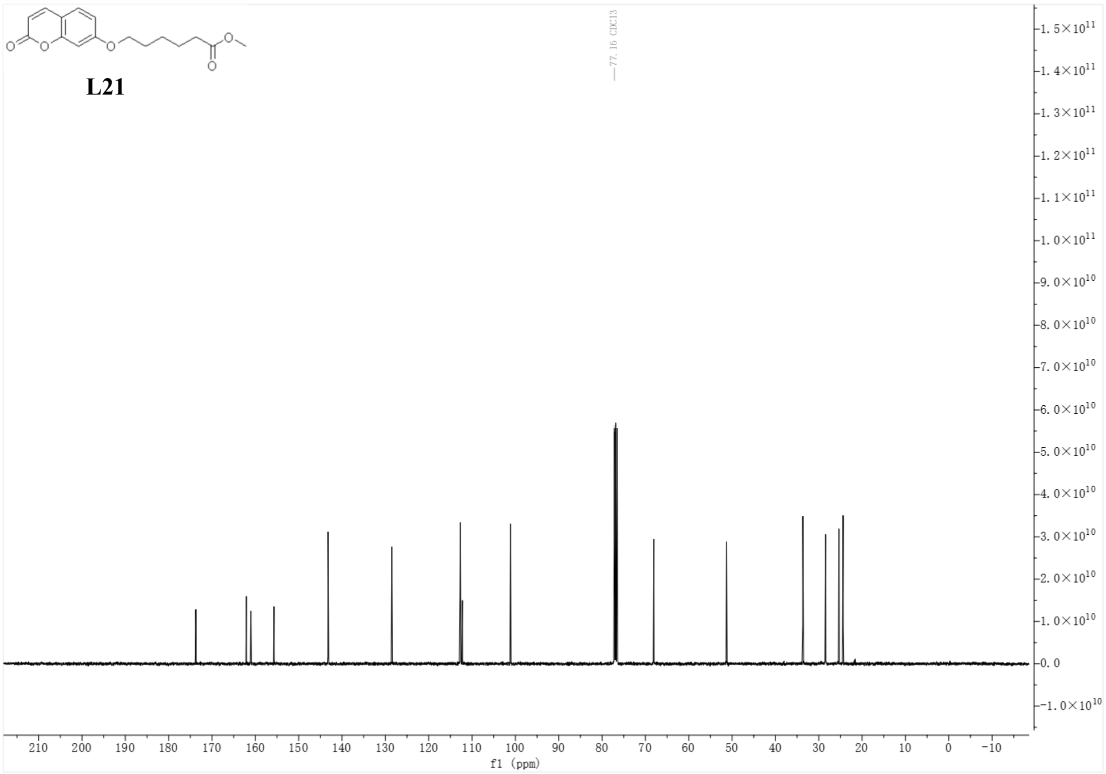

^{1}H NMR and ^{13}C NMR spectroscopic data of **L21**

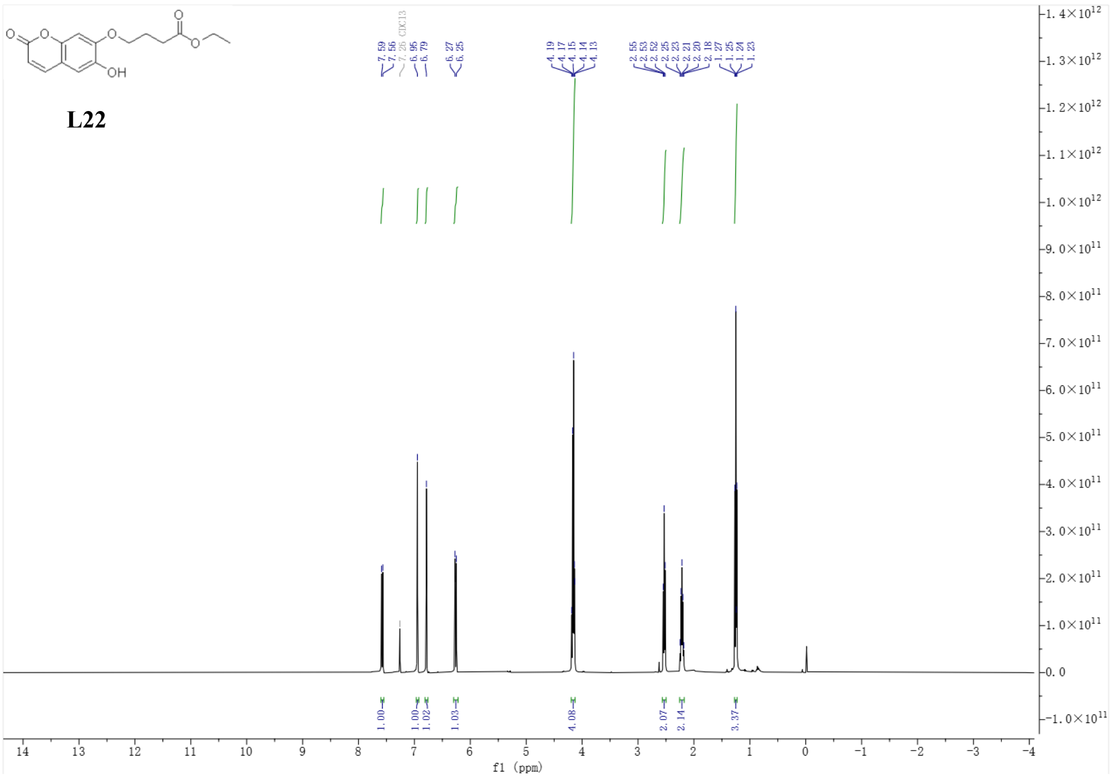

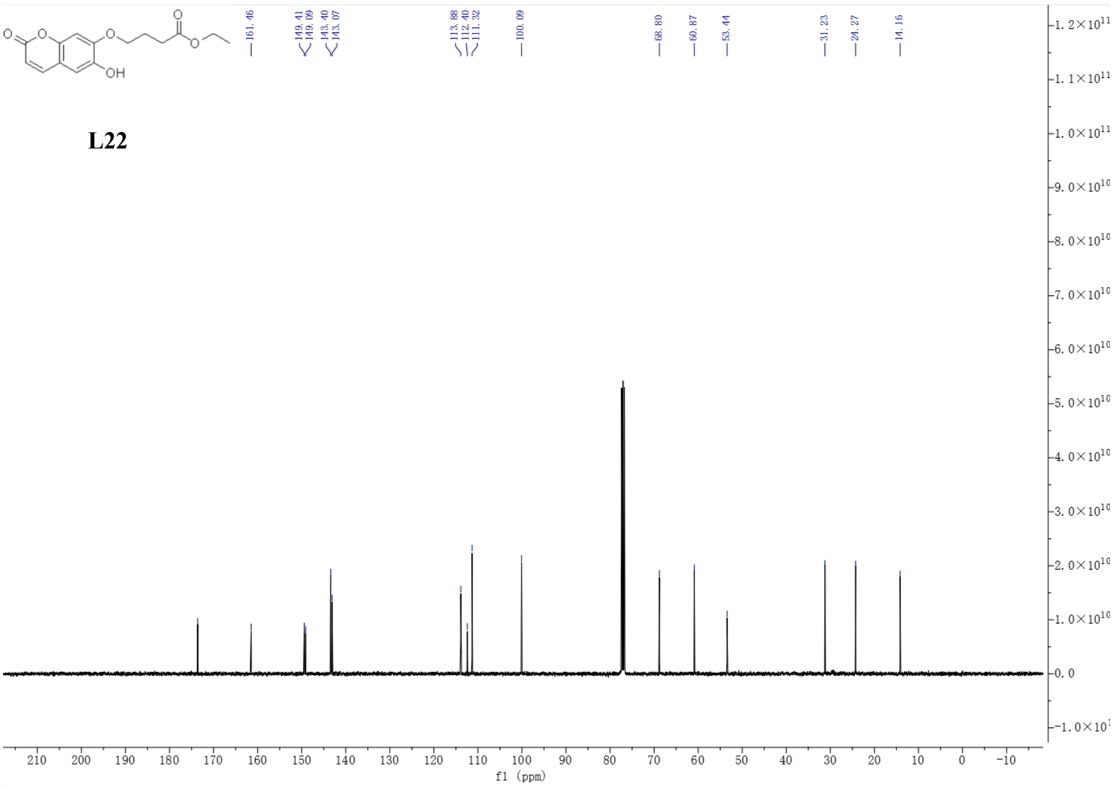

^{1}H NMR and ^{13}C NMR spectroscopic data of **L22**

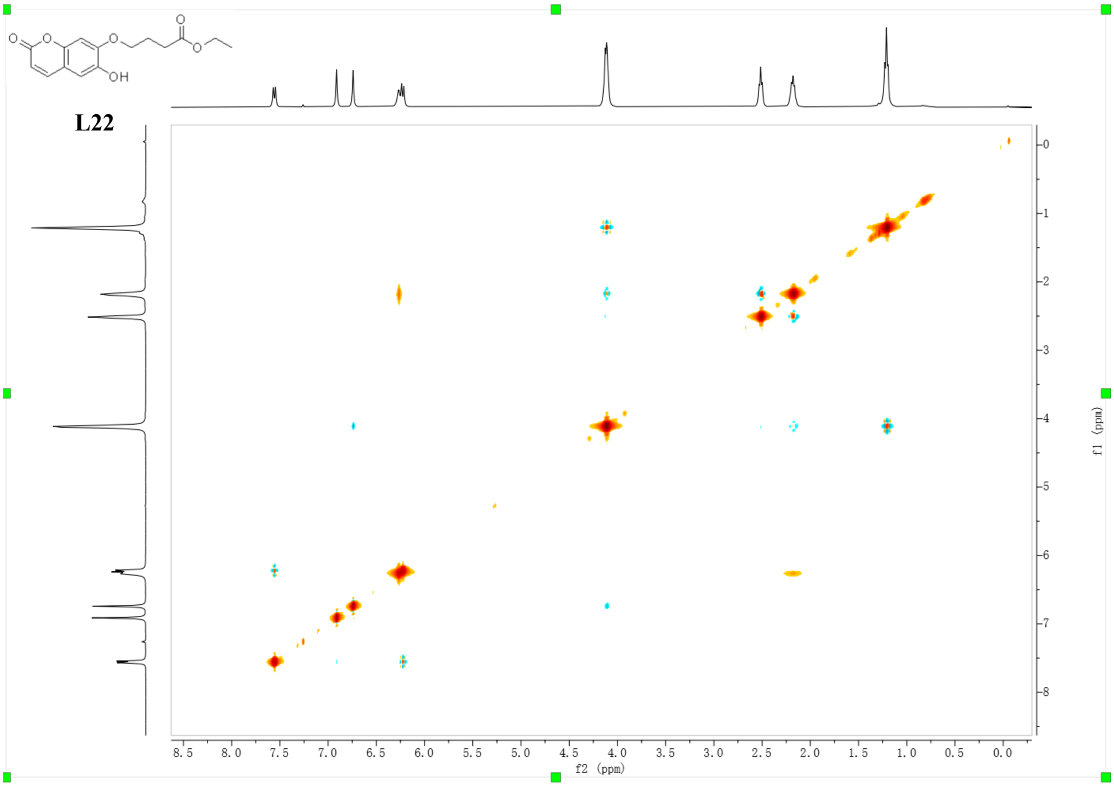

NOESY spectroscopic data of **L22**

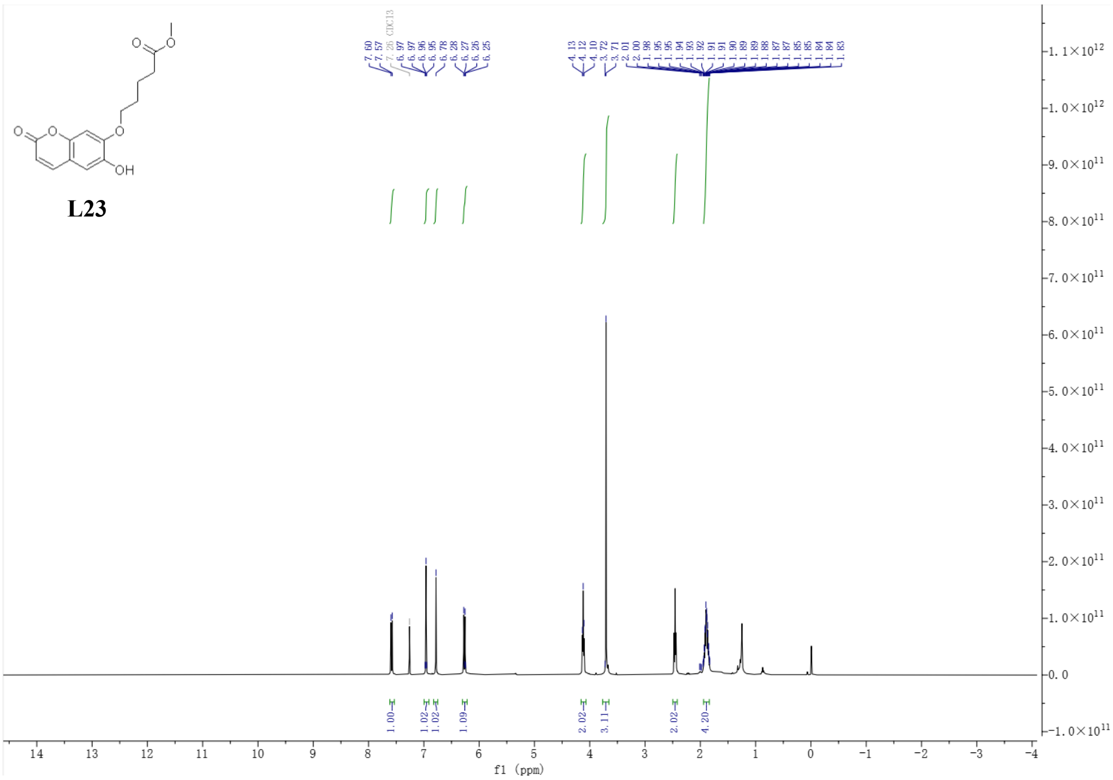

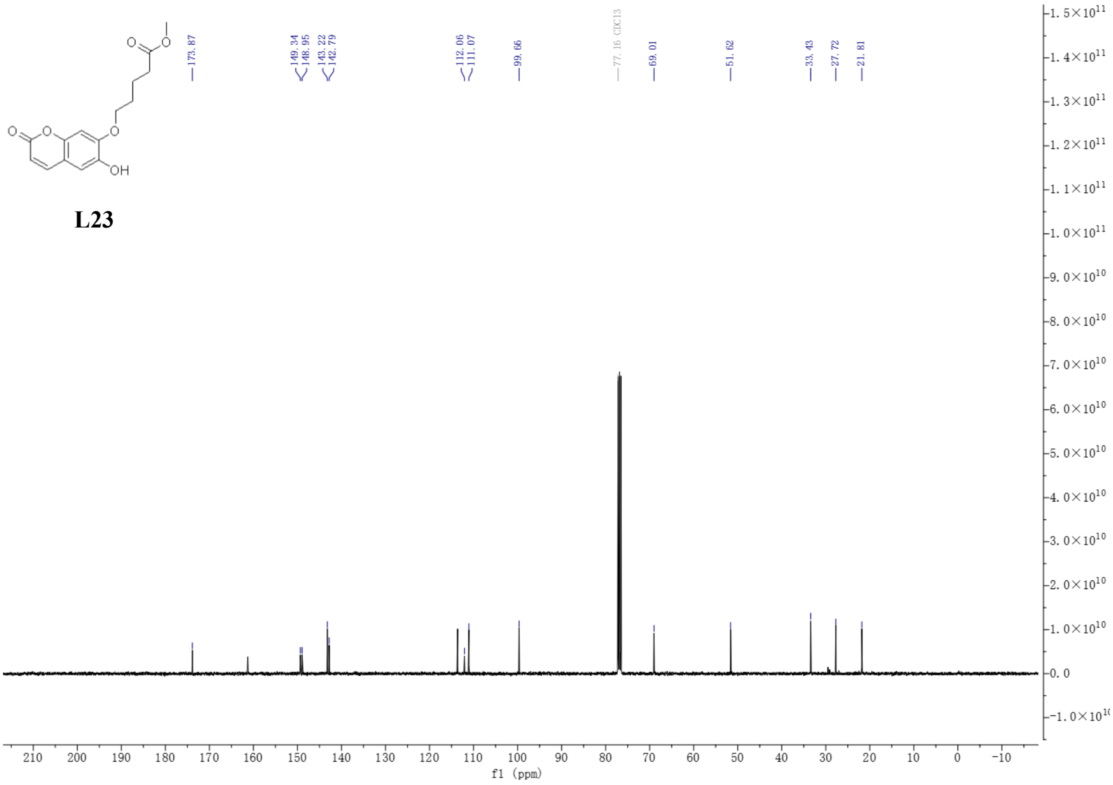

^{1}H NMR and ^{13}C NMR spectroscopic data of **L23**

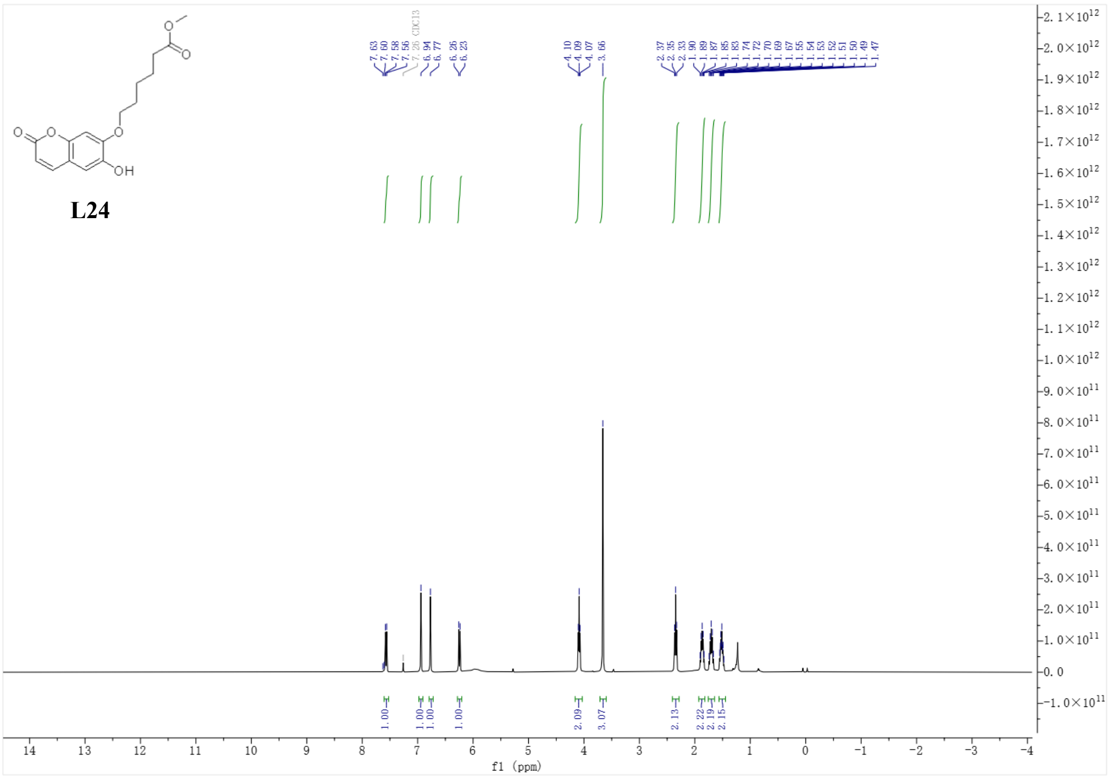

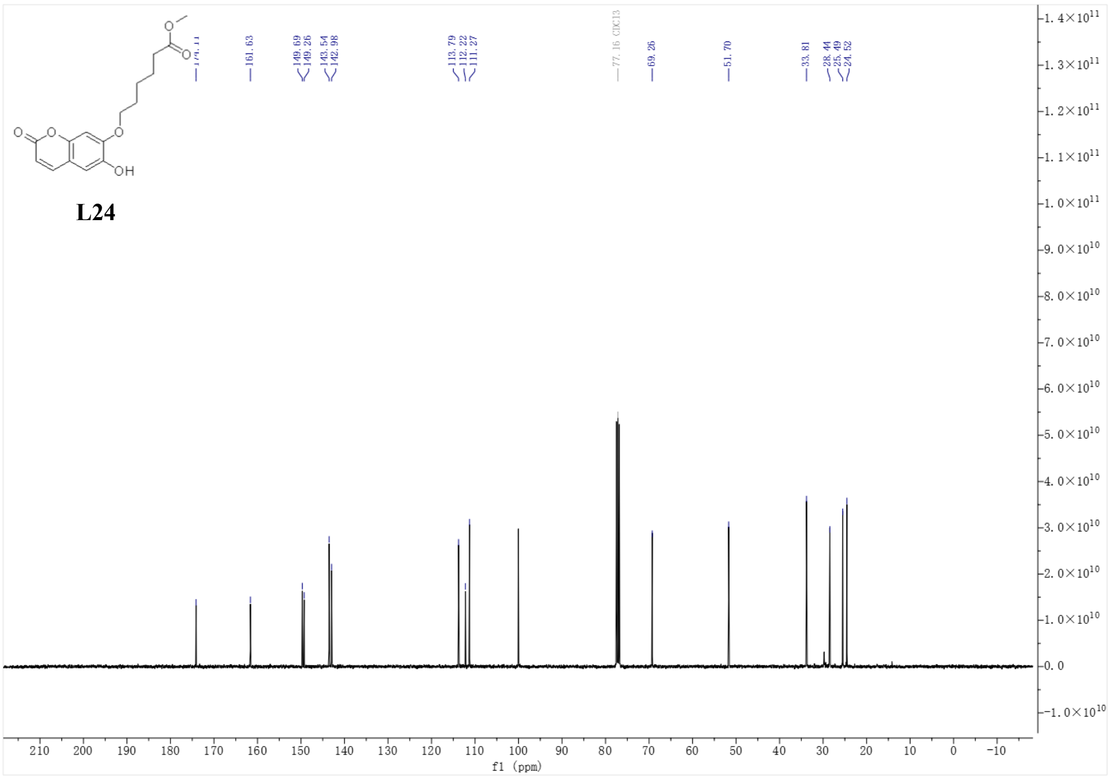

^{1}H NMR and ^{13}C NMR spectroscopic data of **L24**

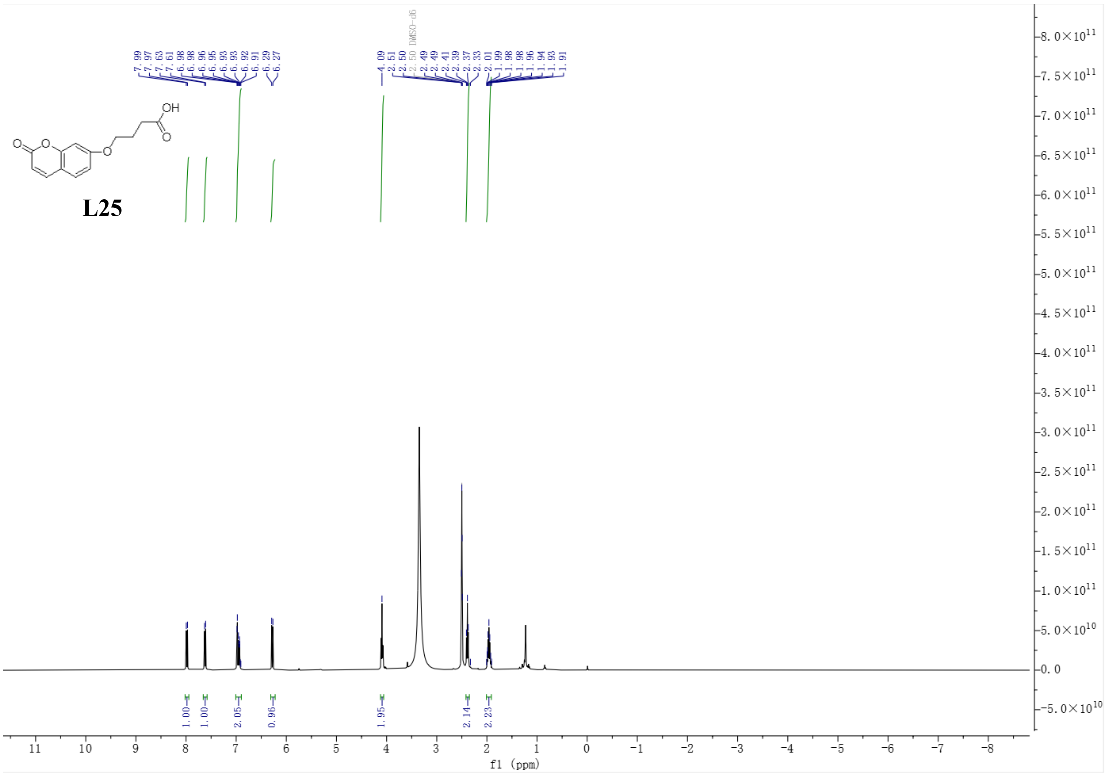

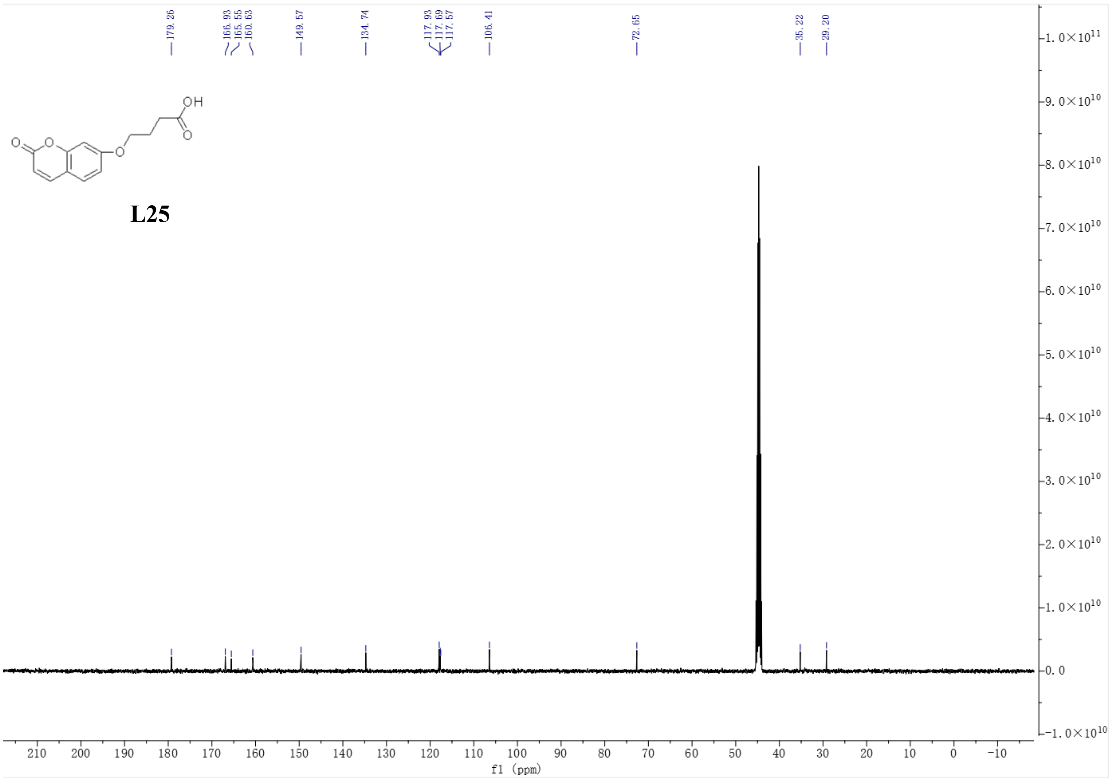

^{1}H NMR and ^{13}C NMR spectroscopic data of **L25**

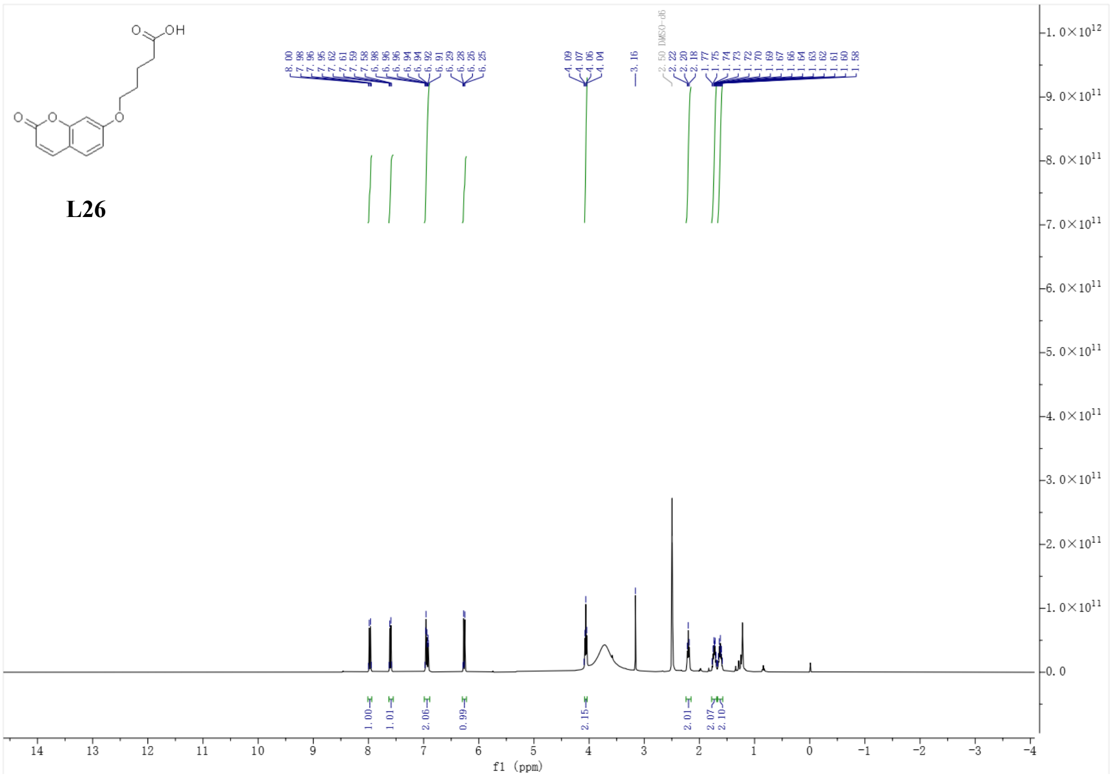

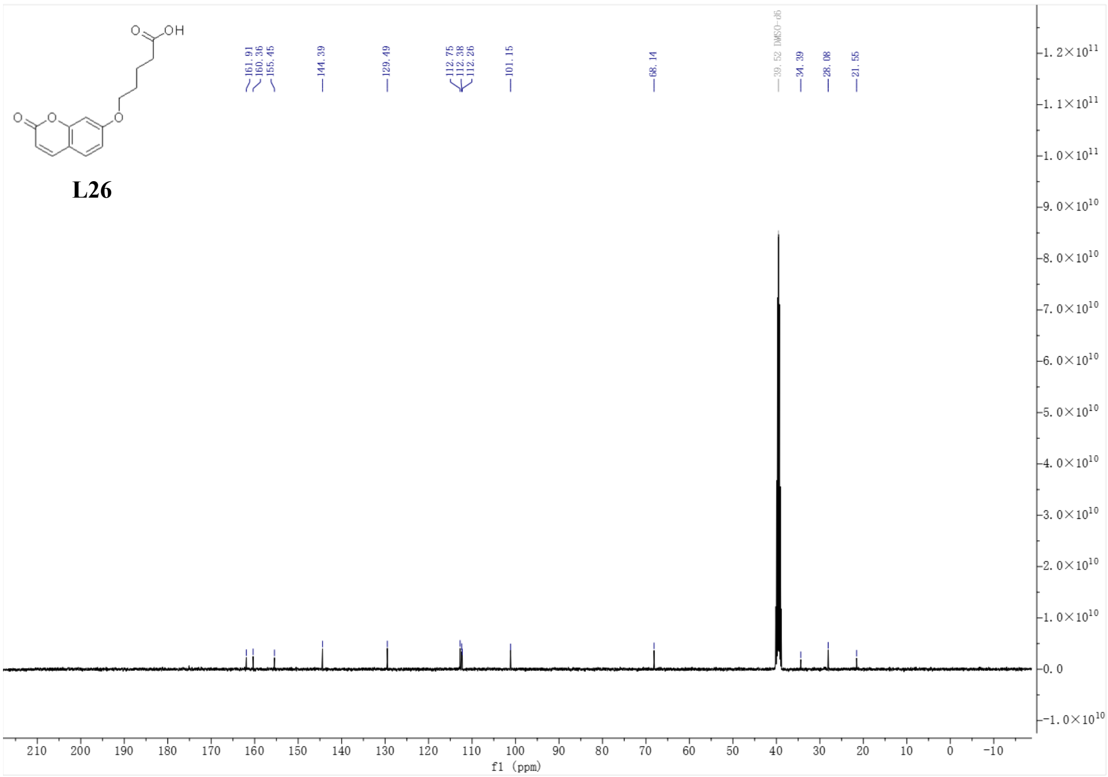

^{1}H NMR and ^{13}C NMR spectroscopic data of **L26**

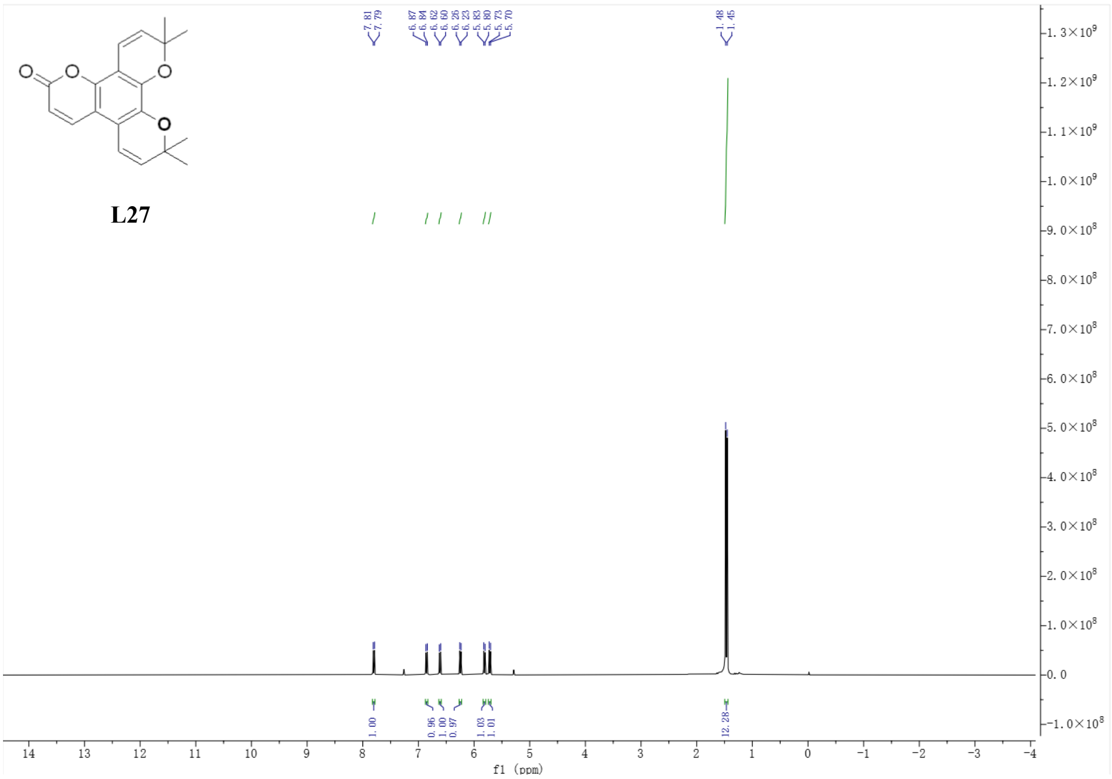

L27

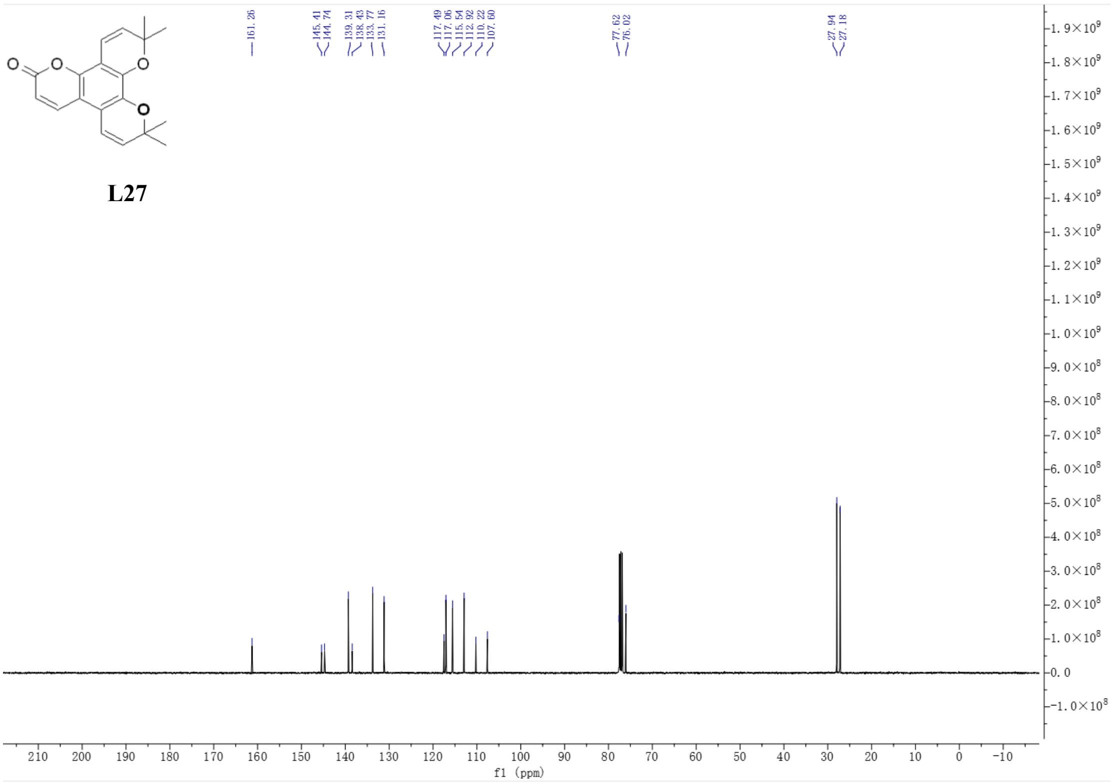

L27

^{1}H NMR and ^{13}C NMR spectroscopic data of **L27**

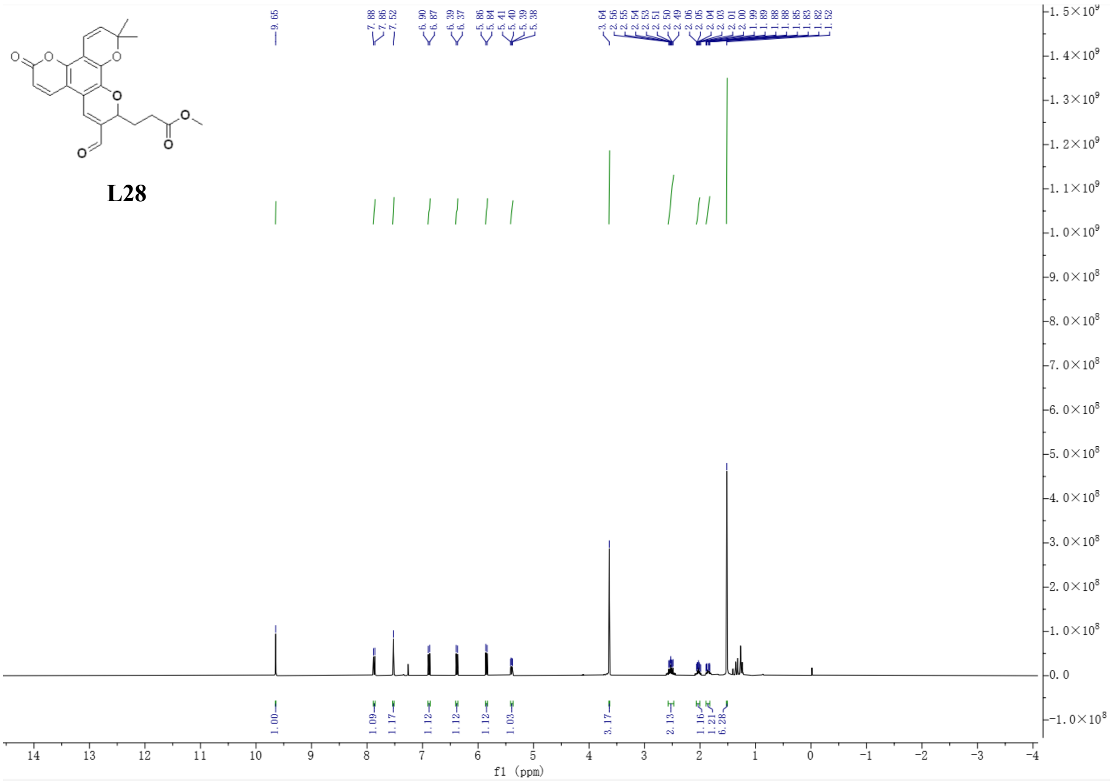

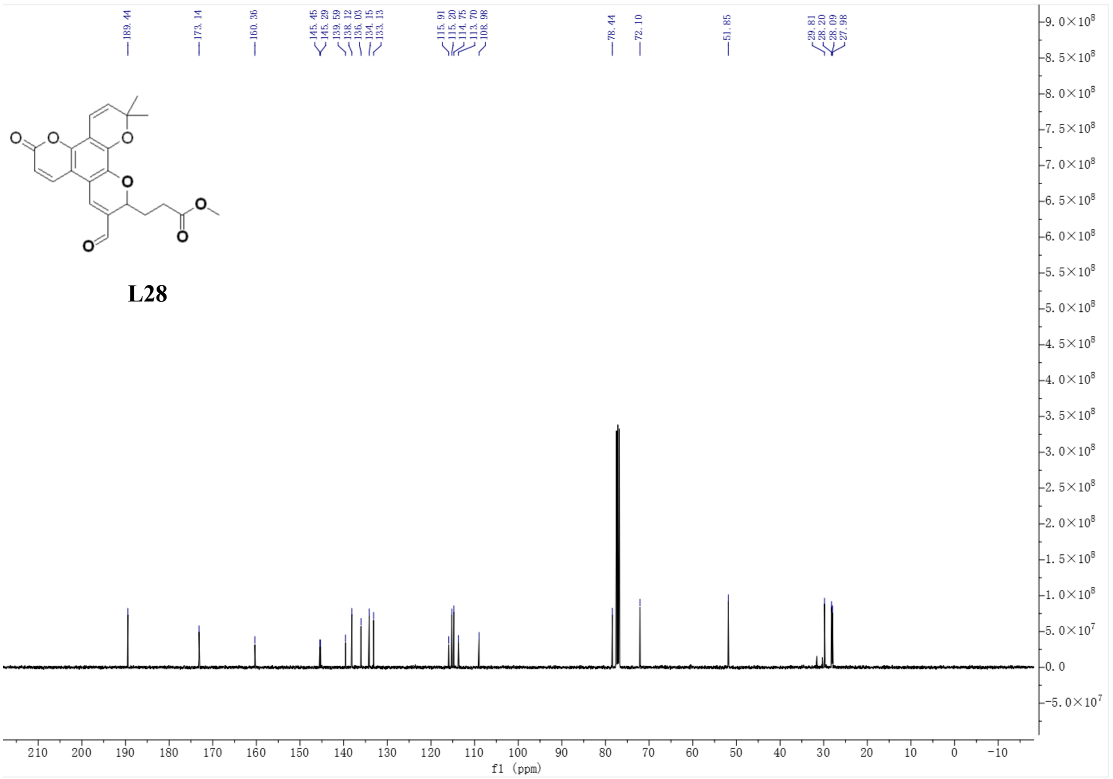

^{1}H NMR and ^{13}C NMR spectroscopic data of **L28**

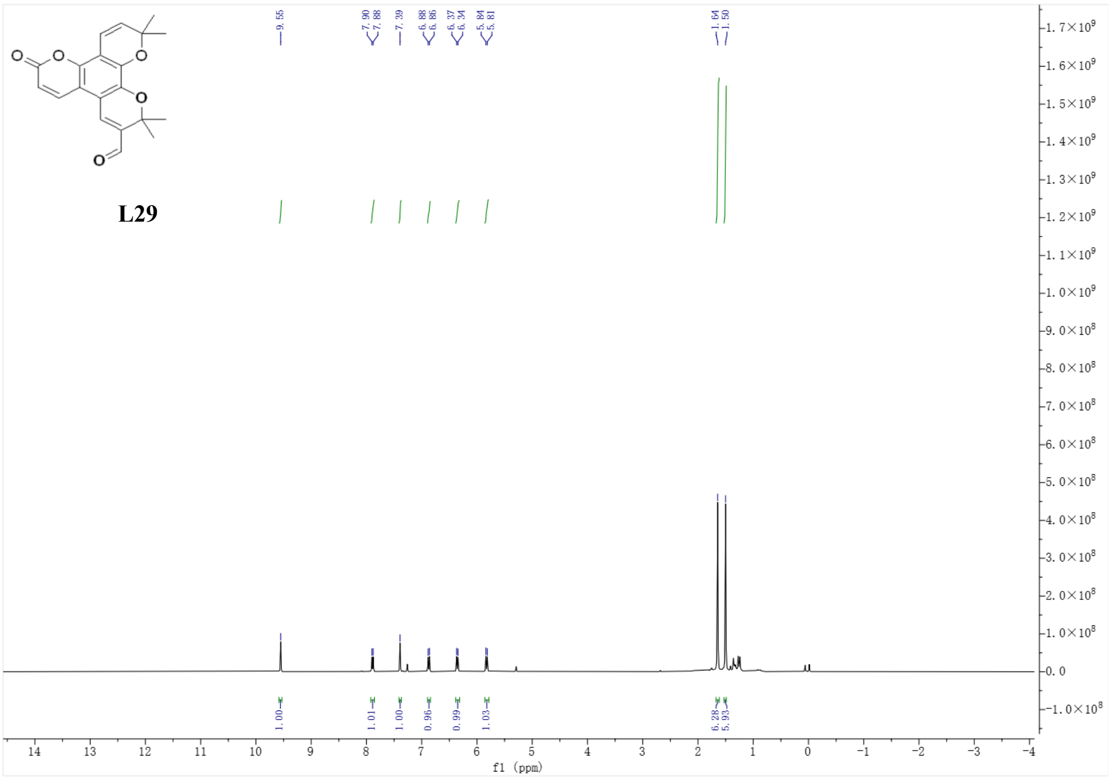

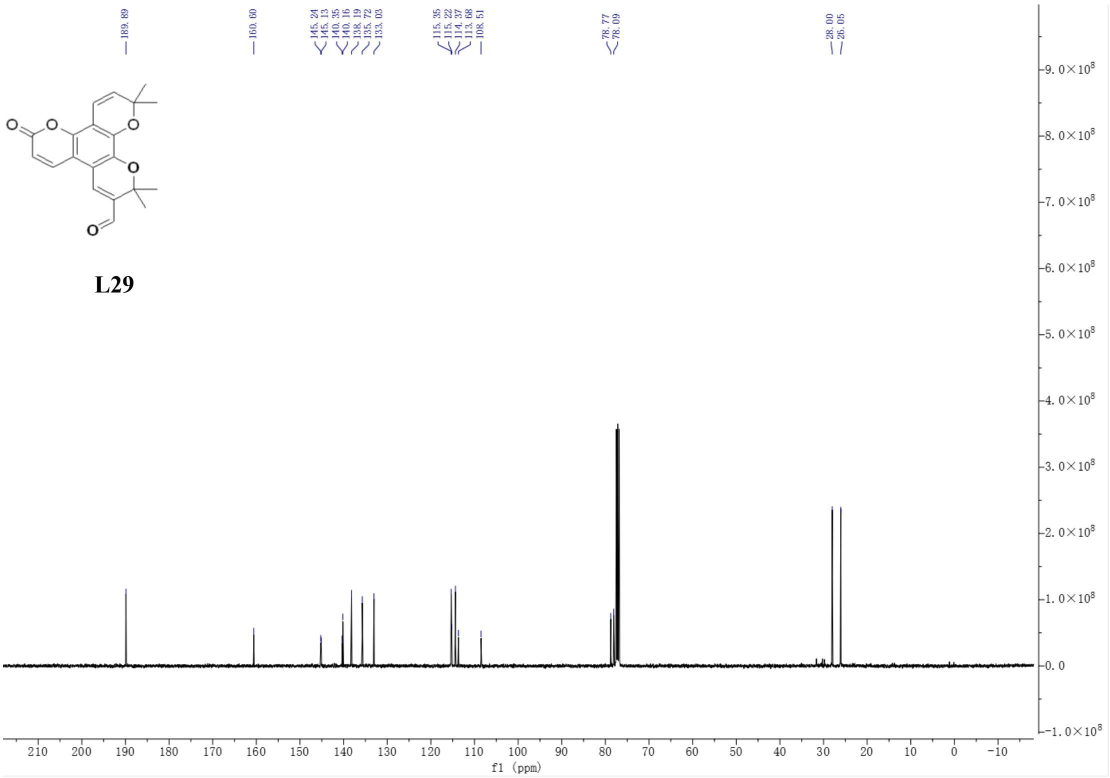

^{1}H NMR and ^{13}C NMR spectroscopic data of **L29**

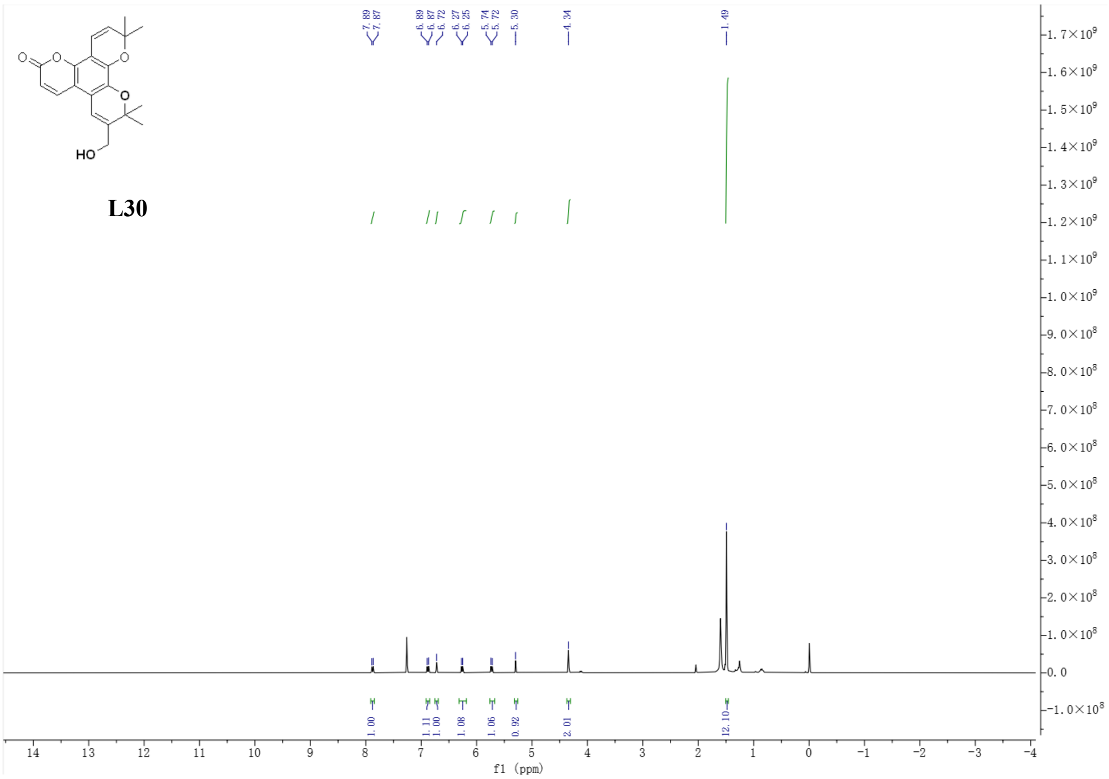

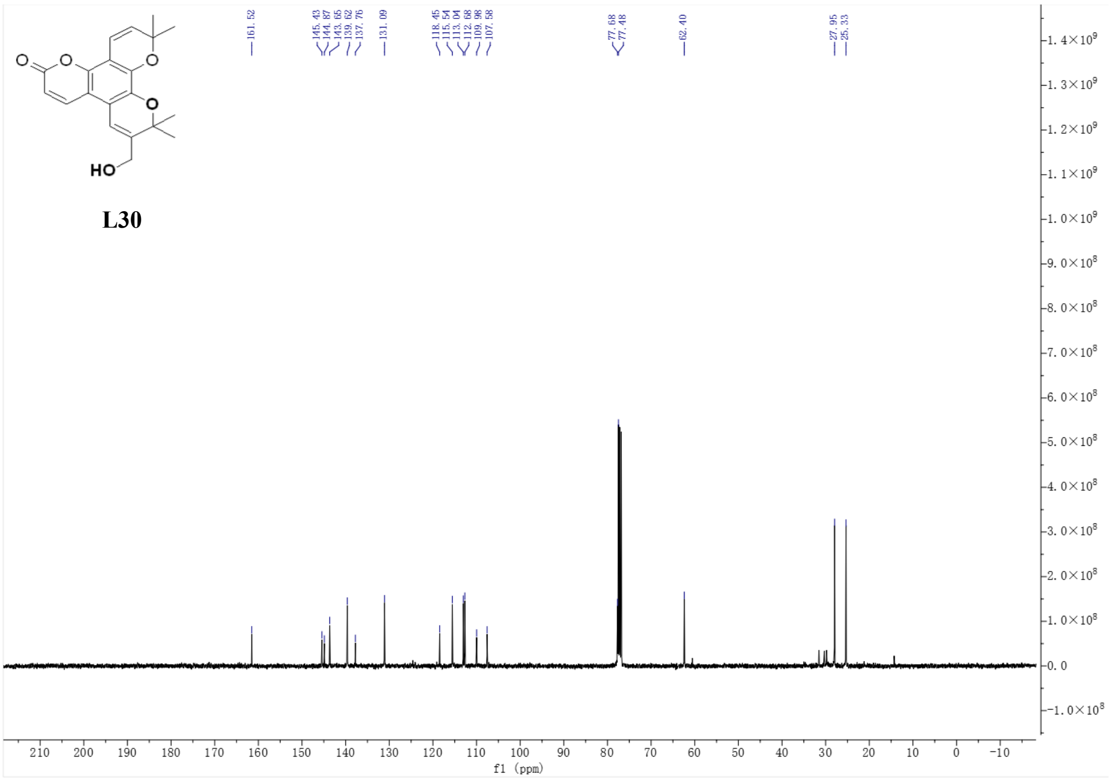

^{1}H NMR and ^{13}C NMR spectroscopic data of **L30**

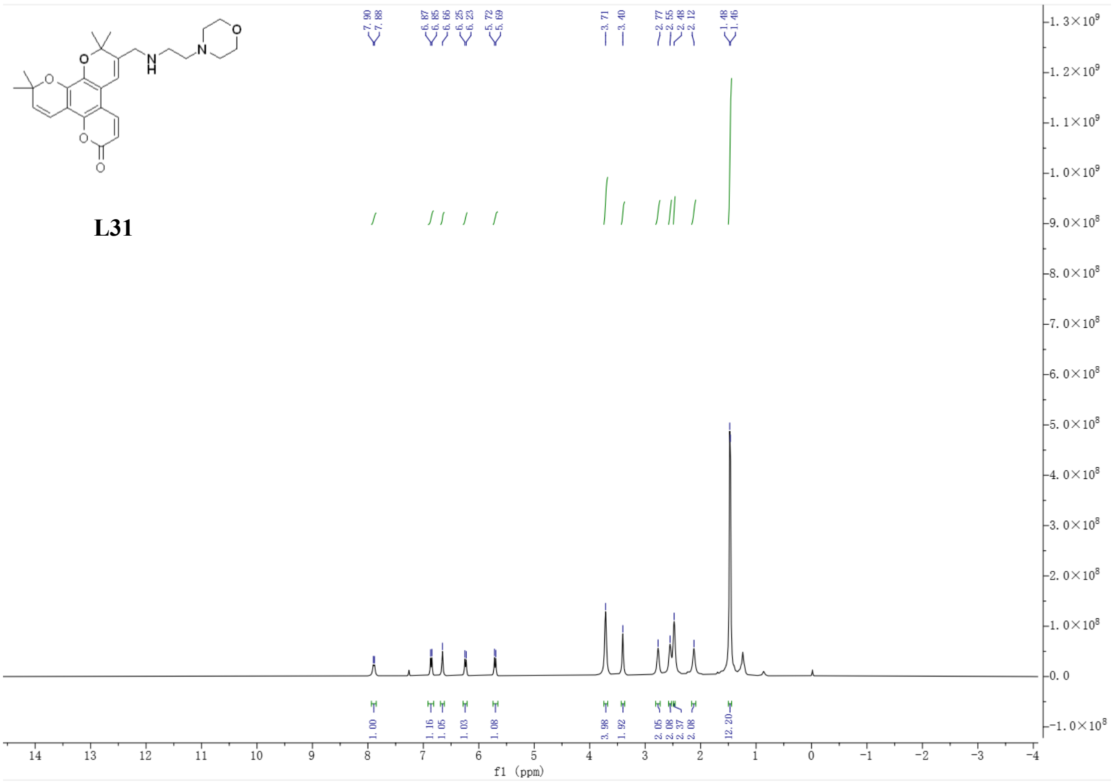

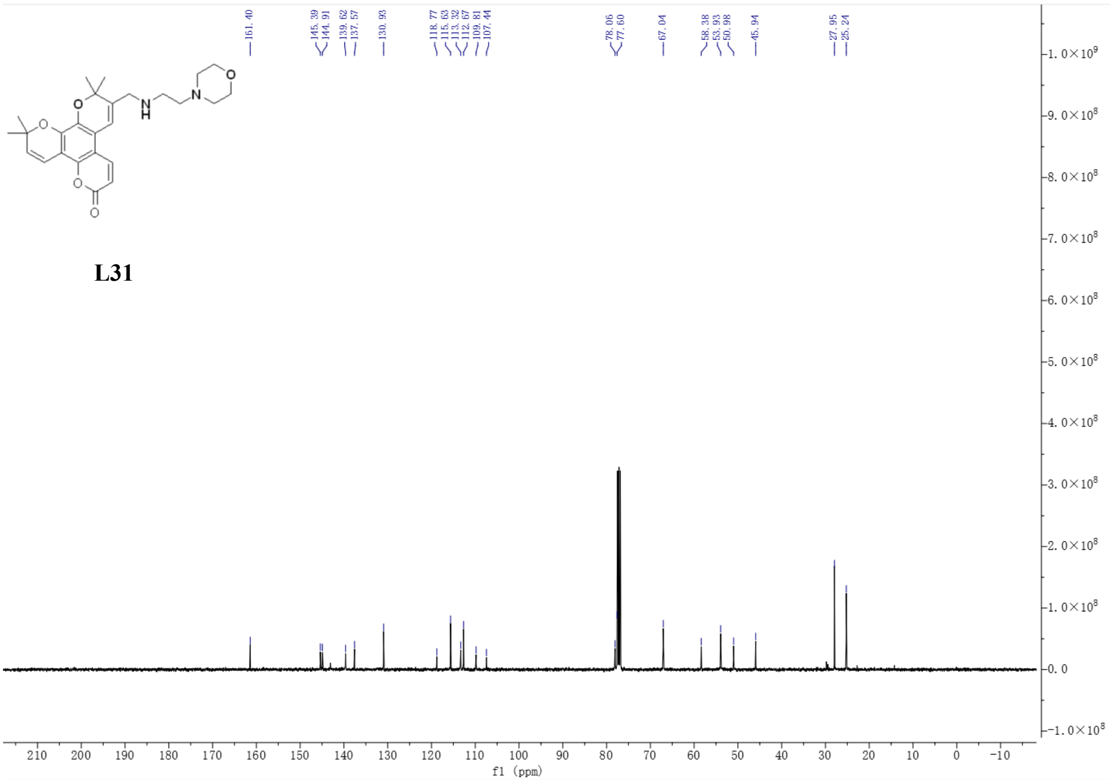

^{1}H NMR and ^{13}C NMR spectroscopic data of **L31**

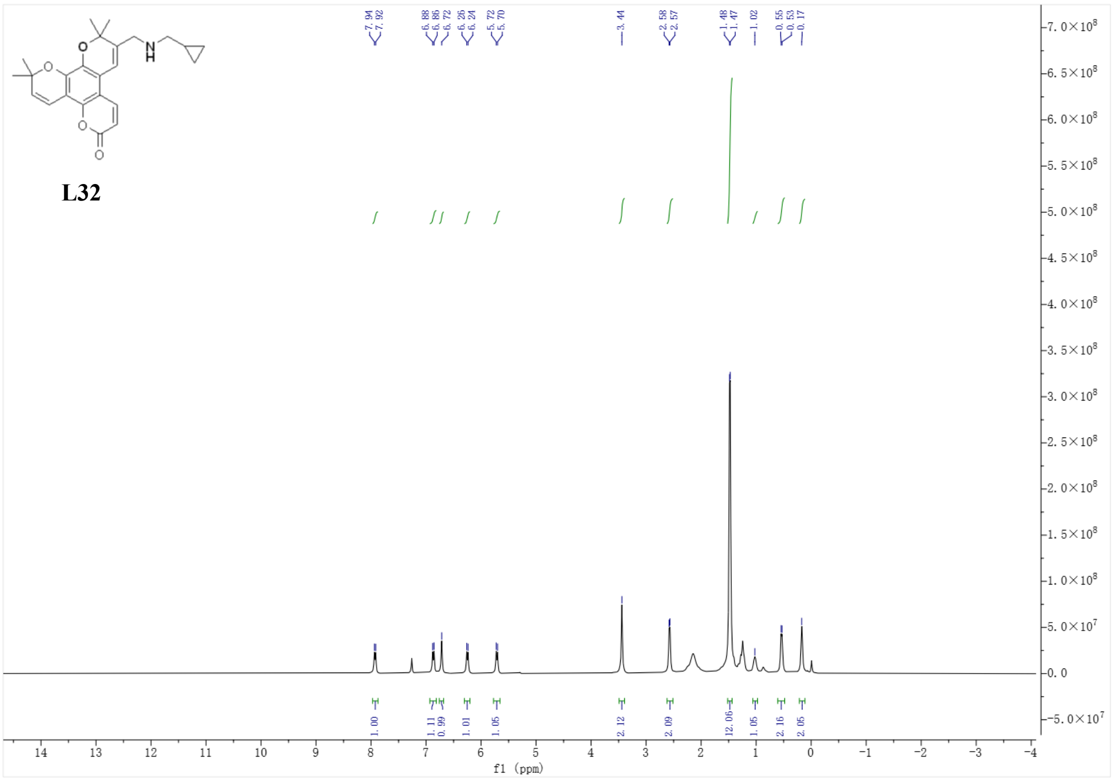

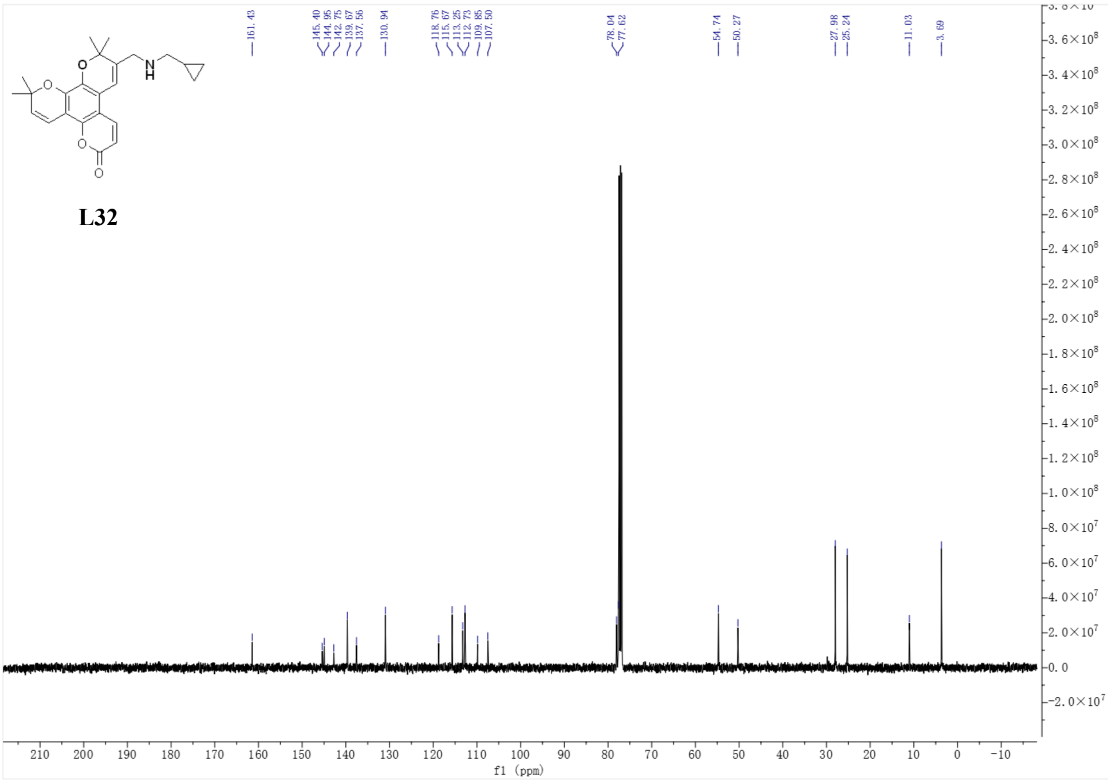

^{1}H NMR and ^{13}C NMR spectroscopic data of **L32**

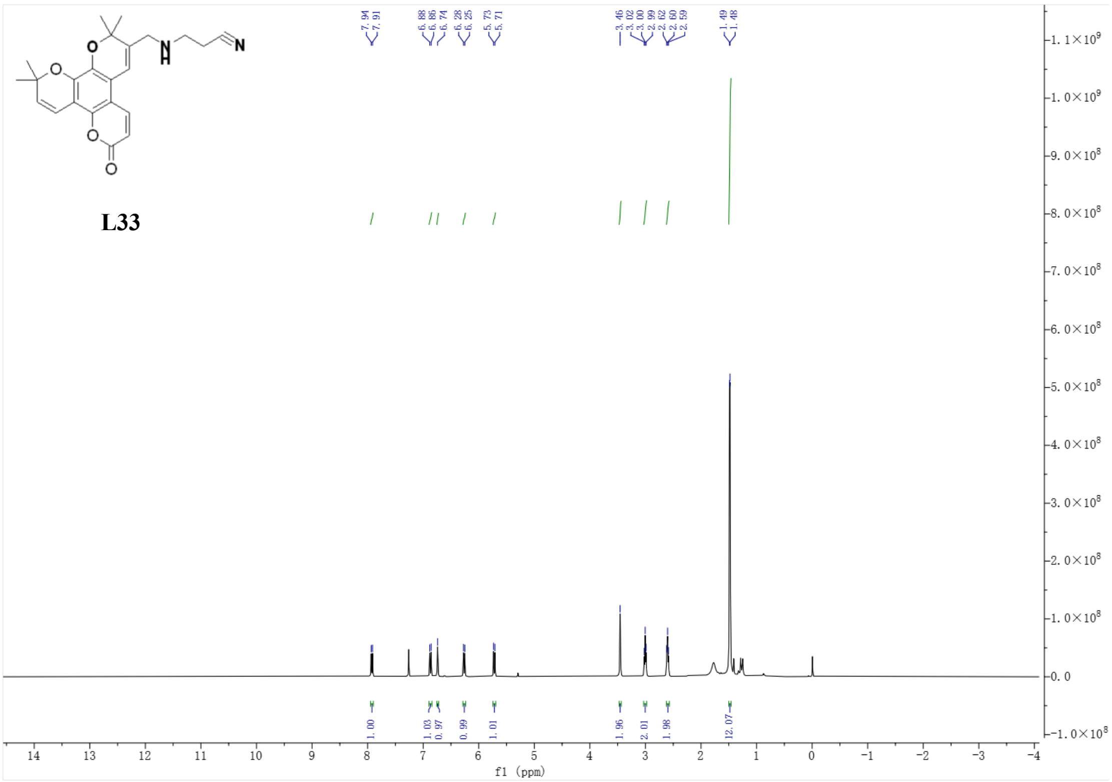

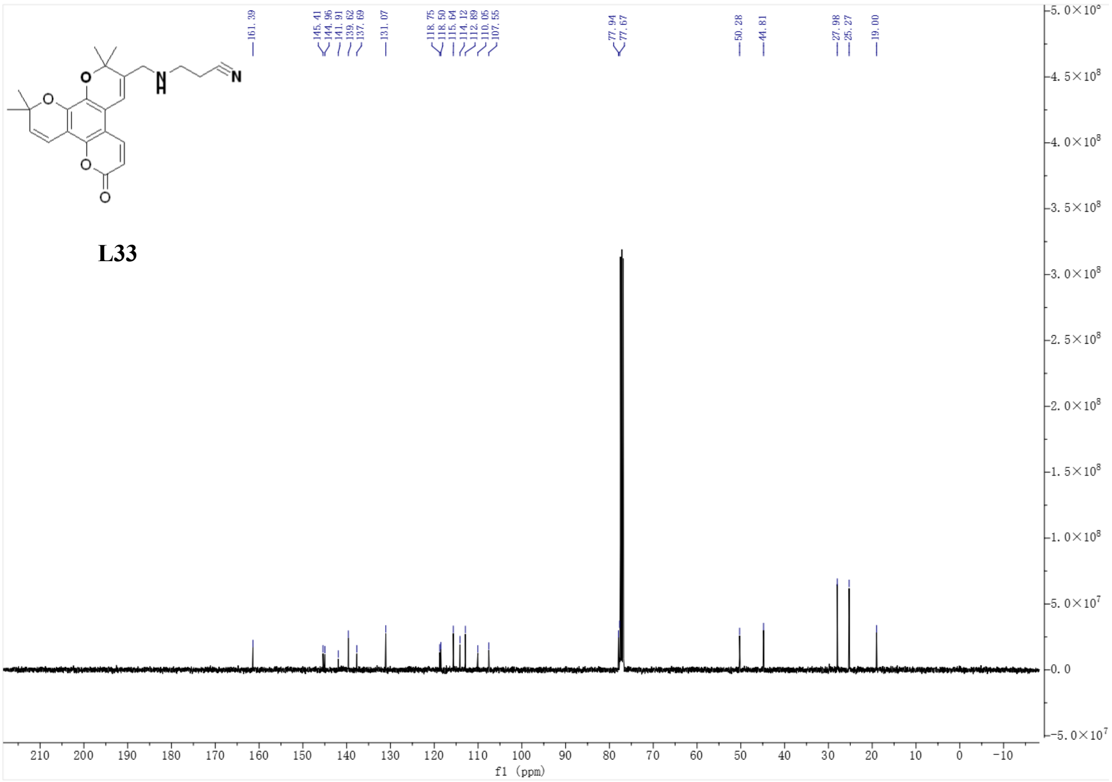

^{1}H NMR and ^{13}C NMR spectroscopic data of **L33**

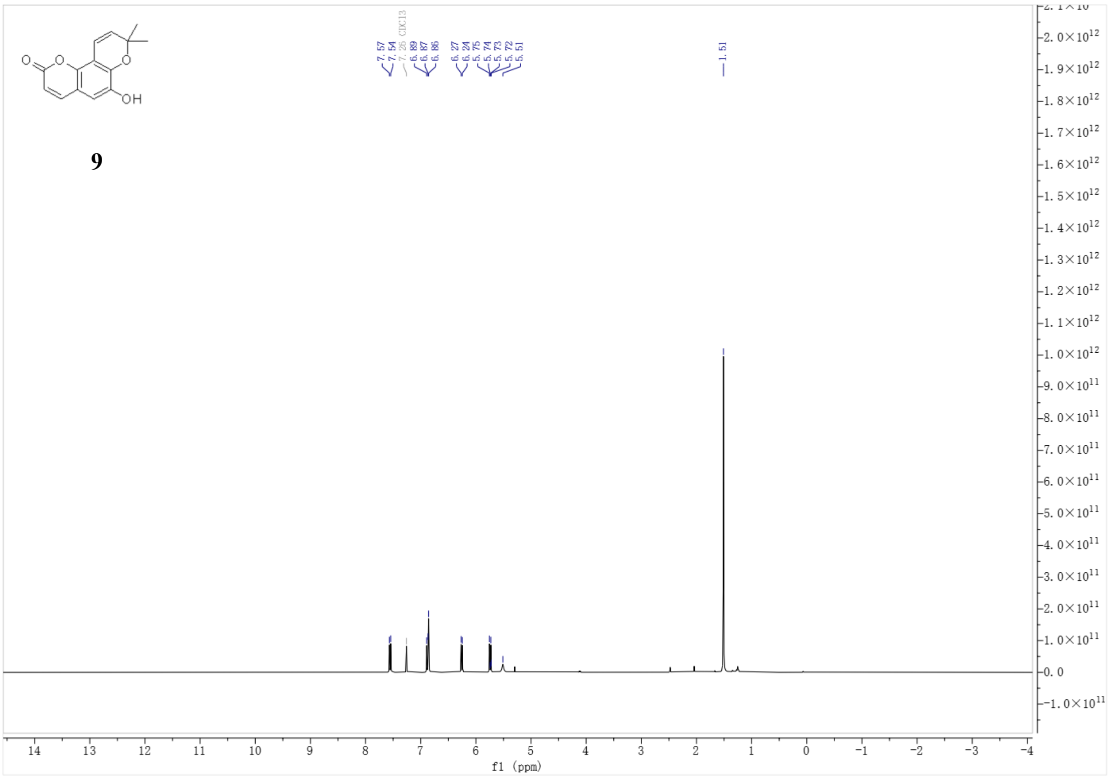

^{1}H NMR spectroscopic data of **9**

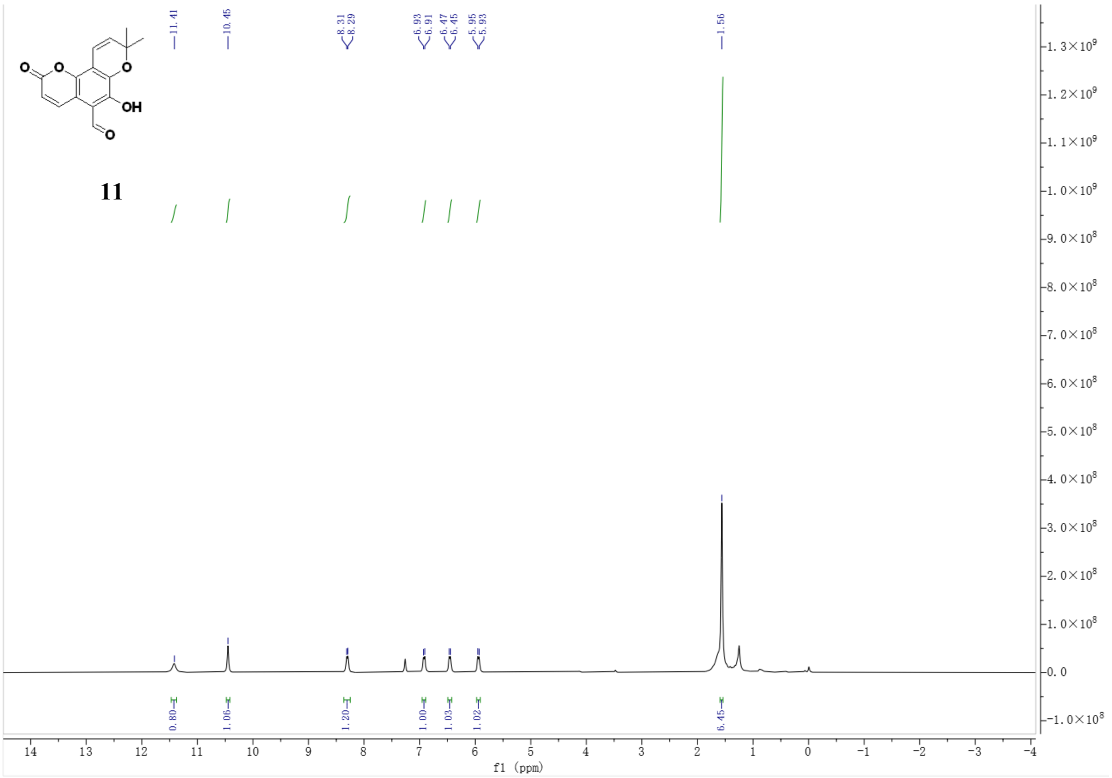

^{1}H NMR spectroscopic data of **11**

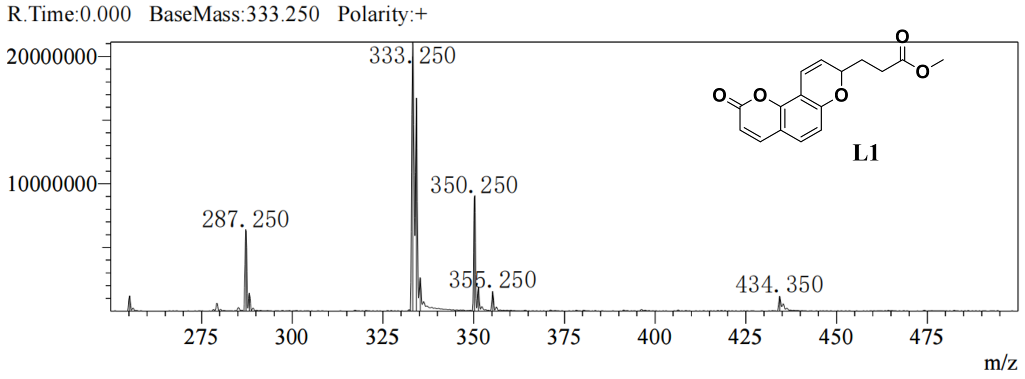

MS data of **L1**

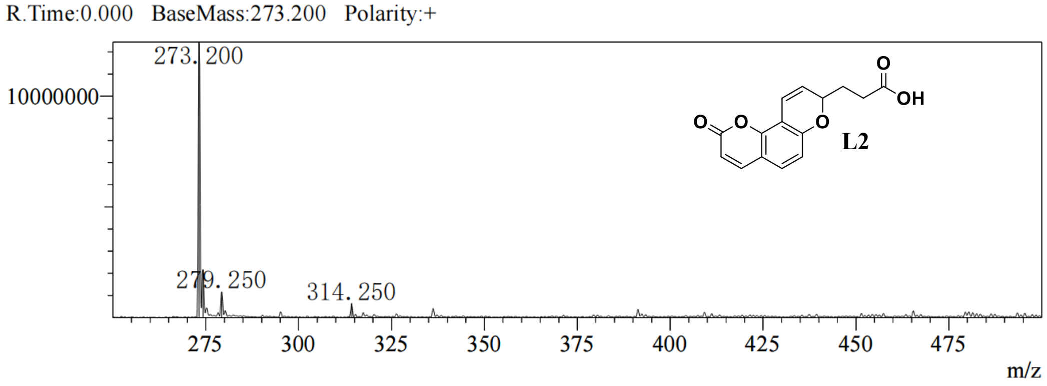

MS data of **L2**

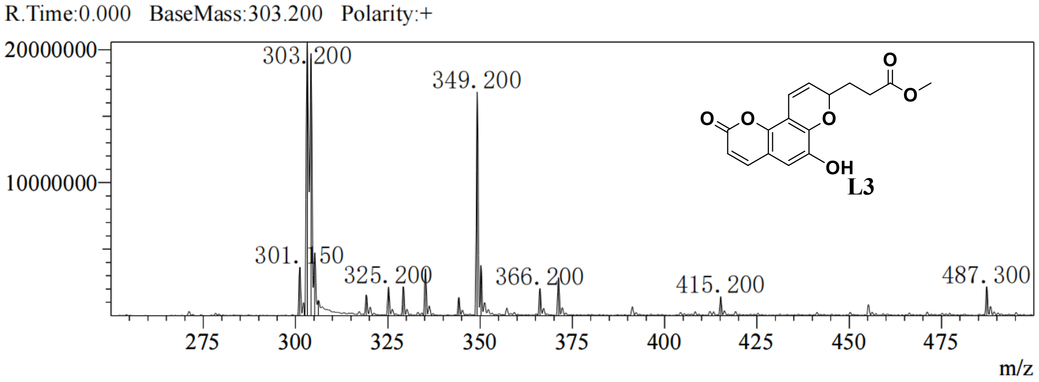

MS data of **L3**

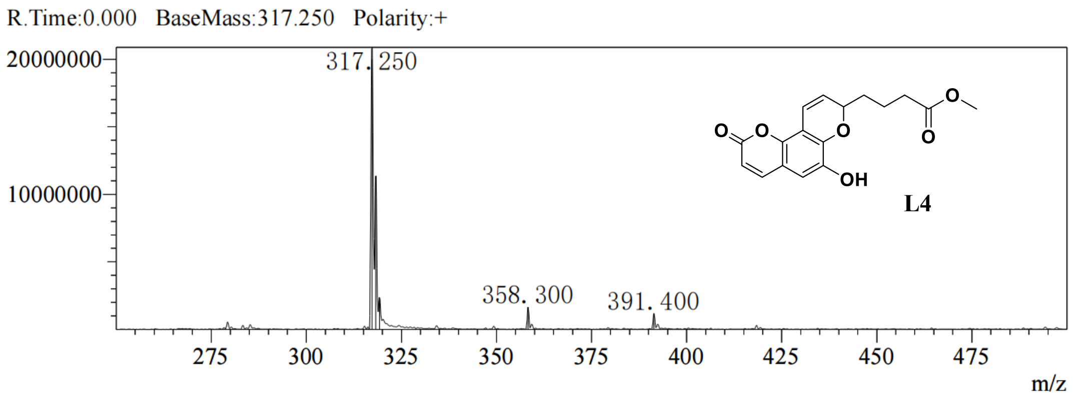

MS data of **L4**

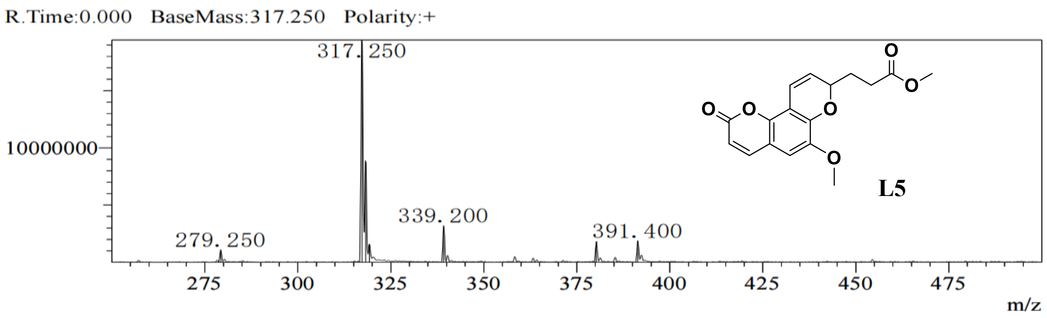

MS data of **L5**

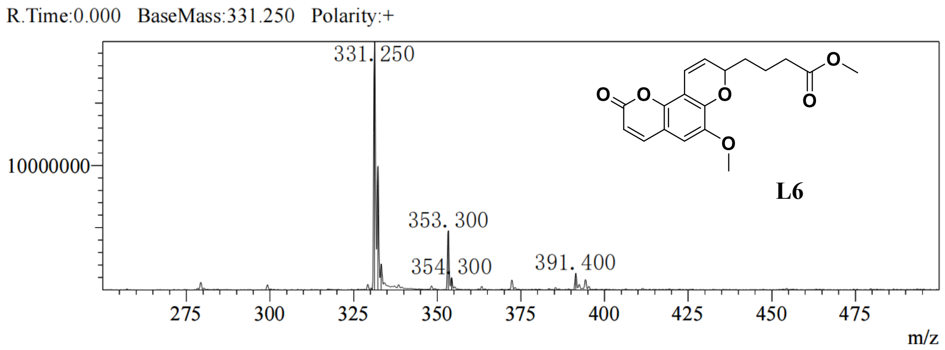

MS data of **L6**

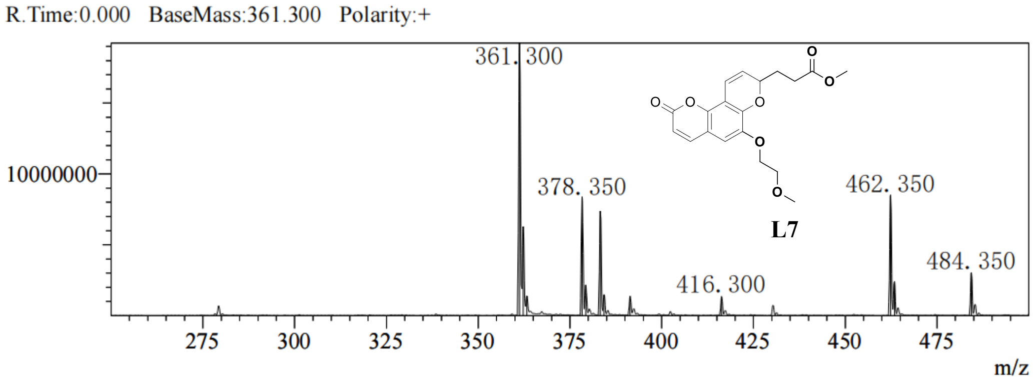

MS data of **L7**

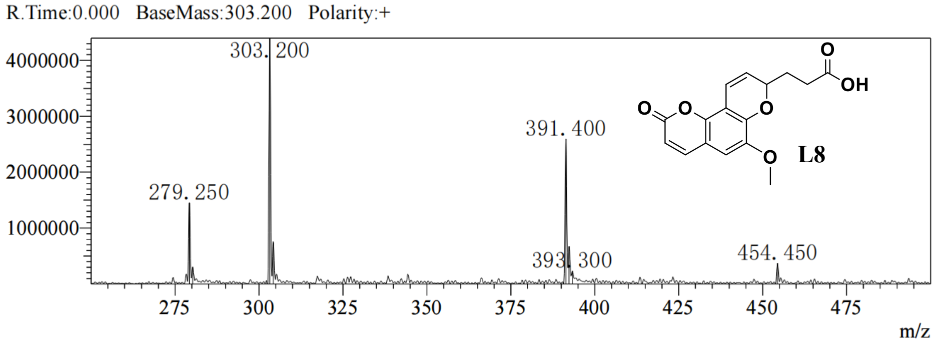

MS data of **L8**

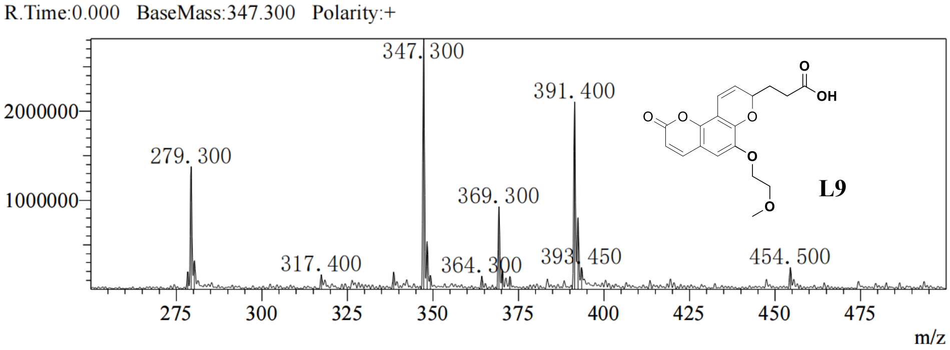

MS data of **L9**

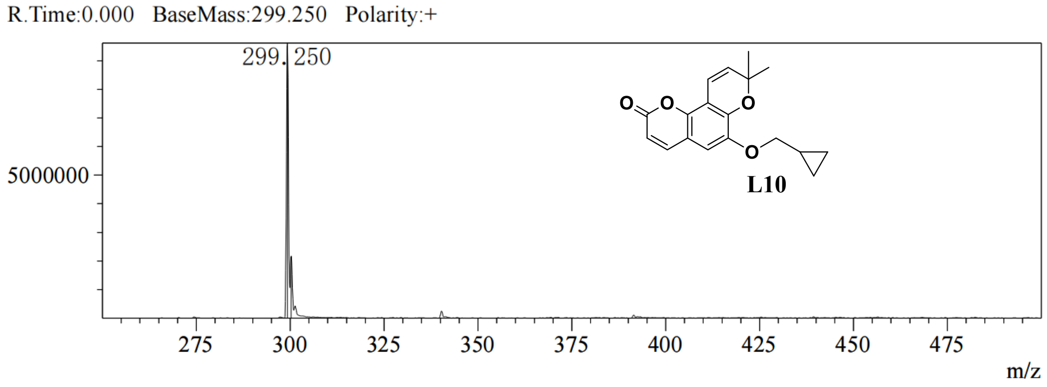

MS data of **L10**

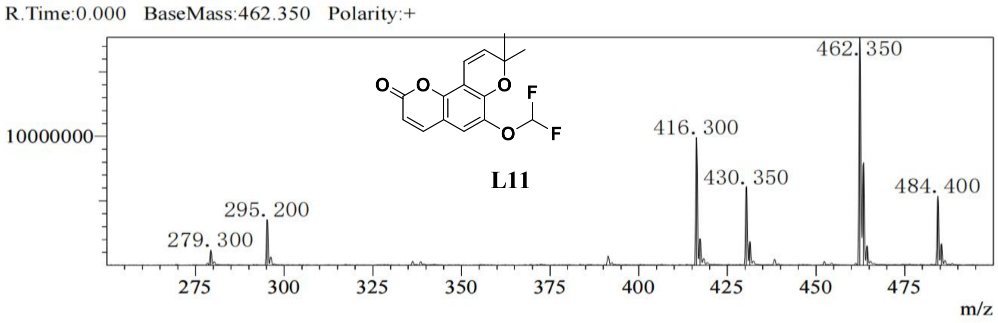

MS data of **L11**

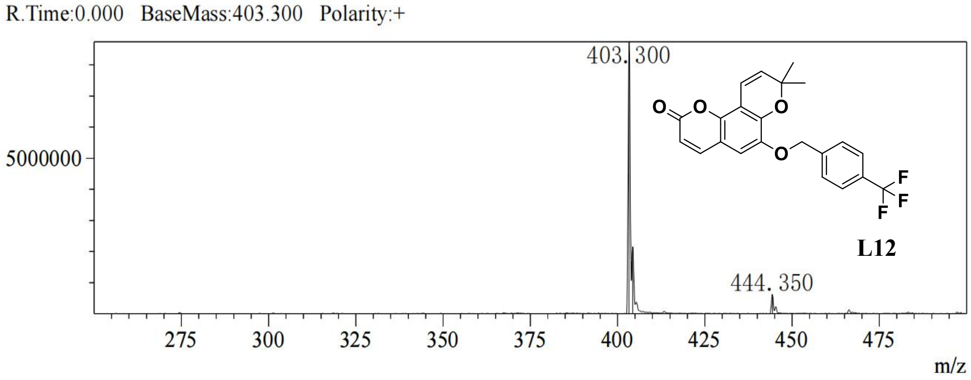

MS data of **L12**

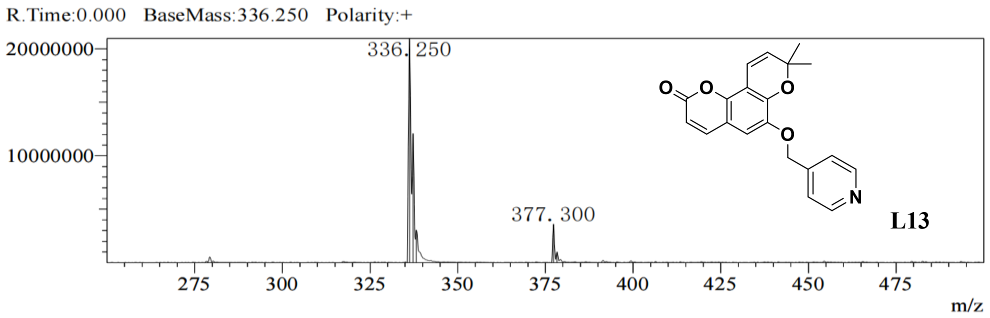

MS data of **L13**

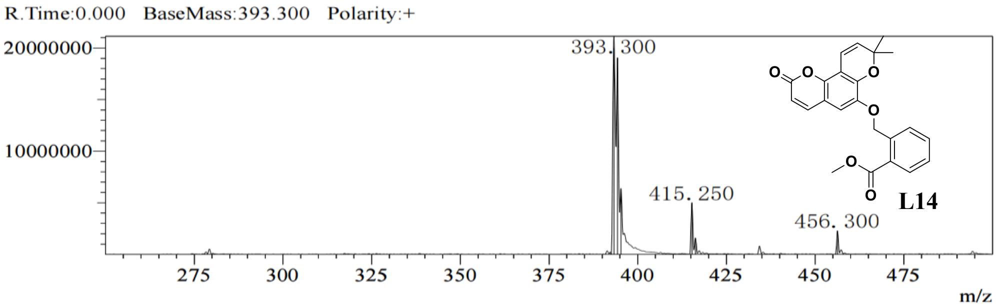

MS data of **L14**

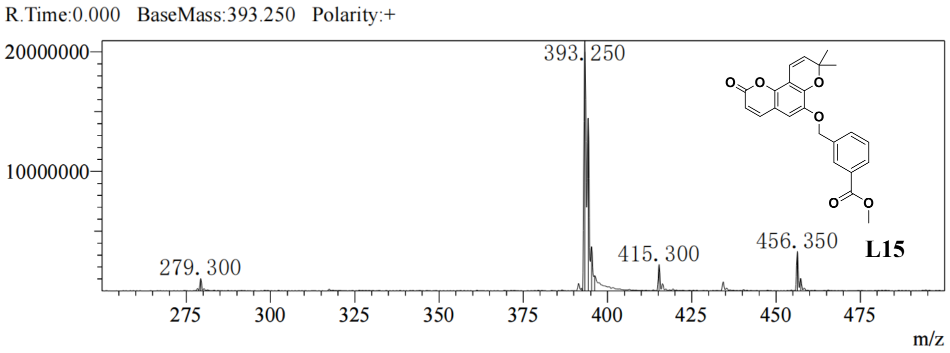

MS data of **L15**

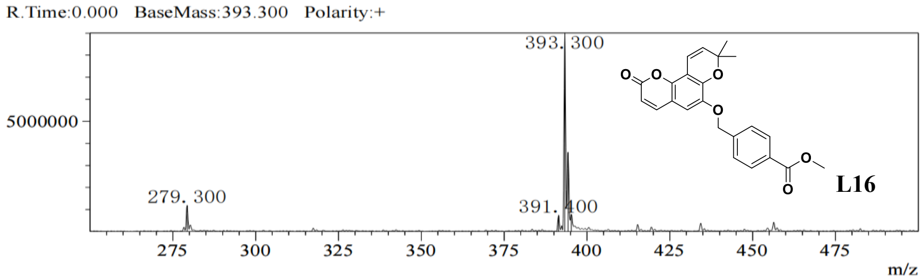

MS data of **L16**

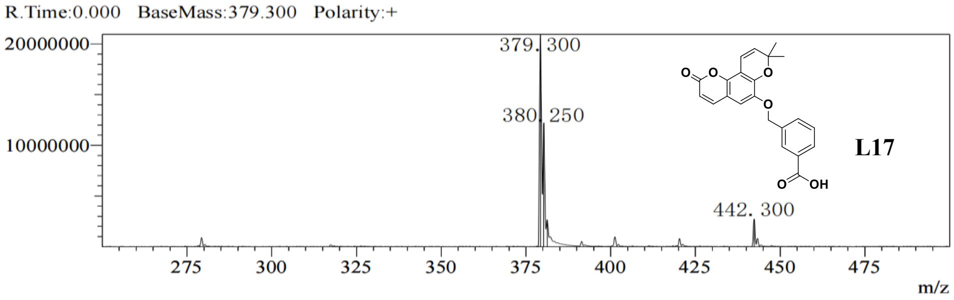

MS data of **L17**

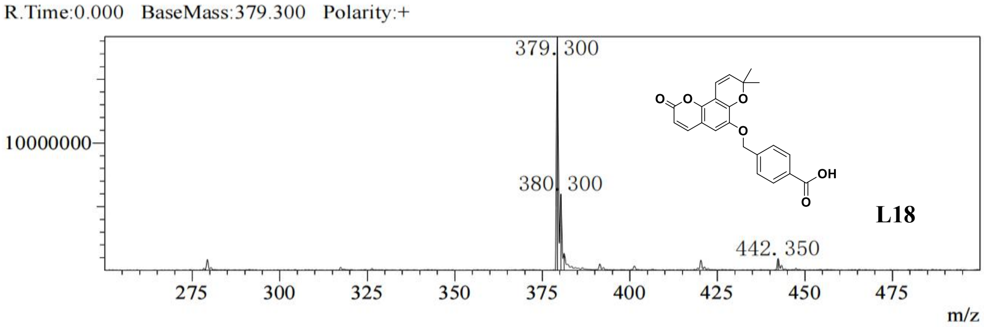

MS data of **L18**

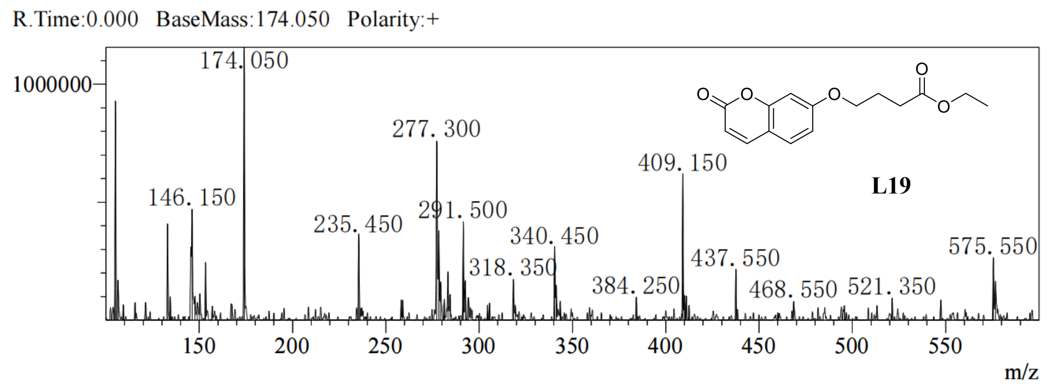

MS data of **L19**

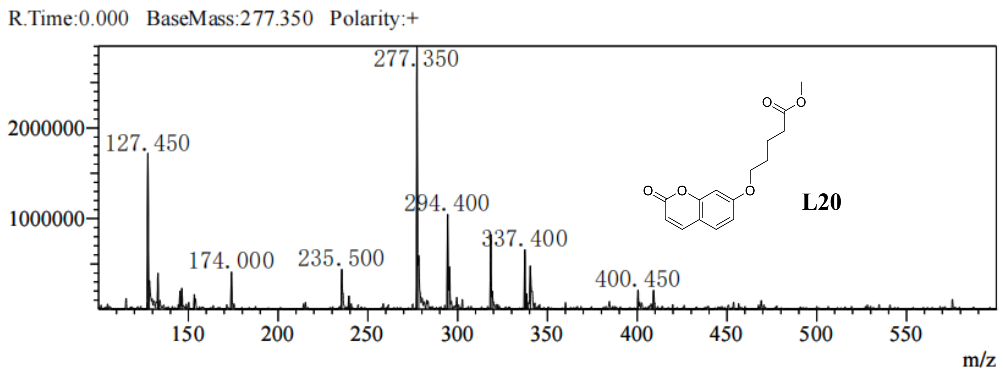

MS data of **L20**

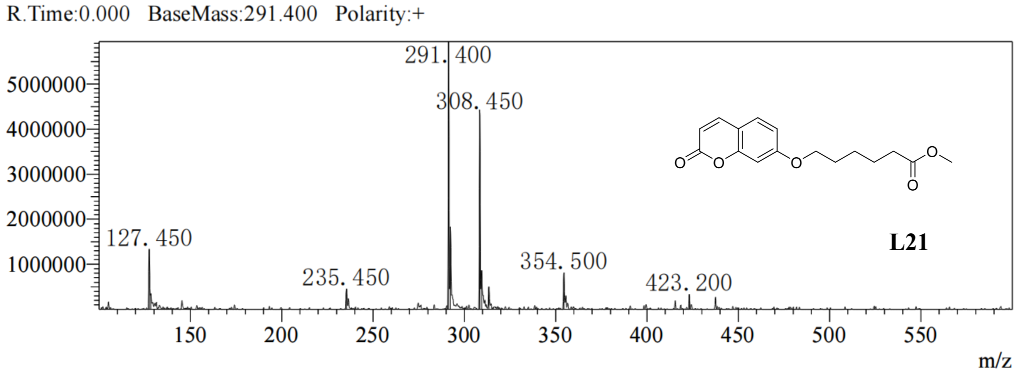

MS data of **L21**

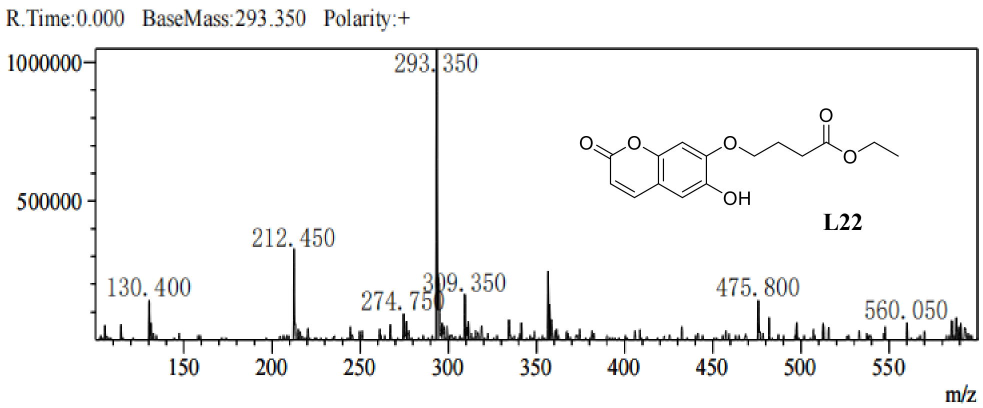

MS data of **L22**

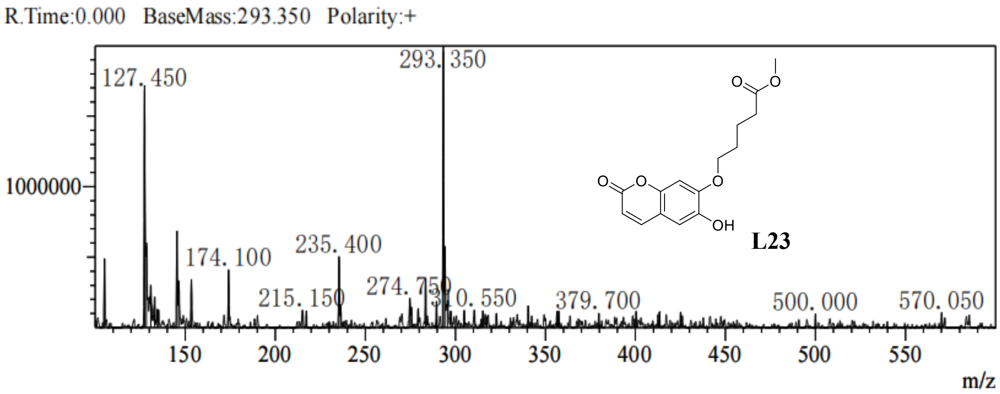

MS data of **L23**

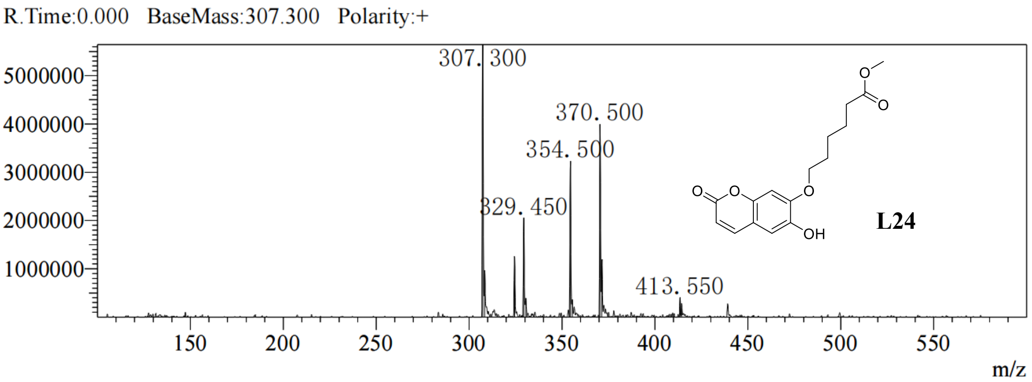

MS data of **L24**

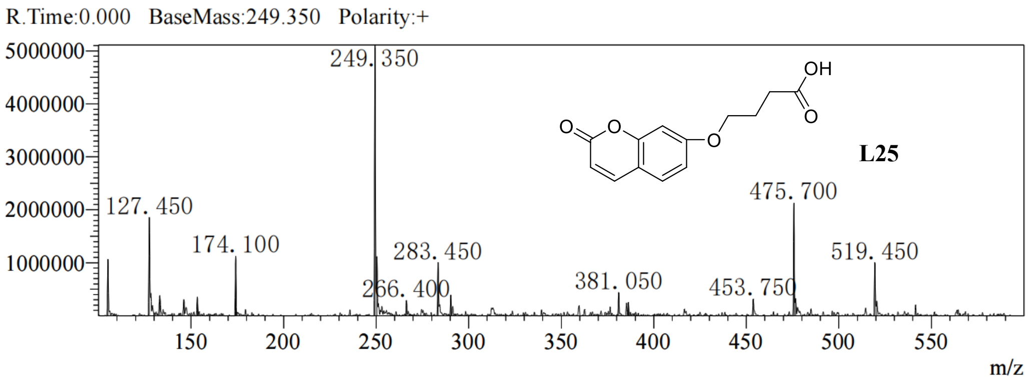

MS data of **L25**

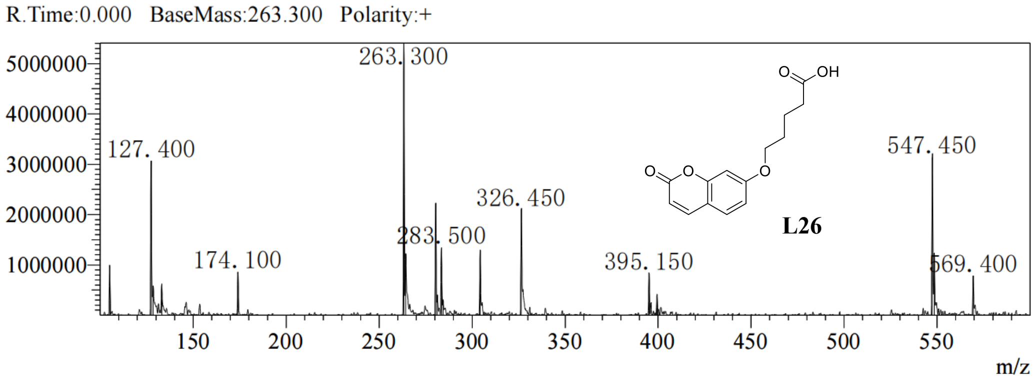

MS data of **L26**

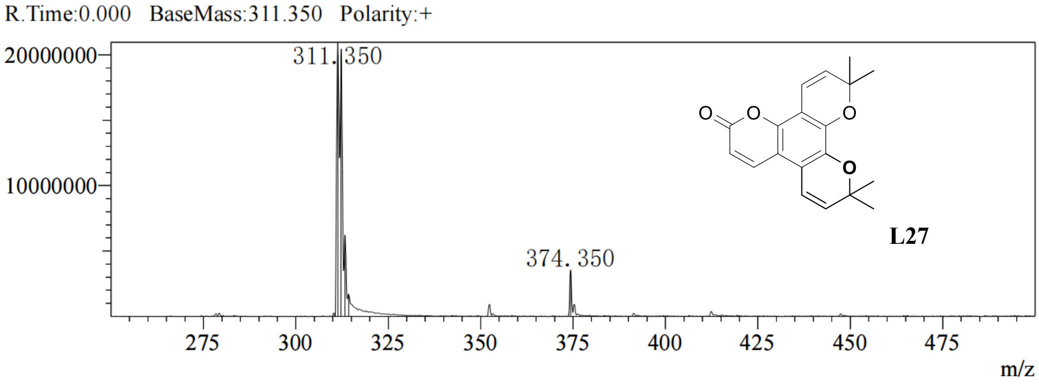

MS data of **L27**

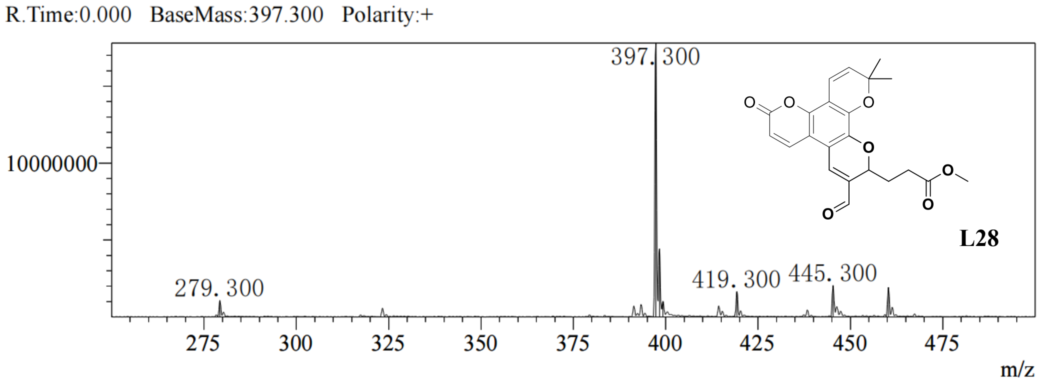

MS data of **L28**

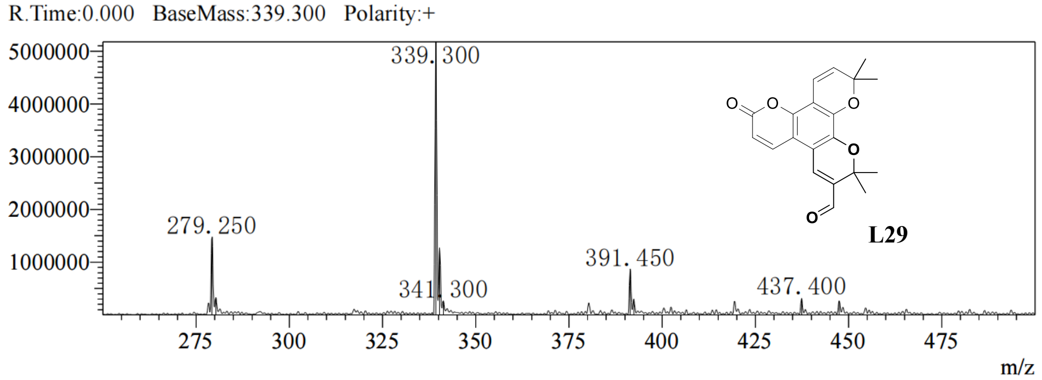

MS data of **L29**

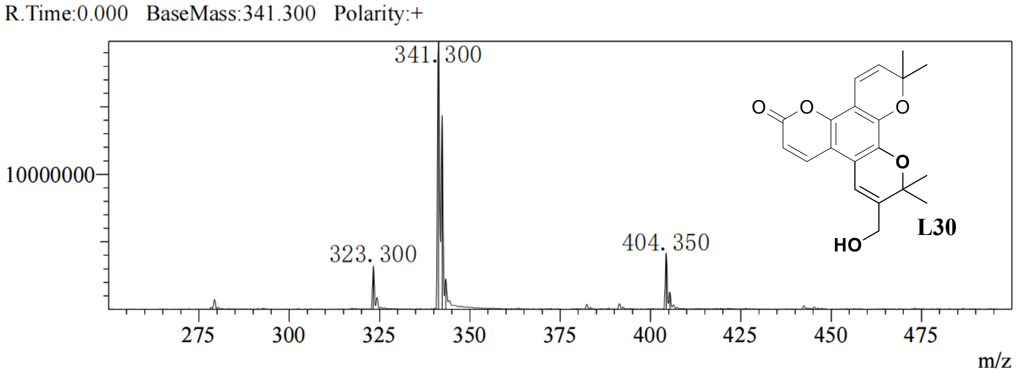

MS data of **L30**

R.Time:0.000 BaseMass:453.400 Polarity:+

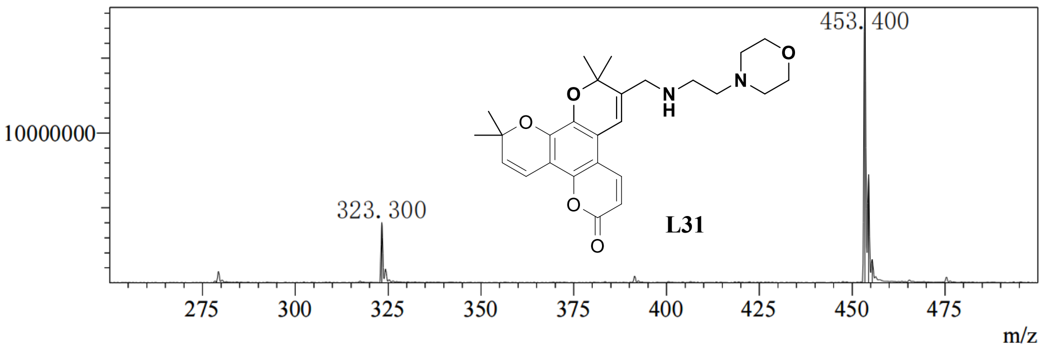

MS data of **L31**

R.Time:0.000 BaseMass:391.250 Polarity:-

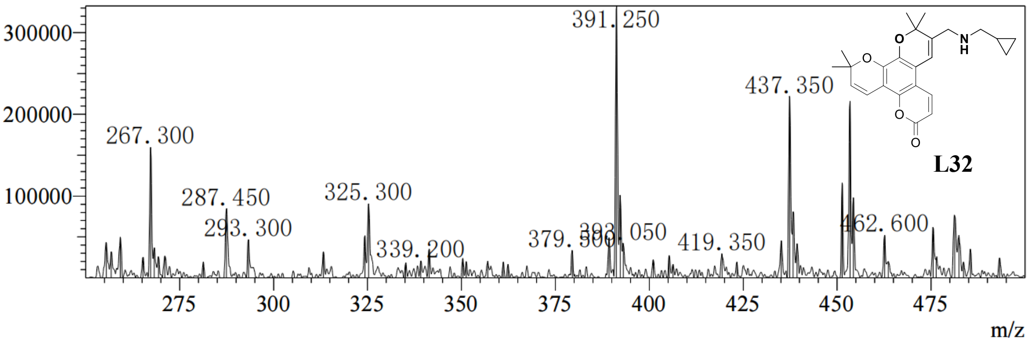

MS data of **L32**

R.Time:0.000 BaseMass:391.350 Polarity:-

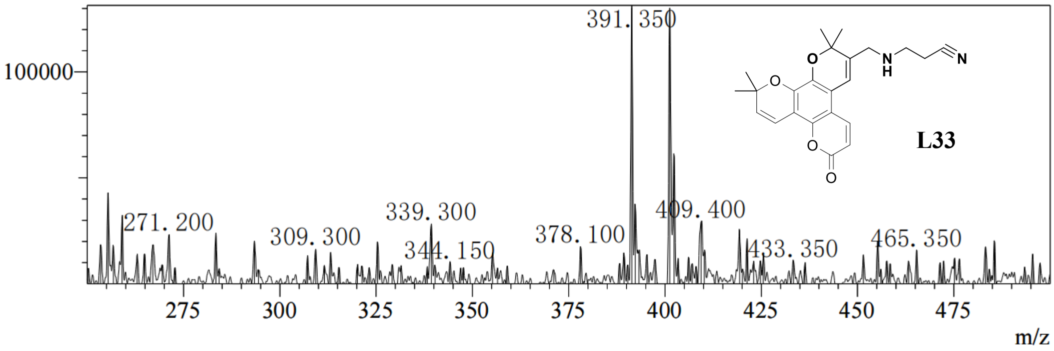

MS data of **L33**

5. HPLC data for tested compounds.

HPLC Report

Inj: 1-4 Inj. Vol : 1 uL
Cloumn: SHIMADZU 250 4.6 5.0 um
Detector: UVDualMode 254 nm / 214 nm
Oven: 30℃
Flow: 0.8 mL/min
Pump: A H2O 0%、B MeOH 30%、C ACN 70%

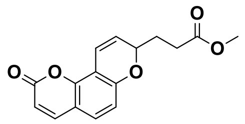

L1

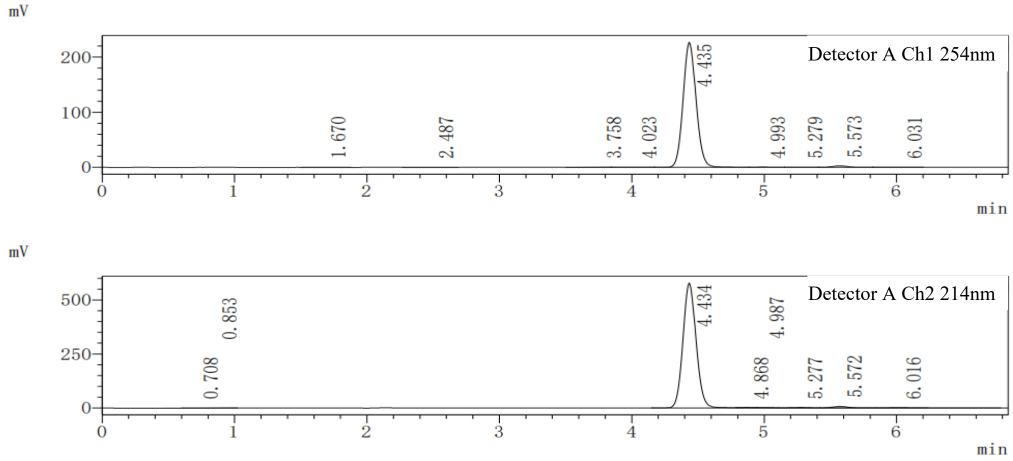

Ch1 254 nm

Peak No.	R.T	Area	Area%
1	1.670	1940	0.123
2	2.487	3386	0.214
3	3.758	1131	0.072
4	4.023	1762	0.111
5	4.435	1540811	97.399
6	4.993	4241	0.268
7	5.279	3333	0.211
8	5.573	21979	1.389
9	6.031	3382	0.214
Total		1581965	100.000

Ch2 214 nm

Peak No.	R.T	Area	Area%
1	0.708	1437	0.035
2	0.853	1508	0.037

3	4.434	3986278	97.788
4	4.868	6436	0.158
5	4.987	4372	0.107
6	5.277	10479	0.257
7	5.572	53617	1.315
8	6.016	12315	0.302
Total		4076442	100.000

HPLC Report

Inj: 1-4   Inj.Vol : 0.5 uL
Cloumn:SHIMADZU 250 4.6 5.0 um
Detector: UVDualMode 254 nm / 214 nm
Oven: 30℃
Flow: 0.8 mL/min
Pump: A H20 0%、B MeOH 30%、C ACN 70%

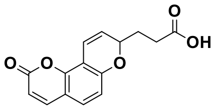

L2

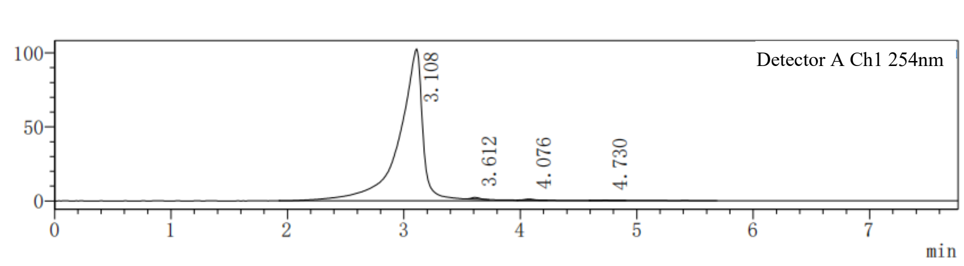

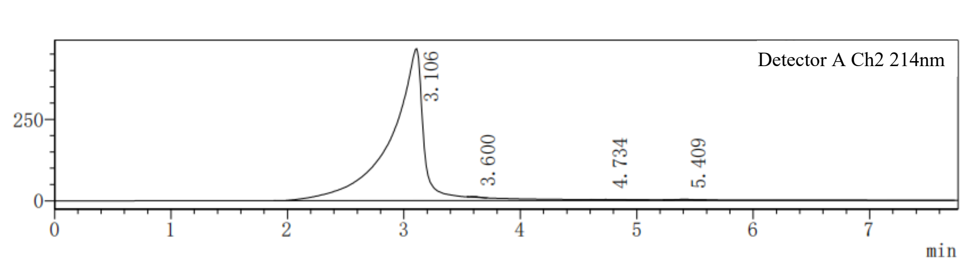

Ch1 254 nm

Peak No.	R.T	Area	Area%
1	3.108	1488097	99.297
2	3.612	5419	0.362
3	4.076	4042	0.270
4	4.730	1072	0.072
Total		1498629	100.000

Ch2 214 nm

Peak No.	R.T	Area	Area%
1	3.106	9663831	99.652
2	3.600	10950	0.113
3	4.734	7190	0.074
4	5.409	15561	0.160
Total		9697532	100.000

HPLC Report

Inj: 1-4 Inj.Vol : 0.5 uL
Cloumn:SHIMADZU 250 4.6 5.0 um
Detector: UVDualMode 254 nm / 214 nm
Oven: 30℃
Flow: 0.8 mL/min
Pump: A H2O 0%、B MeOH 30%、C ACN 70%

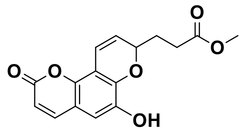

L3

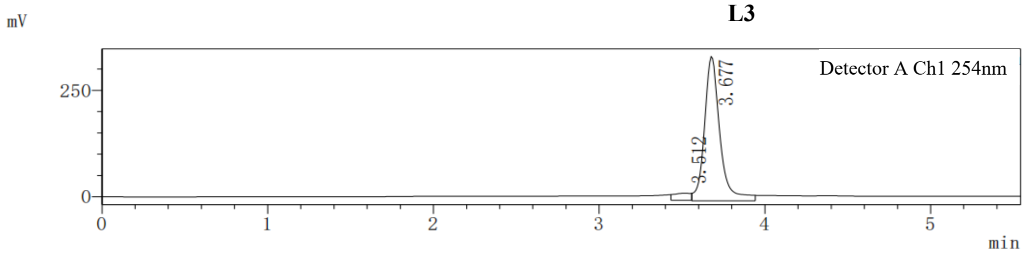

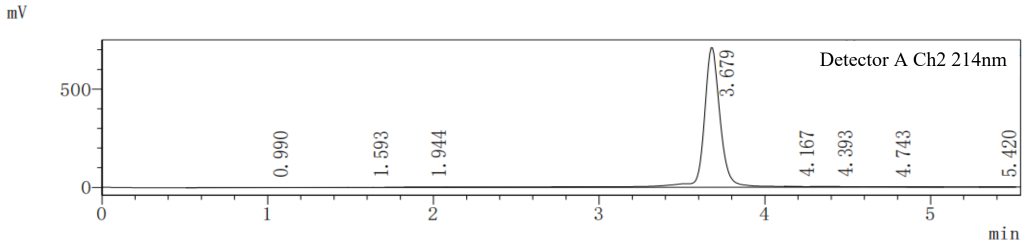

Ch1 254 nm

Peak No.	R.T	Area	Area%
1	3.512	116503	4.978
2	3.677	2224075	95.022
Total		2340578	100.000

Ch2 214 nm

Peak No.	R.T	Area	Area%
1	0.990	30447	0.584
2	1.593	2719	0.052
3	1.944	47010	0.902
4	3.679	5105598	97.946
5	4.169	1921	0.037
6	4.393	15722	0.302
7	4.743	7114	0.136
8	5.420	2153	0.041
Total		5212685	100.000

HPLC Report

Inj: 1-4   Inj.Vol : 0.5 uL
Cloumn:SHIMADZU 250 4.6 5.0 um
Detector: UVDualMode 254 nm / 210 nm
Oven: 30℃
Flow: 0.8 mL/min
Pump: A H2O 0%、B MeOH 30%、C ACN 70%

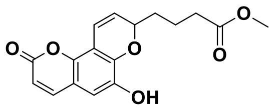

L4

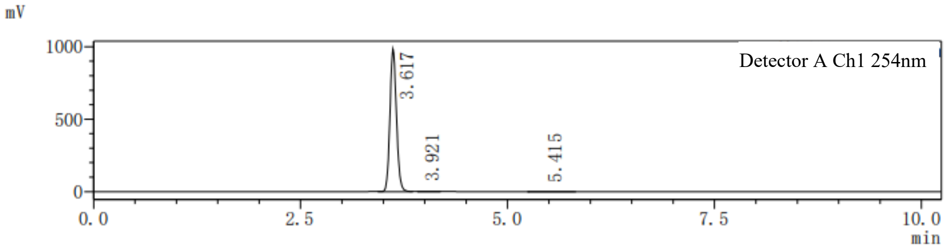

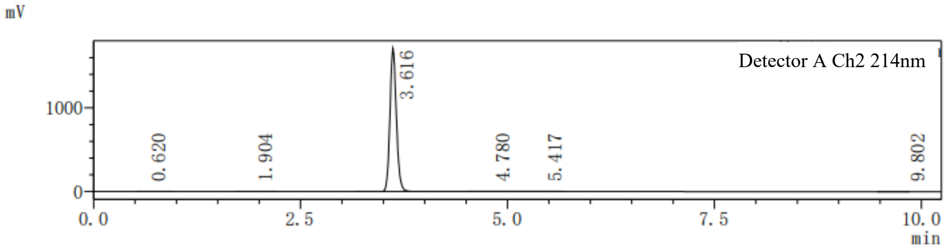

Ch1 254 nm

Peak No.	R.T	Area	Area%
1	3.617	5392820	99.850
2	3.921	7478	0.138
3	5.415	643	0.012
Total		5400941	100.000

Ch2 214 nm

Peak No.	R.T	Area	Area%
1	1.904	1403	0.015
2	3.616	1213	0.013
3	4.780	9231312	99.753
4	5.417	7996	0.086
5	9.802	11036	0.119
6	1.904	1193	0.013
Total		9254153	100.000

HPLC Report

Inj: 1-4   Inj.Vol   : 1 uL
Cloumn:SHIMADZU   250   4.6   5.0 um
Detector: UVDualMode 254 nm / 214 nm
Oven: 30℃
Flow: 0.8 mL/min
Pump: A H2O 0%、B MeOH 30%、C ACN 70%

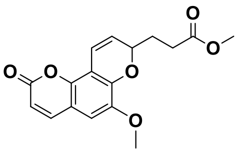

L5

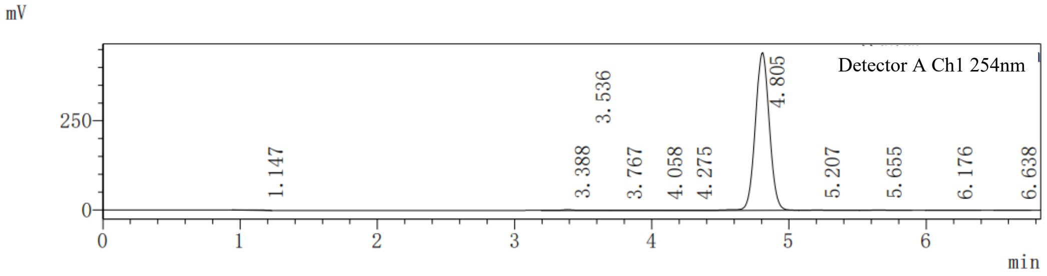

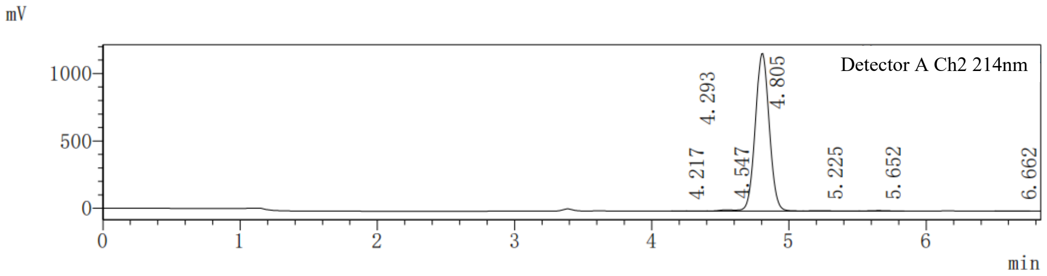

Ch1 254 nm

Peak No.	R.T	Area	Area%
1	1.147	8377	0.262
2	3.388	12508	0.391
3	3.536	4319	0.135
4	3.767	2651	0.083
5	4.058	2367	0.074
6	4.275	4103	0.128
7	4.805	3133151	97.986
8	5.207	13151	0.411
9	5.655	11026	0.345
10	6.176	4804	0.150
11	6.638	1088	0.034
Total		3197545	100.000

Ch2 214 nm

Peak No.	R.T	Area	Area%
1	4.217	8030	0.095
2	4.293	10200	0.121
3	4.547	72170	0.856
4	4.805	8270460	98.051
5	5.225	36751	0.436
6	5.652	35624	0.422
7	6.662	1590	0.019
Total		8434826	100.000

HPLC Report

Inj: 1-4   Inj.Vol   : 1 uL
Cloumn:SHIMADZU 250 4.6 5.0 um
Detector: UVDualMode 254 nm / 214 nm
Oven: 30℃
Flow: 0.8 mL/min
Pump: A H2O 0%、B MeOH 30%、C ACN 70%

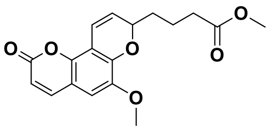

L6

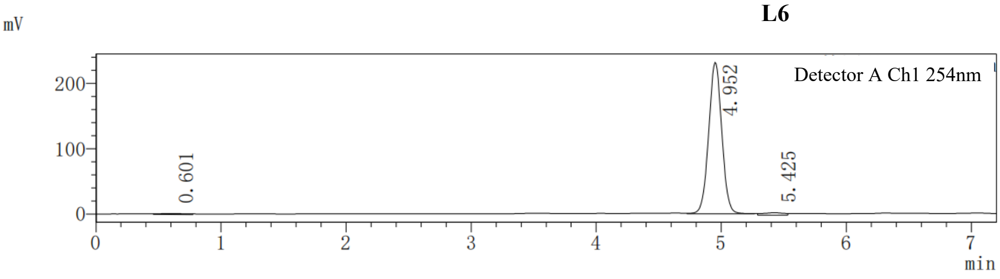

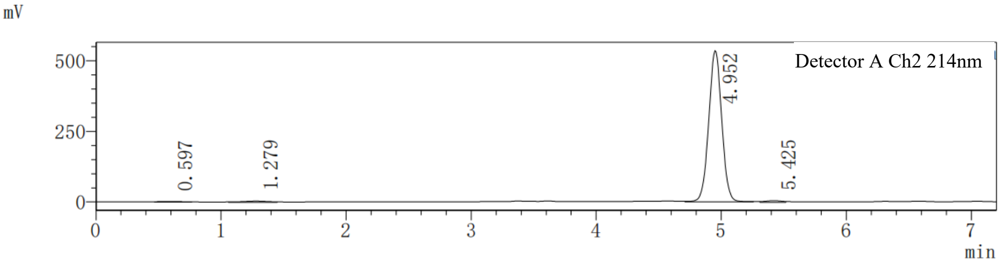

Ch1 254 nm

Peak No.	R.T	Area	Area%
1	0.601	23346	1.328
2	4.952	1687475	96.016
3	5.425	46677	2.656
Total		1757497	100.000

Ch2 214 nm

Peak No.	R.T	Area	Area%
1	0.597	25604	0.639
2	1.279	61120	1.525
3	4.952	3867891	96.538
4	5.425	51978	1.297
Total		4006593	100.000

HPLC Report

Inj: 1-4　 Inj.Vol　: 1 uL
Cloumn:SHIMADZU　250　4.6　5.0 um
Detector: UVDualMode 254 nm / 214 nm
Oven: 30℃
Flow: 0.8 mL/min
Pump: A H2O 0%、B MeOH 30%、C ACN 70%

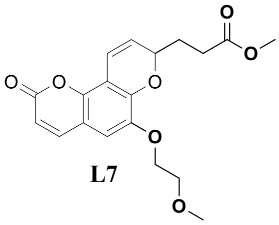

L7

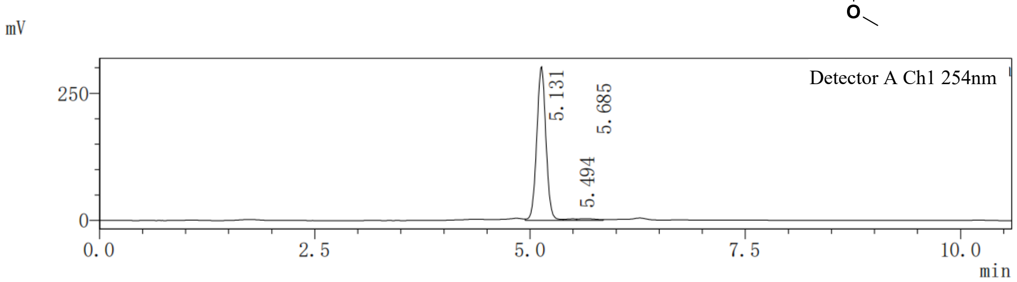

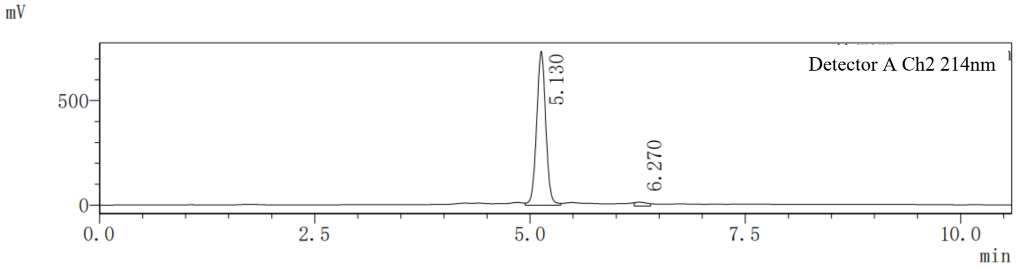

Ch1 254 nm

Peak No.	R.T	Area	Area%
1	5.131	2239807	96.514
2	5.494	27823	1.199
3	5.685	53085	2.287
Total		2320715	100.000

Ch2 214 nm

Peak No.	R.T	Area	Area%
1	5.130	5415217	96.767
2	6.270	180936	3.233
Total		5596153	100.000

HPLC Report

Inj: 1-4 Inj.Vol : 1 uL
Cloumn:SHIMADZU 250 4.6 5.0 um
Detector: UVDualMode 254 nm / 214 nm
Oven: 30℃
Flow: 0.8 mL/min
Pump: A H2O 0%、B MeOH 30%、C ACN 70%

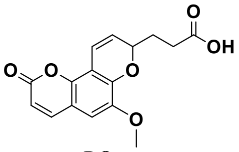

L8

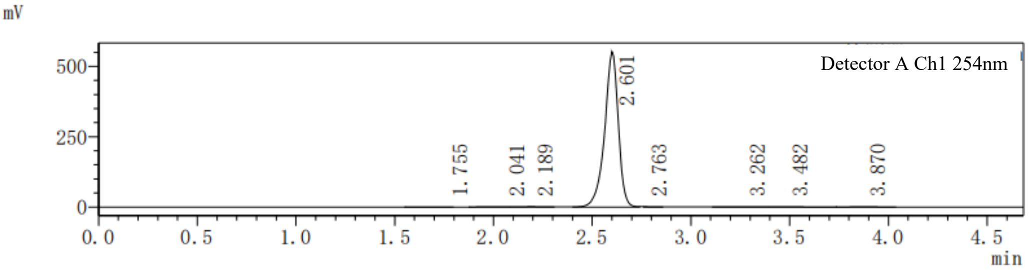

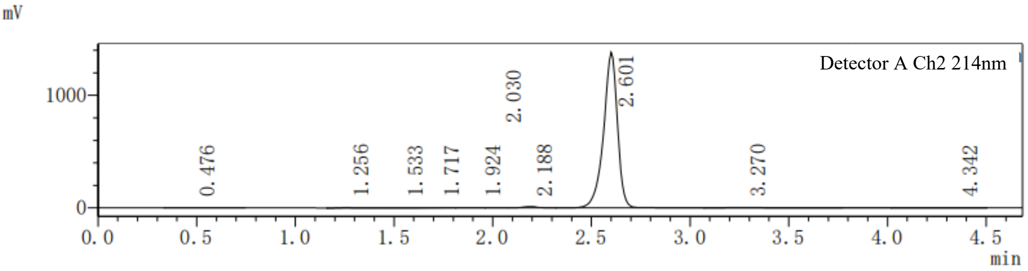

Ch1 254 nm

Peak No.	R.T	Area	Area%
1	1.755	1000	0.037
2	2.041	1482	0.055
3	2.189	7046	0.263
4	2.601	2657658	99.124
5	2.763	6436	0.240
6	3.262	2840	0.106
7	3.482	2590	0.097
8	3.870	2097	0.078
Total		2681150	100.000

Ch2 214 nm

Peak No.	R.T	Area	Area%
1	0.476	4008	0.058
2	1.256	22991	0.331

3	1.533	4926	0.071
4	1.717	8331	0.120
5	1.924	6512	0.094
6	2.030	5806	0.084
7	2.188	63095	0.908
8	2.601	6791382	97.764
9	3.270	38430	0.553
10	4.342	1204	0.017
Total		6946685	100.000

HPLC Report

Inj: 1-4　Inj.Vol　: 1 uL
Cloumn:SHIMADZU　250　4.6　5.0 um
Detector: UVDualMode 254 nm / 214 nm
Oven: 30℃
Flow: 0.8 mL/min
Pump: A H20 0%、B MeOH 30%、C ACN 70%

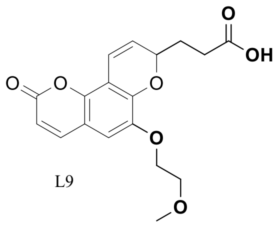

L9

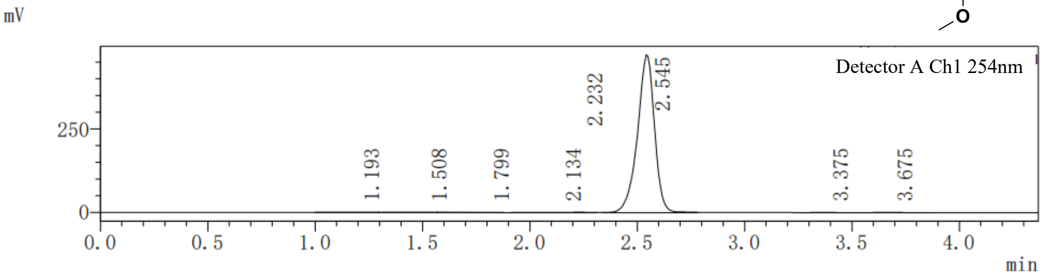

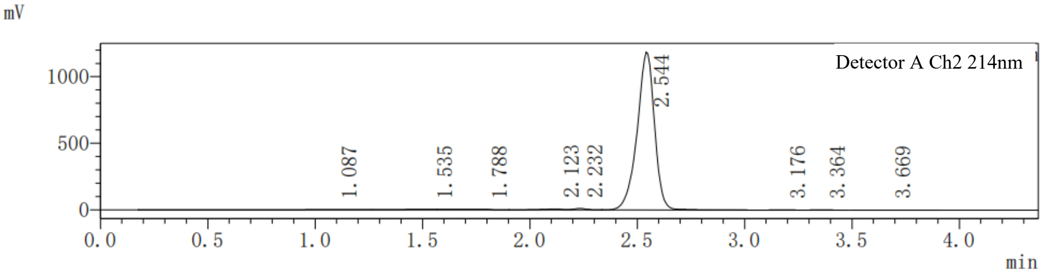

Ch1 254nm

Peak No.	R.T	Area	Area%
1	1.193	2173	0.082
2	1.508	3555	0.135
3	1.799	5747	0.218
4	2.134	5521	0.210
5	2.232	5674	0.215
6	2.545	2609529	99.042
7	3.375	1022	0.039
8	3.675	1546	0.059
Total		2634768	100.000

Ch2 214nm

Peak No.	R.T	Area	Area%
1	1.087	69769	1.000
2	1.535	63427	0.909

3	1.788	73473	1.053
4	2.123	52533	0.753
5	2.232	48370	0.693
6	2.544	6667212	95.526
7	3.176	1277	0.018
8	3.364	1954	0.028
9	3.669	1441	0.021
Total		6979455	100.000

HPLC Report

Inj: 1-4   Inj.Vol   : 1 uL
Cloumn:SHIMADZU 250 4.6 5.0 um
Detector: UVDualMode 254 nm / 214 nm
Oven: 30℃
Flow: 0.8 mL/min
Pump: A H2O 0%、B MeOH 30%、C ACN 70%

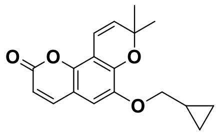

L10

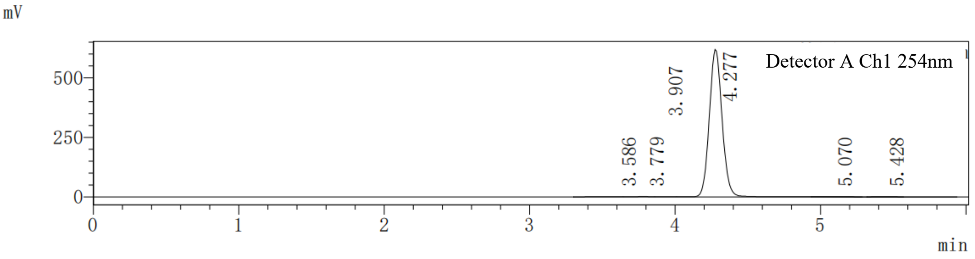

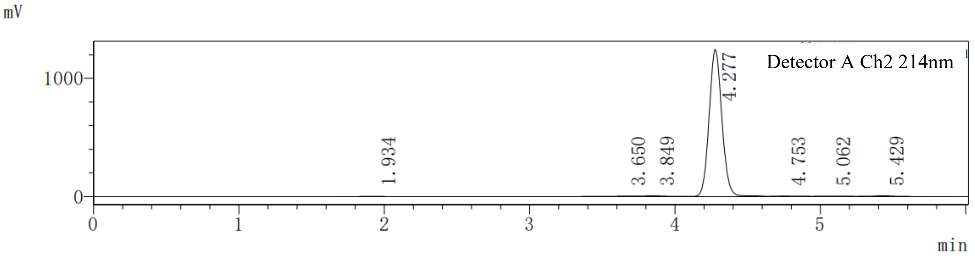

Ch1 254nm

Peak No.	R.T	Area	Area%
1	3.586	4432	0.117
2	3.779	12134	0.320
3	3.907	5851	0.154
4	4.277	3764712	99.292
5	5.070	2861	0.075
6	5.428	1551	0.041
Total		3791541	100.000

Ch2 214nm

Peak No.	R.T	Area	Area%
1	1.934	6772	0.087
2	3.650	35513	0.455
3	3.849	67913	0.869
4	4.277	7635421	97.756

5	4.753	11000	0.141
6	5.062	3598	0.046
7	5.429	50441	0.646
Total		7810658	100.000

HPLC Report

Inj: 1-4 Inj.Vol : 1 uL
Cloumn:SHIMADZU 250 4.6 5.0 um
Detector: UVDualMode 254 nm / 214 nm
Oven: 30℃
Flow: 0.8 mL/min
Pump: A H2O 0%、B MeOH 30%、C ACN 70%

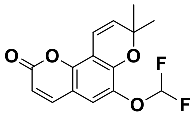

L11

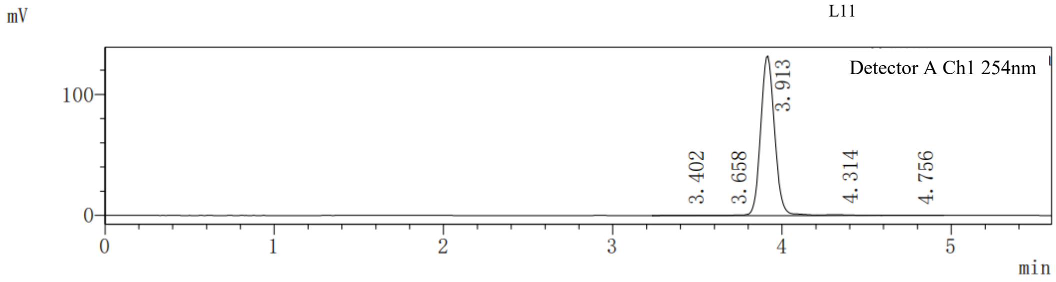

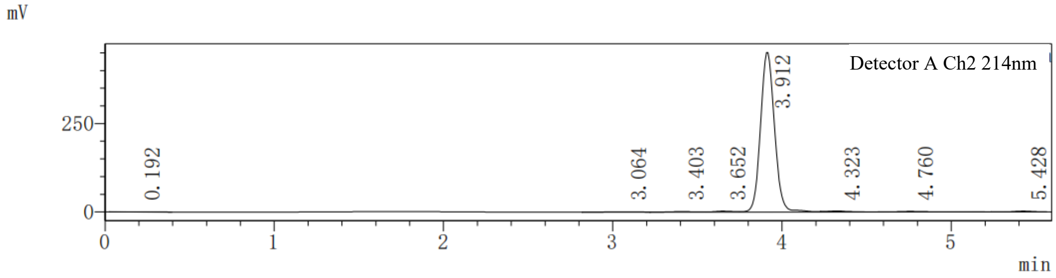

Ch1 254nm

Peak No.	R.T	Area	Area%
1	3.402	1586	0.208
2	3.658	2078	0.273
3	3.913	749107	98.426
4	4.314	7119	0.953
5	4.756	1195	0.157
Total		761085	100.000

Ch2 214nm

Peak No.	R.T	Area	Area%
1	0.192	1957	0.073
2	3.064	2091	0.078
3	3.403	10702	0.400
4	3.652	19199	0.718
5	3.912	2590040	96.912

6	4.323	13394	0.501
7	4.760	17416	0.652
8	5.428	17780	0.665
Total		2672579	100.000

HPLC Report

Inj: 1-4 Inj.Vol : 1 uL
Cloumn:SHIMADZU 250 4.6 5.0 um
Detector: UVDualMode 254 nm / 214 nm
Oven: 30℃
Flow: 0.8 mL/min
Pump: A H2O 0%、B MeOH 30%、C ACN 70%

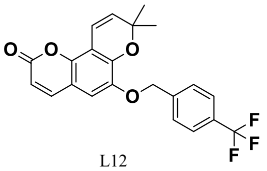

L12

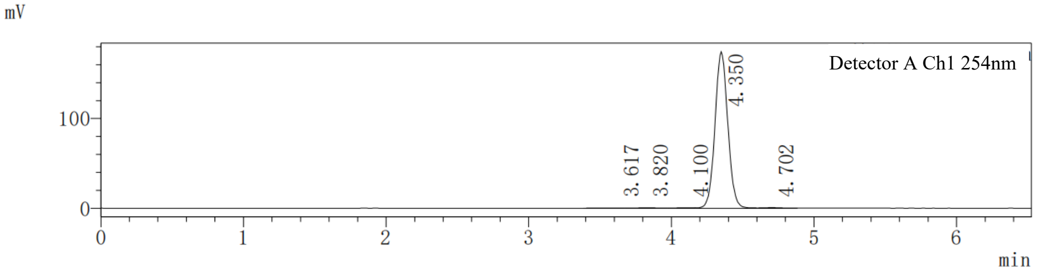

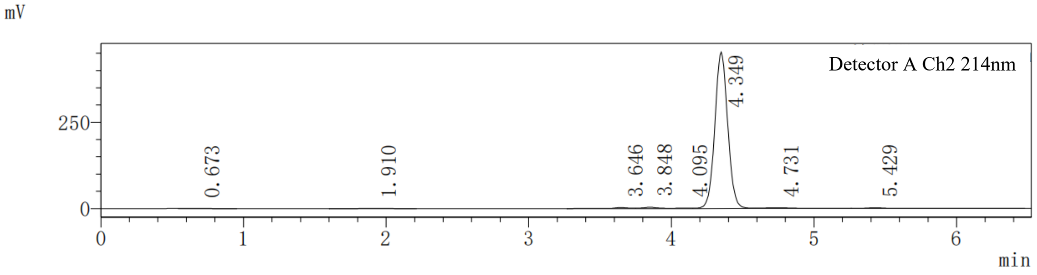

Ch1 254nm

Peak No.	R.T	Area	Area%
1	3.617	1586	0.165
2	3.820	5602	0.505
3	4.100	4882	0.440
4	4.350	1090211	98.315
5	4.702	6374	0.575
Total		1108895	100.000

Ch2 214nm

Peak No.	R.T	Area	Area%
1	0.673	1621	0.055
2	1.910	3297	0.111
3	3.646	23472	0.793
4	3.848	28497	0.963
5	4.095	8811	0.298

6	4.349	2840959	95.972
7	4.731	34176	1.155
8	5.429	19362	0.665
Total		2960196	100.000

HPLC Report

Inj: 1-4   Inj.Vol : 1 uL
Cloumn:SHIMADZU 250 4.6 5.0 um
Detector: UVDualMode 254 nm / 214 nm
Oven: 30℃
Flow: 0.8 mL/min
Pump: A H2O 0%、B MeOH 30%、C ACN 70%

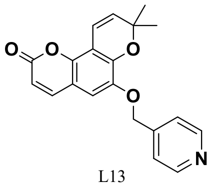

L13

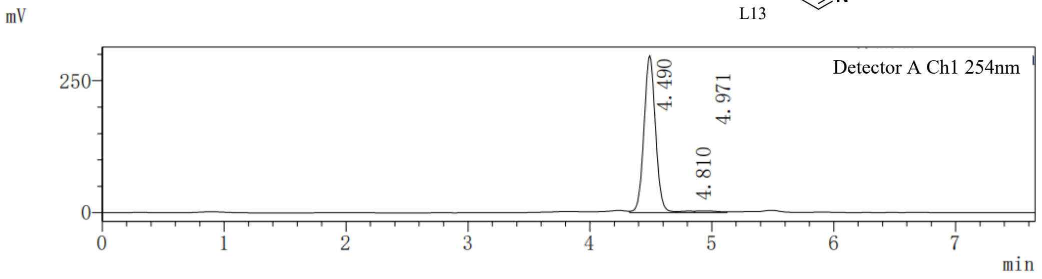

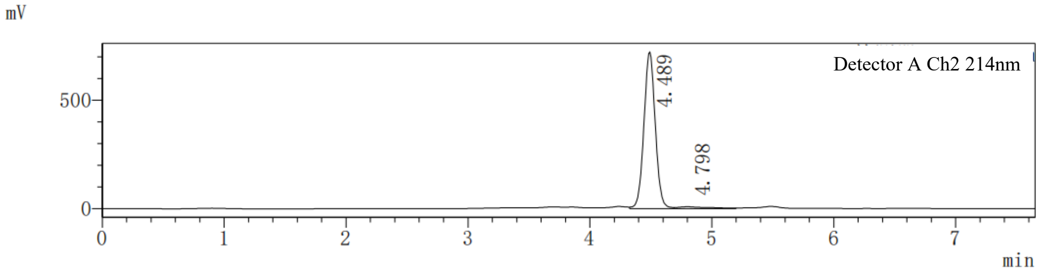

Ch1 254nm

Peak No.	R.T	Area	Area%
1	4.490	1957558	96.500
2	4.810	26238	1.293
3	4.971	44763	2.207
Total		2028559	100.000

L14

Ch2 214nm

Peak No.	R.T	Area	Area%
1	4.489	4679524	96.377
2	4.798	175916	3.623
Total		4855440	100.000

HPLC Report

Inj: 1-4 Inj.Vol : 0.1 uL
Cloumn:SHIMADZU 250 4.6 5.0 um
Detector: UVDualMode 254 nm / 214 nm
Oven: 30℃
Flow: 0.8 mL/min
Pump: A H2O 0%、B MeOH 30%、C ACN 70%

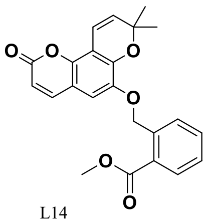

L14

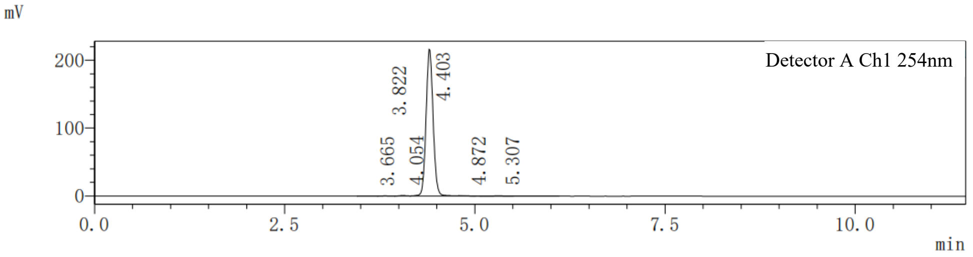

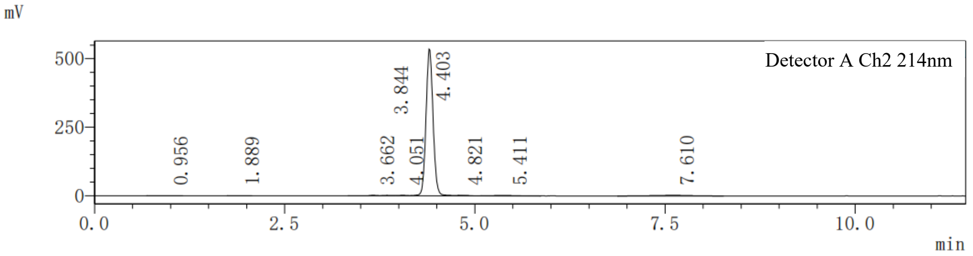

Ch1 254nm

Peak No.	R.T	Area	Area%
1	3.665	1541	0.109
2	3.822	3845	0.271
3	4.054	7783	0.548
4	4.403	1400208	98.672
5	4.872	1355	0.095
6	5.307	4318	0.304
Total		1419050	100.000

Ch2 214nm

Peak No.	R.T	Area	Area%
1	0.956	2898	0.080
2	1.889	1718	0.047
3	3.662	17522	0.482
4	3.844	17110	0.471

5	4.051	18177	0.500
6	4.403	3478425	95.735
7	4.821	2049	0.056
8	5.411	20014	0.551
9	7.610	75491	2.078
Total		3633405	100.000

HPLC Report

Inj: 1-4   Inj.Vol : 1 uL
Cloumn:SHIMADZU 250 4.6 5.0 um
Detector: UVDualMode 254 nm / 214 nm
Oven: 30℃
Flow: 0.8 mL/min
Pump: A H2O 0%、B MeOH 30%、C ACN 70%

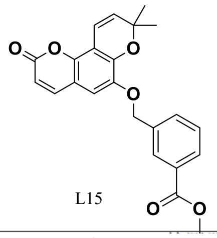

L15

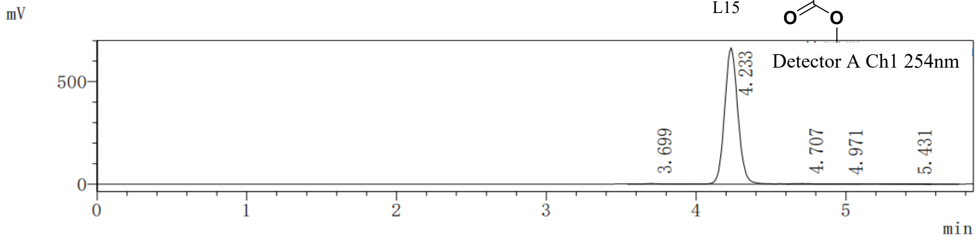

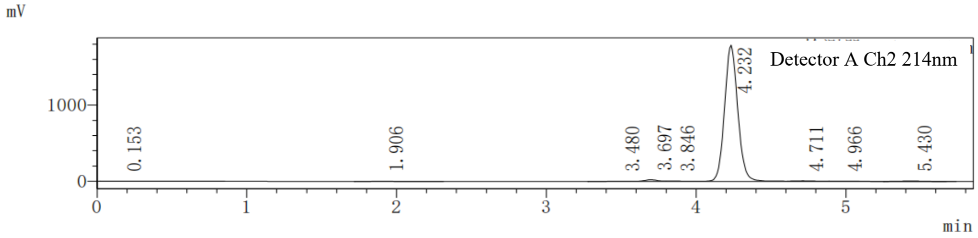

Ch1 254nm

Peak No.	R.T	Area	Area%
1	3.699	23390	0.565
2	4.233	4103710	99.059
3	4.707	10033	0.242
4	4.971	4234	0.102
5	5.431	1332	0.032
Total		4142699	100.000

Ch2 214nm

Peak No.	R.T	Area	Area%
1	0.153	10897	0.096
2	1.906	5129	0.045
3	3.480	10652	0.094
4	3.697	135749	1.202
5	3.846	38726	0.343
6	4.232	11007613	97.449
7	4.711	29424	0.260

8	4.966	11014	0.098
9	5.430	46562	0.412
Total		11295766	100.000

HPLC Report

Inj: 1-4   Inj.Vol : 1 uL
Cloumn:SHIMADZU 250 4.6 5.0 um
Detector: UVDualMode 254 nm / 214 nm
Oven: 30℃
Flow: 0.8 mL/min
Pump: A H2O 0%、B MeOH 30%、C ACN 70%

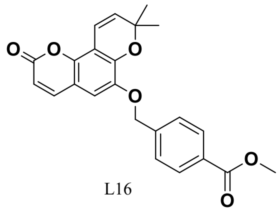

L16

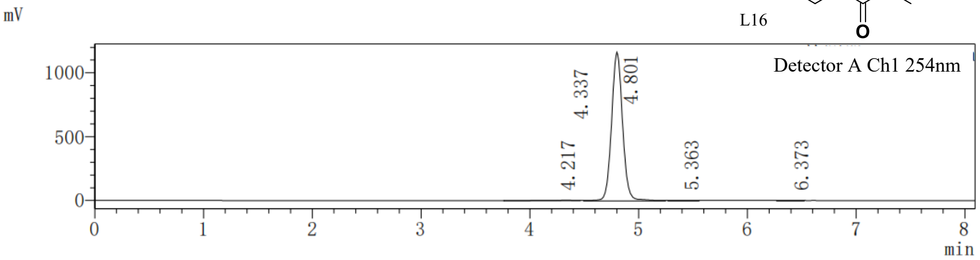

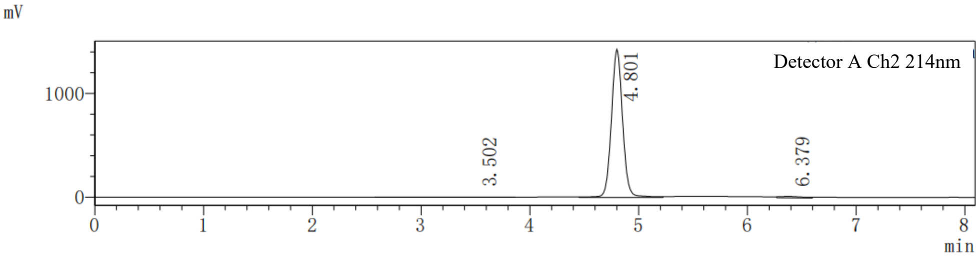

Ch1 254nm

Peak No.	R.T	Area	Area%
1	4.217	17450	0.208
2	4.337	24834	0.295
3	4.801	8222397	97.813
4	5.363	83122	0.989
5	6.373	58478	0.696
Total		8406281	100.000

Ch2 214nm

Peak No.	R.T	Area	Area%
1	3.502	54706	0.522
2	4.801	10213884	97.549
3	6.379	201905	1.928
Total		10470495	100.000

HPLC Report

Inj: 1-4   Inj.Vol : 1 uL
Cloumn:SHIMADZU 250 4.6 5.0 um
Detector: UVDualMode 254 nm / 214 nm
Oven: 30℃
Flow: 0.8 mL/min
Pump: A H2O 0%、B MeOH 30%、C ACN 70%

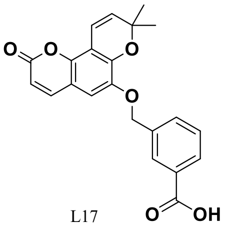

L17

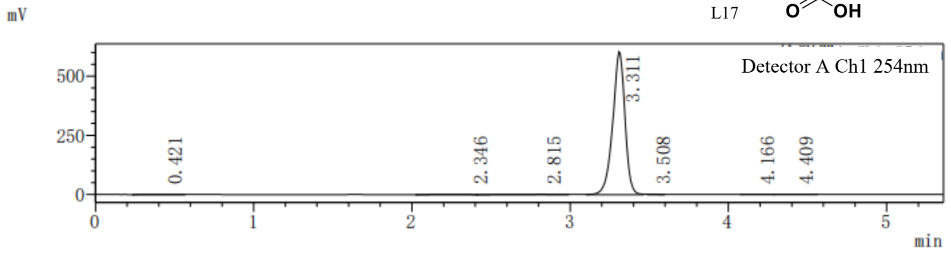

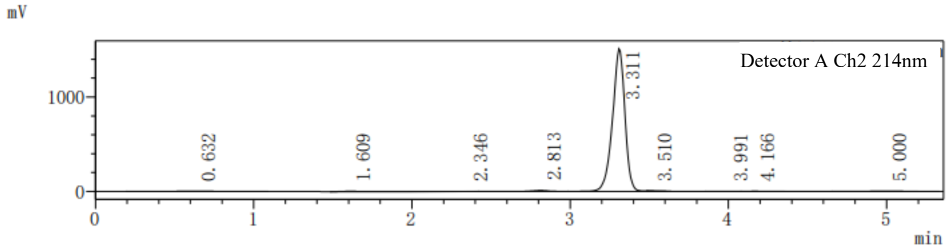

Ch1 254nm

Peak No.	R.T	Area	Area%
1	0.421	2195	0.067
2	2.346	3306	0.101
3	2.815	15434	0.471
4	3.311	3244616	98.933
5	3.508	10948	0.334
6	4.166	1290	0.039
7	4.409	1822	0.056
Total		3279612	100.000

Ch2 214nm

Peak No.	R.T	Area	Area%
1	0.632	38490	0.450
2	1.609	32539	0.380
3	2.346	13737	0.161
4	2.813	94631	1.106

5	3.311	8338689	97.484
6	3.510	2671	0.031
7	3.991	1650	0.019
8	4.166	3770	0.044
9	5.000	27707	0.324
Total		8553885	100.000

HPLC Report

Inj: 1-4 Inj.Vol : 1 uL
Cloumn:SHIMADZU 250 4.6 5.0 um
Detector: UVDualMode 254 nm / 214 nm
Oven: 30℃
Flow: 0.8 mL/min
Pump: A H2O 0%、B MeOH 30%、C ACN 70%

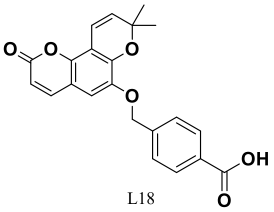

L18

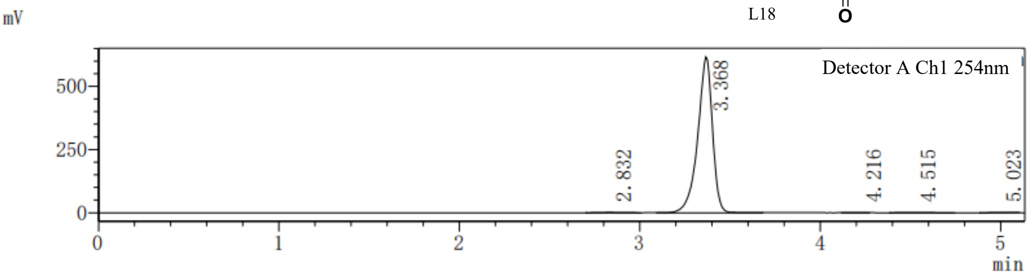

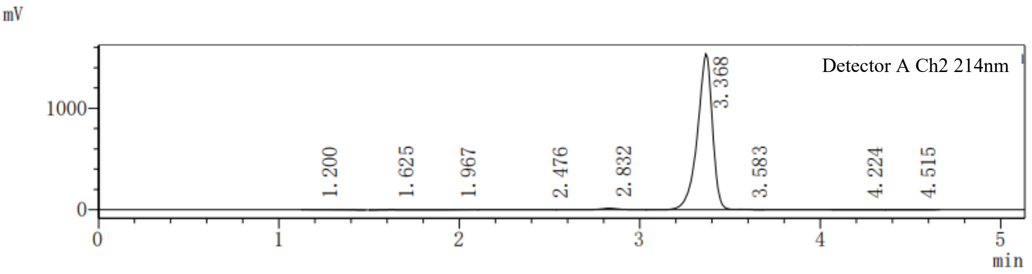

Ch1 254nm

Peak No.	R.T	Area	Area%
1	2.832	8687	0.247
2	3.368	3503846	99.594
3	4.216	2359	0.067
4	4.515	1725	0.049
5	5.023	1513	0.043
Total		3518130	100.000

Ch2 214nm

Peak No.	R.T	Area	Area%
1	1.200	5589	0.062
2	1.625	28739	0.318
3	1.967	6815	0.075
4	2.476	13256	0.147
5	2.832	109954	1.216

6	3.368	8871102	98.087
7	3.583	2511	0.028
8	4.224	2363	0.026
9	4.515	3832	0.042
Total		9044162	100.000

HPLC Report

Inj: 1-4　Inj.Vol　: 1 uL
Cloumn:SHIMADZU　250　4.6　5.0 um
Detector: UVDualMode 254 nm / 214 nm
Oven: 30℃
Flow: 0.8 mL/min
Pump: A H2O 0%、B MeOH 30%、C ACN 70%

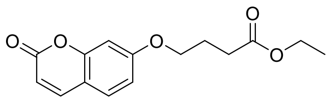

L19

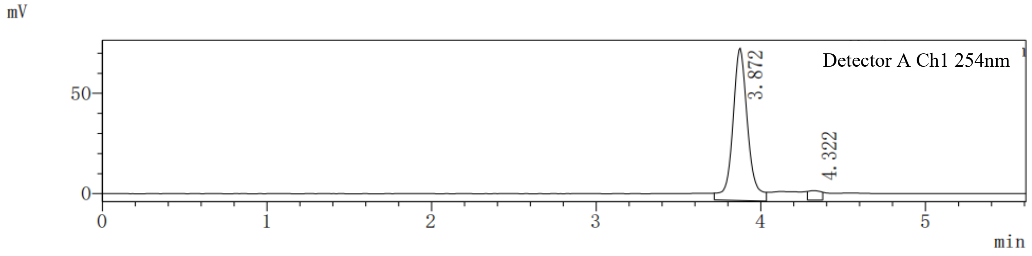

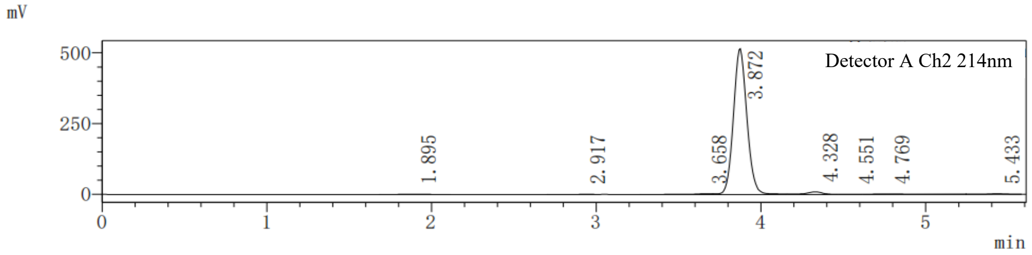

Ch1 254nm

Peak No.	R.T	Area	Area%
1	3.872	477643	95.129
2	4.322	24455	4.871
Total		502098	100.000

Ch2 214nm

Peak No.	R.T	Area	Area%
1	1.895	3107	0.101
2	2.917	1220	0.040
3	3.658	17282	0.564
4	3.872	2932435	95.775
5	4.328	64418	2.104
6	4.551	5555	0.181
7	4.769	21374	0.698
8	5.433	16412	0.536
Total		3061803	100.000

HPLC Report

Inj: 1-4　Inj.Vol　: 0.5 uL
Cloumn:SHIMADZU　250　4.6　5.0 um
Detector: UVDualMode 254 nm / 214 nm
Oven: 30℃
Flow: 0.8 mL/min
Pump: A H2O 0%、B MeOH 30%、C ACN 70%

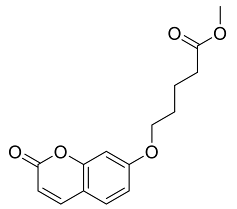

L20

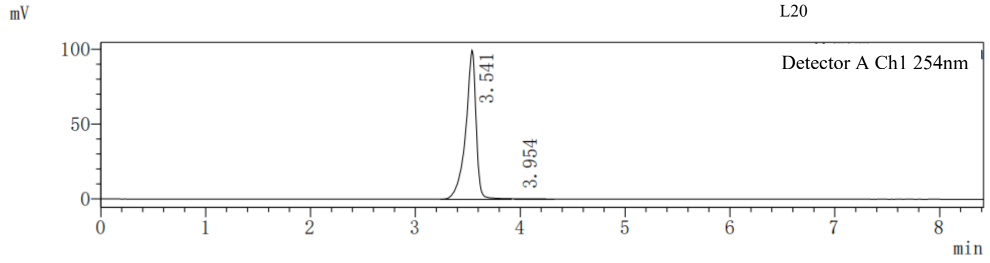

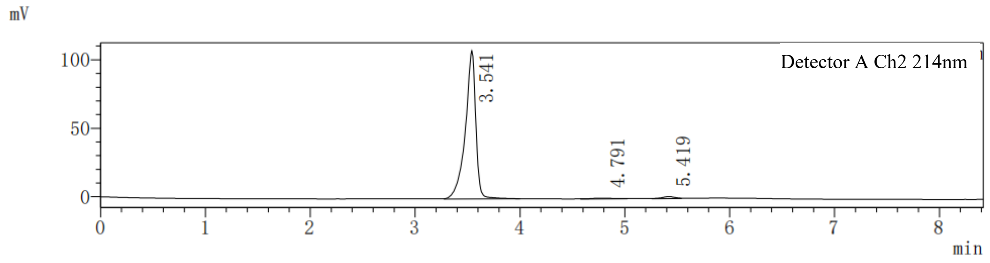

Ch1 254nm

Peak No.	R.T	Area	Area%
1	3.541	690332	98.966
2	3.954	7213	1.034
Total		697545	100.000

Ch2 214nm

Peak No.	R.T	Area	Area%
1	3.541	751251	97.027
2	4.791	9308	1.202
3	5.419	13709	1.771
Total		774268	100.000

HPLC Report

Inj: 1-4　Inj.Vol　: 0.1 uL
Cloumn:SHIMADZU　250　4.6　5.0 um
Detector: UVDualMode 254 nm / 214 nm
Oven: 30℃
Flow: 0.8 mL/min
Pump: A H2O 0%、B MeOH 30%、C ACN 70%

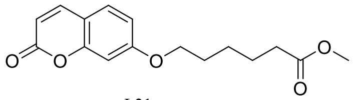

L21

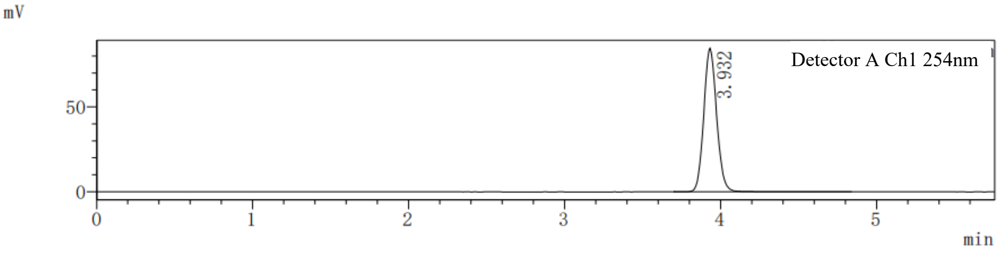

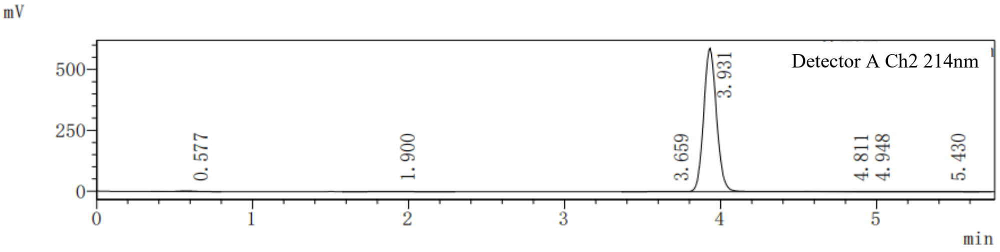

Ch1 254nm

Peak No.	R.T	Area	Area%
1	3.932	487398	100.000
Total		487398	100.000

Ch2 214nm

Peak No.	R.T	Area	Area%
1	0.577	22051	0.642
2	1.900	5221	0.152
3	3.659	4503	0.131
4	3.931	3380888	98.477
5	4.811	7860	0.229
6	4.948	7659	0.223
7	5.430	5010	0.146
Total		3433191	100.000

HPLC Report

Inj: 1-4 Inj.Vol : 0.5 uL
Cloumn:SHIMADZU 250 4.6 5.0 um
Detector: UVDualMode 254 nm / 214 nm
Oven: 30℃
Flow: 0.8 mL/min
Pump: A H20 0%、B MeOH 30%、C ACN 70%

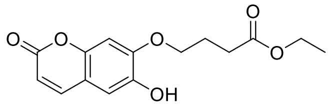

L22

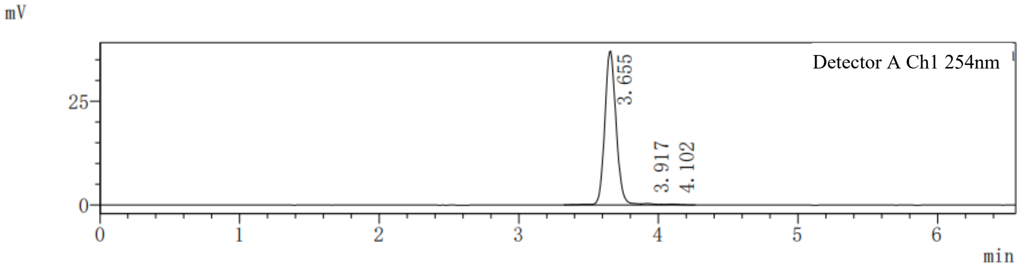

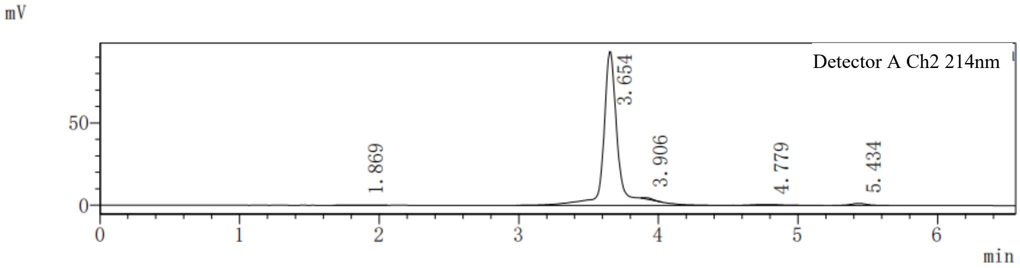

Ch1 254nm

Peak No.	R.T	Area	Area%
1	3.655	210322	98.110
2	3.917	2290	1.068
3	4.102	1762	0.822
Total		214374	100.000

Ch2 214nm

Peak No.	R.T	Area	Area%
1	1.869	1035	0.150
2	3.654	666114	96.743
3	3.906	3166	0.460
4	4.779	8520	1.237
5	5.434	9707	1.410
Total		688543	100.000

HPLC Report

Inj: 1-4   Inj.Vol   : 1 uL
Cloumn:SHIMADZU 250 4.6 5.0 um
Detector: UVDualMode 254 nm / 214 nm
Oven: 30℃
Flow: 0.8 mL/min
Pump: A H20 0%、B MeOH 30%、C ACN 70%

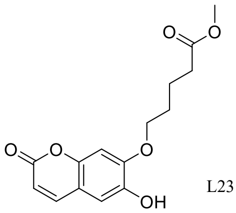

L23

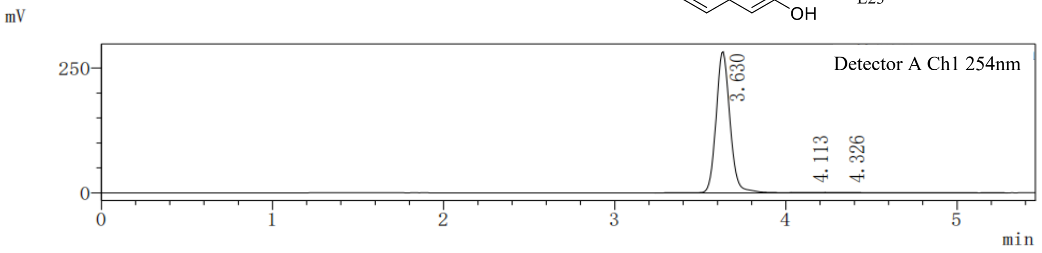

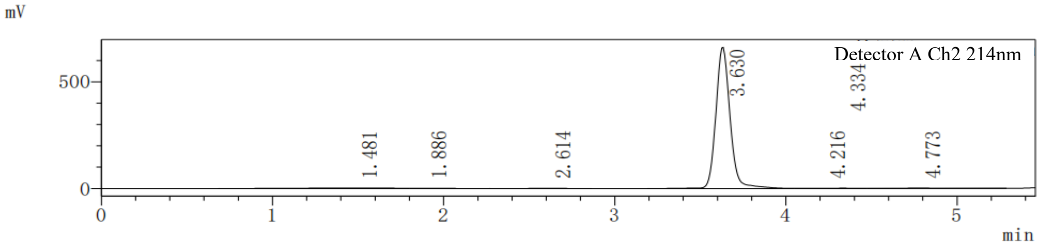

Ch1 254nm

Peak No.	R.T	Area	Area%
1	3.630	1598436	99.844
2	4.113	1359	0.085
3	4.326	1142	0.071
Total		1600938	100.000

Ch2 214nm

Peak No.	R.T	Area	Area%
1	1.481	74031	1.878
2	1.886	11189	0.284
3	2.614	2421	0.061
4	3.630	3813650	96.757
5	4.216	8191	0.208
6	4.334	9618	0.244
7	4.773	22364	0.567
Total		3941464	100.000

HPLC Report

Inj: 1-4　Inj.Vol　: 1 uL
Cloumn:SHIMADZU　250　4.6　5.0 um
Detector: UVDualMode 254 nm / 214 nm
Oven: 30℃
Flow: 0.8 mL/min
Pump: A H2O 0%、B MeOH 30%、C ACN 70%

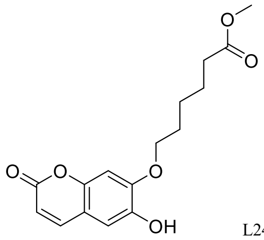

L24

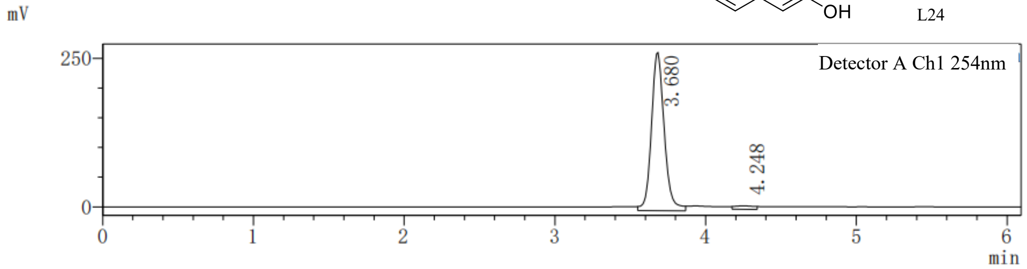

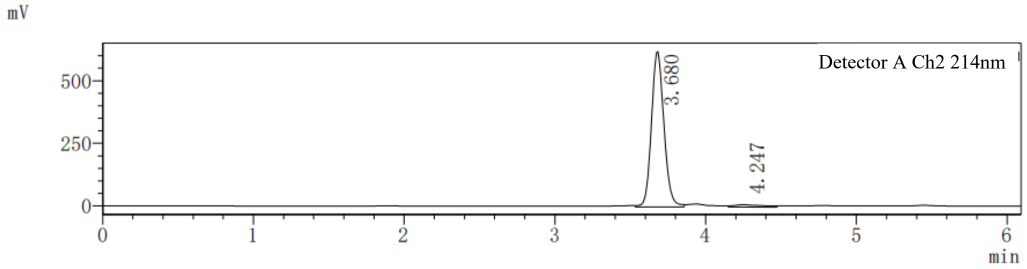

Ch1 254nm

Peak No.	R.T	Area	Area%
1	3.680	1579027	96.773
2	4.248	52658	3.227
Total		1631685	100.000

Ch2 214nm

Peak No.	R.T	Area	Area%
1	3.680	3562271	96.607
2	4.247	125106	3.393
Total		3687377	100.000

HPLC Report

Inj: 1-4   Inj.Vol : 0.5 uL
Cloumn:SHIMADZU 250 4.6 5.0 um
Detector: UVDualMode 254 nm / 214 nm
Oven: 30℃
Flow: 0.8 mL/min
Pump: A H2O 0%、B MeOH 30%、C ACN 70%

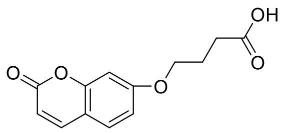

L25

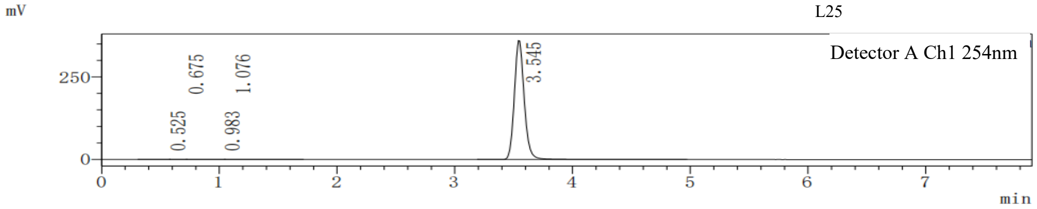

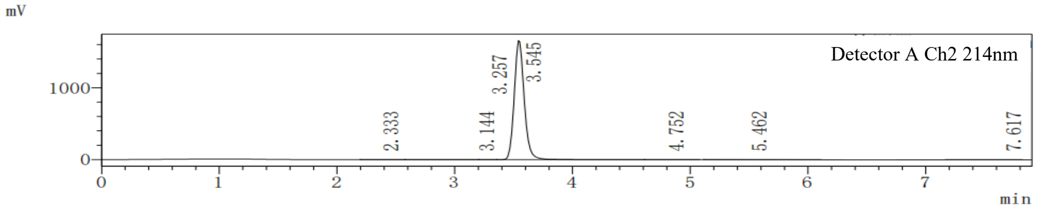

Ch1 254nm

Peak No.	R.T	Area	Area%
1	0.525	1943	0.092
2	0.675	1736	0.082
3	0.983	5846	0.276
4	1.076	6822	0.322
5	3.545	2101097	99.228
Total		2117444	100.000

Ch2 214nm

Peak No.	R.T	Area	Area%
1	2.333	1184	0.012
2	3.144	28901	0.290
3	3.257	13223	0.133
4	3.545	9788384	98.368
5	4.752	10109	0.102
6	5.462	40119	0.403
7	7.617	68865	0.692
Total		9950785	100.000

HPLC Report

Inj: 1-4 Inj.Vol : 0.5 uL
Cloumn:SHIMADZU 250 4.6 5.0 um
Detector: UVDualMode 254 nm / 214 nm
Oven: 30℃
Flow: 0.8 mL/min
Pump: A H2O 0%、B MeOH 30%、C ACN 70%

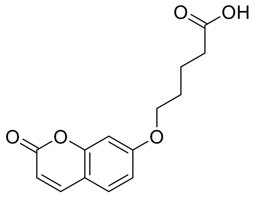

L26

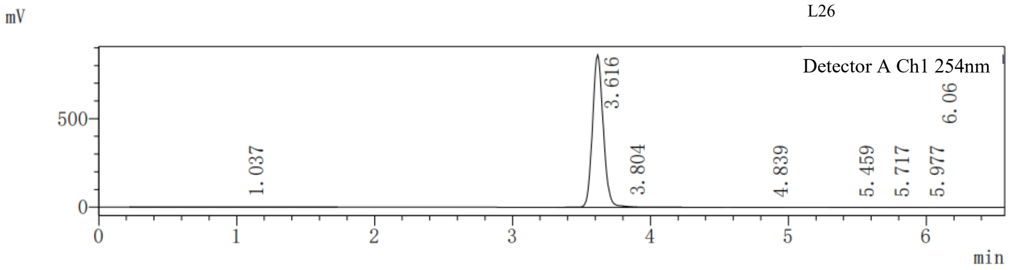

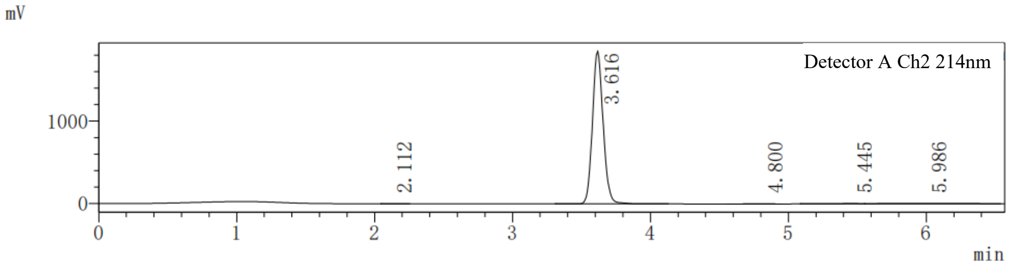

Ch1 254nm

Peak No.	R.T	Area	Area%
1	1.037	35710	0.759
2	3.616	4648983	98.768
3	3.804	14523	0.309
4	4.839	952	0.020
5	5.459	1346	0.029
6	5.717	1154	0.025
7	5.977	2174	0.046
8	6.063	2108	0.045
Total		4706951	100.000

Ch2 214nm

Peak No.	R.T	Area	Area%
1	2.112	1402	0.014
2	3.616	9941085	98.295

3	4.800	5694	0.056
4	5.445	36732	0.363
5	5.986	128574	1.271
Total		10113487	100.000

HPLC Report

Inj: 1-4 Inj.Vol : 1 uL
Cloumn:SHIMADZU 250 4.6 5.0 um
Detector: UVDualMode 254 nm / 214 nm
Oven: 30℃
Flow: 0.8 mL/min
Pump: A H20 0%、B MeOH 30%、C ACN 70%

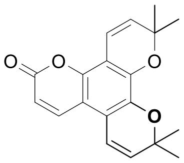

L27

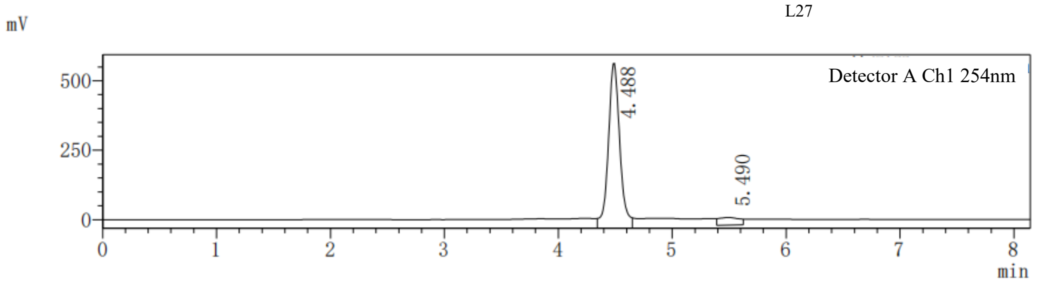

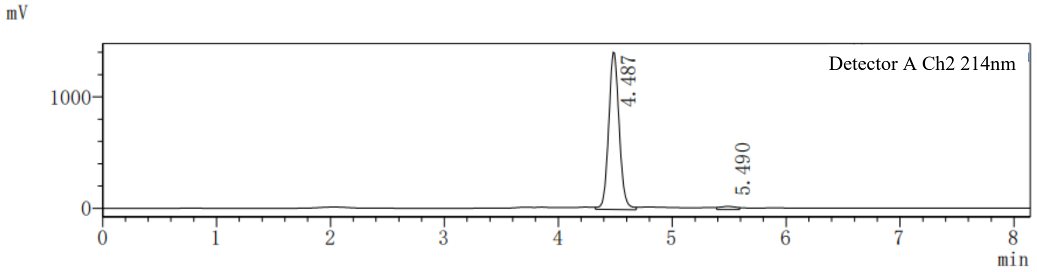

Ch1 254nm

Peak No.	R.T	Area	Area%
1	4.488	6733747	95.081
2	5.490	348352	4.919
Total		7082099	100.000

Ch2 214nm

Peak No.	R.T	Area	Area%
1	4.487	9024064	97.093
2	5.490	270187	2.907
Total		9294252	100.000

HPLC Report

Inj: 1-4   Inj.Vol   : 1 uL
Cloumn:SHIMADZU   250   4.6   5.0 um
Detector: UVDualMode 254 nm / 214 nm
Oven: 30℃
Flow: 0.8 mL/min
Pump: A H2O 0%、B MeOH 30%、C ACN 70%

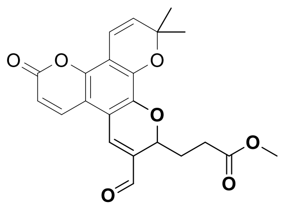

L28

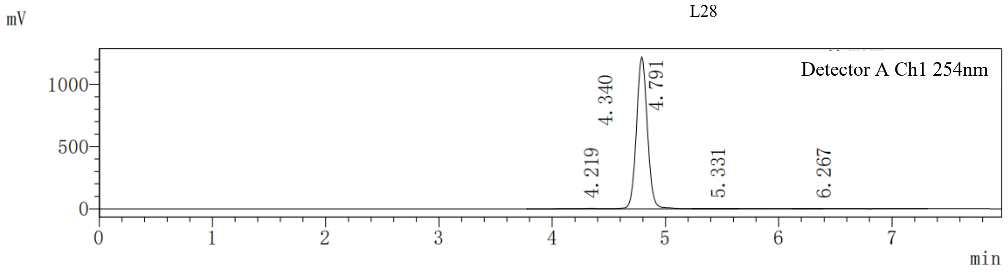

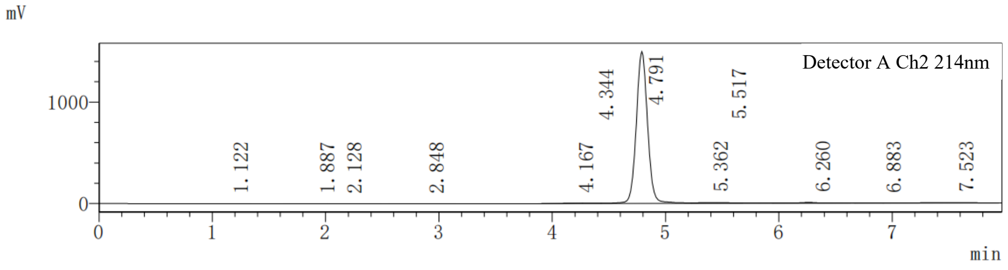

Ch1 254nm

Peak No.	R.T	Area	Area%
1	4.219	16891	0.205
2	4.340	24848	0.301
3	4.791	8195316	99.393
4	5.331	6056	0.073
5	6.267	2234	0.027
Total		8245345	100.000

Ch2 214nm

Peak No.	R.T	Area	Area%
1	1.122	1292	0.012
2	1.887	1791	0.016
3	2.128	2834	0.026
4	2.848	3300	0.030
5	4.167	38811	0.354

6	4.344	29255	0.267
7	4.791	10690409	97.447
8	5.362	14018	0.128
9	5.517	11566	0.105
10	6.260	53491	0.488
11	6.883	2528	0.023
12	7.523	121179	1.105
Total		10970473	100.000

HPLC Report

Inj: 1-4　Inj.Vol　: 1 uL
Cloumn:SHIMADZU　250　4.6　5.0 um
Detector: UVDualMode 254 nm / 214 nm
Oven: 30℃
Flow: 0.8 mL/min
Pump: A H2O 0%、B MeOH 30%、C ACN 70%

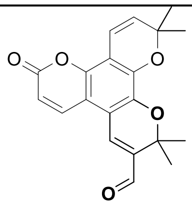

L29

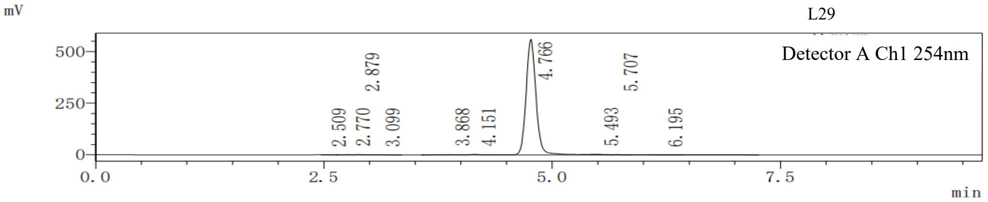

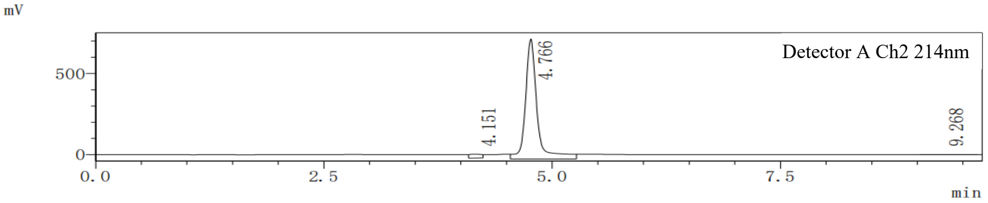

Ch1 254nm

Peak No.	R.T	Area	Area%
1	2.509	1527	0.035
2	2.770	7682	0.178
3	2.879	8875	0.206
4	3.099	1792	0.041
5	3.868	11703	0.271
6	4.151	17028	0.394
7	4.766	4258588	98.618
8	5.493	8010	0.185
9	5.707	1482	0.034
10	6.195	1598	0.037
Total		4318285	100.000

Ch2 214nm

Peak No.	R.T	Area	Area%
1	4.151	217041	3.199
2	4.766	6565783	96.776
3	9.268	1710	0.025
Total		6784535	100.000

HPLC Report

Inj: 1-4　Inj.Vol　: 1 uL
Cloumn:SHIMADZU　250　4.6　5.0 um
Detector: UVDualMode 254 nm / 214 nm
Oven: 30℃
Flow: 0.8 mL/min
Pump: A H2O 0%、B MeOH 30%、C ACN 70%

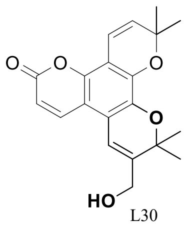

L30

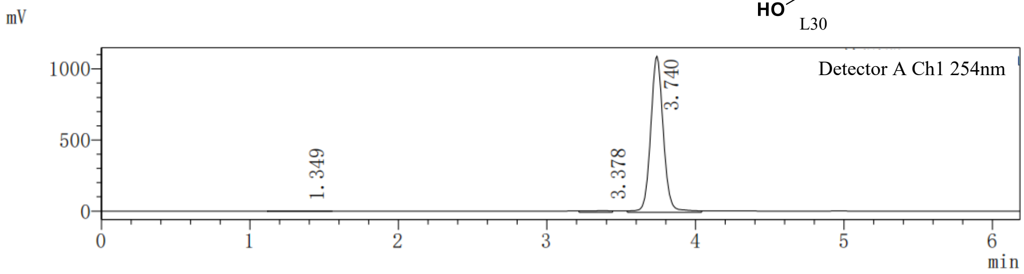

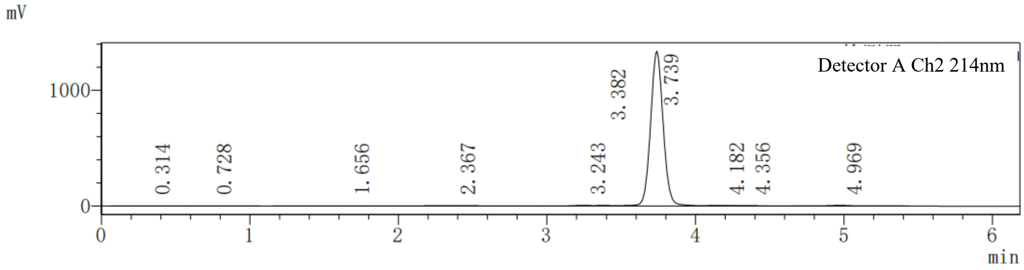

Ch1 254nm

Peak No.	R.T	Area	Area%
1	1.349	2039	0.030
2	3.378	133725	1.992
3	3.740	6576776	97.977
Total		6712540	100.000

Ch2 214nm

Peak No.	R.T	Area	Area%
1	0.314	8383	0.102
2	0.728	2727	0.033
3	1.656	10065	0.122
4	2.367	79130	0.960
5	3.243	52684	0.639
6	3.382	39165	0.475
7	3.739	7985676	96.888

8	4.182	8572	0.104
9	4.356	7080	0.086
10	4.969	48684	0.591
Total		8242166	100.000

HPLC Report

Inj: 1-4　Inj.Vol　: 1 uL
Cloumn:SHIMADZU　250　4.6　5.0 um
Detector: UVDualMode 254 nm / 214 nm
Oven: 30℃
Flow: 0.8 mL/min
Pump: A H2O 0%、B MeOH 30%、C ACN 70%

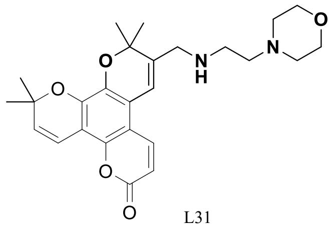

L31

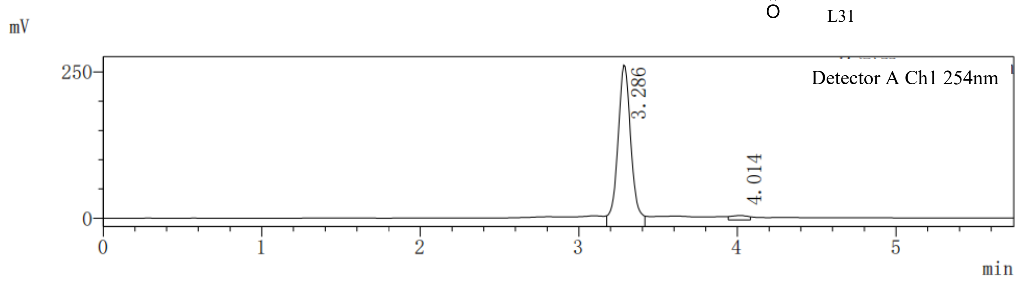

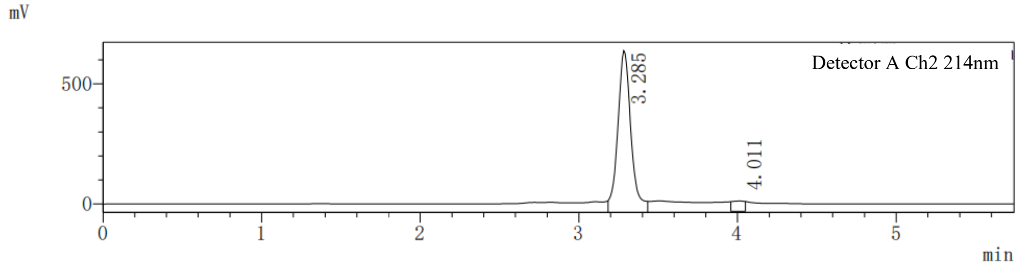

Ch1 254nm

Peak No.	R.T	Area	Area%
1	3.286	2526074	97.842
2	4.014	55725	2.158
Total		2581799	100.000

Ch2 214nm

Peak No.	R.T	Area	Area%
1	3.285	4538529	95.125
2	4.011	232615	4.875
Total		4771144	100.000

Inj: 1-4   Inj.Vol   : 1 uL
Cloumn:SHIMADZU   250   4.6   5.0 um
Detector: UVDualMode 254 nm / 214 nm
Oven: 30℃
Flow: 0.8 mL/min
Pump: A H2O 0%、B MeOH 30%、C ACN 70%

HPLC Report

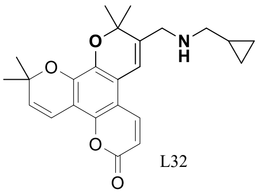

L32

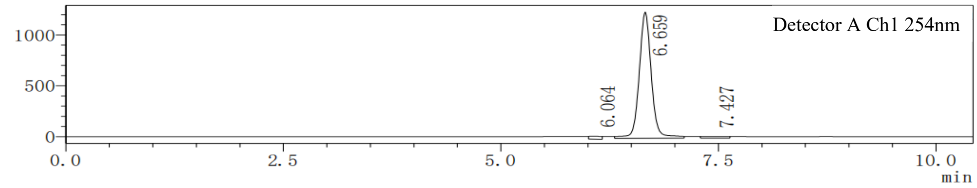

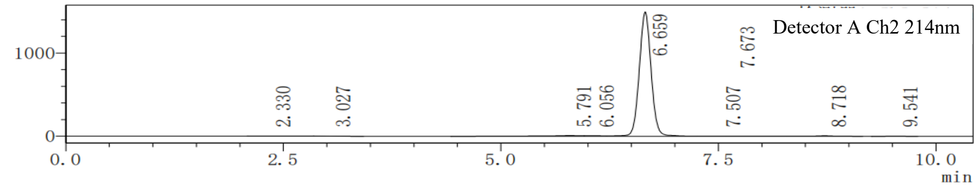

Ch1 254nm

Peak No.	R.T	Area	Area%
1	6.064	273763	2.144
2	6.659	12161204	95.253
3	7.427	332233	2.602
Total		12767200	100.000

Ch2 214nm

Peak No.	R.T	Area	Area%
1	2.330	25369	0.173
2	3.027	4542	0.031
3	5.791	266462	1.816
4	6.056	95379	0.650
5	6.659	14114920	96.192
6	7.507	38047	0.259
7	7.673	45670	0.311
8	8.718	78537	0.535
9	9.541	4804	0.033
Total		14673729	100.000

HPLC Report

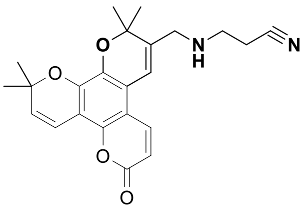

L33

Inj: 1-4 Inj.Vol : 1 uL
Cloumn:SHIMADZU 250 4.6 5.0 um
Detector: UVDualMode 254 nm / 214 nm
Oven: 30℃
Flow: 0.8 mL/min
Pump: A H2O 0%、B MeOH 30%、C ACN 70%

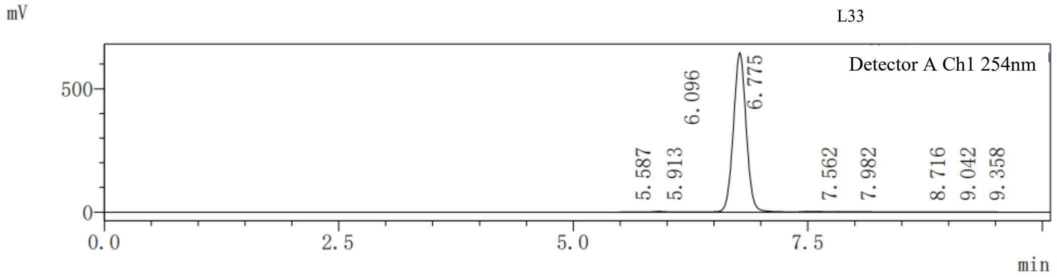

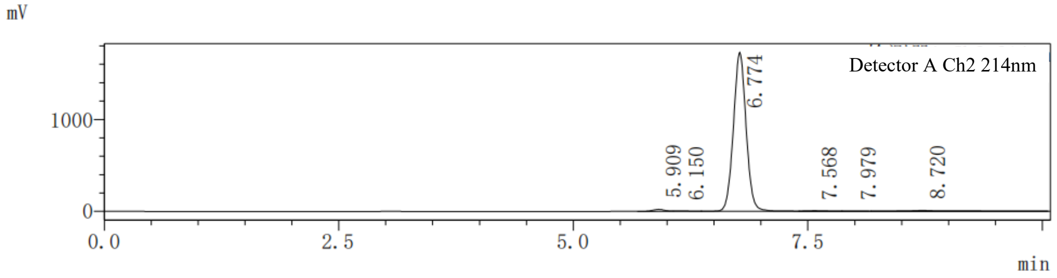

Ch1 254nm

Peak No.	R.T	Area	Area%
1	5.587	1454	0.023
2	5.913	27300	0.437
3	6.096	11066	0.177
4	6.775	6175779	98.925
5	7.562	13846	0.222
6	7.982	5848	0.094
7	8.716	4357	0.070
8	9.042	1774	0.028
9	9.358	1484	0.024
Total		6242908	100.000

Ch2 214nm

Peak No.	R.T	Area	Area%
1	5.909	208377	1.223

2	6.150	56201	0.330
3	6.774	16422966	96.424
4	7.568	47677	0.280
5	7.979	14403	0.085
6	8.720	282426	1.658
Total		17032050	100.000
